# Sequencing of human genomes with nanopore technology

**DOI:** 10.1038/s41467-019-09637-5

**Published:** 2019-04-23

**Authors:** Rory Bowden, Robert W. Davies, Andreas Heger, Alistair T. Pagnamenta, Mariateresa de Cesare, Laura E. Oikkonen, Duncan Parkes, Colin Freeman, Fatima Dhalla, Smita Y. Patel, Niko Popitsch, Camilla L. C. Ip, Hannah E. Roberts, Silvia Salatino, Helen Lockstone, Gerton Lunter, Jenny C. Taylor, David Buck, Michael A. Simpson, Peter Donnelly

**Affiliations:** 1 Wellcome Centre for Human Genetics, University of Oxford, Oxford, OX3 7BN UK; 2 Genomics plc, Oxford, OX1 1JD UK; 3 Program in Genetics and Genomic Biology and The Centre for Applied Genomics, Hospital for Sick Children, Toronto, M5G 0A4 Canada; 4 National Institute for Health Research Oxford Biomedical Research Centre, Oxford, OX4 2PG UK; 5 Department of Clinical Immunology, Oxford University Hospitals, Oxford, OX3 9DU UK; 6 Developmental Immunology Group, MRC Weatherall Institute of Molecular Medicine, University of Oxford, Oxford, OX3 9DS UK; 7 Clinical Immunology Group, National Institute for Health Research Oxford Biomedical Research Centre, Oxford, OX4 2PG UK; 8 Children’s Cancer Research Institute, St. Anna Kinderkrebsforschung, 1090 Vienna, Austria; 9 Department of Statistics, University of Oxford, Oxford, OX1 3LB UK

**Keywords:** Genome informatics, Medical genomics, Genetics research

## Abstract

Whole-genome sequencing (WGS) is becoming widely used in clinical medicine in diagnostic contexts and to inform treatment choice. Here we evaluate the potential of the Oxford Nanopore Technologies (ONT) MinION long-read sequencer for routine WGS by sequencing the reference sample NA12878 and the genome of an individual with ataxia-pancytopenia syndrome and severe immune dysregulation. We develop and apply a novel reference panel-free analytical method to infer and then exploit phase information which improves single-nucleotide variant (SNV) calling performance from otherwise modest levels. In the clinical sample, we identify and directly phase two non-synonymous de novo variants in *SAMD9L*, (OMIM #159550) inferring that they lie on the same paternal haplotype. Whilst consensus SNV-calling error rates from ONT data remain substantially higher than those from short-read methods, we demonstrate the substantial benefits of analytical innovation. Ongoing improvements to base-calling and SNV-calling methodology must continue for nanopore sequencing to establish itself as a primary method for clinical WGS.

## Abstract (web summary)

Nanopore sequencing technology generates longer reads than current technologies, but with more errors. Here, the authors develop new analytical tools to improve accuracy and evaluate the potential of nanopore sequencing for clinical human genomics.

## Introduction

Since the Human Genome Project’s 13-year effort to sequence the human genome using dideoxy chain-termination (Sanger) sequencing^1,2^, technological developments have been crucial in enabling current population-scale human genome sequencing endeavours in research and the clinic (www.nhlbiwgs.org and www.genomicsengland.co.uk). The sequencing-by-synthesis methodology initially developed and commercialised by Solexa, and progressively improved in both accuracy and throughput by Illumina, has transformed the study of the human genome for research and clinical applications^3,4^. Despite such advances, short-read lengths restrict the insight that can be derived from sequencing of an individual genome by limiting the resolution of repetitive regions, complex structural variation, and haplotype phase. The single-molecule sequencing platform developed by Pacific Biosciences of California Inc is capable of reads longer than 10 kb^5,6^, providing an advantage in accessing challenging repetitive regions of the genome^7^ and in phasing and the detection of complex structural variation^8^, but relatively high cost and low throughput have so far limited the technology’s adoption.

All major sequencing technologies so far have been built around modifications of polymerase-mediated DNA synthesis. In contrast, nanopore-based sequencing represents a radically different approach, in which the sequence of nucleic acids is inferred from changes in the ionic current across a membrane as a single DNA molecule passes through a protein nanopore^9,10^. Nanopore sequencing has been commercialised in the form of Oxford Nanopore Technologies’ handheld MinION device, and deployed extensively for sequencing bacterial and viral genomes^11,12^. Until recently the relatively low throughput of the instrument has limited its use for interrogation of the human genome to the sequencing of targeted regions^13–15^. However, recent advances, which both increase the accuracy at which the DNA sequence passing through the protein nanopore can be determined and the speed at which DNA can pass through it, have dramatically increased the throughput of the instrument, making high-coverage sequencing of larger genomes including the human genome feasible.

Initial reports of nanopore human genome sequencing have focused on the benefits of Oxford Nanopore’s long reads in achieving a highly contiguous assembly^16^ or in identifying structural variation in patient samples^17^. This focus is unsurprising, considering the potential difficulties presented by the modest per-base accuracy of Oxford Nanopore reads in attaining the low genome-wide error rates for single-nucleotide variant (SNV) calling that will be required for most clinical applications of genome sequencing using a single technology.

To evaluate the potential of nanopore sequencing for clinical human genomics we have sequenced two human genomes across multiple runs of the portable MinION device. We have resequenced the genomic reference sample NA12878, which has been extensively studied by multiple sequencing technologies, to evaluate and calibrate variant-calling approaches at increasing sequencing depth. We then sequenced DNA from an individual with ataxia-pancytopenia syndrome accompanied by severe immune dysregulation in order to fully resolve a question relating to the phasing of two de novo protein-coding variants that is relevant for a complete molecular genetic diagnosis.

## Results

### Whole-genome sequencing of a reference sample using MinION

**Fig. 1:**
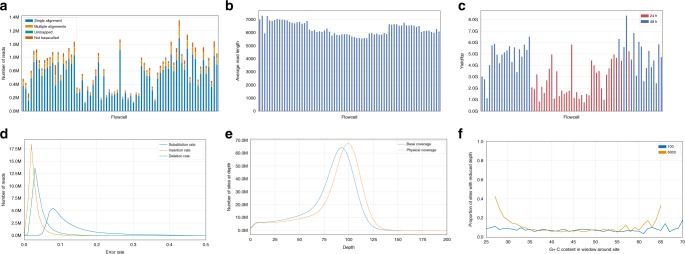
Characteristics of sequencing using ONT for NA12878. **a** Yield per flow cell, with flow cells organized left to right by run date. The total size of the bar represents the number of reads from each flow cell and is split into the proportion of reads that have been mapped in a single alignment (single alignment), mapped in multiple alignments (multiple alignments), have been base-called, but not been mapped (unmapped) and reads that have not been base-called (not basecalled). **b** Average read length per flow cell. **c** Yield (base pairs) per flow cell. **d** Distribution of per-read substitution, insertion and deletion error rates in the high-quality read set. **e** Distribution of genomic coverage. **f** Proportion of sites with a read depth less than 40 binned by G + C content of the surrounding window. Shown are read depth in windows of size 100 bp and 6000 bp

DNA from the reference cell line GM12878 (a lymphoblastoid cell line generated from a female CEPH/Utah individual) obtained commercially (Coriell Institute, sample NA12878) was prepared for sequencing using a PCR-based library protocol that included size selection of fragments of approximately 6 kb (Methods). Libraries were sequenced across 73 individual R9.4 flow cells on 8 MinION instruments, generating a total of 45,740,123 reads (Fig. 1a, Supplementary Table 1), that were base-called using Albacore v2.0.2 and trimmed using Porechop 0.2.2 (Methods). A total of 3,224,356 low-quality reads were discarded by the base-caller (2.6% of total yield, Supplementary Figure 1) and bases were trimmed (1.7% of total yield). The mean read length of 6373 bp (Fig. 1b) was consistent across flow cells and very close to expectation based on the physical size selection of the sequencing library. The total sequence yield was 273.4 Gb with a mean of 3.7 Gb per flow cell (Fig. 1c). The recommended MinION run-time was 48 h; some runs were terminated at 24 h for technical reasons including cases where relatively few pores were producing data and so starting a new flow cell was expected to substantially accelerate data production. Flow cells run for 48 h generated a median 5.0 Gb and runs stopped after 24 h yielded a median 2.1 Gb. Of a total 42,924,782 high-quality reads, 42,631,376 (99.3%) were successfully aligned to the GRCh37 reference genome assembly and 37,859,481 (88.8%) mapped uniquely and in a single block. Base calling took ~3100 CPU hours and mapping of the high-quality reads took ~500 CPU hours on a cluster comprising Intel(R) Xeon(R) CPU E5-2680 v3 2.50 GHz CPUs.

The aligned reads have a mean substitution rate of 12.7% (the frequency at which an aligned base in a read is different from the reference base), a mean deletion rate of 4.7% (the frequency at which a base in the reference sequence is absent within a read aligned to that sequence), and a mean insertion rate of 3.2% (Fig. 1d). Error profiles between flow cells were similar, with two outliers (Supplementary Figure 2). We also evaluated the impact of different base-calling algorithms on read-level accuracy, and found that Albacore v2.0.2 achieved the lowest unfiltered substitution error rate and deletion error rate, while other methods had lower insertion error rates (Supplementary Table 2, Supplementary Figures 3–5).

The average per-base coverage depth (excluding deletions) was 81.7 (Fig. 1e), with 90.4% of the genome covered by at least 40 reads. Physical coverage (per-read, with deletions) was higher, with an average depth of 88.3 and 91.7% of the genome covered to at least 40×. Further, 99.9% of the genome was covered by at least 1 read and 69.4% of the genome had a per-base coverage between 40 and 100. A subset (9.6%) of the genome had reduced coverage (<40×), which may reflect amplification bias in the PCR step of the library preparation protocol (Fig. 1f).

### Single-nucleotide variant discovery in NA12878

We evaluated the performance of SNV calling in NA12878, using the multi-platform Genomes in a Bottle (GIAB) variant calls as a gold standard truth data set^18^. The relatively high per-base, per-read error rate of ONT data yields one or more candidate variants at most positions in the genome, overwhelming most variant callers designed for sequencing technologies with lower error rates. We used FreeBayes in a mode robust to this issue for rapid per-site SNV calling (Methods)^19^. To generate an initial set of variants we ran FreeBayes on the NA12878 chromosome 22 data, choosing parameters that achieved the best F_1_ score, a measure of accuracy that combines estimates of the sensitivity and precision of classification of a sample (Methods). The highest-accuracy variant call set achieved an overall consensus accuracy across the whole genome of 99.9%, in comparisons to the GIAB reference set of variant calls, and we observed a false discovery rate (FDR) of 12.8% and a false-negative rate (FNR) of 14.4%, combining to create an F_1_ score of 86.4% (Table 1). Using these learned parameters on the full-genome set we achieved an FDR of 10.9%, a FNR of 12.5% and an F_1_ score of 88%. Variant calling on the full genome took 83.4 CPU hours on Intel(R) Xeon(R) CPU E5-2680 v3 2.50 GHz CPUs.

**Table 1:** SNV discovery and filtering

Filtering approach	Chromosomes	F1 score	FDR	FNR
QUAL + contamination	22	86.4%	12.8%	14.4%
	All	88.3%	10.9%	12.5%
Phasing + heuristics	22	91.8%	7.9%	8.5%
	All	93.4%	5.3%	7.8%

Filtering approaches are either pre-phasing, optimising over contamination parameters in FreeBayes and QUAL score, or post-phasing, optimising over use of phasing metrics given a fixed QUAL score, strand bias, and contamination parameters. Shown are both results for chromosome 22 on which parameter cutoffs were derived, as well as for the full set of autosomes

**Fig. 2:**
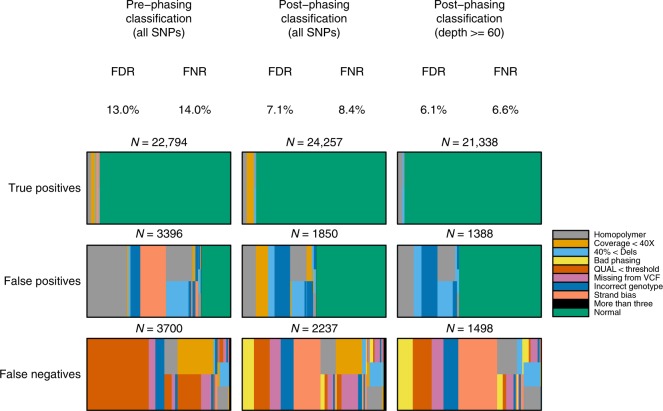
Investigation of residual errors in NA12878 data. Annotation of called or truth SNPs using genomic features or sequencing context in NA12878. Results across columns give the different sets of SNPs, either pre or post-phasing, and for post-phasing, optionally all SNPs or those at high local depth ( > = 60× coverage). Results across rows give SNP classes of true positives, false positives and false negatives. Bars are broken horizontally to reflect multiple possible annotations, while vertical splits represent SNPs with multiple annotations. Annotations are: homopolymer, SNP intersects a homopolymer of length at least 5 bases; Coverage <40×, per-base coverage of less than 40×; 40%<Dels, at least 40% of reads have deletions at that SNP; Bad phasing, the quality control phasing metric for that SNP was below threshold; QUAL < threshold, the quality score for the SNP was below threshold; Missing from VCF, there was insufficient evidence from the variant caller to put this variant in the output VCF; Incorrect genotype, disagreement between truth and called genotype; Strand bias, Freebayes metrics SRP or SAP < 30; More than three, more than three of the above annotations; Normal, no annotations

To better understand potential sources of variant-calling errors, we annotated variant call sites with a range of annotations with respect to both the reference sequence and reads spanning the site. Among others, these included proximity to a homopolymer repeat, lower coverage, strand bias and presence of a large number of short, in-read deletions (Fig. 2a). These annotations indicate that the main drivers for both false positives (FP) and false negatives are homopolymers and low coverage. In addition, many false negatives arise from the use of a high-quality score threshold (QUAL) to maintain an acceptable FDR.

A large fraction of false-positive genotype calls in the initial variant set are heterozygous calls. A benefit of using ONT data is that the long reads, spanning multiple heterozygous sites, provide an opportunity to correct this problem. When reads are divided into two sets representing parental haplotypes, true-positive variant alleles are expected to be present consistently in one or the other phased set, while false-positive variants are expected to be uniformly distributed between sets. To cope with high-coverage data, we developed our own single-sample, read-based, reference panel-free phasing algorithm (Supplementary Note). This method builds on the STITCH model for genotype imputation^20^ and models an individual as having two haplotypes (with no recombination), where the probability of a reference or alternate base for a read drawn from a haplotype is governed by per-haplotype, per-SNP emission probabilities, which we convert into a per-variant phasing-quality metric (Supplementary Note).

We refined our variant calls using filters informed by our investigation of annotations associated with incorrect variant calls, as well as the phasing-quality metric. We found that phase- and annotation-based filtering substantially improved upon the original approach (Table 1, Supplementary Table 3, Supplementary Figure 6). The best results with strand and phasing filters yielded an F_1_ score of 92.2% with FDR of 7.1% and FNR of 8.5% (Table 1, Fig. 2b).

Further annotation of residual errors shows that, as before, coverage remains a powerful predictor of error. When considering putative variant sites with greater than 60× coverage (85% of the genome), we saw an improvement to an F_1_ score of 93.6%, consisting of an FDR of 6.1% and an FNR of 6.6% (Fig. 2c), implying that protocol improvements that reduce or eliminate sources of coverage bias such as PCR have some role to play in improving accuracy. Among residual errors, FPs are enriched in homopolymers and regions of low read depth, and we also find evidence for base-calling and reference-alignment bias (Supplementary Figures 7–9). Remaining FPs without obvious annotation often show evidence that they are impacted by one or more of the confounding annotations, but are not filtered at the parameter values used here, which attempt to balance sensitivity and specificity. By contrast, false negatives reflect a wide variety of erroneous filtering instances that span all classes of confounding annotations, including homopolymers and low depth.

The observed FDR of 5.3% on the whole genome corresponds to 140 thousand FP variant calls. While true-positive sites, representing real genetic variation, are constrained in their genomic location through evolution, FP calls occur more or less independently of genic context. As such, FP variant calls may be disproportionately enriched in disruptive variants, in particular putative loss-of-function (LoF) alleles, which are often the most clinically interesting variants. This problem of false enrichment should be further exacerbated by genic tolerance to LoF mutations^21^. To investigate the present impact of FDR on putative pathogenic FP rates, we annotated variants as LoF based on either introducing a stop-gain mutation or affecting splice site (Methods). We observed that among putative pathogenic variants, FPs are enriched (69/45219, 0.15%) versus true positives (173/788782, 0.02%) (Supplementary Table 4, Supplementary Figure 10). Similarly, we observed that FPs are proportionally enriched in highly constrained genes (pLI > 0.90, 17 FP vs 20 TP) versus non-constrained genes (pLI < = 0.10, 46 FP vs 122 TP). Nonetheless, and reassuringly, though enriched, the absolute number of FPs (20) is similar to true positives (17) among putative high impact LoF variants at the current FDR.

To investigate whether alternative base-callers could yield more accurate SNV calls, we compared SNVs called using reads base-called using Albacore 2.0.2 against three other base-callers (Methods). Interestingly, we observed slightly more accurate results on unfiltered variant calls using the now-discontinued base-caller Metrichor (Supplementary Figures 11-13, Supplementary Table 5), while Albacore again outperformed Metrichor and the other base-callers when phasing-derived filtering was applied (Supplementary Table 6, Supplementary Figure 14). This suggests that base-callers are affected by kmer biases to different degrees, complicating the relationship between per-read error rates and variant-calling performance.

To further clarify the impact of systematic biases in nanopore data, we simulated NA12878 datasets under an idealized model of random per-read base-substitution errors and no genome amplification bias, with read lengths and ratios of substitution/deletion/insertion error rates informed by observed data from a single flow cell. We evaluated variant-calling performance across error rates (Supplementary Figure 15) and sequencing depths (Supplementary Figure 16). At similar depth and error rate to the Albacore v2.0.2 results, simulations indicate an achievable F_1_ score of 99% with an FDR and an FNR of 1% (Supplementary Figure 17). The difference of 10 percentage points between simulated data and observed results in the actual data again indicate that systematic errors dominate. The simulations show that under idealized conditions, but with the current available sequence analysis tools, both FNR and FDR decrease as coverage increases but eventually reach a plateau with the height of the plateau determined by the error profile (Supplementary Figure 17). We traced this failure to converge to perfect FNR/FDR under the idealized error model to alignment artefacts at homopolymers driven by the high deletion error rate in ONT. For instance, reads tend to have shorter homopolymer runs than the reference, so that deletion errors in homopolymers accumulate resulting in deletion variant call errors (Supplementary Figures 18-19), which contrasts with simulated datasets dominated by insertions (of any base), in which individual-read errors are not compounded by alignment artefacts (Supplementary Figure 20). These simulations indicate that the effect of increased sequencing coverage in reducing homopolymer-associated FDRs in nanopore sequencing is currently limited by high genome-wide, per-read deletion rates.

### Phasing in NA12878

Fundamentally, genotypes are inherited through maternal or paternal haplotypes, yet most genotyping methods, including most methods employed with WGS, generate unphased genotype calls. Phased genotypes have value in their own right, enabling many genetic analyses in addition to the improvements in variant-calling accuracy they facilitate. Clinical uses include resolving the co-segregation of multiple heterozygous LoF variants and identifying parent of origin for de novo mutations.

We phased sample genotypes from all base-callers using both WhatsHap^22^ and the novel phasing algorithm described in this paper. We judged the accuracy of phasing by considering the switch error rate, namely the percentage of connections between heterozygous sites that contain a phase-switch error relative to the truth data set of NA12878 that was externally phased using parental samples NA12891 and NA12892. Considering all SNVs on chromosome 22, we observe a switch error rate of 1.91% for WhatsHap and 1.84% for our method. Restricting the analysis to sets of adjacent heterozygous SNPs that are spanned by overlapping reads, the switch error rate reduces to 0.90% for WhatsHap and 0.80% using our method (Supplementary Table 6). This phasing accuracy is similar to that obtainable for phasing common variants from SNP genotyping array data using very large reference panels^23^.

### Large variant discovery in NA12878

Compared to SNVs and small indels, large variants, arbitrarily defined here as those affecting > 100 nucleotides, are relatively rare yet contribute disproportionately to rare disease phenotypes, e.g. ref. ^24^. The impact of large variants on rare disease is likely even greater than currently estimated, because of the technical difficulty of detecting these mutations with existing assays^24^. We used Sniffles^25^, a method that exploits the signature of large variants in aligned ONT reads. We applied this method to chromosome 22, and compared the resulting calls against a truth set of large variants derived from a consensus of several sequencing technologies^26^. Of the 82 variants we discovered, 22 were also found in the truth set. We adjudicated the remaining 60 calls by visual inspection of ONT, Illumina and PacBio read data. We find that 21 are either called by PacBio or are strongly supported by PacBio reads. A further 31 show clear evidence in ONT reads but weak or no support in PacBio reads. These ONT-specific calls may represent true deletions missed by other technologies, artefacts resulting from PCR amplification, or subclonal deletions that have occurred during the cell culture of the NA12878 cell line. Indeed, 6 of the ONT-specific large deletion calls appear to be supported in only a fraction of the reads (Supplementary Figures 21 and 22). The remaining 8 calls show no clear evidence in the data and appear to be FPs generated by the algorithmic approach.

We next sought to assess sensitivity. To reduce the impact of false negatives in the truth set, we focused on deletions only, which are the most abundant and thus clinically relevant subclass of large variants, and are relatively easy to call. Of the 35 deletions over 100 bp in the truth set, we call 21. Of the remaining variants, 11 show evidence of a deletion but the method fails to call it (Supplementary Figure 23), while the remaining three calls show no evidence in ONT data (Supplementary Figure 24). We conclude that the current ONT platform allows the detection of large deletions with a sensitivity between 60% and 91% (21/35 and 32/35).

### Whole-genome sequencing of a clinical sample using MinION

Given the ability to successfully phase heterozygous variant calls with long reads, we sought to use whole-genome nanopore sequencing to resolve a question of clinical interest in the genome of an individual with an undefined immunodysregulatory disorder. In brief, the female patient had initially presented in infancy with recurrent infections, panhypogammaglobulinaemia, thrombocytopaenia, and mild anaemia and had developed chronic inflammatory conditions during childhood and progressive neurological symptoms in early adulthood [Supplementary Material for more detail].

Whole-genome sequencing of the patient and her parents using Illumina 126-base, paired-end reads was used first to exclude variants in several genes known to cause antibody deficiency or cerebellar atrophy. Subsequent analysis revealed 84 high-confidence de novo SNVs, a figure towards the upper end of the expected range, in line with the parental ages at conception (mother was 38 years old and father 39 years old) [see Fig. 1 in ref. ^27^]. Notably, of the 3 of 84 de novo variants predicted to alter protein sequences, two lay in the protein-coding region of a single gene, *SAMD9L*. Rare inherited heterozygous variation in this gene has recently been implicated in autosomal dominant Ataxia-pancytopenia syndrome (OMIM: #159550)^28^ and there is emerging evidence that postnatal reversions in haematopoietic tissues may be associated with milder disease manifestations^29^. Although the two variants we identified (c.1076 G > A and c.3353 A > G; p.R359Q and p.Y1118C, NM_152703.3) lie in the same exon, they are 2277 bp apart and so could not be directly phased using the Illumina paired reads. A paucity of nearby inherited heterozygous variants also prevented us localizing the mutations to parental haplotypes. Resolving these questions is important in interpreting the pathogenic potential of each allele, and the ability to resolve questions of this type is of direct relevance to reproductive decision-making in similar situations.

Sequencing libraries were constructed from whole-blood genomic DNA and sequenced across 34 R9.4 MinION flow cells. Base-calling using Albacore v2.0.2 generated a total of 122 Gb of sequence in 16,692,656 high-quality reads (16,692,656 reads before filtering and trimming), of which 16,684,879 (99.1%) fragments aligned to the reference genome. Results at the flow cell level were similar to NA12878 in PASS/FAIL fraction (Supplementary Figure 25), yield (Supplementary Figure 26), average read length (Supplementary Figure 27), fraction of mapped reads (Supplementary Figure 28), substitution error rate among pass reads (Supplementary Figure 29), and the genome-wide distribution of coverage (Supplementary Figure 30). Variants were called using FreeBayes with contamination parameters 0.7/0.1, and phased and filtered in the same way as the best-performing Albacore v2.0.2 SNP callset for NA12878.

**Fig. 3:**
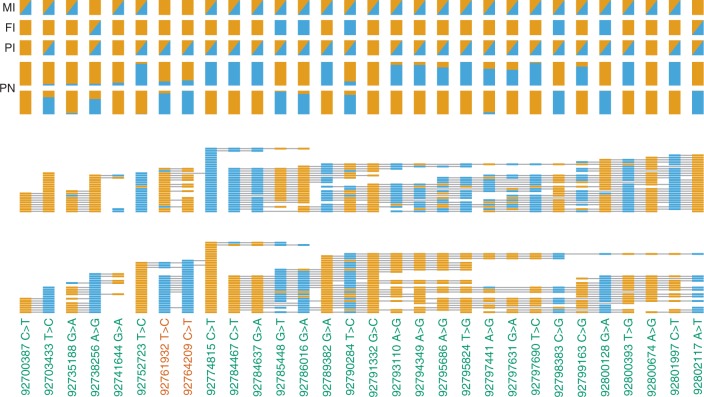
Phasing clinical sample using ONT. Top of figure shows Illumina unphased genotypes for the mother (M I), father (F I) and proband (P I), as well as phased genotypes for the proband using ONT, at bi-allelic PASS SNVs identified by Illumina sequencing that have a heterozygote genotype in at least one member of the trio. Unphased genotypes are represented with triangles in boxes where blue = alt and orange = ref. Phased proband genotypes (P N) are represented by two rows of vertical bars, where each row is an arbitrarily labelled haplotype, and each bar is split by colour according to the probability of that haplotype having reference or alternate base. Middle of figure shows two rows with the reads for haplotype 1 or haplotype 2, where for each read, bases are rectangles, and read span is given by a horizontal line. Gaps represent either a gap (deletion), or a base that corresponds to neither the reference nor the alternate allele. Bottom shows physical position, with sites of interest in red. Note that some of the phase set containing the sites of interest extends another 150 kb distally but is not shown in the interests of clarity. Based on GRCh37 and NM_152703.3, 92761932 T > C corresponds to c.3353 A > G whilst 92764209 C > T is corresponds to c.1076 G > A

Our variant-calling approach identified both the c.1076 G > A and c.3353 A > G variants with the expected heterozygous genotypes. We then phased other nearby variants identified in short-read data using the ONT reads to confirm inheritance and origin of the de novo variants (Fig. 3). Both de novo sites were phased into a phase set 199 kb long. Within this block, 33 reads (size range 6.1–18.9 kb) spanned both de novo variant sites, of which 26 had a base at each of the two de novo sites that matched either the expected reference or the alternate allele. Eleven of these reads contained both de novo alleles and eight contained both reference alleles, indicating that the mutated alleles are *in cis*. For comparison, we observed no read in the NA12878 data that contained both de novo variant alleles out of 89 reads spanning both sites. Seven observed reads from the patient were inconsistent with the *in cis* pattern; four with the apparent de novo allele at the c.1076 G > A site and the reference allele at the c.3353 A > G site and three vice versa, however two of these reads had a higher-than-average substitution rate (24% and 25%, Supplementary Figure 31). The haplotypic conformation of de novo alleles from the ONT reads was confirmed using a series of allele-specific PCR assays (Supplementary Figure 32). Flanking sites in the phase block indicated that the de novo variants arose on the paternally inherited haplotype (Fig. 3).

## Discussion

In this work, we demonstrate the sequencing of the entire human genome on the handheld MinION nanopore sequencing device. Sequencing the reference sample NA12878 to high coverage across 73 flow cells enabled the evaluation of variant-calling approaches in comparison to the GIAB reference data set. We used a PCR-based protocol partly out of necessity, since native library preparation protocols could not produce the amount of library we needed from the available quantity of the clinical-sample DNA, but also, and critically, because relatively small amounts and shorter fragments of DNA reflect the reality of current DNA sampling pathways. Thus our evaluation in the reference sample reflects a realistic assessment of nanopore sequencing of existing clinical DNA samples. Despite per-read error substitution rates of about 13% and deletion rates of about 5%, the consensus accuracy of the 82 × genome was above 99.9%. However, there remain substantial hurdles to accurately identifying and genotyping sites in the genome that harbour non-reference alleles. Many key components for processing ONT sequencing data are under active methodological development, including base-calling from raw signal, long-read mapping and alignment, variant calling and variant filtering. We evaluated four different base-callers in a straightforward approach to variant calling that only evaluates base calls at each site of interest. While similar approaches were originally used for short-read variant callers, e.g., GATK UnifiedGenotyper^30^ and Samtools^31^, improvements in accuracy have since come from the use of local re-alignment or assembly methods such as FreeBayes^19^, Platypus^32^, and GATK HaplotypeCaller^30^. These refined approaches are currently computationally prohibitive for nanopore sequencing data’s per-read error rates and read lengths.

Our analyses identified a series of sequence contexts and coverage parameters in which both false-positive and false-negative calls were made, and used this information to refine our variant call sets. Many false-negative and false-positive variant calls overlapped regions with a large number of within-read deletions, reflecting a mix of homopolymers and local mapping problems. Interestingly, we note a poor relationship between per-read accuracy and SNP-calling accuracy, illustrating the importance of developing new methods with primary endpoints, such as variant calling or assembly, rather than surrogate endpoints, such as read-level error rates. Indeed, for methods such as Metrichor, which produced the most accurate variant call set, residual SNP-calling errors are dominated by homopolymers, whereas by contrast Scrappie, a method that attempts to model homopolymers explicitly, has fewer homopolymer SNP-calling errors among a larger number of errors overall. Further improvements may come from bringing together the best aspects of multiple approaches. While we focused on reducing SNV error rate, the effect of deletions in reads suggests that without improvements in raw signal or in base calling, even greater sequencing depths will be required to reliably genotype short deletions.

We also exploit the length of nanopore reads to reduce the rate of false-positive heterozygous variant calls. We developed a novel phasing algorithm to distinguish between real variants that phase consistently with their neighbours, and false variants that segregate randomly. This method, which becomes increasingly powerful as read length increases, greatly decreased FPs with only a modest increase in false negatives, the latter of which may be mitigated through approaches such as iteratively removing the most poorly phasing SNPs to reduce the influence of multiple false-positive SNPs on phasing of true positives, and pass through of heterozygous SNPs spanned by reads that do not span additional heterozygous variants. Crucially, this method has linear computational scaling in read depth, allowing the full complement of reads to efficiently inform read partitioning and genotyping, in contrast to other approaches under development^33^. Recent work by Jain et al has demonstrated that with appropriate DNA extraction and library preparation protocols, read lengths from human genomes in excess of 100 kb are feasible^16^. Such long reads will undoubtedly further increase the utility of phasing-informed variant calling and filtering through more confident read assignment to haplotypes, and increasing the per-heterozygous variant depth of read coverage to adjacent heterozygous variants.

We sought to directly explore the clinical utility of nanopore sequencing technology for an individual with an undefined immunodysregulatory disorder. In humans and other eukaryotes, multi-nucleotide variants (MNVs) such as the two missense mutations, identified 2277 bp apart in *SAMD9L*, make up around ~3% of all de novo SNVs^34,35^. The variant-calling strategy optimised through our analysis of NA12878 was able to identify and correctly genotype the two de novo *SAMD9L* variants, and long-read phasing unambiguously confirmed that the novel alleles lay *in cis* on the paternally inherited haplotype, a feat unachievable with short reads. The functional impact of the p.R359Q and p.Y1118C variants remains an open question, although several lines of evidence suggest that p.Y1118C is the more likely disease-causing allele: p.Y1118C is a novel variant whereas p.R359Q is present in gnomAD (allele frequency of 2/245,750), p.Y1118C scores higher with in silico prediction tools such as CADD (23.1 vs 10.9) and SIFT (damaging vs tolerated), and published disease-causing mutations in *SAMD9L* are almost invariably in the C-terminal half of the protein^28,29,36–38^. Apart from features specific to this case, the ability to phase de novo alleles in a single step has substantial potential utility, since an estimated 42% of developmental disorders are caused by de novo alleles^39^ whose resolution onto parental haplotypes can inform estimates of recurrence risk^27,40^.

This study represents the first detailed evaluation of the accuracy of ONT sequencing for variant discovery and genotyping on human samples. While promising, the current computational requirements would be overwhelming at higher throughputs, and work is required to implement more efficient algorithms for base calling, mapping and variant calling, or to establish implementations on dedicated hardware. Sequencing of these two human genomes across a combined total of 107 MinION flow cells was a substantial undertaking that entailed logistical, technical, and computational challenges. The recent commercial introduction of the PromethION, a scaled-up nanopore sequencer with on-board data processing, promises to solve many of these challenges for human genome-scale data. Finally, while there remain limitations to the overall accuracy of variant calling, our work highlights several error contexts that would benefit from improvements in methods for base calling, read mapping and consensus variant calling and illustrates a path towards the use of ONT for clinical purposes.

## Methods

### Samples and library preparation

The genomic DNA sample NA12878 from the GM12878 lymphoblastoid cell line genomic DNA used in this study was purchased from the Coriell Institute. The study complied with all relevant regulations for work with human subjects. The patient and her biological parents provided informed consent and were recruited to our study Molecular Genetic Analysis and Clinical Studies of Individuals and Families at Risk of Genetic Disease (West Midlands REC, 13/WM/0466). Patient DNA was extracted from a whole-blood sample.

ONT libraries were prepared as follows: For each of several batches of libraries, 2–4 μg in 150 μl of genomic DNA per library was sheared in a Covaris g-TUBE by spinning twice for 2 min at 7000 rpm in an Eppendorf MiniSpin centrifuge. The sample was split into three 50 μl aliquots, each mixed with 6.5 μl of NEBNext FFPE DNA Repair Buffer, 2 μl of NEBNext FFPE DNA Repair Mix (New England Biolab, M6630), and 3.5 μl of nuclease-free water (NFW) and incubated at 20 °C for 15 min for DNA repair, re-pooled and cleaned up using a 0.8 × volume of AMPure XP beads (Beckman Coulter) according to the manufacturer’s instructions, with final elution in 30 μl of EB (10 mM Tris pH 8.0). The size of the sheared DNA was assessed using a TapeStation Genomic DNA system (Agilent).

To remove small, unwanted fragments of DNA, the sample was size-selected using a BluePippin™ gel cassette (BLF7510, Sage Science) using the 0.75% DF Marker S1 high-pass 6–10 kb vs3 cassette definition, with Range mode and BP start set at 6000 bp. The DNA recovered from the elution well (~40 μl) was brought to 50 μl and end-repaired by the addition of 7 μl NEBNext Ultra II End Prep Reaction Buffer and 3 μl NEBNext Ultra II End Prep Enzyme Mix (New England Biolab, E7546) with incubation for 30 min at 20 °C followed by 30 min at 65 °C. The sample was cleaned up with 1 × volume AMPure XP beads and eluted in 30 μl of EB.

PCR adapters (20 μl) from Oxford Nanopore Technology (SQK-LSK108 Ligation Sequencing Kit 1D) were ligated to the end-repaired DNA with 50 μl of NEB Blunt/TA Ligase Master Mix (M0367) at room temperature for 10 min, followed by clean-up with 1 × volume AMPure XP beads and elution in 48 μl of EB.

The DNA was amplified by adding 2 μl of the primer mix PRM (SQK-LSK108, ONT) and 50 μl of KAPA HiFi HotStart ReadyMix (Kapa Biosystem) and thermal cycling as follows: 95 °C 3 mins; 8 × (98 °C 20 s, 64 °C 15 s, 72 °C 10 min); 72 °C 10 min.

Amplified samples were cleaned up with 0.4 × volume AMPure XP, then eluted in 40 μl of EB and quantified using the Qubit dsHS DNA assay (Thermo Fisher Scientific). A mass of 1–1.5 μg DNA in 50 μl was end-repaired by adding 7 μl NEBNext Ultra II End Prep Reaction Buffer and 3 μl NEBNext Ultra II End Prep Enzyme Mix), incubated for 30 min at 20 °C followed by 30 min at 65 °C, then cleaned up with 1 × AMPure XP beads and eluted in 30 μl of EB.

The end-repaired DNA was ligated with 20 μl Adapter Mix (SQK-LSK108, ONT) using 50 μl NEB Blunt/TA Master Mix for 10 min at room temperature. The adapter-ligated DNA was cleaned up by adding a 0.4 × volume of AMPure XP beads and incubating at room temperature for 5 min. The beads were pelleted on a magnetic rack and the pellet was washed twice by resuspending in 140 μl ABB (SQK-LSK108, ONT) then, after removal of the final wash, resuspended in 25 μl ELB (SQK-LSK108, ONT) and left at room temperature for 10 min. The beads were pelleted again and the supernatant containing the pre-sequencing mix (PSM) was recovered.

The PSM was quantified by Qubit. Each R9.4 flow cell (FLO-MIN106, ONT) was primed according to the manufacturer’s guidelines before loading with a mix containing 12 μl of PSM, 37.5 μl RBF (SQK-LSK108, ONT), and 25.5 μl LLB (SQK-LSK108, ONT). The flow cell was mounted on a MinION Mk 1B device (ONT) for sequencing with the MinKNOW versions 1.1.15–1.1.21 NC_48Hr_Sequencing_Run_FLO-MIN106_SQK-LSK108 script.

### Base calling

Reads were base-called in batches using Albacore v2.0.2 using the R9.4 450 bps linear config. Reads from each flow cell were merged and mapped against the human genome reference assembly (1000 Genomes GRCh37 including decoy sequence and phage lambda) using bwa mem -x ont2d version 0.7.12-r1039^41^.

For comparisons of different base-calling strategies, we used an earlier set of reads which were all base-called with Nanonet v2.0.0 and mapped to the GRCh37 genome. From this data set, we selected reads mapping to chromosome 22 and extracted the corresponding MinION reads from the original Fast5 data set. The Fast5 was then re-called using three alternatives to Nanonet v2.0.0: Albacore v2.0.2, Metrichor v2.43.1 and Scrappie v0.2.2. Albacore v2.0.2 was run using the R9.4 450 bps linear config; Scrappie v0.2.2 was run in default configuration; and Metrichor was run using the 1D workflow. After base calling, the reads in each data set were re-mapped against the human genome reference. For each of these 4 base-called datasets, we also evaluated 3 post-alignment filters for removing poor quality reads. The filters were unfiltered, fixed error (removing all reads with an error rate of 20%) and fixed size (removing 20% of reads with the highest error rate).

### Genomic coverage

Alignment coverage was computed using samtools mpileup^31^ including all reads (options -Q 0 -B -A). Coverage was only computed on canonical chromosomes (1–22, X, MT) excluding regions with assembly gaps of >100 bp. The effective genome size was 2,835,690,258 bases.

### Read error rate

We considered the following definitions of alignment error between a read and the reference genome. The substitution error rate e_sub_ indicates the frequency at which a reference base has been aligned to a read base and substituted by a non-reference base and is the ratio of mismatched base pairs and the total number of aligned bases. The percent identity of an alignment is given as p_id_ = 100 × (1 − e_sub_). The deletion error rate e_del_ indicates the frequency of unaligned reference bases within an alignment of the reference and a read and is computed as the number of bases in the reference sequence that correspond to gaps in the read sequence divided by the length of the aligned reference sequence. The insertion error rate e_ins_ indicates the frequency of unaligned read bases within an alignment and is computed as the number of bases in the read sequence that correspond to gaps in the reference sequence divided by the aligned read sequence.

### Variant calling

To improve run-time, alignments of reads to the reference genome were split into shorter fragments of 100 aligned read base pairs, and the resulting BAM file was used for variant calling. Freebayes version v1.0.2-16-gd466dde^19^ was run using options --no-indels --use-best-n-alleles 4 -C 5 --no-mnps --no-complex --haplotype-length 0 --pooled-continuous, where: -C 5 means a site to contain least 5 variant nucleotides before being considered a putative variant; haplotype construction and local realignment have been turned off (--haplotype-length 0); and variants are called using a simple frequency-based model (--pooled-continuous). To account for the high error rate we used the option --contamination-estimates to modify the expected reference and variant allele frequencies for heterozygous and homozygous variants. We generated variant calls by varying the first contamination parameter of p(read = R|genotype = AR) over 0.6, 0.7, and 0.8, and the second contamination parameter of p(read = A|genotype = AA) over 0.1, 0.2, 0.3, 0.4, 0.5 0.6, respectively.

### Simulations

To simulate reads, we used PBSIM^42^, Version 1.0.3 (5ae589d1)), which samples read lengths and base quality scores from user-provided data, and inserts base substitutions according to the base quality scores with a ratio of substitutions/insertions/deletions provided by the user. We ran PBSIM using options --accuracy-min = 0.40 --accuracy-sd = 0.20 and set difference-ratio to 60:35:5 for the high insertion error rate runs and 30:30:40 for the high deletion error rate runs. Depth-per-haplotype was varied from 5 to 50 in increments of 5 for overall depth between 10- and 100-fold coverage.

For input, we used mapped PASS reads from chromosome 22 for one flow cell (WTON000155), and simulated results for the two haplotypes of NA12878 separately using phased variants from the 1000 Genomes Project (Phase 3) before merging the results. To vary the error rate, base quality scores were uniformly incremented or decremented in the input read data set. For variant calling with simulated data, we used contamination parameters 07/05 throughout.

### Variant-calling accuracy

To evaluate the performance of variant calling, we follow the protocol set out in the precisionFDA challenge (see https://precision.fda.gov/challenges/truth). We used the following method from the tool RTG vcfeval version 3.5.1^43^ against NA12878 NIST/GIAB version 2.19^44^ reference data including only the SNV data and excluding all indel variants. Predicted variants are true positive (TP), if both location and genotype match a reference position. A variant location that is absent from the reference set is called a FP and a position that is a variant in the reference but absent from the predicted set is a false negative (FN). A predicted variant that has the same coordinate as a reference variant but different genotype is recorded both as a false positive and a false negative. The false discovery rate is defined as FDR = FP / (TP + FP) while the FNR is defined as FNR = FN / (TP + FN). The F_1_ measure is the harmonic mean of precision ( = 1−FDR) and sensitivity (recall) ( = 1−FNR), i.e. F_1_ = 2/(1/(1−FDR) + 1/(1−FNR)).

Variant calls from PacBio data and BAM files originate from GiaB/NIST. The BAM files were aligned by the authors using BLASR (v1.3.2). We used variant calls produced by the svclassify tool^26^ to define the set of known large variants in NA12878. Variants annotated as splice_acceptor_variant, splice_donor_variant, or stop_gained by the Variant-Effect-Predictor tool (version 94.5)^45^ were classified as putative LoF alleles and assigned the pLI score of overlapping transcripts (Exac version 0.3.1)^21^.

### Phasing

Here we briefly describe the SEW model underlying the phasing procedure by describing how we would calculate a complete data probability that includes the observed data (reads), as well as hidden parameters of which haplotype each read comes from and each underlying base given knowledge of the underlying parameters of the model. A full description including additional detail and parameter estimation using expectation and maximization steps, as well as additional heuristics, is given in the Supplementary Note.

Consider a read indexed by *r* (*R*_*r*_) that comes from haplotype *H*_*r*_ in *{1,2}* that intersects *J*_*r*_ SNPs with index *j* at positions *u*_*r,j*_, has underlying (unobserved) base *g*_*r,j*_, observed sequenced base *s*_*r,j*_ and base quality *b*_*r,j*_ (where *R*_*r*_ *=* {(*u*_*r,j*,_*s*_*r,j*,_*b*_*r,j*_)|*j* = 1,…,*J*_*r*_}). We assume that *P(H*_*r*_*)* *=* *0.5* (i.e. without further information, each read is equally likely to come from the maternal and paternal haplotypes), as well as calculate

1 $${\mathrm{P}}\left( {S_{r,j}{\mathrm{|}}G_{r,j}} \right) = \psi _{u_{r,j}}^g$$

directly from the phred-scaled base qualities and observed base (defined in the Supplementary Note), and further define

2 $${\mathrm{P}}(G_{r,j} = g|H_r,\theta ) = \theta _{u_{r,j}}^g$$

$\theta _{u_{r,j}}^g \in [0,1]$, as the probability a read has base *g* given it comes from haplotype *H*_*r*_. Then the probability of observing *S*_*r,j*_ is

3 $${\mathrm{P}}(S_{r,j}|H_r,\theta ) = \mathop {\sum }\limits_{g = 0}^1 P\left( {S_{r,j}|G_{r,j} = g,H_r,\theta } \right)P\left( {G_{r,j} = g|H_r,\theta } \right) = \mathop {\sum }\limits_{g = 0}^1 \psi _{u_{r,j}}^g\theta _{u_{r,j}}^g$$

Let *O = {R}*, *H* *=* *{H*_*r*_*}* and *G* *=* *{G*_*r,j*_*}*. Then we can calculate the full data probability by integrating over the reads and the SNPs each read intersects to yield

4 $$P\left( {O,H,G|\theta } \right) = \mathop {\prod }\limits_{r = 1}^{|O|} \mathop {\prod }\limits_{j = 1}^{J_r} P\left( {S_{r,j}|G_{r,j}} \right)P\left( {G_{r,j}|H_r,\theta } \right)P\left( {H_r} \right) = \mathop {\prod }\limits_{r = 1}^{|O|} \mathop {\prod }\limits_{j = 1}^{J_r} 0.5\psi _{u_{r,j}}^g\theta _{u_{r,j}}^g$$

For real variants, the expected value of *θ* is governed by the average per-base error and the underlying genotype at that SNP in the haplotype, while for false variants, no such relationship to phase holds. We can therefore use *θ*, or derived metrics, for quality control.

For quality control purposes, from the phasing, we define quality control metric phase entropy (PE) for SNP *t* as -log10(θ_t,1_ θ_t,2_ (1-θ_t,1_)(1-θ_t,2_)). Similarly, we define phasing-derived strand metrics by first assigning each read to the haplotype for which it has the highest posterior probability of having come from given the data, and then calculate the per-haplotype strand bias (SB1 or SB2) as the sum of reads assigned to that haplotype coming from the forward strand.

We used SEW version 1.0.0 and whatshap version 0.13 + 2.gf854c0b.dirty.

### Reporting summary

Further information on experimental design is available in the Nature Research Reporting Summary linked to this article.

## Supplementary information

Supplementary Information

Reporting Summary

## Data Availability

Sequence data that support the findings of this study have been deposited in ENA (NA12878) with accession PRJEB30620 and in EGA (clinical sample) with the accession EGAS00001003469. All other relevant data are available upon request.

## Code availability

The code developed for phasing of long reads is available in the SEW software available at https://github.com/Genomicsplc/SEW

The code used to analyse the data in the current study are available on request from the authors.
