# Supplementary material for: Sequencing of human genomes with nanopore technology
Source: Nat Commun. 2019 Apr 23;10:1869. doi: 10.1038/s41467-019-09637-5 (PMC6478738; doi:10.1038/s41467-019-09637-5)
Supplement: Supplementary file 1 — Supplementary Information [file 41467_2019_9637_MOESM1_ESM.pdf]

Supplementary Information for "Sequencing of human genomes with nanopore technology"

Bowden *et al.*

March 18, 2019

Contents

1 Supplementary Note **2**
1.1 Patient Medical History and Genetic Findings 2
1.2 Phasing 2
1.2.1 Model formulation and complete data probability 2
1.2.2 Updating 4
1.2.3 Initialization and iteration 5
1.2.4 Heuristics 6
1.3 Base-Caller comparison 6
1.3.1 Base-calling performance 6
1.3.2 Variant calling performance 7

2 Supplementary Tables **7**

3 Supplementary Figures **21**
3.1 Supplementary Figures - NA12878 21
3.2 Supplementary Figures - LVC Snapshots 41
3.2.1 Large variant calls in ONT data not present in reference 41
3.2.2 Large variant calls in ONT data not present in reference - duplications 73
3.2.3 Large variant calls in reference data not called, but with ONT data support . 80
3.2.4 Large variant calls in reference data not present in ONT data 92
3.3 Supplementary Figures - Clinical sample 96

4 References **104**

1 Supplementary Note

1.1 Patient Medical History and Genetic Findings

The female patient, born following IVF to unrelated Caucasian parents, presented at the age of 9 months with recurrent respiratory tract infections, panhypogammaglobulinaemia, thrombocytopaenia and mild anaemia. Replacement immunoglobulin therapy from 17 months of age improved infection frequency and platelet count, and in spite of normal IgA and IgM levels, resulting severe thrombocytopaenia and the onset of other immune conditions have since thwarted the withdrawal of immunoglobulin replacement therapy. Having developed severe enteropathy with granulomatous inflammation, at the age of 12 years she required a pancolectomy due to incontrollable haemorrhage during colonoscopy. Despite this, and treatment with immunomodulating agents including anti-TNF, she remains symptomatic with chronic diarrhoea and fistulation. She developed psoriasis at the age of 20 and a seronegative large-joint oligoarthritis at 24 years requiring management with methrotrexate. After a childhood history of dyspraxia the patient developed progressive neurological symptoms from the age of 20-25 years, including initially poor balance and coordination and difficulty fixating on objects and progressing to a dependence on mobility aids since the age of 28, with additional severe dysarthria and some extra-cerebellar symptoms including difficulty in swallowing and word-finding. Neurological investigations have revealed cerebellar ataxia, gaze-evoked nystagmus in all directions, hyperreflexia, cerebellar atrophy and extensive supratentorial grey and white matter signal changes on MRI and a raised cerebrospinal fluid protein level with no evidence of infection. Tests for known genetic causes of antibody deficiency or cerebellar atrophy were negative.

1.2 Phasing

1.2.1 Model formulation and complete data probability

The math in this section can be seen as an extension of the STITCH approach of Davies *et al.* [1]. Consider a diploid individual with one maternal and one paternal haplotype that intersect a set of T SNPs (indexed $t \in \{1, ..., T\}$) at physical positions L along some chromosome (*i.e.* $L_t \in \mathbb{N}$). Consider a collection of sequencing reads (indexed $r \in \mathbb{N}$) that come from those two haplotypes. Let read r come from haplotype $H_r \in \{1, 2\}$ for arbitrarily labelled maternal (1) and paternal (2) haplotypes. We assume that, without additional information, each read is equally likely to come from the two haplotypes regardless of any parameters θ, or

$$P(H_r = k) = P(H_r = k|\theta) = \frac{1}{2} \tag{1}$$

At a particular SNP, without loss of generality, let 0 be the reference base, 1 the alternate base, and let 2 and 3 the other two bases. Define each observed read $R_r = \{(u_{r,j}, s_{r,j}, b_{r,j})|j = 1, ..., J_r\}$, where each read intersects J_r SNPs, where $u_{r,j}$ is the index of SNP j from read r (*i.e.* $u_{r,j} \in \{1, ..., T\}$), $s_{r,j}$ is the observed sequenced base for SNP j in read r ($s_{r,j} \in \{0, 1, 2, 3\}$), and $b_{r,j}$ is the phred scaled base quality ($b_{r,j} \in \mathbb{N}$). Let $g_{r,j}$ ($g_{r,j} \in \{0, 1, 2, 3\}$) be the real (unobserved) base for read r and SNP j. Define the probability of the underlying sequenced read having the alternate or reference base as being

$$P(G_{r,j} = 1|H_r = k, \theta) = \theta_{u_{r,j},k}$$
$$P(G_{r,j} = 0|H_r = k, \theta) = (1 - \theta_{u_{r,j},k})$$

or, more generally for convenience

$$P(G_{r,j} = g, |H_r = k, \theta) = \theta^g_{u_{r,j},k} \tag{2}$$

with $0 \leq \theta_{t,k} \leq 1$, and where superscript g relates to notation and not to exponentiation. Note that we define $P(G_{r,j} > 1, |H_r = k, \theta) = 0$ under the model.

Fundementally, at each SNP it intersects, each read has a real underlying genotype $g_{r,j}$, and we observe a sequenced base $s_{r,j}$, such that

$$P(S_{r,j} = v|G_{r,j} = g) = \begin{cases} 1 - 10^{\frac{-b_{r,j}}{10}} & v = g \\ \frac{1}{3}10^{\frac{-b_{r,j}}{10}} & v \neq g \end{cases} \tag{3}$$

and again for convenience define

$$P(S_{r,j} = s_{r,j}|G_{r,j} = w) = \phi^w_{r,j} \tag{4}$$

where again superscript w relates to notation and not exponentiation.

We can also calculate the probability of observing base v in read r at SNP $u_{r,j}$ given it came from haplotype k as

$$\begin{aligned} P(S_{r,j} = v|H_r = k, \theta) &= \sum_{g=0}^{1} P(S_{r,j} = v|H_r = k, G_{r,j} = g, \theta)P(G_{r,j} = g|H_r = k, \theta) \\ &= \sum_{g=0}^{1} P(S_{r,j} = v|G_{r,j} = g)P(G_{r,j} = g|H_r = k, \theta) \qquad (5) \\ &= \sum_{g=0}^{1} \phi^g_{r,j}\theta^g_{u_{r,j},k} \qquad (6) \\ &= \phi^1_{r,j}\theta_{u_{r,j},k} + \phi^0_{r,j}(1 - \theta_{u_{r,j},k}) \qquad (7) \end{aligned}$$

We then define the probability of observing a read as the product of the probabilities of observing each sequenced base, or

$$P(R_r|\theta) = \prod_{j=1}^{J_r} P(S_{r,j}|\theta) \tag{8}$$

Finally, we can calculate the joint probability of the observations and hidden parameters as

$$
\begin{aligned}
P(O=\{R_r\},H=h,G=g|\theta) &= \prod_{r=1}^{|O|} P(R_r, H_r=h_r, G_r=g_r|\theta) \\
&= \prod_{r=1}^{|O|}\prod_{j=1}^{J_r} P(S_{r,j}=s_{r,j}, H_r=h_r, G_{r,j}=g_{r,j}|\theta) \\
&= \prod_{r=1}^{|O|}\prod_{j=1}^{J_r} P(S_{r,j}=s_{r,j}|H_r=h_r, G_{r,j}=g_{r,j},\theta)P(H_r=h_r, G_{r,j}=g_{r,j}|\theta) \\
&= \prod_{r=1}^{|O|}\prod_{j=1}^{J_r} P(S_{r,j}=s_{r,j}|G_{r,j}=g_{r,j})P(G_{r,j}=g_{r,j}|H_r=h_r,\theta)P(H_r=h_r|\theta) \\
&= \prod_{r=1}^{|O|}\prod_{j=1}^{J_r} \frac{1}{2}\phi_{r,j}^{g_{r,j}}\theta_{u_{r,j},h_r}^{g_{r,j}} \qquad (9)
\end{aligned}
$$

1.2.2 Updating

Let $Q=\{H,G\}$ and a particular set of hidden values $q=\{h,g\}$ have domain $\mathcal{Q}$. Then in EM we seek to choose θ^{i+1} to maximize

$$
\begin{aligned}
U(\theta^{i+1},\theta^i) &= \mathbb{E}[l(\theta^{i+1})|O,\theta^i] \\
&= \sum_{q\in\mathcal{Q}} P(Q=q|O,\theta^i)\log(P(O,Q|\theta^{i+1})) \qquad (10)
\end{aligned}
$$

Now, note that we can calculate the log-likelihood of the complete data probability as follows

$$
\begin{aligned}
l(\theta) &= \log(P(O=o,H=h,G=g|\theta)) \\
&= \sum_{r,j:u_{r,j}=t,g_{r,j}=1,h_r=1} \log(\frac{1}{2}\phi_{r,j}^1\theta_{t,1}) \\
&+ \sum_{r,j:u_{r,j}=t,g_{r,j}=0,h_r=1} \log(\frac{1}{2}\phi_{r,j}^0(1-\theta_{t,1})) \\
&+ \sum_{r,j:u_{r,j}=t,g_{r,j}=1,h_r=2} \log(\frac{1}{2}\phi_{r,j}^1\theta_{t,2}) \\
&+ \sum_{r,j:u_{r,j}=t,g_{r,j}=0,h_r=2} \log(\frac{1}{2}\phi_{r,j}^0(1-\theta_{t,2})) \qquad (11)
\end{aligned}
$$

Therefore, we can get updates by calculating minima of the likelihood to yield the following updates of $\theta_{t,k}$ using

$$
\begin{aligned}
0 = \frac{d[U(\theta^{i+1}_{t,k}, \theta^{i}_{t,k})]}{d\theta^{i+1}_{t,k}} &= \frac{d}{d\theta^{i+1}_{t,k}}[\sum_{q\in Q} P(Q=q|O,\theta^i)\log(P(O,Q=q|\theta^{i+1}))] \\
&= \frac{d}{d\theta^{i+1}_{t,k}}[\sum_{g=0}^{1}\sum_{r,j:u_{r,j}=t} P(H_r=k, G_{r,j}=g|O,\theta^i)\log(P(O,H_r=k,G_{r,j}=g|\theta^{i+1})] \\
&= \sum_{r,j:u_{r,j}=t} P(H_r=k,G_{r,j}=1|O,\theta^i)\Big[\frac{1}{\theta^{i+1}_{t,1}}\Big] \\
&+ \sum_{r,j:u_{r,j}=t} P(H_r=k,G_{r,j}=0|O,\theta^i)\Big[\frac{1}{1-\theta^{i+1}_{t,1}}\Big] \\
\implies \theta^{i+1}_{t,k} &= \frac{\sum_{r,j:u_{r,j}=t} P(H_r=k,G_{r,j}=1|O,\theta^i)}{\sum_{g=0}^{1}\sum_{r,j:u_{r,j}=t} P(H_r=k,G_{r,j}=g|O,\theta^i)} \qquad (12)
\end{aligned}
$$

We can calculate $P(H_r = k, G_{r,j} = g|O, \theta^i)$ from Equation 12 as follows. First, using Equation 8, we can define

$$
\begin{aligned}
P(R_{r,-j}|H_r=k,\theta) &= \Big[\prod_{i=1,i\neq j}^{J_r} P(S_{r,i}=s_{r,i}|H_r=k,\theta)\Big] \\
&= \frac{P(R_r|H_r=k,\theta)}{P(S_{r,j}=s_{r,j}|H_r=k,\theta)} \qquad (13)
\end{aligned}
$$

then we can calculate

$$
\begin{aligned}
P(H_r=k,G_{r,j}=v|O,\theta) &= \frac{P(O,H_r=k,G_{r,j}=v|\theta)}{\sum_{m=1}^{2}\sum_{w=0}^{1} P(O,H_r=m,G_{r,j}=w|\theta)} \qquad (14) \\
&= \frac{P(R_r,H_r=k,G_{r,j}=v|\theta)}{P(R_r|\theta)} \qquad (15) \\
&= \frac{P(R_r|H_r=k,G_{r,j}=v,\theta)P(G_{r,j}=v|H_r=k,\theta)P(H_r=k|\theta)}{P(R_r|\theta)} \\
&= \frac{[P(R_{r,-j}|H_r=k,\theta)P(S_{r,j}=v|G_{r,j}=v)]P(G_{r,j}=v|H_r=k,\theta)P(H_r=k|\theta)}{P(R_r|\theta)} \\
&= \frac{P(R_{r,-j}|H_r=k,\theta)\frac{1}{2}\phi^{v}_{r,j}\theta^{v}_{r,j}}{P(R_r|\theta)} \qquad (16)
\end{aligned}
$$

where Equation 14 holds due to $P(A|B,C) = \frac{P(A,B|C)}{P(B|C)}$, Equation 15 holds as the probabilities of the reads given parameters are independent, and the terms in the denominators are given in Equations 2, 4, 8, 13.

Therefore, we can use the aforementionned probabilities to find values of θ that locally optimize the likelihood. We use heuristics (described below) in an attempt to find better local minima.

1.2.3 Initialization and iteration

We initialized $\theta_{t,k} \forall t, k$ by performing random draws from the uniform $U(0, 1)$ distribution. We then performed 300 EM iterations, whereby we first calculated $P(H_r = k, G_{r,j} = v|O, \theta)$ given θ, and

then calculated new θ using Equation 12. We also applied the heuristic (described below) at every 20th iteration between iterations 20 and 200.

1.2.4 Heuristics

Expectation maximization is a technique that is guaranteed to find parameters that locally maximize the likelihood function of a statical model. However, if the likelihood function is not concave, there is no guarantee that a global maxima will be obtained. Here, we describe a heuristic approach that attempts to enable discovery of parameter values that enable higher local maxima to be found.

Recall that the model we've described attempts to model an individuals diploid chromosomes using parameters such that haplotype k emits a read containing a reference base at SNP t with probability $\theta_{t,k}$. Ideally, a one-to-one correspondence can be found in which the maternal haplotype refers along it's entire length of the computational haplotype $k = 1$ or $k = 2$. However, since we initialize with random data, then it is likely that local maxima will be found where locally, the maternal haplotype looks like either haplotype $k = 1$ or $k = 2$, and then switches to the other haplotype at some further SNP. We implemented a method that attemps to find such switches and to resolve them.

We therefore scan between all pairs of SNPs and see whether a likelihood calculated using a subset of the data is improved if an artificial phase switch is introduced. More formally, between SNPs t and $t+1$, we get the subset of reads $O_{t,t+1} = \{R_r | \exists j_1, j_2 \text{s.t.} u_{r,j_1} = t, u_{r,j_2} = t+1\}$. We then introduce a phase switch between SNPs t and $t+1$ in a temporary set of parameters, *i.e.* $\theta'_{t,1} = \theta_{t,1}$, $\theta'_{t,2} = \theta_{t,2}$, $\theta'_{t+1,1} = \theta_{t,2}$, $\theta'_{t+1,2} = \theta_{t,1}$. We then perform two iterations using the new parameters and the subset of the dataset to iterate updating with this artificial set of parameters, and calculate a likelihood $l(\theta' | O_{t,t+1})$.

Once this is done for all pairs of SNPs, we begin a resursive process to select a set of phase switches to apply to the dataset. We consider only phase switches that offer an increase in the log-likelihood in the restricted observation set. We then recursively select a phase switch that offers the greatest increase in log likelihood relative to the original parameters among the set of eligible phase switches, and then remove from consideration phase switches from all pairs of SNPs that intersect the phase set of the current pairs of SNPs, where we consider a phase set to be a partionning of the available SNPs $t = 1, ..., T$ into a minimal number of member sets such that every SNP in every member of a phase set can be connected by tiling overlapping reads. The recursion ends when there are no more eligible phase switches to consider.

1.3 Base-Caller comparison

1.3.1 Base-calling performance

To evaluate the impact of different basecalling algorithms on read-level base accuracy and consequent variant calling accuracy, we performed a series of benchmarks.

The benchmarks are based on a data set built in the early phase of the project which used Nanonet v2.0.0 for base-calling. From this data set, we selected reads mapping to chromosome 22 and extracted the corresponding MinION reads from the original Fast5 data set. The Fast5 was then re-called using three alternatives to Nanonet v2.0.0: Albacore v2.0.2, Metrichor v2.43.1 and Scrappie v0.2.2.

Albacore v2.0.2 was run using the R9.4 450bps linear configt; Scrappie v0.2.2 was run in default configuration; Metrichor was run using the 1D workflow. After basecalling, the reads in each dataset

were re-mapped against the human genome reference (see Methods in the main manuscript).

We noted substantial variation in the resulting read-level substitution, deletion and insertion error rates (Figures 3, 5, 4).

Albacore v2.0.2 achieved the lowest unfiltered substitution error rate (mean 14.3%) and deletion error rate (mean 5.22%). The latter is offset by an elevated insertion error rate (mean 3.3%)

1.3.2 Variant calling performance

We tested the variant calling performance characterstics on each of the four base-called data sets described above. In addition, we employed various post-alignment filters to check the effect of removing low-quality reads before variant calling. The filters were "unfiltered", "fixed_error" (removing all reads with an error rate of 20%) and "fixed_size" (removing 20% of reads with the highest error rate) (11). Variant calling and evaluation were performed as described in the Methods section of the main manuscript.

In terms of variant calling performance, the - now discontinued - base caller Metrichor performed best, but with Albacore showing almost comparable performance. Scrappie and Nanonet produced worse results (12). At optimum performance, Metrichor and Albacore had similar false negative rates, but Metrichor overall had lower false positive rates (12). Filtering the read set by quality had little effect on the overall result.

2 Supplementary Tables

Supplementary Table 1: Flow cell statistics Number of reads and bases per flow cell. Numbers are listed separated for reads in the "fail" and "pass" fraction. Additionally, the fraction "trimmed" contains read numbers and lengths after trimming. The trimming tool splits read with internal adapter sequences into multiple "fragments", which are listed separately.

flowcell	nreads_fail	nbases_fail	nreads_pass	nbases_pass	nreads_ trimmed	nbases_ trimmed	nfragments_ trimmed	duration
WTON000137	41,512	105,096,132	431,593	3,078,102,597	431,372	3,029,057,523	433,457	48
WTON000138	30,779	73,738,511	382,754	2,839,232,425	382,569	2,795,253,786	386,098	48
WTON000139	54,424	104,129,343	197,345	1,191,264,106	192,119	1,144,757,110	192,272	48
WTON000140	37,900	93,709,814	554,920	4,101,524,131	554,706	4,037,697,765	557,933	48
WTON000148	69,144	154,770,217	829,788	5,849,143,557	829,475	5,756,980,231	835,912	48
WTON000149	63,218	165,653,291	854,520	5,990,855,945	854,210	5,894,388,345	860,562	48
WTON000150	48,932	108,372,646	708,691	5,022,884,658	708,469	4,943,921,759	714,030	48
WTON000151	39,092	97,054,068	595,340	4,265,899,224	595,140	4,198,997,150	599,913	48
WTON000152	54,077	133,031,371	705,653	5,028,358,967	705,453	4,949,372,623	710,929	48
WTON000153	53,632	123,291,153	735,115	5,224,241,751	734,840	5,142,452,765	740,549	48
WTON000154	47,312	108,184,066	760,533	5,392,469,830	760,268	5,309,107,116	765,855	48
WTON000155	59,070	152,700,575	819,564	5,777,841,881	819,258	5,689,272,580	824,917	48
WTON000176	54,918	156,453,219	631,667	4,380,070,529	631,511	4,313,351,905	636,601	48
WTON000177	52,615	110,746,849	827,487	5,676,129,280	827,265	5,588,628,691	833,755	48
WTON000178	29,094	69,523,464	510,789	3,482,206,300	510,660	3,427,862,104	514,699	48
WTON000179	64,924	149,726,643	797,521	5,504,742,269	797,318	5,421,606,996	803,478	48
WTON000180	51,212	103,509,976	699,531	4,850,850,323	699,372	4,777,863,041	704,838	48
WTON000181	71,607	170,705,359	869,142	5,902,696,089	868,940	5,813,408,076	875,225	48
WTON000182	49,564	114,892,874	793,797	5,434,184,217	793,629	5,351,290,404	799,698	48
WTON000183	81,647	236,006,929	946,106	6,624,540,869	945,825	6,523,082,492	954,466	48
WTON000184	19,454	32,406,910	334,054	2,124,523,616	333,928	2,085,993,606	336,224	24
WTON000185	18,810	37,687,008	315,628	1,971,140,987	315,489	1,936,345,344	317,440	24
WTON000186	33,567	57,129,018	518,624	3,275,959,818	518,379	3,217,927,428	521,805	24
WTON000187	6,386	9,810,451	139,819	862,368,010	139,748	847,210,757	140,564	24
WTON000188	19,418	34,720,980	350,425	2,178,924,310	350,266	2,139,650,750	352,378	24
WTON000189	16,619	28,815,260	263,876	1,630,892,224	263,792	1,601,349,545	265,483	24
WTON000190	23,151	48,048,993	438,470	2,756,504,685	438,322	2,706,237,633	441,121	24
WTON000191	31,584	62,722,137	587,364	3,780,486,126	587,148	3,711,729,553	591,169	24
WTON000192	51,730	108,759,407	815,856	5,052,686,338	815,605	4,962,602,736	822,590	24
WTON000193	11,525	20,955,098	190,104	1,121,520,892	190,003	1,100,494,898	191,438	24
WTON000194	40,129	66,915,296	586,063	3,559,644,976	585,848	3,492,232,023	591,138	24
WTON000195	13,541	21,823,572	227,433	1,352,885,513	227,333	1,327,189,793	229,171	24
WTON000196	17,473	26,984,832	311,603	1,846,286,262	311,473	1,810,925,980	313,941	24
WTON000197	12,583	21,058,903	259,306	1,553,784,752	259,208	1,524,397,629	261,375	24
WTON000198	15,011	24,326,228	279,985	1,680,083,842	279,895	1,648,317,618	282,218	24
WTON000199	15,981	25,880,843	304,974	1,829,020,422	304,863	1,793,936,494	307,466	24
WTON000200	65,659	133,127,318	999,466	5,948,356,415	999,175	5,831,517,882	1,008,050	24
WTON000201	12,611	23,280,328	204,300	1,189,885,461	204,209	1,166,775,612	205,797	24
WTON000202	12,753	20,247,856	296,652	1,714,812,395	296,483	1,680,565,491	298,931	24
WTON000203	9,998	15,576,774	195,529	1,117,477,874	195,429	1,095,294,766	196,880	24
WTON000204	13,008	18,160,021	255,137	1,456,910,268	254,998	1,427,499,519	256,885	24
WTON000205	7,437	10,647,602	139,277	790,212,374	139,194	774,565,197	140,191	24
WTON000206	12,461	25,649,836	268,968	1,537,659,678	268,847	1,506,469,668	270,961	24
WTON000207	13,232	21,676,025	250,682	1,426,220,968	250,535	1,397,341,435	252,387	24
WTON000216	49,400	105,184,882	746,909	4,525,243,943	746,725	4,442,712,268	755,612	24
WTON000217	48,463	101,167,816	672,331	4,090,746,708	672,092	4,016,385,798	679,785	24
WTON000218	35,762	64,630,156	573,037	3,432,898,000	572,830	3,366,789,577	579,985	24
WTON000219	50,005	92,612,258	593,151	3,588,169,793	592,993	3,521,550,307	600,324	24
WTON000220	24,490	38,381,663	341,363	2,034,670,152	341,261	1,995,986,843	345,358	24
WTON000221	11,778	21,261,676	171,568	1,024,755,741	171,517	1,005,061,227	173,662	24
WTON000222	38,766	79,407,678	533,918	3,256,182,193	533,736	3,192,384,518	540,353	24
WTON000223	41,126	75,107,096	634,505	3,818,425,753	634,327	3,747,792,936	641,920	24
WTON000224	50,423	108,962,072	688,819	4,654,681,875	688,590	4,577,030,653	699,006	24
WTON000225	49,500	110,287,423	751,163	5,053,936,760	750,942	4,967,851,057	762,767	24
WTON000226	39,697	90,038,614	703,063	4,752,232,906	702,821	4,672,560,349	713,754	24
WTON000227	83,464	207,817,281	952,053	6,425,190,567	951,785	6,317,226,335	966,729	48
WTON000228	51,064	110,314,036	674,924	4,480,373,869	674,688	4,408,038,531	684,260	24
WTON000229	82,856	193,518,884	856,356	5,705,732,741	856,082	5,611,259,712	868,224	48
WTON000230	111,579	319,607,435	1,236,182	8,499,941,117	1,235,790	8,357,460,428	1,256,306	48
WTON000231	49,321	101,444,036	799,358	5,334,201,627	799,097	5,241,445,266	811,381	24
WTON000232	64,104	182,378,749	690,814	4,604,690,479	690,606	4,525,987,255	698,406	48
WTON000233	98,765	258,618,668	1,041,072	6,968,287,531	1,040,785	6,851,052,701	1,052,483	48
WTON000234	32,007	70,231,177	475,984	3,168,170,095	475,836	3,112,703,520	481,072	48
WTON000235	78,069	207,287,249	940,985	6,176,785,048	940,732	6,071,690,793	950,771	48
WTON000236	81,649	216,493,473	867,351	5,752,004,768	867,115	5,652,602,766	876,243	48
WTON000237	35,451	79,143,307	433,687	2,673,105,951	433,474	2,628,073,640	437,379	48
WTON000238	48,273	104,551,737	616,795	3,852,844,982	616,545	3,783,228,336	624,023	48
WTON000239	89,748	189,457,132	858,978	5,376,761,408	858,656	5,281,633,300	868,983	48
WTON000240	51,327	115,053,002	625,253	3,923,830,841	625,015	3,856,178,474	632,204	48
WTON000241	60,185	144,248,206	732,396	4,515,510,024	732,148	4,435,703,508	740,568	48
WTON000242	40,788	84,361,670	408,424	2,493,330,341	408,291	2,448,422,971	412,842	48
WTON000243	97,289	252,913,251	931,551	5,958,241,328	931,192	5,852,319,809	942,854	48
WTON000244	80,213	174,188,335	778,326	4,822,251,986	778,036	4,737,137,085	786,738	48

Supplementary Table 2: Base-caller comparison results Error rates in mapped reads base-called by different base callers. Shown are the substitution, insertion and deletion error rates, the total number of reads in a set, the number of reads mapped and the average read length. The "basecaller" column list the base-caller and the "process" column indicates how the data set was filtered: "fixed_error" remove alignments with an error rate above 20%, "fixed_size": remove approx. 20% of alignments with highest error rate, "unfiltered": all alignments.

**basecaller**	**process**	**substitution rate percent**	**insertion rate percent**	**deletion rate percent**	**reads total**	**reads mapped**	**average length**
albacore2.0.2-chr22	fixed error	10.86	2.72	4.18	407,274	402,612	6,587
albacore2.0.2-chr22	fixed size	11.63	2.88	4.41	454,583	449,921	6,582
albacore2.0.2-chr22	unfiltered	14.31	3.30	5.22	557,633	552,971	6,454
metrichor-chr22	fixed error	12.62	1.09	7.43	386,870	384,045	6,339
metrichor-chr22	fixed size	13.67	1.20	7.89	448,073	445,248	6,323
metrichor-chr22	unfiltered	16.43	1.54	8.74	558,072	555,207	6,309
nanonet-chr22	fixed error	12.86	1.02	7.69	357,433	357,433	5,915
nanonet-chr22	fixed size	13.72	1.10	8.09	408,177	408,177	5,881
nanonet-chr22	unfiltered	15.83	1.34	8.84	558,269	558,269	5,628
scrappie-chr22	fixed error	11.95	2.80	4.78	382,627	379,770	6,539
scrappie-chr22	fixed size	12.68	2.89	5.09	429,846	426,970	6,551
scrappie-chr22	unfiltered	15.98	3.18	6.37	550,398	547,281	6,731

Supplementary Table 3: Variant-call filtering / Part 1 of 2 Variant calling results for Albacore 2.0.2 using different QUAL filtering options with phasing information. For example, “QUAL > 10 & GT != 0|0 & (0 < SB1&2 < 1) & ((PE1 + 0.5 * QD) > 2.5)” indicates first removing keeping and phasing only sites with QUAL > 10, then after phasing, keep sites where the genotype was not 0|0, strand bias was neither 0 nor 1, and using the phase entropy metric and QD (quality by depth) metrics, (PE1 + 0.5 * QD) > 2.5.

Basecaller	Filtration Approach	F1 score	FDR	FNR	Condition
Albacore	Heuristic with phasing	0.816	0.275	0.066	QUAL > 10
Albacore	Heuristic with phasing	0.86	0.202	0.068	QUAL > 10 & ((PE1 + 0.5 * QD) > 2)
Albacore	Heuristic with phasing	0.867	0.187	0.071	QUAL > 10 & ((PE1 + 0.5 * QD) > 2.25)
Albacore	Heuristic with phasing	0.873	0.174	0.075	QUAL > 10 & ((PE1 + 0.5 * QD) > 2.5)
Albacore	Heuristic with phasing	0.855	0.2	0.081	QUAL > 10 & (max(SRP, SAP) < 30)
Albacore	Heuristic with phasing	0.87	0.173	0.083	QUAL > 10 & (max(SRP, SAP) < 30) & ((PE1 + 0.5 * QD) > 2)
Albacore	Heuristic with phasing	0.874	0.163	0.086	QUAL > 10 & (max(SRP, SAP) < 30) & ((PE1 + 0.5 * QD) > 2.25)
Albacore	Heuristic with phasing	0.877	0.154	0.089	QUAL > 10 & (max(SRP, SAP) < 30) & ((PE1 + 0.5 * QD) > 2.5)
Albacore	Heuristic with phasing	0.816	0.27	0.076	QUAL > 10 & (0 < SB1&2 < 1)
Albacore	Heuristic with phasing	0.86	0.194	0.079	QUAL > 10 & (0 < SB1&2 < 1) & ((PE1 + 0.5 * QD) > 2)
Albacore	Heuristic with phasing	0.867	0.179	0.081	QUAL > 10 & (0 < SB1&2 < 1) & ((PE1 + 0.5 * QD) > 2.25)
Albacore	Heuristic with phasing	0.873	0.166	0.085	QUAL > 10 & (0 < SB1&2 < 1) & ((PE1 + 0.5 * QD) > 2.5)
Albacore	Heuristic with phasing	0.853	0.196	0.091	QUAL > 10 & (0 < SB1&2 < 1) & (max(SRP, SAP) < 30)
Albacore	Heuristic with phasing	0.867	0.169	0.093	QUAL > 10 & (0 < SB1&2 < 1) & (max(SRP, SAP) < 30) & ((PE1 + 0.5 * QD) > 2)
Albacore	Heuristic with phasing	0.871	0.159	0.096	QUAL > 10 & (0 < SB1&2 < 1) & (max(SRP, SAP) < 30) & ((PE1 + 0.5 * QD) > 2.25)
Albacore	Heuristic with phasing	0.875	0.149	0.099	QUAL > 10 & (0 < SB1&2 < 1) & (max(SRP, SAP) < 30) & ((PE1 + 0.5 * QD) > 2.5)
Albacore	Heuristic with phasing	0.904	0.116	0.076	QUAL > 10 & GT != 0|0
Albacore	Heuristic with phasing	0.913	0.097	0.077	QUAL > 10 & GT != 0|0 & ((PE1 + 0.5 * QD) > 2)
Albacore	Heuristic with phasing	0.915	0.091	0.079	QUAL > 10 & GT != 0|0 & ((PE1 + 0.5 * QD) > 2.25)
Albacore	Heuristic with phasing	0.916	0.086	0.081	QUAL > 10 & GT != 0|0 & ((PE1 + 0.5 * QD) > 2.5)
Albacore	Heuristic with phasing	0.917	0.075	0.09	QUAL > 10 & GT != 0|0 & (max(SRP, SAP) < 30)
Albacore	Heuristic with phasing	0.921	0.067	0.091	QUAL > 10 & GT != 0|0 & (max(SRP, SAP) < 30) & ((PE1 + 0.5 * QD) > 2)
Albacore	Heuristic with phasing	0.921	0.065	0.093	QUAL > 10 & GT != 0|0 & (max(SRP, SAP) < 30) & ((PE1 + 0.5 * QD) > 2.25)
Albacore	Heuristic with phasing	0.921	0.063	0.095	QUAL > 10 & GT != 0|0 & (max(SRP, SAP) < 30) & ((PE1 + 0.5 * QD) > 2.5)
Albacore	Heuristic with phasing	0.904	0.106	0.086	QUAL > 10 & GT != 0|0 & (0 < SB1&2 < 1)
Albacore	Heuristic with phasing	0.913	0.087	0.087	QUAL > 10 & GT != 0|0 & (0 < SB1&2 < 1) & ((PE1 + 0.5 * QD) > 2)
Albacore	Heuristic with phasing	0.915	0.081	0.089	QUAL > 10 & GT != 0|0 & (0 < SB1&2 < 1) & ((PE1 + 0.5 * QD) > 2.25)
Albacore	Heuristic with phasing	0.916	0.076	0.091	QUAL > 10 & GT != 0|0 & (0 < SB1&2 < 1) & ((PE1 + 0.5 * QD) > 2.5)
Albacore	Heuristic with phasing	0.915	0.069	0.1	QUAL > 10 & GT != 0|0 & (0 < SB1&2 < 1) & (max(SRP, SAP) < 30)
Albacore	Heuristic with phasing	0.919	0.061	0.101	QUAL > 10 & GT != 0|0 & (0 < SB1&2 < 1) & (max(SRP, SAP) < 30) & ((PE1 + 0.5 * QD) > 2)
Albacore	Heuristic with phasing	0.918	0.059	0.103	QUAL > 10 & GT != 0|0 & (0 < SB1&2 < 1) & (max(SRP, SAP) < 30) & ((PE1 + 0.5 * QD) > 2.25)
Albacore	Heuristic with phasing	0.918	0.057	0.105	QUAL > 10 & GT != 0|0 & (0 < SB1&2 < 1) & (max(SRP, SAP) < 30) & ((PE1 + 0.5 * QD) > 2.5)
Albacore	Heuristic with phasing	0.789	0.322	0.057	QUAL > 1
Albacore	Heuristic with phasing	0.852	0.22	0.061	QUAL > 1 & ((PE1 + 0.5 * QD) > 2)
Albacore	Heuristic with phasing	0.862	0.202	0.064	QUAL > 1 & ((PE1 + 0.5 * QD) > 2.25)
Albacore	Heuristic with phasing	0.868	0.188	0.068	QUAL > 1 & ((PE1 + 0.5 * QD) > 2.5)
Albacore	Heuristic with phasing	0.841	0.231	0.072	QUAL > 1 & (max(SRP, SAP) < 30)
Albacore	Heuristic with phasing	0.867	0.183	0.076	QUAL > 1 & (max(SRP, SAP) < 30) & ((PE1 + 0.5 * QD) > 2)
Albacore	Heuristic with phasing	0.872	0.172	0.079	QUAL > 1 & (max(SRP, SAP) < 30) & ((PE1 + 0.5 * QD) > 2.25)
Albacore	Heuristic with phasing	0.876	0.161	0.083	QUAL > 1 & (max(SRP, SAP) < 30) & ((PE1 + 0.5 * QD) > 2.5)
Albacore	Heuristic with phasing	0.788	0.315	0.072	QUAL > 1 & (0 < SB1&2 < 1)
Albacore	Heuristic with phasing	0.852	0.21	0.075	QUAL > 1 & (0 < SB1&2 < 1) & ((PE1 + 0.5 * QD) > 2)
Albacore	Heuristic with phasing	0.861	0.192	0.078	QUAL > 1 & (0 < SB1&2 < 1) & ((PE1 + 0.5 * QD) > 2.25)
Albacore	Heuristic with phasing	0.867	0.178	0.082	QUAL > 1 & (0 < SB1&2 < 1) & ((PE1 + 0.5 * QD) > 2.5)
Albacore	Heuristic with phasing	0.838	0.226	0.087	QUAL > 1 & (0 < SB1&2 < 1) & (max(SRP, SAP) < 30)
Albacore	Heuristic with phasing	0.864	0.178	0.09	QUAL > 1 & (0 < SB1&2 < 1) & (max(SRP, SAP) < 30) & ((PE1 + 0.5 * QD) > 2)
Albacore	Heuristic with phasing	0.868	0.167	0.093	QUAL > 1 & (0 < SB1&2 < 1) & (max(SRP, SAP) < 30) & ((PE1 + 0.5 * QD) > 2.25)
Albacore	Heuristic with phasing	0.873	0.156	0.097	QUAL > 1 & (0 < SB1&2 < 1) & (max(SRP, SAP) < 30) & ((PE1 + 0.5 * QD) > 2.5)
Albacore	Heuristic with phasing	0.896	0.136	0.069	QUAL > 1 & GT != 0|0
Albacore	Heuristic with phasing	0.91	0.11	0.07	QUAL > 1 & GT != 0|0 & ((PE1 + 0.5 * QD) > 2)
Albacore	Heuristic with phasing	0.912	0.103	0.073	QUAL > 1 & GT != 0|0 & ((PE1 + 0.5 * QD) > 2.25)
Albacore	Heuristic with phasing	0.914	0.097	0.075	QUAL > 1 & GT != 0|0 & ((PE1 + 0.5 * QD) > 2.5)
Albacore	Heuristic with phasing	0.916	0.083	0.084	QUAL > 1 & GT != 0|0 & (max(SRP, SAP) < 30)
Albacore	Heuristic with phasing	0.922	0.071	0.085	QUAL > 1 & GT != 0|0 & (max(SRP, SAP) < 30) & ((PE1 + 0.5 * QD) > 2)
Albacore	Heuristic with phasing	0.922	0.069	0.087	QUAL > 1 & GT != 0|0 & (max(SRP, SAP) < 30) & ((PE1 + 0.5 * QD) > 2.25)
Albacore	Heuristic with phasing	0.922	0.066	0.089	QUAL > 1 & GT != 0|0 & (max(SRP, SAP) < 30) & ((PE1 + 0.5 * QD) > 2.5)
Albacore	Heuristic with phasing	0.897	0.123	0.083	QUAL > 1 & GT != 0|0 & (0 < SB1&2 < 1)
Albacore	Heuristic with phasing	0.909	0.097	0.085	QUAL > 1 & GT != 0|0 & (0 < SB1&2 < 1) & ((PE1 + 0.5 * QD) > 2)
Albacore	Heuristic with phasing	0.911	0.09	0.087	QUAL > 1 & GT != 0|0 & (0 < SB1&2 < 1) & ((PE1 + 0.5 * QD) > 2.25)
Albacore	Heuristic with phasing	0.913	0.085	0.089	QUAL > 1 & GT != 0|0 & (0 < SB1&2 < 1) & ((PE1 + 0.5 * QD) > 2.5)
Albacore	Heuristic with phasing	0.913	0.076	0.097	QUAL > 1 & GT != 0|0 & (0 < SB1&2 < 1) & (max(SRP, SAP) < 30)
Albacore	Heuristic with phasing	0.918	0.064	0.099	QUAL > 1 & GT != 0|0 & (0 < SB1&2 < 1) & (max(SRP, SAP) < 30) & ((PE1 + 0.5 * QD) > 2)
Albacore	Heuristic with phasing	0.918	0.062	0.101	QUAL > 1 & GT != 0|0 & (0 < SB1&2 < 1) & (max(SRP, SAP) < 30) & ((PE1 + 0.5 * QD) > 2.25)
Albacore	Heuristic with phasing	0.918	0.06	0.103	QUAL > 1 & GT != 0|0 & (0 < SB1&2 < 1) & (max(SRP, SAP) < 30) & ((PE1 + 0.5 * QD) > 2.5)
Albacore	Heuristic with phasing	0.831	0.248	0.072	QUAL > 20
Albacore	Heuristic with phasing	0.864	0.19	0.074	QUAL > 20 & ((PE1 + 0.5 * QD) > 2)
Albacore	Heuristic with phasing	0.871	0.176	0.076	QUAL > 20 & ((PE1 + 0.5 * QD) > 2.25)
Albacore	Heuristic with phasing	0.876	0.165	0.079	QUAL > 20 & ((PE1 + 0.5 * QD) > 2.5)
Albacore	Heuristic with phasing	0.862	0.183	0.087	QUAL > 20 & (max(SRP, SAP) < 30)
Albacore	Heuristic with phasing	0.872	0.164	0.089	QUAL > 20 & (max(SRP, SAP) < 30) & ((PE1 + 0.5 * QD) > 2)
Albacore	Heuristic with phasing	0.875	0.156	0.091	QUAL > 20 & (max(SRP, SAP) < 30) & ((PE1 + 0.5 * QD) > 2.25)
Albacore	Heuristic with phasing	0.878	0.148	0.094	QUAL > 20 & (max(SRP, SAP) < 30) & ((PE1 + 0.5 * QD) > 2.5)

Supplementary Table 3: Variant-call filtering / Part 2 of 2

Basecaller	Filtration Approach	F1 score	FDR	FNR	Condition
Albacore	Heuristic with phasing	0.83	0.243	0.082	QUAL > 20 & (0 < SB1&2 < 1)
Albacore	Heuristic with phasing	0.863	0.184	0.084	QUAL > 20 & (0 < SB1&2 < 1) & ((PE1 + 0.5 * QD) > 2)
Albacore	Heuristic with phasing	0.87	0.17	0.087	QUAL > 20 & (0 < SB1&2 < 1) & ((PE1 + 0.5 * QD) > 2.25)
Albacore	Heuristic with phasing	0.874	0.159	0.09	QUAL > 20 & (0 < SB1&2 < 1) & ((PE1 + 0.5 * QD) > 2.5)
Albacore	Heuristic with phasing	0.86	0.18	0.097	QUAL > 20 & (0 < SB1&2 < 1) & (max(SRP, SAP) < 30)
Albacore	Heuristic with phasing	0.869	0.161	0.099	QUAL > 20 & (0 < SB1&2 < 1) & (max(SRP, SAP) < 30) & ((PE1 + 0.5 * QD) > 2)
Albacore	Heuristic with phasing	0.873	0.152	0.101	QUAL > 20 & (0 < SB1&2 < 1) & (max(SRP, SAP) < 30) & ((PE1 + 0.5 * QD) > 2.25)
Albacore	Heuristic with phasing	0.876	0.144	0.104	QUAL > 20 & (0 < SB1&2 < 1) & (max(SRP, SAP) < 30) & ((PE1 + 0.5 * QD) > 2.5)
Albacore	Heuristic with phasing	0.907	0.104	0.081	QUAL > 20 & GT != 0|0
Albacore	Heuristic with phasing	0.915	0.089	0.081	QUAL > 20 & GT != 0|0 & ((PE1 + 0.5 * QD) > 2)
Albacore	Heuristic with phasing	0.916	0.084	0.083	QUAL > 20 & GT != 0|0 & ((PE1 + 0.5 * QD) > 2.25)
Albacore	Heuristic with phasing	0.917	0.08	0.085	QUAL > 20 & GT != 0|0 & ((PE1 + 0.5 * QD) > 2.5)
Albacore	Heuristic with phasing	0.918	0.069	0.095	QUAL > 20 & GT != 0|0 & (max(SRP, SAP) < 30)
Albacore	Heuristic with phasing	0.92	0.064	0.096	QUAL > 20 & GT != 0|0 & (max(SRP, SAP) < 30) & ((PE1 + 0.5 * QD) > 2)
Albacore	Heuristic with phasing	0.92	0.062	0.097	QUAL > 20 & GT != 0|0 & (max(SRP, SAP) < 30) & ((PE1 + 0.5 * QD) > 2.25)
Albacore	Heuristic with phasing	0.92	0.06	0.099	QUAL > 20 & GT != 0|0 & (max(SRP, SAP) < 30) & ((PE1 + 0.5 * QD) > 2.5)
Albacore	Heuristic with phasing	0.906	0.096	0.091	QUAL > 20 & GT != 0|0 & (0 < SB1&2 < 1)
Albacore	Heuristic with phasing	0.913	0.081	0.092	QUAL > 20 & GT != 0|0 & (0 < SB1&2 < 1) & ((PE1 + 0.5 * QD) > 2)
Albacore	Heuristic with phasing	0.915	0.076	0.093	QUAL > 20 & GT != 0|0 & (0 < SB1&2 < 1) & ((PE1 + 0.5 * QD) > 2.25)
Albacore	Heuristic with phasing	0.916	0.072	0.096	QUAL > 20 & GT != 0|0 & (0 < SB1&2 < 1) & ((PE1 + 0.5 * QD) > 2.5)
Albacore	Heuristic with phasing	0.915	0.064	0.105	QUAL > 20 & GT != 0|0 & (0 < SB1&2 < 1) & (max(SRP, SAP) < 30)
Albacore	Heuristic with phasing	0.917	0.059	0.106	QUAL > 20 & GT != 0|0 & (0 < SB1&2 < 1) & (max(SRP, SAP) < 30) & ((PE1 + 0.5 * QD) > 2)
Albacore	Heuristic with phasing	0.917	0.057	0.107	QUAL > 20 & GT != 0|0 & (0 < SB1&2 < 1) & (max(SRP, SAP) < 30) & ((PE1 + 0.5 * QD) > 2.25)
Albacore	Heuristic with phasing	0.917	0.055	0.109	QUAL > 20 & GT != 0|0 & (0 < SB1&2 < 1) & (max(SRP, SAP) < 30) & ((PE1 + 0.5 * QD) > 2.5)
Albacore	Heuristic with phasing	0.842	0.225	0.078	QUAL > 30
Albacore	Heuristic with phasing	0.868	0.178	0.08	QUAL > 30 & ((PE1 + 0.5 * QD) > 2)
Albacore	Heuristic with phasing	0.874	0.166	0.082	QUAL > 30 & ((PE1 + 0.5 * QD) > 2.25)
Albacore	Heuristic with phasing	0.878	0.157	0.085	QUAL > 30 & ((PE1 + 0.5 * QD) > 2.5)
Albacore	Heuristic with phasing	0.867	0.169	0.093	QUAL > 30 & (max(SRP, SAP) < 30)
Albacore	Heuristic with phasing	0.874	0.156	0.094	QUAL > 30 & (max(SRP, SAP) < 30) & ((PE1 + 0.5 * QD) > 2)
Albacore	Heuristic with phasing	0.877	0.148	0.096	QUAL > 30 & (max(SRP, SAP) < 30) & ((PE1 + 0.5 * QD) > 2.25)
Albacore	Heuristic with phasing	0.879	0.142	0.099	QUAL > 30 & (max(SRP, SAP) < 30) & ((PE1 + 0.5 * QD) > 2.5)
Albacore	Heuristic with phasing	0.84	0.221	0.088	QUAL > 30 & (0 < SB1&2 < 1)
Albacore	Heuristic with phasing	0.867	0.173	0.089	QUAL > 30 & (0 < SB1&2 < 1) & ((PE1 + 0.5 * QD) > 2)
Albacore	Heuristic with phasing	0.873	0.16	0.092	QUAL > 30 & (0 < SB1&2 < 1) & ((PE1 + 0.5 * QD) > 2.25)
Albacore	Heuristic with phasing	0.876	0.151	0.095	QUAL > 30 & (0 < SB1&2 < 1) & ((PE1 + 0.5 * QD) > 2.5)
Albacore	Heuristic with phasing	0.865	0.166	0.102	QUAL > 30 & (0 < SB1&2 < 1) & (max(SRP, SAP) < 30)
Albacore	Heuristic with phasing	0.871	0.152	0.104	QUAL > 30 & (0 < SB1&2 < 1) & (max(SRP, SAP) < 30) & ((PE1 + 0.5 * QD) > 2)
Albacore	Heuristic with phasing	0.874	0.145	0.106	QUAL > 30 & (0 < SB1&2 < 1) & (max(SRP, SAP) < 30) & ((PE1 + 0.5 * QD) > 2.25)
Albacore	Heuristic with phasing	0.876	0.138	0.109	QUAL > 30 & (0 < SB1&2 < 1) & (max(SRP, SAP) < 30) & ((PE1 + 0.5 * QD) > 2.5)
Albacore	Heuristic with phasing	0.908	0.096	0.087	QUAL > 30 & GT != 0|0
Albacore	Heuristic with phasing	0.915	0.083	0.087	QUAL > 30 & GT != 0|0 & ((PE1 + 0.5 * QD) > 2)
Albacore	Heuristic with phasing	0.916	0.078	0.089	QUAL > 30 & GT != 0|0 & ((PE1 + 0.5 * QD) > 2.25)
Albacore	Heuristic with phasing	0.917	0.075	0.091	QUAL > 30 & GT != 0|0 & ((PE1 + 0.5 * QD) > 2.5)
Albacore	Heuristic with phasing	0.916	0.066	0.101	QUAL > 30 & GT != 0|0 & (max(SRP, SAP) < 30)
Albacore	Heuristic with phasing	0.919	0.061	0.101	QUAL > 30 & GT != 0|0 & (max(SRP, SAP) < 30) & ((PE1 + 0.5 * QD) > 2)
Albacore	Heuristic with phasing	0.918	0.06	0.103	QUAL > 30 & GT != 0|0 & (max(SRP, SAP) < 30) & ((PE1 + 0.5 * QD) > 2.25)
Albacore	Heuristic with phasing	0.918	0.058	0.105	QUAL > 30 & GT != 0|0 & (max(SRP, SAP) < 30) & ((PE1 + 0.5 * QD) > 2.5)
Albacore	Heuristic with phasing	0.907	0.089	0.096	QUAL > 30 & GT != 0|0 & (0 < SB1&2 < 1)
Albacore	Heuristic with phasing	0.913	0.076	0.097	QUAL > 30 & GT != 0|0 & (0 < SB1&2 < 1) & ((PE1 + 0.5 * QD) > 2)
Albacore	Heuristic with phasing	0.915	0.071	0.098	QUAL > 30 & GT != 0|0 & (0 < SB1&2 < 1) & ((PE1 + 0.5 * QD) > 2.25)
Albacore	Heuristic with phasing	0.916	0.067	0.1	QUAL > 30 & GT != 0|0 & (0 < SB1&2 < 1) & ((PE1 + 0.5 * QD) > 2.5)
Albacore	Heuristic with phasing	0.914	0.061	0.11	QUAL > 30 & GT != 0|0 & (0 < SB1&2 < 1) & (max(SRP, SAP) < 30)
Albacore	Heuristic with phasing	0.916	0.057	0.11	QUAL > 30 & GT != 0|0 & (0 < SB1&2 < 1) & (max(SRP, SAP) < 30) & ((PE1 + 0.5 * QD) > 2)
Albacore	Heuristic with phasing	0.916	0.055	0.112	QUAL > 30 & GT != 0|0 & (0 < SB1&2 < 1) & (max(SRP, SAP) < 30) & ((PE1 + 0.5 * QD) > 2.25)
Albacore	Heuristic with phasing	0.915	0.053	0.114	QUAL > 30 & GT != 0|0 & (0 < SB1&2 < 1) & (max(SRP, SAP) < 30) & ((PE1 + 0.5 * QD) > 2.5)

Supplementary Table 4: pLI analysis Proportion of LoF mutations in false and true positive call sets. The proportion is stratified by pLI range. False positives are enriched for LoF mutations in genes with high pLI scores.

	False positives			True positives		
**pLI range**	**not LoF**	**LoF**	**LoF / %**	**not LoF**	**LoF**	**LoF / %**
(0.0, 0.1]	22,874	46	0.200698	400,824	122	0.030428
(0.1, 0.2]	1,074	0	0	19,132	6	0.031351
(0.2, 0.3]	682	0	0	14,771	4	0.027073
(0.3, 0.4]	1,111	0	0	19,111	6	0.031386
(0.4, 0.5]	962	0	0	14,985	1	0.006673
(0.5, 0.6]	1,025	0	0	17,431	8	0.045874
(0.6, 0.7]	1,009	1	0.09901	16,571	3	0.018101
(0.7, 0.8]	1,166	1	0.08569	21,897	3	0.013699
(0.8, 0.9]	1,758	1	0.05685	28,095	3	0.010677
(0.9, 1.0]	13,558	20	0.147297	235,965	17	0.007204

Supplementary Table 5: Comparison of base callers without phasing / Part 1 of 3 Variant calling results using different base callers and input filtering methods. “filtering”: filtering method applied to BAM files (“unfiltered”, “fixed error”: remove all alignments above error rate of 20%, “fixed size”: remove approx. 20% of alignments with highest error rate,), “RH/RA”: contamination level thresholds for freebayes, “threshold”: QUAL threshold for optimal F1 performance, “f measure“: F1 measure, “unfiltered false negative rate”: number of reference variant sites missing irrespective of QUAL threshold and genotype.

basecaller	filtering	RH/RA	threshold	f measure	false negative rate	false discovery rate	false negative rate before optimization	false negative rate after optimization
albacore2.0.2	fixed error	06 01	73.59	0.8635	0.1502	0.1223	0.030761682	0.053785763
albacore2.0.2	fixed error	06 02	83.179	0.8588	0.1507	0.1314	0.030761682	0.047180494
albacore2.0.2	fixed error	06 03	103.173	0.8499	0.1658	0.1338	0.030761682	0.042160489
albacore2.0.2	fixed error	06 04	129.735	0.8309	0.1945	0.1421	0.030761682	0.038537027
albacore2.0.2	fixed error	06 05	145.366	0.7818	0.2325	0.2033	0.030761682	0.036574319
albacore2.0.2	fixed error	06 06	176.386	0.565	0.4444	0.4253	0.030761682	0.035592964
albacore2.0.2	fixed error	07 01	69.364	0.8646	0.1465	0.1241	0.030761682	0.052464709
albacore2.0.2	fixed error	07 02	86.04	0.8632	0.1539	0.1189	0.030761682	0.045783951
albacore2.0.2	fixed error	07 03	105.733	0.858	0.1643	0.1184	0.030761682	0.040763947
albacore2.0.2	fixed error	07 04	131.575	0.8483	0.1832	0.1176	0.030761682	0.038159583
albacore2.0.2	fixed error	07 05	152.461	0.83	0.1967	0.1415	0.030761682	0.036612063
albacore2.0.2	fixed error	07 06	193.014	0.7824	0.2545	0.1768	0.030761682	0.035592964
albacore2.0.2	fixed error	08 01	51.105	0.8557	0.1465	0.142	0.030761682	0.057597947
albacore2.0.2	fixed error	08 02	67.166	0.8573	0.1494	0.136	0.030761682	0.049407413
albacore2.0.2	fixed error	08 03	91.091	0.8573	0.1619	0.1225	0.030761682	0.042688911
albacore2.0.2	fixed error	08 04	108.074	0.8535	0.1633	0.129	0.030761682	0.038952216
albacore2.0.2	fixed error	08 05	142.235	0.8434	0.1885	0.1222	0.030761682	0.037064996
albacore2.0.2	fixed error	08 06	184.167	0.8228	0.2224	0.1264	0.030761682	0.035932664
albacore2.0.2	fixed size	06 01	79.646	0.8638	0.1443	0.1278	0.027062731	0.049822601
albacore2.0.2	fixed size	06 02	98.753	0.8585	0.1547	0.1278	0.027062731	0.043217332
albacore2.0.2	fixed size	06 03	116.321	0.848	0.166	0.1376	0.027062731	0.038008606
albacore2.0.2	fixed size	06 04	142.77	0.8285	0.192	0.1499	0.027062731	0.035064543
albacore2.0.2	fixed size	06 05	176.452	0.7767	0.2506	0.1939	0.027062731	0.03340379
albacore2.0.2	fixed size	06 06	194.993	0.5646	0.4417	0.4289	0.027062731	0.032573413
albacore2.0.2	fixed size	07 01	79.184	0.8648	0.1458	0.1243	0.027062731	0.048463803
albacore2.0.2	fixed size	07 02	97.317	0.8626	0.1524	0.1217	0.027062731	0.041896278
albacore2.0.2	fixed size	07 03	111.943	0.8579	0.1545	0.1294	0.027062731	0.037178229
albacore2.0.2	fixed size	07 04	144.396	0.8477	0.1788	0.1241	0.027062731	0.034762588
albacore2.0.2	fixed size	07 05	175.075	0.8286	0.2019	0.1385	0.027062731	0.033554767
albacore2.0.2	fixed size	07 06	214.35	0.7791	0.2561	0.1822	0.027062731	0.03272439
albacore2.0.2	fixed size	08 01	58.072	0.855	0.1446	0.1455	0.027062731	0.053521552
albacore2.0.2	fixed size	08 02	75.014	0.8573	0.1463	0.139	0.027062731	0.045293274
albacore2.0.2	fixed size	08 03	99.41	0.8567	0.1573	0.1289	0.027062731	0.039178682
albacore2.0.2	fixed size	08 04	125.706	0.8529	0.1689	0.124	0.027062731	0.035366498
albacore2.0.2	fixed size	08 05	156.627	0.8433	0.1843	0.1271	0.027062731	0.033932211
albacore2.0.2	fixed size	08 06	203.304	0.823	0.2194	0.1299	0.027062731	0.033026346
albacore2.0.2	unfiltered	06 01	84.359	0.8608	0.145	0.1332	0.0255907	0.049822601
albacore2.0.2	unfiltered	06 02	99.265	0.8548	0.1498	0.1406	0.0255907	0.043066355
albacore2.0.2	unfiltered	06 03	124.149	0.8439	0.1665	0.1455	0.0255907	0.037631162
albacore2.0.2	unfiltered	06 04	151.685	0.8222	0.1922	0.1628	0.0255907	0.034347399
albacore2.0.2	unfiltered	06 05	184.437	0.7635	0.2532	0.219	0.0255907	0.032535668
albacore2.0.2	unfiltered	06 06	212.968	0.5611	0.442	0.4357	0.0255907	0.031441081
albacore2.0.2	unfiltered	07 01	82.292	0.8609	0.1457	0.1324	0.0255907	0.048312826
albacore2.0.2	unfiltered	07 02	100.777	0.8596	0.1497	0.1309	0.0255907	0.041707556
albacore2.0.2	unfiltered	07 03	126.001	0.8542	0.1614	0.1297	0.0255907	0.036687552
albacore2.0.2	unfiltered	07 04	157.048	0.843	0.1808	0.1317	0.0255907	0.033894467
albacore2.0.2	unfiltered	07 05	188.188	0.8217	0.2033	0.1517	0.0255907	0.032422435
albacore2.0.2	unfiltered	07 06	235.204	0.7661	0.2657	0.1992	0.0255907	0.031592059
albacore2.0.2	unfiltered	08 01	69.668	0.8514	0.1565	0.1405	0.0255907	0.052917642
albacore2.0.2	unfiltered	08 02	86.329	0.8535	0.1558	0.137	0.0255907	0.04491583
albacore2.0.2	unfiltered	08 03	102.181	0.8538	0.1509	0.1413	0.0255907	0.038197328
albacore2.0.2	unfiltered	08 04	137.991	0.8492	0.1715	0.1289	0.0255907	0.034649355
albacore2.0.2	unfiltered	08 05	172.986	0.8388	0.1877	0.1329	0.0255907	0.033101834
albacore2.0.2	unfiltered	08 06	222.488	0.8154	0.2239	0.1411	0.0255907	0.031818525
metrichor	fixed error	06 01	1.004	0.7813	0.2647	0.1665	0.056390126	0.228353589
metrichor	fixed error	06 02	1.015	0.8144	0.2171	0.1513	0.056390126	0.190344984
metrichor	fixed error	06 03	1.121	0.8449	0.1686	0.141	0.056390126	0.147429607
metrichor	fixed error	06 04	5.901	0.8641	0.1529	0.1182	0.056390126	0.110024911
metrichor	fixed error	06 05	17.928	0.8613	0.1593	0.117	0.056390126	0.081905337
metrichor	fixed error	06 06	38.971	0.6046	0.4101	0.38	0.056390126	0.068053144
metrichor	fixed error	07 01	1.006	0.7453	0.3067	0.1942	0.056390126	0.24794293
metrichor	fixed error	07 02	1.011	0.7894	0.2468	0.1708	0.056390126	0.2030271
metrichor	fixed error	07 03	1.02	0.829	0.1872	0.1541	0.056390126	0.156375028
metrichor	fixed error	07 04	4.779	0.8589	0.156	0.1258	0.056390126	0.111572431
metrichor	fixed error	07 05	17.256	0.8744	0.1458	0.1044	0.056390126	0.081641126
metrichor	fixed error	07 06	46.286	0.8461	0.1877	0.1172	0.056390126	0.068468332
metrichor	fixed error	08 01	1.004	0.6423	0.4251	0.2725	0.056390126	0.312750057
metrichor	fixed error	08 02	1.001	0.7009	0.3551	0.2325	0.056390126	0.264814675
metrichor	fixed error	08 03	1.008	0.763	0.2739	0.196	0.056390126	0.206877029
metrichor	fixed error	08 04	1.017	0.8165	0.1928	0.1739	0.056390126	0.145617876
metrichor	fixed error	08 05	9.309	0.8503	0.1657	0.1331	0.056390126	0.098097682
metrichor	fixed error	08 06	40.093	0.8398	0.1975	0.1192	0.056390126	0.074356458
metrichor	fixed size	06 01	1	0.776	0.2615	0.1825	0.05178531	0.222805163
metrichor	fixed size	06 02	1.001	0.8122	0.2092	0.1653	0.05178531	0.181173096

Supplementary Table 5: Comparison of base callers without phasing / Part 2 of 3

basecaller	filtering	RH/RA	threshold	f measure	false negative rate	false discovery rate	false negative rate before optimization	false negative rate after optimization
metrichor	fixed size	06 03	4.227	0.8431	0.1782	0.1345	0.05178531	0.137276364
metrichor	fixed size	06 04	13.973	0.8644	0.162	0.1076	0.05178531	0.100060391
metrichor	fixed size	06 05	25.604	0.8602	0.1619	0.1166	0.05178531	0.074771646
metrichor	fixed size	06 06	46.836	0.6027	0.4073	0.3869	0.05178531	0.062278252
metrichor	fixed size	07 01	1.001	0.7379	0.3062	0.2121	0.05178531	0.242847437
metrichor	fixed size	07 02	1.044	0.7856	0.2416	0.1853	0.05178531	0.194912056
metrichor	fixed size	07 03	1.25	0.8254	0.182	0.1671	0.05178531	0.146674719
metrichor	fixed size	07 04	5.64	0.8589	0.1471	0.1351	0.05178531	0.101947611
metrichor	fixed size	07 05	21.419	0.8748	0.1401	0.1098	0.05178531	0.074394202
metrichor	fixed size	07 06	56.236	0.8439	0.1868	0.123	0.05178531	0.062806673
metrichor	fixed size	08 01	1	0.6316	0.4305	0.2912	0.05178531	0.312259379
metrichor	fixed size	08 02	1.003	0.6935	0.3561	0.2487	0.05178531	0.261342191
metrichor	fixed size	08 03	1.007	0.7567	0.2709	0.2135	0.05178531	0.198912961
metrichor	fixed size	08 04	2.011	0.8123	0.1947	0.1805	0.05178531	0.135011701
metrichor	fixed size	08 05	13.292	0.8494	0.1633	0.1375	0.05178531	0.089039028
metrichor	fixed size	08 06	53.897	0.8374	0.2097	0.1097	0.05178531	0.068128633
metrichor	unfiltered	06 01	1.264	0.7645	0.2743	0.1924	0.049784857	0.23084472
metrichor	unfiltered	06 02	2.328	0.8045	0.2253	0.1634	0.049784857	0.184758813
metrichor	unfiltered	06 03	5.663	0.8398	0.1834	0.1356	0.049784857	0.138937118
metrichor	unfiltered	06 04	15.194	0.8631	0.1614	0.1109	0.049784857	0.099305503
metrichor	unfiltered	06 05	25.849	0.8554	0.1591	0.1297	0.049784857	0.07239375
metrichor	unfiltered	06 06	55.17	0.5954	0.4142	0.3948	0.049784857	0.060466521
metrichor	unfiltered	07 01	1	0.7241	0.3184	0.2277	0.049784857	0.251000226
metrichor	unfiltered	07 02	1.002	0.7744	0.2496	0.1999	0.049784857	0.199781083
metrichor	unfiltered	07 03	2.71	0.8197	0.1948	0.1652	0.049784857	0.147316374
metrichor	unfiltered	07 04	10.508	0.8569	0.1581	0.1275	0.049784857	0.100173624
metrichor	unfiltered	07 05	23.93	0.871	0.1395	0.1183	0.049784857	0.071638862
metrichor	unfiltered	07 06	64.265	0.8349	0.1921	0.1362	0.049784857	0.060919453
metrichor	unfiltered	08 01	1.006	0.6155	0.4455	0.3083	0.049784857	0.323620442
metrichor	unfiltered	08 02	1.003	0.6796	0.3684	0.2645	0.049784857	0.269381747
metrichor	unfiltered	08 03	1.01	0.7463	0.2784	0.2273	0.049784857	0.202460935
metrichor	unfiltered	08 04	3.69	0.8041	0.2078	0.1836	0.049784857	0.134219068
metrichor	unfiltered	08 05	13.726	0.8436	0.1605	0.1522	0.049784857	0.086925342
metrichor	unfiltered	08 06	59.828	0.8288	0.2106	0.1276	0.049784857	0.065562014
nanonet	fixed error	06 01	45.666	0.7752	0.2557	0.1911	0.060013588	0.114290028
nanonet	fixed error	06 02	63.309	0.7686	0.275	0.1823	0.060013588	0.095795274
nanonet	fixed error	06 03	78.893	0.7535	0.2866	0.2015	0.060013588	0.080961727
nanonet	fixed error	06 04	100.897	0.7208	0.3218	0.2309	0.060013588	0.069940364
nanonet	fixed error	06 05	125.201	0.6526	0.3826	0.3079	0.060013588	0.065373292
nanonet	fixed error	06 06	154.795	0.4472	0.5676	0.537	0.060013588	0.064429682
nanonet	fixed error	07 01	42.815	0.7801	0.2575	0.1783	0.060013588	0.111534687
nanonet	fixed error	07 02	57.086	0.777	0.2644	0.1765	0.060013588	0.092473768
nanonet	fixed error	07 03	75.746	0.7661	0.28	0.1815	0.060013588	0.07737601
nanonet	fixed error	07 04	101.954	0.744	0.3167	0.1835	0.060013588	0.068090889
nanonet	fixed error	07 05	124.305	0.7053	0.3397	0.2432	0.060013588	0.064882615
nanonet	fixed error	07 06	166.194	0.6252	0.4338	0.3021	0.060013588	0.064467427
nanonet	fixed error	08 01	20.355	0.7683	0.2406	0.2226	0.060013588	0.126972145
nanonet	fixed error	08 02	39.634	0.7756	0.2618	0.1829	0.060013588	0.10013588
nanonet	fixed error	08 03	58.483	0.7724	0.2748	0.1739	0.060013588	0.081301427
nanonet	fixed error	08 04	81.601	0.753	0.2986	0.1871	0.060013588	0.070015853
nanonet	fixed error	08 05	111.41	0.7156	0.3409	0.2173	0.060013588	0.065335548
nanonet	fixed error	08 06	156.233	0.6499	0.4278	0.2479	0.060013588	0.064542915
nanonet	fixed size	06 01	60.721	0.7723	0.2635	0.1883	0.054918095	0.101909866
nanonet	fixed size	06 02	77.279	0.7645	0.274	0.1926	0.054918095	0.085717521
nanonet	fixed size	06 03	95.679	0.7478	0.2893	0.2111	0.054918095	0.073186382
nanonet	fixed size	06 04	122.093	0.7154	0.3288	0.2342	0.054918095	0.064052238
nanonet	fixed size	06 05	151.4	0.6434	0.3981	0.3089	0.054918095	0.059938099
nanonet	fixed size	06 06	182.049	0.4471	0.5683	0.5365	0.054918095	0.059183211
nanonet	fixed size	07 01	53.519	0.7792	0.2549	0.1835	0.054918095	0.098739337
nanonet	fixed size	07 02	71.135	0.7756	0.2643	0.1799	0.054918095	0.082207292
nanonet	fixed size	07 03	90.123	0.7637	0.2755	0.1925	0.054918095	0.069676153
nanonet	fixed size	07 04	120.463	0.7419	0.3143	0.192	0.054918095	0.062655696
nanonet	fixed size	07 05	150.077	0.7024	0.3499	0.2361	0.054918095	0.059673888
nanonet	fixed size	07 06	192.514	0.6196	0.436	0.3125	0.054918095	0.059183211
nanonet	fixed size	08 01	35.518	0.7697	0.2617	0.1961	0.054918095	0.11104401
nanonet	fixed size	08 02	51.589	0.7766	0.2619	0.1806	0.054918095	0.087906696
nanonet	fixed size	08 03	72.837	0.7716	0.2752	0.1751	0.054918095	0.072129539
nanonet	fixed size	08 04	99.166	0.7522	0.3003	0.1868	0.054918095	0.063750283
nanonet	fixed size	08 05	132.213	0.7151	0.3413	0.218	0.054918095	0.060089077
nanonet	fixed size	08 06	182.676	0.6484	0.4328	0.2433	0.054918095	0.059220956
nanonet	unfiltered	06 01	69.829	0.7587	0.2635	0.2176	0.051294633	0.096587907
nanonet	unfiltered	06 02	91.359	0.7479	0.2814	0.2202	0.051294633	0.081339171
nanonet	unfiltered	06 03	117.539	0.7273	0.3112	0.2296	0.051294633	0.068657054
nanonet	unfiltered	06 04	144.685	0.6909	0.3472	0.2662	0.051294633	0.059749377

Supplementary Table 5: Comparison of base callers without phasing / Part 3 of 3

basecaller	filtering	RH/RA	threshold	f measure	false negative rate	false discovery rate	false negative rate before optimization	false negative rate after optimization
metrichor	fixed size	06 03	4.227	0.8431	0.1782	0.1345	0.05178531	0.137276364
metrichor	fixed size	06 04	13.973	0.8644	0.162	0.1076	0.05178531	0.100060391
metrichor	fixed size	06 05	25.604	0.8602	0.1619	0.1166	0.05178531	0.074771646
metrichor	fixed size	06 06	46.836	0.6027	0.4073	0.3869	0.05178531	0.062278252
metrichor	fixed size	07 01	1.001	0.7379	0.3062	0.2121	0.05178531	0.242847437
metrichor	fixed size	07 02	1.044	0.7856	0.2416	0.1853	0.05178531	0.194912056
metrichor	fixed size	07 03	1.25	0.8254	0.182	0.1671	0.05178531	0.146674719
metrichor	fixed size	07 04	5.64	0.8589	0.1471	0.1351	0.05178531	0.101947611
metrichor	fixed size	07 05	21.419	0.8748	0.1401	0.1098	0.05178531	0.074394202
metrichor	fixed size	07 06	56.236	0.8439	0.1868	0.123	0.05178531	0.062806673
metrichor	fixed size	08 01	1	0.6316	0.4305	0.2912	0.05178531	0.312259379
metrichor	fixed size	08 02	1.003	0.6935	0.3561	0.2487	0.05178531	0.261342191
metrichor	fixed size	08 03	1.007	0.7567	0.2709	0.2135	0.05178531	0.198912961
metrichor	fixed size	08 04	2.011	0.8123	0.1947	0.1805	0.05178531	0.135011701
metrichor	fixed size	08 05	13.292	0.8494	0.1633	0.1375	0.05178531	0.089039028
metrichor	fixed size	08 06	53.897	0.8374	0.2097	0.1097	0.05178531	0.068128633
metrichor	unfiltered	06 01	1.264	0.7645	0.2743	0.1924	0.049784857	0.23084472
metrichor	unfiltered	06 02	2.328	0.8045	0.2253	0.1634	0.049784857	0.184758813
metrichor	unfiltered	06 03	5.663	0.8398	0.1834	0.1356	0.049784857	0.138937118
metrichor	unfiltered	06 04	15.194	0.8631	0.1614	0.1109	0.049784857	0.099305503
metrichor	unfiltered	06 05	25.849	0.8554	0.1591	0.1297	0.049784857	0.07239375
metrichor	unfiltered	06 06	55.17	0.5954	0.4142	0.3948	0.049784857	0.060466521
metrichor	unfiltered	07 01	1	0.7241	0.3184	0.2277	0.049784857	0.251000226
metrichor	unfiltered	07 02	1.002	0.7744	0.2496	0.1999	0.049784857	0.199781083
metrichor	unfiltered	07 03	2.71	0.8197	0.1948	0.1652	0.049784857	0.147316374
metrichor	unfiltered	07 04	10.508	0.8569	0.1581	0.1275	0.049784857	0.100173624
metrichor	unfiltered	07 05	23.93	0.871	0.1395	0.1183	0.049784857	0.071638862
metrichor	unfiltered	07 06	64.265	0.8349	0.1921	0.1362	0.049784857	0.060919453
metrichor	unfiltered	08 01	1.006	0.6155	0.4455	0.3083	0.049784857	0.323620442
metrichor	unfiltered	08 02	1.003	0.6796	0.3684	0.2645	0.049784857	0.269381747
metrichor	unfiltered	08 03	1.01	0.7463	0.2784	0.2273	0.049784857	0.202460935
metrichor	unfiltered	08 04	3.69	0.8041	0.2078	0.1836	0.049784857	0.134219068
metrichor	unfiltered	08 05	13.726	0.8436	0.1605	0.1522	0.049784857	0.086925342
metrichor	unfiltered	08 06	59.828	0.8288	0.2106	0.1276	0.049784857	0.065562014
nanonet	fixed error	06 01	45.666	0.7752	0.2557	0.1911	0.060013588	0.114290028
nanonet	fixed error	06 02	63.309	0.7686	0.275	0.1823	0.060013588	0.095795274
nanonet	fixed error	06 03	78.893	0.7535	0.2866	0.2015	0.060013588	0.080961727
nanonet	fixed error	06 04	100.897	0.7208	0.3218	0.2309	0.060013588	0.069940364
nanonet	fixed error	06 05	125.201	0.6526	0.3826	0.3079	0.060013588	0.065373292
nanonet	fixed error	06 06	154.795	0.4472	0.5676	0.537	0.060013588	0.064429682
nanonet	fixed error	07 01	42.815	0.7801	0.2575	0.1783	0.060013588	0.111534687
nanonet	fixed error	07 02	57.086	0.777	0.2644	0.1765	0.060013588	0.092473768
nanonet	fixed error	07 03	75.746	0.7661	0.28	0.1815	0.060013588	0.07737601
nanonet	fixed error	07 04	101.954	0.744	0.3167	0.1835	0.060013588	0.068090889
nanonet	fixed error	07 05	124.305	0.7053	0.3397	0.2432	0.060013588	0.064882615
nanonet	fixed error	07 06	166.194	0.6252	0.4338	0.3021	0.060013588	0.064467427
nanonet	fixed error	08 01	20.355	0.7683	0.2406	0.2226	0.060013588	0.126972145
nanonet	fixed error	08 02	39.634	0.7756	0.2618	0.1829	0.060013588	0.10013588
nanonet	fixed error	08 03	58.483	0.7724	0.2748	0.1739	0.060013588	0.081301427
nanonet	fixed error	08 04	81.601	0.753	0.2986	0.1871	0.060013588	0.070015853
nanonet	fixed error	08 05	111.41	0.7156	0.3409	0.2173	0.060013588	0.065335548
nanonet	fixed error	08 06	156.233	0.6499	0.4278	0.2479	0.060013588	0.064542915
nanonet	fixed size	06 01	60.721	0.7723	0.2635	0.1883	0.054918095	0.101909866
nanonet	fixed size	06 02	77.279	0.7645	0.274	0.1926	0.054918095	0.085717521
nanonet	fixed size	06 03	95.679	0.7478	0.2893	0.2111	0.054918095	0.073186382
nanonet	fixed size	06 04	122.093	0.7154	0.3288	0.2342	0.054918095	0.064052238
nanonet	fixed size	06 05	151.4	0.6434	0.3981	0.3089	0.054918095	0.059938099
nanonet	fixed size	06 06	182.049	0.4471	0.5683	0.5365	0.054918095	0.059183211
nanonet	fixed size	07 01	53.519	0.7792	0.2549	0.1835	0.054918095	0.098739337
nanonet	fixed size	07 02	71.135	0.7756	0.2643	0.1799	0.054918095	0.082207292
nanonet	fixed size	07 03	90.123	0.7637	0.2755	0.1925	0.054918095	0.069676153
nanonet	fixed size	07 04	120.463	0.7419	0.3143	0.192	0.054918095	0.062655696
nanonet	fixed size	07 05	150.077	0.7024	0.3499	0.2361	0.054918095	0.059673888
nanonet	fixed size	07 06	192.514	0.6196	0.436	0.3125	0.054918095	0.059183211
nanonet	fixed size	08 01	35.518	0.7697	0.2617	0.1961	0.054918095	0.11104401
nanonet	fixed size	08 02	51.589	0.7766	0.2619	0.1806	0.054918095	0.087906696
nanonet	fixed size	08 03	72.837	0.7716	0.2752	0.1751	0.054918095	0.072129539
nanonet	fixed size	08 04	99.166	0.7522	0.3003	0.1868	0.054918095	0.063750283
nanonet	fixed size	08 05	132.213	0.7151	0.3413	0.218	0.054918095	0.060089077
nanonet	fixed size	08 06	182.676	0.6484	0.4328	0.2433	0.054918095	0.059220956
nanonet	unfiltered	06 01	69.829	0.7587	0.2635	0.2176	0.051294633	0.096587907
nanonet	unfiltered	06 02	91.359	0.7479	0.2814	0.2202	0.051294633	0.081339171
nanonet	unfiltered	06 03	117.539	0.7273	0.3112	0.2296	0.051294633	0.068657054
nanonet	unfiltered	06 04	144.685	0.6909	0.3472	0.2662	0.051294633	0.059749377

Supplementary Table 6: Comparison of base callers with phasing / Part 1 of 5 Variant calling results for different base callers using different QUAL filtering options with phasing information. For example, "QUAL > 10 & GT != 0|0 & (0 < SB1&2 < 1) & ((PE1 + 0.5 * QD) > 2.5)" indicates first removing keeping and phasing only sites with QUAL > 10, then after phasing, keep sites where the genotype was not 0|0, strand bias was neither 0 nor 1, and using the phase entropy metric and QD (quality by depth) metrics, (PE1 + 0.5 * QD) > 2.5.

Basecaller	Filtration Approach	F1 score	FDR	FNR	Condition
Albacore	Heuristic with phasing	0.828	0.257	0.066	QUAL > 10
Albacore	Heuristic with phasing	0.867	0.19	0.068	QUAL > 10 & ((PE1 + 0.5 * QD) > 2)
Albacore	Heuristic with phasing	0.874	0.175	0.07	QUAL > 10 & ((PE1 + 0.5 * QD) > 2.25)
Albacore	Heuristic with phasing	0.879	0.163	0.074	QUAL > 10 & ((PE1 + 0.5 * QD) > 2.5)
Albacore	Heuristic with phasing	0.865	0.187	0.076	QUAL > 10 & (max(SRP, SAP) < 30)
Albacore	Heuristic with phasing	0.878	0.162	0.078	QUAL > 10 & (max(SRP, SAP) < 30) & ((PE1 + 0.5 * QD) > 2)
Albacore	Heuristic with phasing	0.882	0.153	0.08	QUAL > 10 & (max(SRP, SAP) < 30) & ((PE1 + 0.5 * QD) > 2.25)
Albacore	Heuristic with phasing	0.886	0.143	0.084	QUAL > 10 & (max(SRP, SAP) < 30) & ((PE1 + 0.5 * QD) > 2.5)
Albacore	Heuristic with phasing	0.826	0.25	0.08	QUAL > 10 & (0 < SB1&2 < 1)
Albacore	Heuristic with phasing	0.866	0.181	0.082	QUAL > 10 & (0 < SB1&2 < 1) & ((PE1 + 0.5 * QD) > 2)
Albacore	Heuristic with phasing	0.873	0.166	0.084	QUAL > 10 & (0 < SB1&2 < 1) & ((PE1 + 0.5 * QD) > 2.25)
Albacore	Heuristic with phasing	0.878	0.154	0.088	QUAL > 10 & (0 < SB1&2 < 1) & ((PE1 + 0.5 * QD) > 2.5)
Albacore	Heuristic with phasing	0.861	0.183	0.09	QUAL > 10 & (0 < SB1&2 < 1) & (max(SRP, SAP) < 30)
Albacore	Heuristic with phasing	0.874	0.157	0.092	QUAL > 10 & (0 < SB1&2 < 1) & (max(SRP, SAP) < 30) & ((PE1 + 0.5 * QD) > 2)
Albacore	Heuristic with phasing	0.878	0.148	0.094	QUAL > 10 & (0 < SB1&2 < 1) & (max(SRP, SAP) < 30) & ((PE1 + 0.5 * QD) > 2.25)
Albacore	Heuristic with phasing	0.881	0.139	0.098	QUAL > 10 & (0 < SB1&2 < 1) & (max(SRP, SAP) < 30) & ((PE1 + 0.5 * QD) > 2.5)
Albacore	Heuristic with phasing	0.91	0.106	0.074	QUAL > 10 & GT != 0|0
Albacore	Heuristic with phasing	0.918	0.089	0.075	QUAL > 10 & GT != 0|0 & ((PE1 + 0.5 * QD) > 2)
Albacore	Heuristic with phasing	0.92	0.083	0.076	QUAL > 10 & GT != 0|0 & ((PE1 + 0.5 * QD) > 2.25)
Albacore	Heuristic with phasing	0.921	0.078	0.079	QUAL > 10 & GT != 0|0 & ((PE1 + 0.5 * QD) > 2.5)
Albacore	Heuristic with phasing	0.924	0.067	0.085	QUAL > 10 & GT != 0|0 & (max(SRP, SAP) < 30)
Albacore	Heuristic with phasing	0.927	0.06	0.085	QUAL > 10 & GT != 0|0 & (max(SRP, SAP) < 30) & ((PE1 + 0.5 * QD) > 2)
Albacore	Heuristic with phasing	0.927	0.058	0.087	QUAL > 10 & GT != 0|0 & (max(SRP, SAP) < 30) & ((PE1 + 0.5 * QD) > 2.25)
Albacore	Heuristic with phasing	0.927	0.056	0.089	QUAL > 10 & GT != 0|0 & (max(SRP, SAP) < 30) & ((PE1 + 0.5 * QD) > 2.5)
Albacore	Heuristic with phasing	0.909	0.094	0.088	QUAL > 10 & GT != 0|0 & (0 < SB1&2 < 1)
Albacore	Heuristic with phasing	0.917	0.077	0.089	QUAL > 10 & GT != 0|0 & (0 < SB1&2 < 1) & ((PE1 + 0.5 * QD) > 2)
Albacore	Heuristic with phasing	0.919	0.071	0.09	QUAL > 10 & GT != 0|0 & (0 < SB1&2 < 1) & ((PE1 + 0.5 * QD) > 2.25)
Albacore	Heuristic with phasing	0.92	0.067	0.093	QUAL > 10 & GT != 0|0 & (0 < SB1&2 < 1) & ((PE1 + 0.5 * QD) > 2.5)
Albacore	Heuristic with phasing	0.92	0.062	0.098	QUAL > 10 & GT != 0|0 & (0 < SB1&2 < 1) & (max(SRP, SAP) < 30)
Albacore	Heuristic with phasing	0.922	0.055	0.099	QUAL > 10 & GT != 0|0 & (0 < SB1&2 < 1) & (max(SRP, SAP) < 30) & ((PE1 + 0.5 * QD) > 2)
Albacore	Heuristic with phasing	0.923	0.053	0.1	QUAL > 10 & GT != 0|0 & (0 < SB1&2 < 1) & (max(SRP, SAP) < 30) & ((PE1 + 0.5 * QD) > 2.25)
Albacore	Heuristic with phasing	0.922	0.051	0.103	QUAL > 10 & GT != 0|0 & (0 < SB1&2 < 1) & (max(SRP, SAP) < 30) & ((PE1 + 0.5 * QD) > 2.5)
Metrichor	Heuristic with phasing	0.876	0.142	0.106	QUAL > 10
Metrichor	Heuristic with phasing	0.891	0.108	0.109	QUAL > 10 & ((PE1 + 0.5 * QD) > 2)
Metrichor	Heuristic with phasing	0.894	0.1	0.112	QUAL > 10 & ((PE1 + 0.5 * QD) > 2.25)
Metrichor	Heuristic with phasing	0.895	0.094	0.116	QUAL > 10 & ((PE1 + 0.5 * QD) > 2.5)
Metrichor	Heuristic with phasing	0.878	0.123	0.12	QUAL > 10 & (max(SRP, SAP) < 30)
Metrichor	Heuristic with phasing	0.888	0.101	0.122	QUAL > 10 & (max(SRP, SAP) < 30) & ((PE1 + 0.5 * QD) > 2)
Metrichor	Heuristic with phasing	0.89	0.094	0.125	QUAL > 10 & (max(SRP, SAP) < 30) & ((PE1 + 0.5 * QD) > 2.25)
Metrichor	Heuristic with phasing	0.891	0.088	0.129	QUAL > 10 & (max(SRP, SAP) < 30) & ((PE1 + 0.5 * QD) > 2.5)
Metrichor	Heuristic with phasing	0.879	0.129	0.113	QUAL > 10 & (0 < SB1&2 < 1)
Metrichor	Heuristic with phasing	0.894	0.095	0.116	QUAL > 10 & (0 < SB1&2 < 1) & ((PE1 + 0.5 * QD) > 2)
Metrichor	Heuristic with phasing	0.897	0.087	0.119	QUAL > 10 & (0 < SB1&2 < 1) & ((PE1 + 0.5 * QD) > 2.25)
Metrichor	Heuristic with phasing	0.898	0.081	0.123	QUAL > 10 & (0 < SB1&2 < 1) & ((PE1 + 0.5 * QD) > 2.5)
Metrichor	Heuristic with phasing	0.881	0.11	0.127	QUAL > 10 & (0 < SB1&2 < 1) & (max(SRP, SAP) < 30)
Metrichor	Heuristic with phasing	0.89	0.089	0.13	QUAL > 10 & (0 < SB1&2 < 1) & (max(SRP, SAP) < 30) & ((PE1 + 0.5 * QD) > 2)
Metrichor	Heuristic with phasing	0.893	0.081	0.132	QUAL > 10 & (0 < SB1&2 < 1) & (max(SRP, SAP) < 30) & ((PE1 + 0.5 * QD) > 2.25)
Metrichor	Heuristic with phasing	0.893	0.076	0.136	QUAL > 10 & (0 < SB1&2 < 1) & (max(SRP, SAP) < 30) & ((PE1 + 0.5 * QD) > 2.5)
Metrichor	Heuristic with phasing	0.896	0.096	0.111	QUAL > 10 & GT != 0|0
Metrichor	Heuristic with phasing	0.901	0.084	0.113	QUAL > 10 & GT != 0|0 & ((PE1 + 0.5 * QD) > 2)
Metrichor	Heuristic with phasing	0.903	0.079	0.115	QUAL > 10 & GT != 0|0 & ((PE1 + 0.5 * QD) > 2.25)
Metrichor	Heuristic with phasing	0.902	0.076	0.119	QUAL > 10 & GT != 0|0 & ((PE1 + 0.5 * QD) > 2.5)
Metrichor	Heuristic with phasing	0.893	0.088	0.125	QUAL > 10 & GT != 0|0 & (max(SRP, SAP) < 30)
Metrichor	Heuristic with phasing	0.897	0.079	0.126	QUAL > 10 & GT != 0|0 & (max(SRP, SAP) < 30) & ((PE1 + 0.5 * QD) > 2)
Metrichor	Heuristic with phasing	0.898	0.074	0.128	QUAL > 10 & GT != 0|0 & (max(SRP, SAP) < 30) & ((PE1 + 0.5 * QD) > 2.25)
Metrichor	Heuristic with phasing	0.897	0.071	0.132	QUAL > 10 & GT != 0|0 & (max(SRP, SAP) < 30) & ((PE1 + 0.5 * QD) > 2.5)
Metrichor	Heuristic with phasing	0.899	0.082	0.119	QUAL > 10 & GT != 0|0 & (0 < SB1&2 < 1)
Metrichor	Heuristic with phasing	0.904	0.071	0.12	QUAL > 10 & GT != 0|0 & (0 < SB1&2 < 1) & ((PE1 + 0.5 * QD) > 2)
Metrichor	Heuristic with phasing	0.905	0.066	0.122	QUAL > 10 & GT != 0|0 & (0 < SB1&2 < 1) & ((PE1 + 0.5 * QD) > 2.25)
Metrichor	Heuristic with phasing	0.905	0.062	0.126	QUAL > 10 & GT != 0|0 & (0 < SB1&2 < 1) & ((PE1 + 0.5 * QD) > 2.5)
Metrichor	Heuristic with phasing	0.896	0.074	0.132	QUAL > 10 & GT != 0|0 & (0 < SB1&2 < 1) & (max(SRP, SAP) < 30)
Metrichor	Heuristic with phasing	0.899	0.066	0.134	QUAL > 10 & GT != 0|0 & (0 < SB1&2 < 1) & (max(SRP, SAP) < 30) & ((PE1 + 0.5 * QD) > 2)
Metrichor	Heuristic with phasing	0.9	0.061	0.135	QUAL > 10 & GT != 0|0 & (0 < SB1&2 < 1) & (max(SRP, SAP) < 30) & ((PE1 + 0.5 * QD) > 2.25)
Metrichor	Heuristic with phasing	0.9	0.058	0.139	QUAL > 10 & GT != 0|0 & (0 < SB1&2 < 1) & (max(SRP, SAP) < 30) & ((PE1 + 0.5 * QD) > 2.5)
Nanonet	Heuristic with phasing	0.708	0.4	0.138	QUAL > 10
Nanonet	Heuristic with phasing	0.818	0.216	0.145	QUAL > 10 & ((PE1 + 0.5 * QD) > 2)
Nanonet	Heuristic with phasing	0.831	0.185	0.152	QUAL > 10 & ((PE1 + 0.5 * QD) > 2.25)
Nanonet	Heuristic with phasing	0.836	0.166	0.161	QUAL > 10 & ((PE1 + 0.5 * QD) > 2.5)
Nanonet	Heuristic with phasing	0.789	0.263	0.15	QUAL > 10 & (max(SRP, SAP) < 30)
Nanonet	Heuristic with phasing	0.846	0.151	0.156	QUAL > 10 & (max(SRP, SAP) < 30) & ((PE1 + 0.5 * QD) > 2)
Nanonet	Heuristic with phasing	0.853	0.131	0.163	QUAL > 10 & (max(SRP, SAP) < 30) & ((PE1 + 0.5 * QD) > 2.25)
Nanonet	Heuristic with phasing	0.853	0.12	0.172	QUAL > 10 & (max(SRP, SAP) < 30) & ((PE1 + 0.5 * QD) > 2.5)
Nanonet	Heuristic with phasing	0.721	0.365	0.165	QUAL > 10 & (0 < SB1&2 < 1)
Nanonet	Heuristic with phasing	0.836	0.157	0.171	QUAL > 10 & (0 < SB1&2 < 1) & ((PE1 + 0.5 * QD) > 2)
Nanonet	Heuristic with phasing	0.847	0.126	0.178	QUAL > 10 & (0 < SB1&2 < 1) & ((PE1 + 0.5 * QD) > 2.25)
Nanonet	Heuristic with phasing	0.85	0.109	0.187	QUAL > 10 & (0 < SB1&2 < 1) & ((PE1 + 0.5 * QD) > 2.5)
Nanonet	Heuristic with phasing	0.798	0.226	0.176	QUAL > 10 & (0 < SB1&2 < 1) & (max(SRP, SAP) < 30)
Nanonet	Heuristic with phasing	0.854	0.107	0.182	QUAL > 10 & (0 < SB1&2 < 1) & (max(SRP, SAP) < 30) & ((PE1 + 0.5 * QD) > 2)
Nanonet	Heuristic with phasing	0.86	0.087	0.188	QUAL > 10 & (0 < SB1&2 < 1) & (max(SRP, SAP) < 30) & ((PE1 + 0.5 * QD) > 2.25)
Nanonet	Heuristic with phasing	0.859	0.077	0.197	QUAL > 10 & (0 < SB1&2 < 1) & (max(SRP, SAP) < 30) & ((PE1 + 0.5 * QD) > 2.5)
Nanonet	Heuristic with phasing	0.789	0.268	0.145	QUAL > 10 & GT != 0|0
Nanonet	Heuristic with phasing	0.824	0.203	0.148	QUAL > 10 & GT != 0|0 & ((PE1 + 0.5 * QD) > 2)
Nanonet	Heuristic with phasing	0.835	0.177	0.153	QUAL > 10 & GT != 0|0 & ((PE1 + 0.5 * QD) > 2.25)
Nanonet	Heuristic with phasing	0.839	0.159	0.162	QUAL > 10 & GT != 0|0 & ((PE1 + 0.5 * QD) > 2.5)
Nanonet	Heuristic with phasing	0.836	0.172	0.156	QUAL > 10 & GT != 0|0 & (max(SRP, SAP) < 30)
Nanonet	Heuristic with phasing	0.85	0.14	0.159	QUAL > 10 & GT != 0|0 & (max(SRP, SAP) < 30) & ((PE1 + 0.5 * QD) > 2)
Nanonet	Heuristic with phasing	0.855	0.124	0.165	QUAL > 10 & GT != 0|0 & (max(SRP, SAP) < 30) & ((PE1 + 0.5 * QD) > 2.25)
Nanonet	Heuristic with phasing	0.855	0.114	0.173	QUAL > 10 & GT != 0|0 & (max(SRP, SAP) < 30) & ((PE1 + 0.5 * QD) > 2.5)
Nanonet	Heuristic with phasing	0.808	0.212	0.171	QUAL > 10 & GT != 0|0 & (0 < SB1&2 < 1)
Nanonet	Heuristic with phasing	0.841	0.143	0.174	QUAL > 10 & GT != 0|0 & (0 < SB1&2 < 1) & ((PE1 + 0.5 * QD) > 2)
Nanonet	Heuristic with phasing	0.85	0.118	0.179	QUAL > 10 & GT != 0|0 & (0 < SB1&2 < 1) & ((PE1 + 0.5 * QD) > 2.25)
Nanonet	Heuristic with phasing	0.853	0.102	0.188	QUAL > 10 & GT != 0|0 & (0 < SB1&2 < 1) & ((PE1 + 0.5 * QD) > 2.5)
Nanonet	Heuristic with phasing	0.846	0.125	0.182	QUAL > 10 & GT != 0|0 & (0 < SB1&2 < 1) & (max(SRP, SAP) < 30)
Nanonet	Heuristic with phasing	0.858	0.095	0.184	QUAL > 10 & GT != 0|0 & (0 < SB1&2 < 1) & (max(SRP, SAP) < 30) & ((PE1 + 0.5 * QD) > 2)
Nanonet	Heuristic with phasing	0.862	0.08	0.189	QUAL > 10 & GT != 0|0 & (0 < SB1&2 < 1) & (max(SRP, SAP) < 30) & ((PE1 + 0.5 * QD) > 2.25)
Nanonet	Heuristic with phasing	0.861	0.071	0.198	QUAL > 10 & GT != 0|0 & (0 < SB1&2 < 1) & (max(SRP, SAP) < 30) & ((PE1 + 0.5 * QD) > 2.5)
Scrappie	Heuristic with phasing	0.667	0.462	0.122	QUAL > 10
Scrappie	Heuristic with phasing	0.669	0.46	0.122	QUAL > 10 & ((PE1 + 0.5 * QD) > 2)
Scrappie	Heuristic with phasing	0.668	0.46	0.123	QUAL > 10 & ((PE1 + 0.5 * QD) > 2.25)
Scrappie	Heuristic with phasing	0.669	0.459	0.123	QUAL > 10 & ((PE1 + 0.5 * QD) > 2.5)
Scrappie	Heuristic with phasing	0.7	0.413	0.132	QUAL > 10 & (max(SRP, SAP) < 30)
Scrappie	Heuristic with phasing	0.701	0.412	0.133	QUAL > 10 & (max(SRP, SAP) < 30) & ((PE1 + 0.5 * QD) > 2)
Scrappie	Heuristic with phasing	0.701	0.412	0.133	QUAL > 10 & (max(SRP, SAP) < 30) & ((PE1 + 0.5 * QD) > 2.25)
Scrappie	Heuristic with phasing	0.701	0.411	0.133	QUAL > 10 & (max(SRP, SAP) < 30) & ((PE1 + 0.5 * QD) > 2.5)

Supplementary Table 6: Comparison of base callers with phasing / Part 2 of 5

<table>
<tr><th>Basecaller</th><th>Filtration Approach</th><th>F1 score</th><th>FDR</th><th>FNR</th><th>Condition</th></tr>
<tr><td>Scrappie</td><td>Heuristic with phasing</td><td>0.666</td><td>0.461</td><td>0.127</td><td>QUAL > 10 & (0 < SB1&2 < 1)</td></tr>
<tr><td>Scrappie</td><td>Heuristic with phasing</td><td>0.667</td><td>0.46</td><td>0.127</td><td>QUAL > 10 & (0 < SB1&2 < 1) & ((PE1 + 0.5 * QD) > 2)</td></tr>
<tr><td>Scrappie</td><td>Heuristic with phasing</td><td>0.668</td><td>0.459</td><td>0.127</td><td>QUAL > 10 & (0 < SB1&2 < 1) & ((PE1 + 0.5 * QD) > 2.25)</td></tr>
<tr><td>Scrappie</td><td>Heuristic with phasing</td><td>0.669</td><td>0.458</td><td>0.127</td><td>QUAL > 10 & (0 < SB1&2 < 1) & ((PE1 + 0.5 * QD) > 2.5)</td></tr>
<tr><td>Scrappie</td><td>Heuristic with phasing</td><td>0.699</td><td>0.412</td><td>0.137</td><td>QUAL > 10 & (0 < SB1&2 < 1) & (max(SRP, SAP) < 30)</td></tr>
<tr><td>Scrappie</td><td>Heuristic with phasing</td><td>0.699</td><td>0.412</td><td>0.137</td><td>QUAL > 10 & (0 < SB1&2 < 1) & (max(SRP, SAP) < 30) & ((PE1 + 0.5 * QD) > 2)</td></tr>
<tr><td>Scrappie</td><td>Heuristic with phasing</td><td>0.7</td><td>0.411</td><td>0.137</td><td>QUAL > 10 & (0 < SB1&2 < 1) & (max(SRP, SAP) < 30) & ((PE1 + 0.5 * QD) > 2.25)</td></tr>
<tr><td>Scrappie</td><td>Heuristic with phasing</td><td>0.7</td><td>0.411</td><td>0.137</td><td>QUAL > 10 & (0 < SB1&2 < 1) & (max(SRP, SAP) < 30) & ((PE1 + 0.5 * QD) > 2.5)</td></tr>
<tr><td>Scrappie</td><td>Heuristic with phasing</td><td>0.87</td><td>0.107</td><td>0.151</td><td>QUAL > 10 & GT != 0|0</td></tr>
<tr><td>Scrappie</td><td>Heuristic with phasing</td><td>0.871</td><td>0.105</td><td>0.151</td><td>QUAL > 10 & GT != 0|0 & ((PE1 + 0.5 * QD) > 2)</td></tr>
<tr><td>Scrappie</td><td>Heuristic with phasing</td><td>0.872</td><td>0.104</td><td>0.151</td><td>QUAL > 10 & GT != 0|0 & ((PE1 + 0.5 * QD) > 2.25)</td></tr>
<tr><td>Scrappie</td><td>Heuristic with phasing</td><td>0.873</td><td>0.102</td><td>0.151</td><td>QUAL > 10 & GT != 0|0 & ((PE1 + 0.5 * QD) > 2.5)</td></tr>
<tr><td>Scrappie</td><td>Heuristic with phasing</td><td>0.876</td><td>0.084</td><td>0.16</td><td>QUAL > 10 & GT != 0|0 & (max(SRP, SAP) < 30)</td></tr>
<tr><td>Scrappie</td><td>Heuristic with phasing</td><td>0.876</td><td>0.083</td><td>0.161</td><td>QUAL > 10 & GT != 0|0 & (max(SRP, SAP) < 30) & ((PE1 + 0.5 * QD) > 2)</td></tr>
<tr><td>Scrappie</td><td>Heuristic with phasing</td><td>0.877</td><td>0.082</td><td>0.161</td><td>QUAL > 10 & GT != 0|0 & (max(SRP, SAP) < 30) & ((PE1 + 0.5 * QD) > 2.25)</td></tr>
<tr><td>Scrappie</td><td>Heuristic with phasing</td><td>0.877</td><td>0.081</td><td>0.161</td><td>QUAL > 10 & GT != 0|0 & (max(SRP, SAP) < 30) & ((PE1 + 0.5 * QD) > 2.5)</td></tr>
<tr><td>Scrappie</td><td>Heuristic with phasing</td><td>0.87</td><td>0.104</td><td>0.155</td><td>QUAL > 10 & GT != 0|0 & (0 < SB1&2 < 1)</td></tr>
<tr><td>Scrappie</td><td>Heuristic with phasing</td><td>0.871</td><td>0.102</td><td>0.155</td><td>QUAL > 10 & GT != 0|0 & (0 < SB1&2 < 1) & ((PE1 + 0.5 * QD) > 2)</td></tr>
<tr><td>Scrappie</td><td>Heuristic with phasing</td><td>0.872</td><td>0.1</td><td>0.155</td><td>QUAL > 10 & GT != 0|0 & (0 < SB1&2 < 1) & ((PE1 + 0.5 * QD) > 2.25)</td></tr>
<tr><td>Scrappie</td><td>Heuristic with phasing</td><td>0.872</td><td>0.099</td><td>0.156</td><td>QUAL > 10 & GT != 0|0 & (0 < SB1&2 < 1) & ((PE1 + 0.5 * QD) > 2.5)</td></tr>
<tr><td>Scrappie</td><td>Heuristic with phasing</td><td>0.876</td><td>0.081</td><td>0.164</td><td>QUAL > 10 & GT != 0|0 & (0 < SB1&2 < 1) & (max(SRP, SAP) < 30)</td></tr>
<tr><td>Scrappie</td><td>Heuristic with phasing</td><td>0.875</td><td>0.08</td><td>0.165</td><td>QUAL > 10 & GT != 0|0 & (0 < SB1&2 < 1) & (max(SRP, SAP) < 30) & ((PE1 + 0.5 * QD) > 2)</td></tr>
<tr><td>Scrappie</td><td>Heuristic with phasing</td><td>0.876</td><td>0.079</td><td>0.165</td><td>QUAL > 10 & GT != 0|0 & (0 < SB1&2 < 1) & (max(SRP, SAP) < 30) & ((PE1 + 0.5 * QD) > 2.25)</td></tr>
<tr><td>Scrappie</td><td>Heuristic with phasing</td><td>0.876</td><td>0.078</td><td>0.165</td><td>QUAL > 10 & GT != 0|0 & (0 < SB1&2 < 1) & (max(SRP, SAP) < 30) & ((PE1 + 0.5 * QD) > 2.5)</td></tr>
<tr><td>Albacore</td><td>Heuristic with phasing</td><td>0.798</td><td>0.309</td><td>0.057</td><td>QUAL > 1</td></tr>
<tr><td>Albacore</td><td>Heuristic with phasing</td><td>0.857</td><td>0.211</td><td>0.061</td><td>QUAL > 1 & ((PE1 + 0.5 * QD) > 2)</td></tr>
<tr><td>Albacore</td><td>Heuristic with phasing</td><td>0.866</td><td>0.195</td><td>0.063</td><td>QUAL > 1 & ((PE1 + 0.5 * QD) > 2.25)</td></tr>
<tr><td>Albacore</td><td>Heuristic with phasing</td><td>0.872</td><td>0.181</td><td>0.067</td><td>QUAL > 1 & ((PE1 + 0.5 * QD) > 2.5)</td></tr>
<tr><td>Albacore</td><td>Heuristic with phasing</td><td>0.849</td><td>0.221</td><td>0.068</td><td>QUAL > 1 & (max(SRP, SAP) < 30)</td></tr>
<tr><td>Albacore</td><td>Heuristic with phasing</td><td>0.873</td><td>0.176</td><td>0.071</td><td>QUAL > 1 & (max(SRP, SAP) < 30) & ((PE1 + 0.5 * QD) > 2)</td></tr>
<tr><td>Albacore</td><td>Heuristic with phasing</td><td>0.879</td><td>0.165</td><td>0.073</td><td>QUAL > 1 & (max(SRP, SAP) < 30) & ((PE1 + 0.5 * QD) > 2.25)</td></tr>
<tr><td>Albacore</td><td>Heuristic with phasing</td><td>0.883</td><td>0.154</td><td>0.077</td><td>QUAL > 1 & (max(SRP, SAP) < 30) & ((PE1 + 0.5 * QD) > 2.5)</td></tr>
<tr><td>Albacore</td><td>Heuristic with phasing</td><td>0.797</td><td>0.299</td><td>0.076</td><td>QUAL > 1 & (0 < SB1&2 < 1)</td></tr>
<tr><td>Albacore</td><td>Heuristic with phasing</td><td>0.857</td><td>0.199</td><td>0.079</td><td>QUAL > 1 & (0 < SB1&2 < 1) & ((PE1 + 0.5 * QD) > 2)</td></tr>
<tr><td>Albacore</td><td>Heuristic with phasing</td><td>0.865</td><td>0.182</td><td>0.082</td><td>QUAL > 1 & (0 < SB1&2 < 1) & ((PE1 + 0.5 * QD) > 2.25)</td></tr>
<tr><td>Albacore</td><td>Heuristic with phasing</td><td>0.871</td><td>0.168</td><td>0.086</td><td>QUAL > 1 & (0 < SB1&2 < 1) & ((PE1 + 0.5 * QD) > 2.5)</td></tr>
<tr><td>Albacore</td><td>Heuristic with phasing</td><td>0.845</td><td>0.215</td><td>0.086</td><td>QUAL > 1 & (0 < SB1&2 < 1) & (max(SRP, SAP) < 30)</td></tr>
<tr><td>Albacore</td><td>Heuristic with phasing</td><td>0.869</td><td>0.17</td><td>0.089</td><td>QUAL > 1 & (0 < SB1&2 < 1) & (max(SRP, SAP) < 30) & ((PE1 + 0.5 * QD) > 2)</td></tr>
<tr><td>Albacore</td><td>Heuristic with phasing</td><td>0.874</td><td>0.159</td><td>0.091</td><td>QUAL > 1 & (0 < SB1&2 < 1) & (max(SRP, SAP) < 30) & ((PE1 + 0.5 * QD) > 2.25)</td></tr>
<tr><td>Albacore</td><td>Heuristic with phasing</td><td>0.878</td><td>0.148</td><td>0.095</td><td>QUAL > 1 & (0 < SB1&2 < 1) & (max(SRP, SAP) < 30) & ((PE1 + 0.5 * QD) > 2.5)</td></tr>
<tr><td>Albacore</td><td>Heuristic with phasing</td><td>0.9</td><td>0.129</td><td>0.068</td><td>QUAL > 1 & GT != 0|0</td></tr>
<tr><td>Albacore</td><td>Heuristic with phasing</td><td>0.913</td><td>0.104</td><td>0.069</td><td>QUAL > 1 & GT != 0|0 & ((PE1 + 0.5 * QD) > 2)</td></tr>
<tr><td>Albacore</td><td>Heuristic with phasing</td><td>0.916</td><td>0.097</td><td>0.071</td><td>QUAL > 1 & GT != 0|0 & ((PE1 + 0.5 * QD) > 2.25)</td></tr>
<tr><td>Albacore</td><td>Heuristic with phasing</td><td>0.916</td><td>0.093</td><td>0.074</td><td>QUAL > 1 & GT != 0|0 & ((PE1 + 0.5 * QD) > 2.5)</td></tr>
<tr><td>Albacore</td><td>Heuristic with phasing</td><td>0.921</td><td>0.079</td><td>0.078</td><td>QUAL > 1 & GT != 0|0 & (max(SRP, SAP) < 30)</td></tr>
<tr><td>Albacore</td><td>Heuristic with phasing</td><td>0.927</td><td>0.066</td><td>0.079</td><td>QUAL > 1 & GT != 0|0 & (max(SRP, SAP) < 30) & ((PE1 + 0.5 * QD) > 2)</td></tr>
<tr><td>Albacore</td><td>Heuristic with phasing</td><td>0.927</td><td>0.064</td><td>0.081</td><td>QUAL > 1 & GT != 0|0 & (max(SRP, SAP) < 30) & ((PE1 + 0.5 * QD) > 2.25)</td></tr>
<tr><td>Albacore</td><td>Heuristic with phasing</td><td>0.927</td><td>0.062</td><td>0.084</td><td>QUAL > 1 & GT != 0|0 & (max(SRP, SAP) < 30) & ((PE1 + 0.5 * QD) > 2.5)</td></tr>
<tr><td>Albacore</td><td>Heuristic with phasing</td><td>0.901</td><td>0.112</td><td>0.086</td><td>QUAL > 1 & GT != 0|0 & (0 < SB1&2 < 1)</td></tr>
<tr><td>Albacore</td><td>Heuristic with phasing</td><td>0.912</td><td>0.088</td><td>0.087</td><td>QUAL > 1 & GT != 0|0 & (0 < SB1&2 < 1) & ((PE1 + 0.5 * QD) > 2)</td></tr>
<tr><td>Albacore</td><td>Heuristic with phasing</td><td>0.914</td><td>0.082</td><td>0.089</td><td>QUAL > 1 & GT != 0|0 & (0 < SB1&2 < 1) & ((PE1 + 0.5 * QD) > 2.25)</td></tr>
<tr><td>Albacore</td><td>Heuristic with phasing</td><td>0.915</td><td>0.077</td><td>0.092</td><td>QUAL > 1 & GT != 0|0 & (0 < SB1&2 < 1) & ((PE1 + 0.5 * QD) > 2.5)</td></tr>
<tr><td>Albacore</td><td>Heuristic with phasing</td><td>0.917</td><td>0.07</td><td>0.096</td><td>QUAL > 1 & GT != 0|0 & (0 < SB1&2 < 1) & (max(SRP, SAP) < 30)</td></tr>
<tr><td>Albacore</td><td>Heuristic with phasing</td><td>0.922</td><td>0.059</td><td>0.097</td><td>QUAL > 1 & GT != 0|0 & (0 < SB1&2 < 1) & (max(SRP, SAP) < 30) & ((PE1 + 0.5 * QD) > 2)</td></tr>
<tr><td>Albacore</td><td>Heuristic with phasing</td><td>0.922</td><td>0.057</td><td>0.098</td><td>QUAL > 1 & GT != 0|0 & (0 < SB1&2 < 1) & (max(SRP, SAP) < 30) & ((PE1 + 0.5 * QD) > 2.25)</td></tr>
<tr><td>Albacore</td><td>Heuristic with phasing</td><td>0.921</td><td>0.055</td><td>0.101</td><td>QUAL > 1 & GT != 0|0 & (0 < SB1&2 < 1) & (max(SRP, SAP) < 30) & ((PE1 + 0.5 * QD) > 2.5)</td></tr>
<tr><td>Metrichor</td><td>Heuristic with phasing</td><td>0.839</td><td>0.227</td><td>0.083</td><td>QUAL > 1</td></tr>
<tr><td>Metrichor</td><td>Heuristic with phasing</td><td>0.882</td><td>0.147</td><td>0.087</td><td>QUAL > 1 & ((PE1 + 0.5 * QD) > 2)</td></tr>
<tr><td>Metrichor</td><td>Heuristic with phasing</td><td>0.885</td><td>0.137</td><td>0.091</td><td>QUAL > 1 & ((PE1 + 0.5 * QD) > 2.25)</td></tr>
<tr><td>Metrichor</td><td>Heuristic with phasing</td><td>0.887</td><td>0.13</td><td>0.095</td><td>QUAL > 1 & ((PE1 + 0.5 * QD) > 2.5)</td></tr>
<tr><td>Metrichor</td><td>Heuristic with phasing</td><td>0.851</td><td>0.196</td><td>0.097</td><td>QUAL > 1 & (max(SRP, SAP) < 30)</td></tr>
<tr><td>Metrichor</td><td>Heuristic with phasing</td><td>0.881</td><td>0.137</td><td>0.101</td><td>QUAL > 1 & (max(SRP, SAP) < 30) & ((PE1 + 0.5 * QD) > 2)</td></tr>
<tr><td>Metrichor</td><td>Heuristic with phasing</td><td>0.884</td><td>0.128</td><td>0.104</td><td>QUAL > 1 & (max(SRP, SAP) < 30) & ((PE1 + 0.5 * QD) > 2.25)</td></tr>
<tr><td>Metrichor</td><td>Heuristic with phasing</td><td>0.885</td><td>0.121</td><td>0.108</td><td>QUAL > 1 & (max(SRP, SAP) < 30) & ((PE1 + 0.5 * QD) > 2.5)</td></tr>
<tr><td>Metrichor</td><td>Heuristic with phasing</td><td>0.847</td><td>0.206</td><td>0.093</td><td>QUAL > 1 & (0 < SB1&2 < 1)</td></tr>
<tr><td>Metrichor</td><td>Heuristic with phasing</td><td>0.889</td><td>0.125</td><td>0.097</td><td>QUAL > 1 & (0 < SB1&2 < 1) & ((PE1 + 0.5 * QD) > 2)</td></tr>
<tr><td>Metrichor</td><td>Heuristic with phasing</td><td>0.892</td><td>0.115</td><td>0.1</td><td>QUAL > 1 & (0 < SB1&2 < 1) & ((PE1 + 0.5 * QD) > 2.25)</td></tr>
<tr><td>Metrichor</td><td>Heuristic with phasing</td><td>0.893</td><td>0.108</td><td>0.105</td><td>QUAL > 1 & (0 < SB1&2 < 1) & ((PE1 + 0.5 * QD) > 2.5)</td></tr>
<tr><td>Metrichor</td><td>Heuristic with phasing</td><td>0.858</td><td>0.174</td><td>0.107</td><td>QUAL > 1 & (0 < SB1&2 < 1) & (max(SRP, SAP) < 30)</td></tr>
<tr><td>Metrichor</td><td>Heuristic with phasing</td><td>0.887</td><td>0.116</td><td>0.11</td><td>QUAL > 1 & (0 < SB1&2 < 1) & (max(SRP, SAP) < 30) & ((PE1 + 0.5 * QD) > 2)</td></tr>
<tr><td>Metrichor</td><td>Heuristic with phasing</td><td>0.89</td><td>0.107</td><td>0.113</td><td>QUAL > 1 & (0 < SB1&2 < 1) & (max(SRP, SAP) < 30) & ((PE1 + 0.5 * QD) > 2.25)</td></tr>
<tr><td>Metrichor</td><td>Heuristic with phasing</td><td>0.891</td><td>0.1</td><td>0.118</td><td>QUAL > 1 & (0 < SB1&2 < 1) & (max(SRP, SAP) < 30) & ((PE1 + 0.5 * QD) > 2.5)</td></tr>
<tr><td>Metrichor</td><td>Heuristic with phasing</td><td>0.884</td><td>0.141</td><td>0.09</td><td>QUAL > 1 & GT != 0|0</td></tr>
<tr><td>Metrichor</td><td>Heuristic with phasing</td><td>0.898</td><td>0.111</td><td>0.092</td><td>QUAL > 1 & GT != 0|0 & ((PE1 + 0.5 * QD) > 2)</td></tr>
<tr><td>Metrichor</td><td>Heuristic with phasing</td><td>0.899</td><td>0.106</td><td>0.095</td><td>QUAL > 1 & GT != 0|0 & ((PE1 + 0.5 * QD) > 2.25)</td></tr>
<tr><td>Metrichor</td><td>Heuristic with phasing</td><td>0.899</td><td>0.102</td><td>0.099</td><td>QUAL > 1 & GT != 0|0 & ((PE1 + 0.5 * QD) > 2.5)</td></tr>
<tr><td>Metrichor</td><td>Heuristic with phasing</td><td>0.883</td><td>0.13</td><td>0.103</td><td>QUAL > 1 & GT != 0|0 & (max(SRP, SAP) < 30)</td></tr>
<tr><td>Metrichor</td><td>Heuristic with phasing</td><td>0.895</td><td>0.104</td><td>0.106</td><td>QUAL > 1 & GT != 0|0 & (max(SRP, SAP) < 30) & ((PE1 + 0.5 * QD) > 2)</td></tr>
<tr><td>Metrichor</td><td>Heuristic with phasing</td><td>0.896</td><td>0.099</td><td>0.108</td><td>QUAL > 1 & GT != 0|0 & (max(SRP, SAP) < 30) & ((PE1 + 0.5 * QD) > 2.25)</td></tr>
<tr><td>Metrichor</td><td>Heuristic with phasing</td><td>0.896</td><td>0.095</td><td>0.112</td><td>QUAL > 1 & GT != 0|0 & (max(SRP, SAP) < 30) & ((PE1 + 0.5 * QD) > 2.5)</td></tr>
<tr><td>Metrichor</td><td>Heuristic with phasing</td><td>0.893</td><td>0.115</td><td>0.099</td><td>QUAL > 1 & GT != 0|0 & (0 < SB1&2 < 1)</td></tr>
<tr><td>Metrichor</td><td>Heuristic with phasing</td><td>0.905</td><td>0.087</td><td>0.102</td><td>QUAL > 1 & GT != 0|0 & (0 < SB1&2 < 1) & ((PE1 + 0.5 * QD) > 2)</td></tr>
<tr><td>Metrichor</td><td>Heuristic with phasing</td><td>0.906</td><td>0.083</td><td>0.104</td><td>QUAL > 1 & GT != 0|0 & (0 < SB1&2 < 1) & ((PE1 + 0.5 * QD) > 2.25)</td></tr>
<tr><td>Metrichor</td><td>Heuristic with phasing</td><td>0.906</td><td>0.079</td><td>0.108</td><td>QUAL > 1 & GT != 0|0 & (0 < SB1&2 < 1) & ((PE1 + 0.5 * QD) > 2.5)</td></tr>
<tr><td>Metrichor</td><td>Heuristic with phasing</td><td>0.891</td><td>0.105</td><td>0.113</td><td>QUAL > 1 & GT != 0|0 & (0 < SB1&2 < 1) & (max(SRP, SAP) < 30)</td></tr>
<tr><td>Metrichor</td><td>Heuristic with phasing</td><td>0.902</td><td>0.081</td><td>0.115</td><td>QUAL > 1 & GT != 0|0 & (0 < SB1&2 < 1) & (max(SRP, SAP) < 30) & ((PE1 + 0.5 * QD) > 2)</td></tr>
<tr><td>Metrichor</td><td>Heuristic with phasing</td><td>0.903</td><td>0.077</td><td>0.117</td><td>QUAL > 1 & GT != 0|0 & (0 < SB1&2 < 1) & (max(SRP, SAP) < 30) & ((PE1 + 0.5 * QD) > 2.25)</td></tr>
<tr><td>Metrichor</td><td>Heuristic with phasing</td><td>0.902</td><td>0.073</td><td>0.121</td><td>QUAL > 1 & GT != 0|0 & (0 < SB1&2 < 1) & (max(SRP, SAP) < 30) & ((PE1 + 0.5 * QD) > 2.5)</td></tr>
<tr><td>Nanonet</td><td>Heuristic with phasing</td><td>0.641</td><td>0.498</td><td>0.113</td><td>QUAL > 1</td></tr>
<tr><td>Nanonet</td><td>Heuristic with phasing</td><td>0.8</td><td>0.264</td><td>0.125</td><td>QUAL > 1 & ((PE1 + 0.5 * QD) > 2)</td></tr>
<tr><td>Nanonet</td><td>Heuristic with phasing</td><td>0.814</td><td>0.23</td><td>0.136</td><td>QUAL > 1 & ((PE1 + 0.5 * QD) > 2.25)</td></tr>
<tr><td>Nanonet</td><td>Heuristic with phasing</td><td>0.821</td><td>0.208</td><td>0.148</td><td>QUAL > 1 & ((PE1 + 0.5 * QD) > 2.5)</td></tr>
<tr><td>Nanonet</td><td>Heuristic with phasing</td><td>0.75</td><td>0.344</td><td>0.125</td><td>QUAL > 1 & (max(SRP, SAP) < 30)</td></tr>
<tr><td>Nanonet</td><td>Heuristic with phasing</td><td>0.847</td><td>0.169</td><td>0.137</td><td>QUAL > 1 & (max(SRP, SAP) < 30) & ((PE1 + 0.5 * QD) > 2)</td></tr>
<tr><td>Nanonet</td><td>Heuristic with phasing</td><td>0.852</td><td>0.148</td><td>0.147</td><td>QUAL > 1 & (max(SRP, SAP) < 30) & ((PE1 + 0.5 * QD) > 2.25)</td></tr>
<tr><td>Nanonet</td><td>Heuristic with phasing</td><td>0.852</td><td>0.136</td><td>0.159</td><td>QUAL > 1 & (max(SRP, SAP) < 30) & ((PE1 + 0.5 * QD) > 2.5)</td></tr>
<tr><td>Nanonet</td><td>Heuristic with phasing</td><td>0.66</td><td>0.456</td><td>0.16</td><td>QUAL > 1 & (0 < SB1&2 < 1)</td></tr>
<tr><td>Nanonet</td><td>Heuristic with phasing</td><td>0.825</td><td>0.18</td><td>0.17</td><td>QUAL > 1 & (0 < SB1&2 < 1) & ((PE1 + 0.5 * QD) > 2)</td></tr>
<tr><td>Nanonet</td><td>Heuristic with phasing</td><td>0.837</td><td>0.147</td><td>0.179</td><td>QUAL > 1 & (0 < SB1&2 < 1) & ((PE1 + 0.5 * QD) > 2.25)</td></tr>
<tr><td>Nanonet</td><td>Heuristic with phasing</td><td>0.84</td><td>0.127</td><td>0.19</td><td>QUAL > 1 & (0 < SB1&2 < 1) & ((PE1 + 0.5 * QD) > 2.5)</td></tr>
<tr><td>Nanonet</td><td>Heuristic with phasing</td><td>0.759</td><td>0.3</td><td>0.17</td><td>QUAL > 1 & (0 < SB1&2 < 1) & (max(SRP, SAP) < 30)</td></tr>
<tr><td>Nanonet</td><td>Heuristic with phasing</td><td>0.852</td><td>0.114</td><td>0.18</td><td>QUAL > 1 & (0 < SB1&2 < 1) & (max(SRP, SAP) < 30) & ((PE1 + 0.5 * QD) > 2)</td></tr>
<tr><td>Nanonet</td><td>Heuristic with phasing</td><td>0.855</td><td>0.095</td><td>0.189</td><td>QUAL > 1 & (0 < SB1&2 < 1) & (max(SRP, SAP) < 30) & ((PE1 + 0.5 * QD) > 2.25)</td></tr>
<tr><td>Nanonet</td><td>Heuristic with phasing</td><td>0.854</td><td>0.084</td><td>0.2</td><td>QUAL > 1 & (0 < SB1&2 < 1) & (max(SRP, SAP) < 30) & ((PE1 + 0.5 * QD) > 2.5)</td></tr>
</table>

Supplementary Table 6: Comparison of base callers with phasing / Part 3 of 5

<table>
<tr><th>Basecaller</th><th>Filtration Approach</th><th>F1 score</th><th>FDR</th><th>FNR</th><th>Condition</th></tr>
<tr><td>Nanonet</td><td>Heuristic with phasing</td><td>0.755</td><td>0.336</td><td>0.125</td><td>QUAL > 1 & GT != 0|0</td></tr>
<tr><td>Nanonet</td><td>Heuristic with phasing</td><td>0.807</td><td>0.248</td><td>0.13</td><td>QUAL > 1 & GT != 0|0 & ((PE1 + 0.5 * QD) > 2)</td></tr>
<tr><td>Nanonet</td><td>Heuristic with phasing</td><td>0.819</td><td>0.219</td><td>0.139</td><td>QUAL > 1 & GT != 0|0 & ((PE1 + 0.5 * QD) > 2.25)</td></tr>
<tr><td>Nanonet</td><td>Heuristic with phasing</td><td>0.825</td><td>0.198</td><td>0.15</td><td>QUAL > 1 & GT != 0|0 & ((PE1 + 0.5 * QD) > 2.5)</td></tr>
<tr><td>Nanonet</td><td>Heuristic with phasing</td><td>0.823</td><td>0.214</td><td>0.137</td><td>QUAL > 1 & GT != 0|0 & (max(SRP, SAP) < 30)</td></tr>
<tr><td>Nanonet</td><td>Heuristic with phasing</td><td>0.852</td><td>0.154</td><td>0.141</td><td>QUAL > 1 & GT != 0|0 & (max(SRP, SAP) < 30) & ((PE1 + 0.5 * QD) > 2)</td></tr>
<tr><td>Nanonet</td><td>Heuristic with phasing</td><td>0.856</td><td>0.138</td><td>0.15</td><td>QUAL > 1 & GT != 0|0 & (max(SRP, SAP) < 30) & ((PE1 + 0.5 * QD) > 2.25)</td></tr>
<tr><td>Nanonet</td><td>Heuristic with phasing</td><td>0.856</td><td>0.127</td><td>0.161</td><td>QUAL > 1 & GT != 0|0 & (max(SRP, SAP) < 30) & ((PE1 + 0.5 * QD) > 2.5)</td></tr>
<tr><td>Nanonet</td><td>Heuristic with phasing</td><td>0.784</td><td>0.257</td><td>0.17</td><td>QUAL > 1 & GT != 0|0 & (0 < SB1&2 < 1)</td></tr>
<tr><td>Nanonet</td><td>Heuristic with phasing</td><td>0.831</td><td>0.163</td><td>0.174</td><td>QUAL > 1 & GT != 0|0 & (0 < SB1&2 < 1) & ((PE1 + 0.5 * QD) > 2)</td></tr>
<tr><td>Nanonet</td><td>Heuristic with phasing</td><td>0.841</td><td>0.136</td><td>0.181</td><td>QUAL > 1 & GT != 0|0 & (0 < SB1&2 < 1) & ((PE1 + 0.5 * QD) > 2.25)</td></tr>
<tr><td>Nanonet</td><td>Heuristic with phasing</td><td>0.844</td><td>0.117</td><td>0.192</td><td>QUAL > 1 & GT != 0|0 & (0 < SB1&2 < 1) & ((PE1 + 0.5 * QD) > 2.5)</td></tr>
<tr><td>Nanonet</td><td>Heuristic with phasing</td><td>0.833</td><td>0.153</td><td>0.18</td><td>QUAL > 1 & GT != 0|0 & (0 < SB1&2 < 1) & (max(SRP, SAP) < 30)</td></tr>
<tr><td>Nanonet</td><td>Heuristic with phasing</td><td>0.856</td><td>0.1</td><td>0.184</td><td>QUAL > 1 & GT != 0|0 & (0 < SB1&2 < 1) & (max(SRP, SAP) < 30) & ((PE1 + 0.5 * QD) > 2)</td></tr>
<tr><td>Nanonet</td><td>Heuristic with phasing</td><td>0.859</td><td>0.085</td><td>0.191</td><td>QUAL > 1 & GT != 0|0 & (0 < SB1&2 < 1) & (max(SRP, SAP) < 30) & ((PE1 + 0.5 * QD) > 2.25)</td></tr>
<tr><td>Nanonet</td><td>Heuristic with phasing</td><td>0.857</td><td>0.076</td><td>0.201</td><td>QUAL > 1 & GT != 0|0 & (0 < SB1&2 < 1) & (max(SRP, SAP) < 30) & ((PE1 + 0.5 * QD) > 2.5)</td></tr>
<tr><td>Scrappie</td><td>Heuristic with phasing</td><td>0.528</td><td>0.629</td><td>0.083</td><td>QUAL > 1</td></tr>
<tr><td>Scrappie</td><td>Heuristic with phasing</td><td>0.529</td><td>0.628</td><td>0.084</td><td>QUAL > 1 & ((PE1 + 0.5 * QD) > 2)</td></tr>
<tr><td>Scrappie</td><td>Heuristic with phasing</td><td>0.529</td><td>0.628</td><td>0.084</td><td>QUAL > 1 & ((PE1 + 0.5 * QD) > 2.25)</td></tr>
<tr><td>Scrappie</td><td>Heuristic with phasing</td><td>0.53</td><td>0.627</td><td>0.084</td><td>QUAL > 1 & ((PE1 + 0.5 * QD) > 2.5)</td></tr>
<tr><td>Scrappie</td><td>Heuristic with phasing</td><td>0.582</td><td>0.571</td><td>0.094</td><td>QUAL > 1 & (max(SRP, SAP) < 30)</td></tr>
<tr><td>Scrappie</td><td>Heuristic with phasing</td><td>0.582</td><td>0.571</td><td>0.094</td><td>QUAL > 1 & (max(SRP, SAP) < 30) & ((PE1 + 0.5 * QD) > 2)</td></tr>
<tr><td>Scrappie</td><td>Heuristic with phasing</td><td>0.582</td><td>0.571</td><td>0.094</td><td>QUAL > 1 & (max(SRP, SAP) < 30) & ((PE1 + 0.5 * QD) > 2.25)</td></tr>
<tr><td>Scrappie</td><td>Heuristic with phasing</td><td>0.583</td><td>0.57</td><td>0.095</td><td>QUAL > 1 & (max(SRP, SAP) < 30) & ((PE1 + 0.5 * QD) > 2.5)</td></tr>
<tr><td>Scrappie</td><td>Heuristic with phasing</td><td>0.528</td><td>0.628</td><td>0.092</td><td>QUAL > 1 & (0 < SB1&2 < 1)</td></tr>
<tr><td>Scrappie</td><td>Heuristic with phasing</td><td>0.529</td><td>0.627</td><td>0.092</td><td>QUAL > 1 & (0 < SB1&2 < 1) & ((PE1 + 0.5 * QD) > 2)</td></tr>
<tr><td>Scrappie</td><td>Heuristic with phasing</td><td>0.53</td><td>0.626</td><td>0.093</td><td>QUAL > 1 & (0 < SB1&2 < 1) & ((PE1 + 0.5 * QD) > 2.25)</td></tr>
<tr><td>Scrappie</td><td>Heuristic with phasing</td><td>0.53</td><td>0.626</td><td>0.093</td><td>QUAL > 1 & (0 < SB1&2 < 1) & ((PE1 + 0.5 * QD) > 2.5)</td></tr>
<tr><td>Scrappie</td><td>Heuristic with phasing</td><td>0.582</td><td>0.57</td><td>0.102</td><td>QUAL > 1 & (0 < SB1&2 < 1) & (max(SRP, SAP) < 30)</td></tr>
<tr><td>Scrappie</td><td>Heuristic with phasing</td><td>0.581</td><td>0.57</td><td>0.103</td><td>QUAL > 1 & (0 < SB1&2 < 1) & (max(SRP, SAP) < 30) & ((PE1 + 0.5 * QD) > 2)</td></tr>
<tr><td>Scrappie</td><td>Heuristic with phasing</td><td>0.582</td><td>0.569</td><td>0.103</td><td>QUAL > 1 & (0 < SB1&2 < 1) & (max(SRP, SAP) < 30) & ((PE1 + 0.5 * QD) > 2.25)</td></tr>
<tr><td>Scrappie</td><td>Heuristic with phasing</td><td>0.582</td><td>0.569</td><td>0.103</td><td>QUAL > 1 & (0 < SB1&2 < 1) & (max(SRP, SAP) < 30) & ((PE1 + 0.5 * QD) > 2.5)</td></tr>
<tr><td>Scrappie</td><td>Heuristic with phasing</td><td>0.868</td><td>0.144</td><td>0.119</td><td>QUAL > 1 & GT != 0|0</td></tr>
<tr><td>Scrappie</td><td>Heuristic with phasing</td><td>0.869</td><td>0.141</td><td>0.12</td><td>QUAL > 1 & GT != 0|0 & ((PE1 + 0.5 * QD) > 2)</td></tr>
<tr><td>Scrappie</td><td>Heuristic with phasing</td><td>0.87</td><td>0.139</td><td>0.12</td><td>QUAL > 1 & GT != 0|0 & ((PE1 + 0.5 * QD) > 2.25)</td></tr>
<tr><td>Scrappie</td><td>Heuristic with phasing</td><td>0.871</td><td>0.137</td><td>0.12</td><td>QUAL > 1 & GT != 0|0 & ((PE1 + 0.5 * QD) > 2.5)</td></tr>
<tr><td>Scrappie</td><td>Heuristic with phasing</td><td>0.883</td><td>0.104</td><td>0.129</td><td>QUAL > 1 & GT != 0|0 & (max(SRP, SAP) < 30)</td></tr>
<tr><td>Scrappie</td><td>Heuristic with phasing</td><td>0.884</td><td>0.102</td><td>0.129</td><td>QUAL > 1 & GT != 0|0 & (max(SRP, SAP) < 30) & ((PE1 + 0.5 * QD) > 2)</td></tr>
<tr><td>Scrappie</td><td>Heuristic with phasing</td><td>0.885</td><td>0.101</td><td>0.129</td><td>QUAL > 1 & GT != 0|0 & (max(SRP, SAP) < 30) & ((PE1 + 0.5 * QD) > 2.25)</td></tr>
<tr><td>Scrappie</td><td>Heuristic with phasing</td><td>0.885</td><td>0.1</td><td>0.13</td><td>QUAL > 1 & GT != 0|0 & (max(SRP, SAP) < 30) & ((PE1 + 0.5 * QD) > 2.5)</td></tr>
<tr><td>Scrappie</td><td>Heuristic with phasing</td><td>0.868</td><td>0.135</td><td>0.128</td><td>QUAL > 1 & GT != 0|0 & (0 < SB1&2 < 1)</td></tr>
<tr><td>Scrappie</td><td>Heuristic with phasing</td><td>0.87</td><td>0.132</td><td>0.128</td><td>QUAL > 1 & GT != 0|0 & (0 < SB1&2 < 1) & ((PE1 + 0.5 * QD) > 2)</td></tr>
<tr><td>Scrappie</td><td>Heuristic with phasing</td><td>0.871</td><td>0.13</td><td>0.128</td><td>QUAL > 1 & GT != 0|0 & (0 < SB1&2 < 1) & ((PE1 + 0.5 * QD) > 2.25)</td></tr>
<tr><td>Scrappie</td><td>Heuristic with phasing</td><td>0.872</td><td>0.128</td><td>0.128</td><td>QUAL > 1 & GT != 0|0 & (0 < SB1&2 < 1) & ((PE1 + 0.5 * QD) > 2.5)</td></tr>
<tr><td>Scrappie</td><td>Heuristic with phasing</td><td>0.882</td><td>0.098</td><td>0.137</td><td>QUAL > 1 & GT != 0|0 & (0 < SB1&2 < 1) & (max(SRP, SAP) < 30)</td></tr>
<tr><td>Scrappie</td><td>Heuristic with phasing</td><td>0.883</td><td>0.097</td><td>0.137</td><td>QUAL > 1 & GT != 0|0 & (0 < SB1&2 < 1) & (max(SRP, SAP) < 30) & ((PE1 + 0.5 * QD) > 2)</td></tr>
<tr><td>Scrappie</td><td>Heuristic with phasing</td><td>0.883</td><td>0.096</td><td>0.138</td><td>QUAL > 1 & GT != 0|0 & (0 < SB1&2 < 1) & (max(SRP, SAP) < 30) & ((PE1 + 0.5 * QD) > 2.25)</td></tr>
<tr><td>Scrappie</td><td>Heuristic with phasing</td><td>0.883</td><td>0.094</td><td>0.138</td><td>QUAL > 1 & GT != 0|0 & (0 < SB1&2 < 1) & (max(SRP, SAP) < 30) & ((PE1 + 0.5 * QD) > 2.5)</td></tr>
<tr><td>Albacore</td><td>Heuristic with phasing</td><td>0.843</td><td>0.227</td><td>0.073</td><td>QUAL > 20</td></tr>
<tr><td>Albacore</td><td>Heuristic with phasing</td><td>0.871</td><td>0.178</td><td>0.074</td><td>QUAL > 20 & ((PE1 + 0.5 * QD) > 2)</td></tr>
<tr><td>Albacore</td><td>Heuristic with phasing</td><td>0.878</td><td>0.164</td><td>0.076</td><td>QUAL > 20 & ((PE1 + 0.5 * QD) > 2.25)</td></tr>
<tr><td>Albacore</td><td>Heuristic with phasing</td><td>0.881</td><td>0.154</td><td>0.08</td><td>QUAL > 20 & ((PE1 + 0.5 * QD) > 2.5)</td></tr>
<tr><td>Albacore</td><td>Heuristic with phasing</td><td>0.871</td><td>0.17</td><td>0.084</td><td>QUAL > 20 & (max(SRP, SAP) < 30)</td></tr>
<tr><td>Albacore</td><td>Heuristic with phasing</td><td>0.88</td><td>0.153</td><td>0.085</td><td>QUAL > 20 & (max(SRP, SAP) < 30) & ((PE1 + 0.5 * QD) > 2)</td></tr>
<tr><td>Albacore</td><td>Heuristic with phasing</td><td>0.883</td><td>0.145</td><td>0.087</td><td>QUAL > 20 & (max(SRP, SAP) < 30) & ((PE1 + 0.5 * QD) > 2.25)</td></tr>
<tr><td>Albacore</td><td>Heuristic with phasing</td><td>0.886</td><td>0.137</td><td>0.09</td><td>QUAL > 20 & (max(SRP, SAP) < 30) & ((PE1 + 0.5 * QD) > 2.5)</td></tr>
<tr><td>Albacore</td><td>Heuristic with phasing</td><td>0.842</td><td>0.221</td><td>0.085</td><td>QUAL > 20 & (0 < SB1&2 < 1)</td></tr>
<tr><td>Albacore</td><td>Heuristic with phasing</td><td>0.869</td><td>0.171</td><td>0.086</td><td>QUAL > 20 & (0 < SB1&2 < 1) & ((PE1 + 0.5 * QD) > 2)</td></tr>
<tr><td>Albacore</td><td>Heuristic with phasing</td><td>0.876</td><td>0.157</td><td>0.088</td><td>QUAL > 20 & (0 < SB1&2 < 1) & ((PE1 + 0.5 * QD) > 2.25)</td></tr>
<tr><td>Albacore</td><td>Heuristic with phasing</td><td>0.88</td><td>0.146</td><td>0.092</td><td>QUAL > 20 & (0 < SB1&2 < 1) & ((PE1 + 0.5 * QD) > 2.5)</td></tr>
<tr><td>Albacore</td><td>Heuristic with phasing</td><td>0.868</td><td>0.166</td><td>0.095</td><td>QUAL > 20 & (0 < SB1&2 < 1) & (max(SRP, SAP) < 30)</td></tr>
<tr><td>Albacore</td><td>Heuristic with phasing</td><td>0.876</td><td>0.149</td><td>0.097</td><td>QUAL > 20 & (0 < SB1&2 < 1) & (max(SRP, SAP) < 30) & ((PE1 + 0.5 * QD) > 2)</td></tr>
<tr><td>Albacore</td><td>Heuristic with phasing</td><td>0.879</td><td>0.142</td><td>0.098</td><td>QUAL > 20 & (0 < SB1&2 < 1) & (max(SRP, SAP) < 30) & ((PE1 + 0.5 * QD) > 2.25)</td></tr>
<tr><td>Albacore</td><td>Heuristic with phasing</td><td>0.882</td><td>0.134</td><td>0.102</td><td>QUAL > 20 & (0 < SB1&2 < 1) & (max(SRP, SAP) < 30) & ((PE1 + 0.5 * QD) > 2.5)</td></tr>
<tr><td>Albacore</td><td>Heuristic with phasing</td><td>0.913</td><td>0.094</td><td>0.08</td><td>QUAL > 20 & GT != 0|0</td></tr>
<tr><td>Albacore</td><td>Heuristic with phasing</td><td>0.919</td><td>0.081</td><td>0.081</td><td>QUAL > 20 & GT != 0|0 & ((PE1 + 0.5 * QD) > 2)</td></tr>
<tr><td>Albacore</td><td>Heuristic with phasing</td><td>0.921</td><td>0.076</td><td>0.082</td><td>QUAL > 20 & GT != 0|0 & ((PE1 + 0.5 * QD) > 2.25)</td></tr>
<tr><td>Albacore</td><td>Heuristic with phasing</td><td>0.922</td><td>0.071</td><td>0.085</td><td>QUAL > 20 & GT != 0|0 & ((PE1 + 0.5 * QD) > 2.5)</td></tr>
<tr><td>Albacore</td><td>Heuristic with phasing</td><td>0.923</td><td>0.062</td><td>0.091</td><td>QUAL > 20 & GT != 0|0 & (max(SRP, SAP) < 30)</td></tr>
<tr><td>Albacore</td><td>Heuristic with phasing</td><td>0.926</td><td>0.057</td><td>0.091</td><td>QUAL > 20 & GT != 0|0 & (max(SRP, SAP) < 30) & ((PE1 + 0.5 * QD) > 2)</td></tr>
<tr><td>Albacore</td><td>Heuristic with phasing</td><td>0.926</td><td>0.055</td><td>0.092</td><td>QUAL > 20 & GT != 0|0 & (max(SRP, SAP) < 30) & ((PE1 + 0.5 * QD) > 2.25)</td></tr>
<tr><td>Albacore</td><td>Heuristic with phasing</td><td>0.926</td><td>0.053</td><td>0.095</td><td>QUAL > 20 & GT != 0|0 & (max(SRP, SAP) < 30) & ((PE1 + 0.5 * QD) > 2.5)</td></tr>
<tr><td>Albacore</td><td>Heuristic with phasing</td><td>0.911</td><td>0.085</td><td>0.092</td><td>QUAL > 20 & GT != 0|0 & (0 < SB1&2 < 1)</td></tr>
<tr><td>Albacore</td><td>Heuristic with phasing</td><td>0.917</td><td>0.072</td><td>0.093</td><td>QUAL > 20 & GT != 0|0 & (0 < SB1&2 < 1) & ((PE1 + 0.5 * QD) > 2)</td></tr>
<tr><td>Albacore</td><td>Heuristic with phasing</td><td>0.919</td><td>0.067</td><td>0.094</td><td>QUAL > 20 & GT != 0|0 & (0 < SB1&2 < 1) & ((PE1 + 0.5 * QD) > 2.25)</td></tr>
<tr><td>Albacore</td><td>Heuristic with phasing</td><td>0.92</td><td>0.063</td><td>0.097</td><td>QUAL > 20 & GT != 0|0 & (0 < SB1&2 < 1) & ((PE1 + 0.5 * QD) > 2.5)</td></tr>
<tr><td>Albacore</td><td>Heuristic with phasing</td><td>0.92</td><td>0.057</td><td>0.102</td><td>QUAL > 20 & GT != 0|0 & (0 < SB1&2 < 1) & (max(SRP, SAP) < 30)</td></tr>
<tr><td>Albacore</td><td>Heuristic with phasing</td><td>0.922</td><td>0.052</td><td>0.103</td><td>QUAL > 20 & GT != 0|0 & (0 < SB1&2 < 1) & (max(SRP, SAP) < 30) & ((PE1 + 0.5 * QD) > 2)</td></tr>
<tr><td>Albacore</td><td>Heuristic with phasing</td><td>0.922</td><td>0.051</td><td>0.104</td><td>QUAL > 20 & GT != 0|0 & (0 < SB1&2 < 1) & (max(SRP, SAP) < 30) & ((PE1 + 0.5 * QD) > 2.25)</td></tr>
<tr><td>Albacore</td><td>Heuristic with phasing</td><td>0.922</td><td>0.049</td><td>0.106</td><td>QUAL > 20 & GT != 0|0 & (0 < SB1&2 < 1) & (max(SRP, SAP) < 30) & ((PE1 + 0.5 * QD) > 2.5)</td></tr>
<tr><td>Metrichor</td><td>Heuristic with phasing</td><td>0.883</td><td>0.107</td><td>0.126</td><td>QUAL > 20</td></tr>
<tr><td>Metrichor</td><td>Heuristic with phasing</td><td>0.891</td><td>0.088</td><td>0.129</td><td>QUAL > 20 & ((PE1 + 0.5 * QD) > 2)</td></tr>
<tr><td>Metrichor</td><td>Heuristic with phasing</td><td>0.893</td><td>0.081</td><td>0.131</td><td>QUAL > 20 & ((PE1 + 0.5 * QD) > 2.25)</td></tr>
<tr><td>Metrichor</td><td>Heuristic with phasing</td><td>0.894</td><td>0.076</td><td>0.135</td><td>QUAL > 20 & ((PE1 + 0.5 * QD) > 2.5)</td></tr>
<tr><td>Metrichor</td><td>Heuristic with phasing</td><td>0.882</td><td>0.094</td><td>0.14</td><td>QUAL > 20 & (max(SRP, SAP) < 30)</td></tr>
<tr><td>Metrichor</td><td>Heuristic with phasing</td><td>0.887</td><td>0.082</td><td>0.142</td><td>QUAL > 20 & (max(SRP, SAP) < 30) & ((PE1 + 0.5 * QD) > 2)</td></tr>
<tr><td>Metrichor</td><td>Heuristic with phasing</td><td>0.888</td><td>0.076</td><td>0.145</td><td>QUAL > 20 & (max(SRP, SAP) < 30) & ((PE1 + 0.5 * QD) > 2.25)</td></tr>
<tr><td>Metrichor</td><td>Heuristic with phasing</td><td>0.888</td><td>0.072</td><td>0.148</td><td>QUAL > 20 & (max(SRP, SAP) < 30) & ((PE1 + 0.5 * QD) > 2.5)</td></tr>
<tr><td>Metrichor</td><td>Heuristic with phasing</td><td>0.884</td><td>0.099</td><td>0.133</td><td>QUAL > 20 & (0 < SB1&2 < 1)</td></tr>
<tr><td>Metrichor</td><td>Heuristic with phasing</td><td>0.892</td><td>0.079</td><td>0.136</td><td>QUAL > 20 & (0 < SB1&2 < 1) & ((PE1 + 0.5 * QD) > 2)</td></tr>
<tr><td>Metrichor</td><td>Heuristic with phasing</td><td>0.893</td><td>0.073</td><td>0.138</td><td>QUAL > 20 & (0 < SB1&2 < 1) & ((PE1 + 0.5 * QD) > 2.25)</td></tr>
<tr><td>Metrichor</td><td>Heuristic with phasing</td><td>0.894</td><td>0.067</td><td>0.142</td><td>QUAL > 20 & (0 < SB1&2 < 1) & ((PE1 + 0.5 * QD) > 2.5)</td></tr>
<tr><td>Metrichor</td><td>Heuristic with phasing</td><td>0.883</td><td>0.086</td><td>0.146</td><td>QUAL > 20 & (0 < SB1&2 < 1) & (max(SRP, SAP) < 30)</td></tr>
<tr><td>Metrichor</td><td>Heuristic with phasing</td><td>0.887</td><td>0.074</td><td>0.149</td><td>QUAL > 20 & (0 < SB1&2 < 1) & (max(SRP, SAP) < 30) & ((PE1 + 0.5 * QD) > 2)</td></tr>
<tr><td>Metrichor</td><td>Heuristic with phasing</td><td>0.888</td><td>0.069</td><td>0.151</td><td>QUAL > 20 & (0 < SB1&2 < 1) & (max(SRP, SAP) < 30) & ((PE1 + 0.5 * QD) > 2.25)</td></tr>
<tr><td>Metrichor</td><td>Heuristic with phasing</td><td>0.888</td><td>0.064</td><td>0.155</td><td>QUAL > 20 & (0 < SB1&2 < 1) & (max(SRP, SAP) < 30) & ((PE1 + 0.5 * QD) > 2.5)</td></tr>
<tr><td>Metrichor</td><td>Heuristic with phasing</td><td>0.896</td><td>0.075</td><td>0.131</td><td>QUAL > 20 & GT != 0|0</td></tr>
<tr><td>Metrichor</td><td>Heuristic with phasing</td><td>0.898</td><td>0.069</td><td>0.132</td><td>QUAL > 20 & GT != 0|0 & ((PE1 + 0.5 * QD) > 2)</td></tr>
<tr><td>Metrichor</td><td>Heuristic with phasing</td><td>0.899</td><td>0.065</td><td>0.134</td><td>QUAL > 20 & GT != 0|0 & ((PE1 + 0.5 * QD) > 2.25)</td></tr>
<tr><td>Metrichor</td><td>Heuristic with phasing</td><td>0.898</td><td>0.062</td><td>0.138</td><td>QUAL > 20 & GT != 0|0 & ((PE1 + 0.5 * QD) > 2.5)</td></tr>
<tr><td>Metrichor</td><td>Heuristic with phasing</td><td>0.892</td><td>0.068</td><td>0.144</td><td>QUAL > 20 & GT != 0|0 & (max(SRP, SAP) < 30)</td></tr>
<tr><td>Metrichor</td><td>Heuristic with phasing</td><td>0.893</td><td>0.064</td><td>0.146</td><td>QUAL > 20 & GT != 0|0 & (max(SRP, SAP) < 30) & ((PE1 + 0.5 * QD) > 2)</td></tr>
<tr><td>Metrichor</td><td>Heuristic with phasing</td><td>0.894</td><td>0.061</td><td>0.147</td><td>QUAL > 20 & GT != 0|0 & (max(SRP, SAP) < 30) & ((PE1 + 0.5 * QD) > 2.25)</td></tr>
<tr><td>Metrichor</td><td>Heuristic with phasing</td><td>0.893</td><td>0.058</td><td>0.151</td><td>QUAL > 20 & GT != 0|0 & (max(SRP, SAP) < 30) & ((PE1 + 0.5 * QD) > 2.5)</td></tr>
</table>

Supplementary Table 6: Comparison of base callers with phasing / Part 4 of 5

<table>
<tr><th>Basecaller</th><th>Filtration Approach</th><th>F1 score</th><th>FDR</th><th>FNR</th><th>Condition</th></tr>
<tr><td>Metrichor</td><td>Heuristic with phasing</td><td>0.897</td><td>0.066</td><td>0.138</td><td>QUAL > 20 & GT != 0|0 & (0 < SB1&2 < 1)</td></tr>
<tr><td>Metrichor</td><td>Heuristic with phasing</td><td>0.899</td><td>0.06</td><td>0.139</td><td>QUAL > 20 & GT != 0|0 & (0 < SB1&2 < 1) & ((PE1 + 0.5 * QD) > 2)</td></tr>
<tr><td>Metrichor</td><td>Heuristic with phasing</td><td>0.899</td><td>0.056</td><td>0.141</td><td>QUAL > 20 & GT != 0|0 & (0 < SB1&2 < 1) & ((PE1 + 0.5 * QD) > 2.25)</td></tr>
<tr><td>Metrichor</td><td>Heuristic with phasing</td><td>0.899</td><td>0.053</td><td>0.145</td><td>QUAL > 20 & GT != 0|0 & (0 < SB1&2 < 1) & ((PE1 + 0.5 * QD) > 2.5)</td></tr>
<tr><td>Metrichor</td><td>Heuristic with phasing</td><td>0.892</td><td>0.06</td><td>0.151</td><td>QUAL > 20 & GT != 0|0 & (0 < SB1&2 < 1) & (max(SRP, SAP) < 30)</td></tr>
<tr><td>Metrichor</td><td>Heuristic with phasing</td><td>0.893</td><td>0.056</td><td>0.152</td><td>QUAL > 20 & GT != 0|0 & (0 < SB1&2 < 1) & (max(SRP, SAP) < 30) & ((PE1 + 0.5 * QD) > 2)</td></tr>
<tr><td>Metrichor</td><td>Heuristic with phasing</td><td>0.894</td><td>0.053</td><td>0.154</td><td>QUAL > 20 & GT != 0|0 & (0 < SB1&2 < 1) & (max(SRP, SAP) < 30) & ((PE1 + 0.5 * QD) > 2.25)</td></tr>
<tr><td>Metrichor</td><td>Heuristic with phasing</td><td>0.893</td><td>0.05</td><td>0.157</td><td>QUAL > 20 & GT != 0|0 & (0 < SB1&2 < 1) & (max(SRP, SAP) < 30) & ((PE1 + 0.5 * QD) > 2.5)</td></tr>
<tr><td>Nanonet</td><td>Heuristic with phasing</td><td>0.74</td><td>0.34</td><td>0.159</td><td>QUAL > 20</td></tr>
<tr><td>Nanonet</td><td>Heuristic with phasing</td><td>0.823</td><td>0.19</td><td>0.163</td><td>QUAL > 20 & ((PE1 + 0.5 * QD) > 2)</td></tr>
<tr><td>Nanonet</td><td>Heuristic with phasing</td><td>0.834</td><td>0.163</td><td>0.168</td><td>QUAL > 20 & ((PE1 + 0.5 * QD) > 2.25)</td></tr>
<tr><td>Nanonet</td><td>Heuristic with phasing</td><td>0.838</td><td>0.146</td><td>0.177</td><td>QUAL > 20 & ((PE1 + 0.5 * QD) > 2.5)</td></tr>
<tr><td>Nanonet</td><td>Heuristic with phasing</td><td>0.804</td><td>0.22</td><td>0.171</td><td>QUAL > 20 & (max(SRP, SAP) < 30)</td></tr>
<tr><td>Nanonet</td><td>Heuristic with phasing</td><td>0.843</td><td>0.138</td><td>0.175</td><td>QUAL > 20 & (max(SRP, SAP) < 30) & ((PE1 + 0.5 * QD) > 2)</td></tr>
<tr><td>Nanonet</td><td>Heuristic with phasing</td><td>0.848</td><td>0.122</td><td>0.18</td><td>QUAL > 20 & (max(SRP, SAP) < 30) & ((PE1 + 0.5 * QD) > 2.25)</td></tr>
<tr><td>Nanonet</td><td>Heuristic with phasing</td><td>0.848</td><td>0.112</td><td>0.188</td><td>QUAL > 20 & (max(SRP, SAP) < 30) & ((PE1 + 0.5 * QD) > 2.5)</td></tr>
<tr><td>Nanonet</td><td>Heuristic with phasing</td><td>0.749</td><td>0.312</td><td>0.178</td><td>QUAL > 20 & (0 < SB1&2 < 1)</td></tr>
<tr><td>Nanonet</td><td>Heuristic with phasing</td><td>0.836</td><td>0.145</td><td>0.182</td><td>QUAL > 20 & (0 < SB1&2 < 1) & ((PE1 + 0.5 * QD) > 2)</td></tr>
<tr><td>Nanonet</td><td>Heuristic with phasing</td><td>0.847</td><td>0.117</td><td>0.187</td><td>QUAL > 20 & (0 < SB1&2 < 1) & ((PE1 + 0.5 * QD) > 2.25)</td></tr>
<tr><td>Nanonet</td><td>Heuristic with phasing</td><td>0.848</td><td>0.102</td><td>0.196</td><td>QUAL > 20 & (0 < SB1&2 < 1) & ((PE1 + 0.5 * QD) > 2.5)</td></tr>
<tr><td>Nanonet</td><td>Heuristic with phasing</td><td>0.81</td><td>0.191</td><td>0.189</td><td>QUAL > 20 & (0 < SB1&2 < 1) & (max(SRP, SAP) < 30)</td></tr>
<tr><td>Nanonet</td><td>Heuristic with phasing</td><td>0.85</td><td>0.102</td><td>0.193</td><td>QUAL > 20 & (0 < SB1&2 < 1) & (max(SRP, SAP) < 30) & ((PE1 + 0.5 * QD) > 2)</td></tr>
<tr><td>Nanonet</td><td>Heuristic with phasing</td><td>0.855</td><td>0.085</td><td>0.198</td><td>QUAL > 20 & (0 < SB1&2 < 1) & (max(SRP, SAP) < 30) & ((PE1 + 0.5 * QD) > 2.25)</td></tr>
<tr><td>Nanonet</td><td>Heuristic with phasing</td><td>0.855</td><td>0.075</td><td>0.206</td><td>QUAL > 20 & (0 < SB1&2 < 1) & (max(SRP, SAP) < 30) & ((PE1 + 0.5 * QD) > 2.5)</td></tr>
<tr><td>Nanonet</td><td>Heuristic with phasing</td><td>0.802</td><td>0.229</td><td>0.164</td><td>QUAL > 20 & GT != 0|0</td></tr>
<tr><td>Nanonet</td><td>Heuristic with phasing</td><td>0.827</td><td>0.179</td><td>0.166</td><td>QUAL > 20 & GT != 0|0 & ((PE1 + 0.5 * QD) > 2)</td></tr>
<tr><td>Nanonet</td><td>Heuristic with phasing</td><td>0.837</td><td>0.156</td><td>0.17</td><td>QUAL > 20 & GT != 0|0 & ((PE1 + 0.5 * QD) > 2.25)</td></tr>
<tr><td>Nanonet</td><td>Heuristic with phasing</td><td>0.84</td><td>0.141</td><td>0.178</td><td>QUAL > 20 & GT != 0|0 & ((PE1 + 0.5 * QD) > 2.5)</td></tr>
<tr><td>Nanonet</td><td>Heuristic with phasing</td><td>0.838</td><td>0.147</td><td>0.176</td><td>QUAL > 20 & GT != 0|0 & (max(SRP, SAP) < 30)</td></tr>
<tr><td>Nanonet</td><td>Heuristic with phasing</td><td>0.846</td><td>0.129</td><td>0.177</td><td>QUAL > 20 & GT != 0|0 & (max(SRP, SAP) < 30) & ((PE1 + 0.5 * QD) > 2)</td></tr>
<tr><td>Nanonet</td><td>Heuristic with phasing</td><td>0.85</td><td>0.116</td><td>0.181</td><td>QUAL > 20 & GT != 0|0 & (max(SRP, SAP) < 30) & ((PE1 + 0.5 * QD) > 2.25)</td></tr>
<tr><td>Nanonet</td><td>Heuristic with phasing</td><td>0.85</td><td>0.106</td><td>0.189</td><td>QUAL > 20 & GT != 0|0 & (max(SRP, SAP) < 30) & ((PE1 + 0.5 * QD) > 2.5)</td></tr>
<tr><td>Nanonet</td><td>Heuristic with phasing</td><td>0.814</td><td>0.188</td><td>0.183</td><td>QUAL > 20 & GT != 0|0 & (0 < SB1&2 < 1)</td></tr>
<tr><td>Nanonet</td><td>Heuristic with phasing</td><td>0.841</td><td>0.133</td><td>0.184</td><td>QUAL > 20 & GT != 0|0 & (0 < SB1&2 < 1) & ((PE1 + 0.5 * QD) > 2)</td></tr>
<tr><td>Nanonet</td><td>Heuristic with phasing</td><td>0.849</td><td>0.11</td><td>0.188</td><td>QUAL > 20 & GT != 0|0 & (0 < SB1&2 < 1) & ((PE1 + 0.5 * QD) > 2.25)</td></tr>
<tr><td>Nanonet</td><td>Heuristic with phasing</td><td>0.851</td><td>0.096</td><td>0.196</td><td>QUAL > 20 & GT != 0|0 & (0 < SB1&2 < 1) & ((PE1 + 0.5 * QD) > 2.5)</td></tr>
<tr><td>Nanonet</td><td>Heuristic with phasing</td><td>0.846</td><td>0.111</td><td>0.193</td><td>QUAL > 20 & GT != 0|0 & (0 < SB1&2 < 1) & (max(SRP, SAP) < 30)</td></tr>
<tr><td>Nanonet</td><td>Heuristic with phasing</td><td>0.853</td><td>0.092</td><td>0.195</td><td>QUAL > 20 & GT != 0|0 & (0 < SB1&2 < 1) & (max(SRP, SAP) < 30) & ((PE1 + 0.5 * QD) > 2)</td></tr>
<tr><td>Nanonet</td><td>Heuristic with phasing</td><td>0.857</td><td>0.079</td><td>0.199</td><td>QUAL > 20 & GT != 0|0 & (0 < SB1&2 < 1) & (max(SRP, SAP) < 30) & ((PE1 + 0.5 * QD) > 2.25)</td></tr>
<tr><td>Nanonet</td><td>Heuristic with phasing</td><td>0.857</td><td>0.07</td><td>0.206</td><td>QUAL > 20 & GT != 0|0 & (0 < SB1&2 < 1) & (max(SRP, SAP) < 30) & ((PE1 + 0.5 * QD) > 2.5)</td></tr>
<tr><td>Scrappie</td><td>Heuristic with phasing</td><td>0.727</td><td>0.359</td><td>0.161</td><td>QUAL > 20</td></tr>
<tr><td>Scrappie</td><td>Heuristic with phasing</td><td>0.727</td><td>0.358</td><td>0.161</td><td>QUAL > 20 & ((PE1 + 0.5 * QD) > 2)</td></tr>
<tr><td>Scrappie</td><td>Heuristic with phasing</td><td>0.728</td><td>0.357</td><td>0.161</td><td>QUAL > 20 & ((PE1 + 0.5 * QD) > 2.25)</td></tr>
<tr><td>Scrappie</td><td>Heuristic with phasing</td><td>0.729</td><td>0.356</td><td>0.161</td><td>QUAL > 20 & ((PE1 + 0.5 * QD) > 2.5)</td></tr>
<tr><td>Scrappie</td><td>Heuristic with phasing</td><td>0.746</td><td>0.322</td><td>0.17</td><td>QUAL > 20 & (max(SRP, SAP) < 30)</td></tr>
<tr><td>Scrappie</td><td>Heuristic with phasing</td><td>0.747</td><td>0.321</td><td>0.17</td><td>QUAL > 20 & (max(SRP, SAP) < 30) & ((PE1 + 0.5 * QD) > 2)</td></tr>
<tr><td>Scrappie</td><td>Heuristic with phasing</td><td>0.747</td><td>0.321</td><td>0.171</td><td>QUAL > 20 & (max(SRP, SAP) < 30) & ((PE1 + 0.5 * QD) > 2.25)</td></tr>
<tr><td>Scrappie</td><td>Heuristic with phasing</td><td>0.747</td><td>0.32</td><td>0.171</td><td>QUAL > 20 & (max(SRP, SAP) < 30) & ((PE1 + 0.5 * QD) > 2.5)</td></tr>
<tr><td>Scrappie</td><td>Heuristic with phasing</td><td>0.726</td><td>0.359</td><td>0.164</td><td>QUAL > 20 & (0 < SB1&2 < 1)</td></tr>
<tr><td>Scrappie</td><td>Heuristic with phasing</td><td>0.726</td><td>0.358</td><td>0.165</td><td>QUAL > 20 & (0 < SB1&2 < 1) & ((PE1 + 0.5 * QD) > 2)</td></tr>
<tr><td>Scrappie</td><td>Heuristic with phasing</td><td>0.727</td><td>0.357</td><td>0.165</td><td>QUAL > 20 & (0 < SB1&2 < 1) & ((PE1 + 0.5 * QD) > 2.25)</td></tr>
<tr><td>Scrappie</td><td>Heuristic with phasing</td><td>0.727</td><td>0.356</td><td>0.165</td><td>QUAL > 20 & (0 < SB1&2 < 1) & ((PE1 + 0.5 * QD) > 2.5)</td></tr>
<tr><td>Scrappie</td><td>Heuristic with phasing</td><td>0.745</td><td>0.322</td><td>0.174</td><td>QUAL > 20 & (0 < SB1&2 < 1) & (max(SRP, SAP) < 30)</td></tr>
<tr><td>Scrappie</td><td>Heuristic with phasing</td><td>0.745</td><td>0.321</td><td>0.174</td><td>QUAL > 20 & (0 < SB1&2 < 1) & (max(SRP, SAP) < 30) & ((PE1 + 0.5 * QD) > 2)</td></tr>
<tr><td>Scrappie</td><td>Heuristic with phasing</td><td>0.745</td><td>0.321</td><td>0.174</td><td>QUAL > 20 & (0 < SB1&2 < 1) & (max(SRP, SAP) < 30) & ((PE1 + 0.5 * QD) > 2.25)</td></tr>
<tr><td>Scrappie</td><td>Heuristic with phasing</td><td>0.746</td><td>0.32</td><td>0.174</td><td>QUAL > 20 & (0 < SB1&2 < 1) & (max(SRP, SAP) < 30) & ((PE1 + 0.5 * QD) > 2.5)</td></tr>
<tr><td>Scrappie</td><td>Heuristic with phasing</td><td>0.859</td><td>0.093</td><td>0.185</td><td>QUAL > 20 & GT != 0|0</td></tr>
<tr><td>Scrappie</td><td>Heuristic with phasing</td><td>0.859</td><td>0.091</td><td>0.185</td><td>QUAL > 20 & GT != 0|0 & ((PE1 + 0.5 * QD) > 2)</td></tr>
<tr><td>Scrappie</td><td>Heuristic with phasing</td><td>0.86</td><td>0.09</td><td>0.185</td><td>QUAL > 20 & GT != 0|0 & ((PE1 + 0.5 * QD) > 2.25)</td></tr>
<tr><td>Scrappie</td><td>Heuristic with phasing</td><td>0.861</td><td>0.088</td><td>0.185</td><td>QUAL > 20 & GT != 0|0 & ((PE1 + 0.5 * QD) > 2.5)</td></tr>
<tr><td>Scrappie</td><td>Heuristic with phasing</td><td>0.861</td><td>0.076</td><td>0.194</td><td>QUAL > 20 & GT != 0|0 & (max(SRP, SAP) < 30)</td></tr>
<tr><td>Scrappie</td><td>Heuristic with phasing</td><td>0.861</td><td>0.075</td><td>0.194</td><td>QUAL > 20 & GT != 0|0 & (max(SRP, SAP) < 30) & ((PE1 + 0.5 * QD) > 2)</td></tr>
<tr><td>Scrappie</td><td>Heuristic with phasing</td><td>0.862</td><td>0.074</td><td>0.194</td><td>QUAL > 20 & GT != 0|0 & (max(SRP, SAP) < 30) & ((PE1 + 0.5 * QD) > 2.25)</td></tr>
<tr><td>Scrappie</td><td>Heuristic with phasing</td><td>0.862</td><td>0.073</td><td>0.194</td><td>QUAL > 20 & GT != 0|0 & (max(SRP, SAP) < 30) & ((PE1 + 0.5 * QD) > 2.5)</td></tr>
<tr><td>Scrappie</td><td>Heuristic with phasing</td><td>0.857</td><td>0.092</td><td>0.189</td><td>QUAL > 20 & GT != 0|0 & (0 < SB1&2 < 1)</td></tr>
<tr><td>Scrappie</td><td>Heuristic with phasing</td><td>0.858</td><td>0.09</td><td>0.189</td><td>QUAL > 20 & GT != 0|0 & (0 < SB1&2 < 1) & ((PE1 + 0.5 * QD) > 2)</td></tr>
<tr><td>Scrappie</td><td>Heuristic with phasing</td><td>0.859</td><td>0.088</td><td>0.189</td><td>QUAL > 20 & GT != 0|0 & (0 < SB1&2 < 1) & ((PE1 + 0.5 * QD) > 2.25)</td></tr>
<tr><td>Scrappie</td><td>Heuristic with phasing</td><td>0.859</td><td>0.087</td><td>0.189</td><td>QUAL > 20 & GT != 0|0 & (0 < SB1&2 < 1) & ((PE1 + 0.5 * QD) > 2.5)</td></tr>
<tr><td>Scrappie</td><td>Heuristic with phasing</td><td>0.859</td><td>0.075</td><td>0.198</td><td>QUAL > 20 & GT != 0|0 & (0 < SB1&2 < 1) & (max(SRP, SAP) < 30)</td></tr>
<tr><td>Scrappie</td><td>Heuristic with phasing</td><td>0.86</td><td>0.074</td><td>0.198</td><td>QUAL > 20 & GT != 0|0 & (0 < SB1&2 < 1) & (max(SRP, SAP) < 30) & ((PE1 + 0.5 * QD) > 2)</td></tr>
<tr><td>Scrappie</td><td>Heuristic with phasing</td><td>0.86</td><td>0.073</td><td>0.198</td><td>QUAL > 20 & GT != 0|0 & (0 < SB1&2 < 1) & (max(SRP, SAP) < 30) & ((PE1 + 0.5 * QD) > 2.25)</td></tr>
<tr><td>Scrappie</td><td>Heuristic with phasing</td><td>0.86</td><td>0.072</td><td>0.198</td><td>QUAL > 20 & GT != 0|0 & (0 < SB1&2 < 1) & (max(SRP, SAP) < 30) & ((PE1 + 0.5 * QD) > 2.5)</td></tr>
<tr><td>Albacore</td><td>Heuristic with phasing</td><td>0.854</td><td>0.202</td><td>0.081</td><td>QUAL > 30</td></tr>
<tr><td>Albacore</td><td>Heuristic with phasing</td><td>0.875</td><td>0.164</td><td>0.082</td><td>QUAL > 30 & ((PE1 + 0.5 * QD) > 2)</td></tr>
<tr><td>Albacore</td><td>Heuristic with phasing</td><td>0.881</td><td>0.152</td><td>0.084</td><td>QUAL > 30 & ((PE1 + 0.5 * QD) > 2.25)</td></tr>
<tr><td>Albacore</td><td>Heuristic with phasing</td><td>0.884</td><td>0.143</td><td>0.087</td><td>QUAL > 30 & ((PE1 + 0.5 * QD) > 2.5)</td></tr>
<tr><td>Albacore</td><td>Heuristic with phasing</td><td>0.876</td><td>0.153</td><td>0.092</td><td>QUAL > 30 & (max(SRP, SAP) < 30)</td></tr>
<tr><td>Albacore</td><td>Heuristic with phasing</td><td>0.882</td><td>0.142</td><td>0.093</td><td>QUAL > 30 & (max(SRP, SAP) < 30) & ((PE1 + 0.5 * QD) > 2)</td></tr>
<tr><td>Albacore</td><td>Heuristic with phasing</td><td>0.885</td><td>0.136</td><td>0.094</td><td>QUAL > 30 & (max(SRP, SAP) < 30) & ((PE1 + 0.5 * QD) > 2.25)</td></tr>
<tr><td>Albacore</td><td>Heuristic with phasing</td><td>0.887</td><td>0.129</td><td>0.097</td><td>QUAL > 30 & (max(SRP, SAP) < 30) & ((PE1 + 0.5 * QD) > 2.5)</td></tr>
<tr><td>Albacore</td><td>Heuristic with phasing</td><td>0.853</td><td>0.197</td><td>0.09</td><td>QUAL > 30 & (0 < SB1&2 < 1)</td></tr>
<tr><td>Albacore</td><td>Heuristic with phasing</td><td>0.874</td><td>0.159</td><td>0.091</td><td>QUAL > 30 & (0 < SB1&2 < 1) & ((PE1 + 0.5 * QD) > 2)</td></tr>
<tr><td>Albacore</td><td>Heuristic with phasing</td><td>0.88</td><td>0.146</td><td>0.093</td><td>QUAL > 30 & (0 < SB1&2 < 1) & ((PE1 + 0.5 * QD) > 2.25)</td></tr>
<tr><td>Albacore</td><td>Heuristic with phasing</td><td>0.884</td><td>0.137</td><td>0.095</td><td>QUAL > 30 & (0 < SB1&2 < 1) & ((PE1 + 0.5 * QD) > 2.5)</td></tr>
<tr><td>Albacore</td><td>Heuristic with phasing</td><td>0.874</td><td>0.15</td><td>0.1</td><td>QUAL > 30 & (0 < SB1&2 < 1) & (max(SRP, SAP) < 30)</td></tr>
<tr><td>Albacore</td><td>Heuristic with phasing</td><td>0.88</td><td>0.139</td><td>0.101</td><td>QUAL > 30 & (0 < SB1&2 < 1) & (max(SRP, SAP) < 30) & ((PE1 + 0.5 * QD) > 2)</td></tr>
<tr><td>Albacore</td><td>Heuristic with phasing</td><td>0.882</td><td>0.133</td><td>0.103</td><td>QUAL > 30 & (0 < SB1&2 < 1) & (max(SRP, SAP) < 30) & ((PE1 + 0.5 * QD) > 2.25)</td></tr>
<tr><td>Albacore</td><td>Heuristic with phasing</td><td>0.884</td><td>0.126</td><td>0.105</td><td>QUAL > 30 & (0 < SB1&2 < 1) & (max(SRP, SAP) < 30) & ((PE1 + 0.5 * QD) > 2.5)</td></tr>
<tr><td>Albacore</td><td>Heuristic with phasing</td><td>0.915</td><td>0.083</td><td>0.087</td><td>QUAL > 30 & GT != 0|0</td></tr>
<tr><td>Albacore</td><td>Heuristic with phasing</td><td>0.919</td><td>0.073</td><td>0.088</td><td>QUAL > 30 & GT != 0|0 & ((PE1 + 0.5 * QD) > 2)</td></tr>
<tr><td>Albacore</td><td>Heuristic with phasing</td><td>0.921</td><td>0.068</td><td>0.089</td><td>QUAL > 30 & GT != 0|0 & ((PE1 + 0.5 * QD) > 2.25)</td></tr>
<tr><td>Albacore</td><td>Heuristic with phasing</td><td>0.922</td><td>0.065</td><td>0.091</td><td>QUAL > 30 & GT != 0|0 & ((PE1 + 0.5 * QD) > 2.5)</td></tr>
<tr><td>Albacore</td><td>Heuristic with phasing</td><td>0.922</td><td>0.057</td><td>0.098</td><td>QUAL > 30 & GT != 0|0 & (max(SRP, SAP) < 30)</td></tr>
<tr><td>Albacore</td><td>Heuristic with phasing</td><td>0.924</td><td>0.053</td><td>0.098</td><td>QUAL > 30 & GT != 0|0 & (max(SRP, SAP) < 30) & ((PE1 + 0.5 * QD) > 2)</td></tr>
<tr><td>Albacore</td><td>Heuristic with phasing</td><td>0.924</td><td>0.051</td><td>0.099</td><td>QUAL > 30 & GT != 0|0 & (max(SRP, SAP) < 30) & ((PE1 + 0.5 * QD) > 2.25)</td></tr>
<tr><td>Albacore</td><td>Heuristic with phasing</td><td>0.924</td><td>0.05</td><td>0.101</td><td>QUAL > 30 & GT != 0|0 & (max(SRP, SAP) < 30) & ((PE1 + 0.5 * QD) > 2.5)</td></tr>
<tr><td>Albacore</td><td>Heuristic with phasing</td><td>0.914</td><td>0.076</td><td>0.096</td><td>QUAL > 30 & GT != 0|0 & (0 < SB1&2 < 1)</td></tr>
<tr><td>Albacore</td><td>Heuristic with phasing</td><td>0.918</td><td>0.066</td><td>0.097</td><td>QUAL > 30 & GT != 0|0 & (0 < SB1&2 < 1) & ((PE1 + 0.5 * QD) > 2)</td></tr>
<tr><td>Albacore</td><td>Heuristic with phasing</td><td>0.92</td><td>0.061</td><td>0.098</td><td>QUAL > 30 & GT != 0|0 & (0 < SB1&2 < 1) & ((PE1 + 0.5 * QD) > 2.25)</td></tr>
<tr><td>Albacore</td><td>Heuristic with phasing</td><td>0.921</td><td>0.058</td><td>0.1</td><td>QUAL > 30 & GT != 0|0 & (0 < SB1&2 < 1) & ((PE1 + 0.5 * QD) > 2.5)</td></tr>
<tr><td>Albacore</td><td>Heuristic with phasing</td><td>0.92</td><td>0.053</td><td>0.106</td><td>QUAL > 30 & GT != 0|0 & (0 < SB1&2 < 1) & (max(SRP, SAP) < 30)</td></tr>
<tr><td>Albacore</td><td>Heuristic with phasing</td><td>0.921</td><td>0.049</td><td>0.107</td><td>QUAL > 30 & GT != 0|0 & (0 < SB1&2 < 1) & (max(SRP, SAP) < 30) & ((PE1 + 0.5 * QD) > 2)</td></tr>
<tr><td>Albacore</td><td>Heuristic with phasing</td><td>0.922</td><td>0.047</td><td>0.107</td><td>QUAL > 30 & GT != 0|0 & (0 < SB1&2 < 1) & (max(SRP, SAP) < 30) & ((PE1 + 0.5 * QD) > 2.25)</td></tr>
<tr><td>Albacore</td><td>Heuristic with phasing</td><td>0.921</td><td>0.046</td><td>0.11</td><td>QUAL > 30 & GT != 0|0 & (0 < SB1&2 < 1) & (max(SRP, SAP) < 30) & ((PE1 + 0.5 * QD) > 2.5)</td></tr>
</table>

Supplementary Table 6: Comparison of base callers with phasing / Part 5 of 5

Basecaller	Filtration Approach	F1 score	FDR	FNR	Condition
Metrichor	Heuristic with phasing	0.882	0.085	0.149	QUAL > 30
Metrichor	Heuristic with phasing	0.887	0.072	0.151	QUAL > 30 & ((PE1 + 0.5 * QD) > 2)
Metrichor	Heuristic with phasing	0.888	0.067	0.153	QUAL > 30 & ((PE1 + 0.5 * QD) > 2.25)
Metrichor	Heuristic with phasing	0.888	0.062	0.157	QUAL > 30 & ((PE1 + 0.5 * QD) > 2.5)
Metrichor	Heuristic with phasing	0.879	0.075	0.163	QUAL > 30 & (max(SRP, SAP) < 30)
Metrichor	Heuristic with phasing	0.881	0.068	0.165	QUAL > 30 & (max(SRP, SAP) < 30) & ((PE1 + 0.5 * QD) > 2)
Metrichor	Heuristic with phasing	0.882	0.063	0.167	QUAL > 30 & (max(SRP, SAP) < 30) & ((PE1 + 0.5 * QD) > 2.25)
Metrichor	Heuristic with phasing	0.882	0.059	0.17	QUAL > 30 & (max(SRP, SAP) < 30) & ((PE1 + 0.5 * QD) > 2.5)
Metrichor	Heuristic with phasing	0.881	0.079	0.156	QUAL > 30 & (0 < SB1&2 < 1)
Metrichor	Heuristic with phasing	0.885	0.067	0.159	QUAL > 30 & (0 < SB1&2 < 1) & ((PE1 + 0.5 * QD) > 2)
Metrichor	Heuristic with phasing	0.886	0.061	0.161	QUAL > 30 & (0 < SB1&2 < 1) & ((PE1 + 0.5 * QD) > 2.25)
Metrichor	Heuristic with phasing	0.886	0.057	0.164	QUAL > 30 & (0 < SB1&2 < 1) & ((PE1 + 0.5 * QD) > 2.5)
Metrichor	Heuristic with phasing	0.877	0.07	0.17	QUAL > 30 & (0 < SB1&2 < 1) & (max(SRP, SAP) < 30)
Metrichor	Heuristic with phasing	0.88	0.062	0.172	QUAL > 30 & (0 < SB1&2 < 1) & (max(SRP, SAP) < 30) & ((PE1 + 0.5 * QD) > 2)
Metrichor	Heuristic with phasing	0.88	0.058	0.174	QUAL > 30 & (0 < SB1&2 < 1) & (max(SRP, SAP) < 30) & ((PE1 + 0.5 * QD) > 2.25)
Metrichor	Heuristic with phasing	0.88	0.054	0.177	QUAL > 30 & (0 < SB1&2 < 1) & (max(SRP, SAP) < 30) & ((PE1 + 0.5 * QD) > 2.5)
Metrichor	Heuristic with phasing	0.89	0.062	0.153	QUAL > 30 & GT != 0|0
Metrichor	Heuristic with phasing	0.892	0.057	0.154	QUAL > 30 & GT != 0|0 & ((PE1 + 0.5 * QD) > 2)
Metrichor	Heuristic with phasing	0.892	0.055	0.156	QUAL > 30 & GT != 0|0 & ((PE1 + 0.5 * QD) > 2.25)
Metrichor	Heuristic with phasing	0.891	0.052	0.159	QUAL > 30 & GT != 0|0 & ((PE1 + 0.5 * QD) > 2.5)
Metrichor	Heuristic with phasing	0.886	0.056	0.166	QUAL > 30 & GT != 0|0 & (max(SRP, SAP) < 30)
Metrichor	Heuristic with phasing	0.886	0.053	0.168	QUAL > 30 & GT != 0|0 & (max(SRP, SAP) < 30) & ((PE1 + 0.5 * QD) > 2)
Metrichor	Heuristic with phasing	0.886	0.051	0.169	QUAL > 30 & GT != 0|0 & (max(SRP, SAP) < 30) & ((PE1 + 0.5 * QD) > 2.25)
Metrichor	Heuristic with phasing	0.885	0.049	0.172	QUAL > 30 & GT != 0|0 & (max(SRP, SAP) < 30) & ((PE1 + 0.5 * QD) > 2.5)
Metrichor	Heuristic with phasing	0.889	0.056	0.16	QUAL > 30 & GT != 0|0 & (0 < SB1&2 < 1)
Metrichor	Heuristic with phasing	0.89	0.052	0.162	QUAL > 30 & GT != 0|0 & (0 < SB1&2 < 1) & ((PE1 + 0.5 * QD) > 2)
Metrichor	Heuristic with phasing	0.89	0.049	0.163	QUAL > 30 & GT != 0|0 & (0 < SB1&2 < 1) & ((PE1 + 0.5 * QD) > 2.25)
Metrichor	Heuristic with phasing	0.89	0.046	0.166	QUAL > 30 & GT != 0|0 & (0 < SB1&2 < 1) & ((PE1 + 0.5 * QD) > 2.5)
Metrichor	Heuristic with phasing	0.883	0.051	0.174	QUAL > 30 & GT != 0|0 & (0 < SB1&2 < 1) & (max(SRP, SAP) < 30)
Metrichor	Heuristic with phasing	0.884	0.048	0.175	QUAL > 30 & GT != 0|0 & (0 < SB1&2 < 1) & (max(SRP, SAP) < 30) & ((PE1 + 0.5 * QD) > 2)
Metrichor	Heuristic with phasing	0.884	0.046	0.176	QUAL > 30 & GT != 0|0 & (0 < SB1&2 < 1) & (max(SRP, SAP) < 30) & ((PE1 + 0.5 * QD) > 2.25)
Metrichor	Heuristic with phasing	0.883	0.044	0.179	QUAL > 30 & GT != 0|0 & (0 < SB1&2 < 1) & (max(SRP, SAP) < 30) & ((PE1 + 0.5 * QD) > 2.5)
Nanonet	Heuristic with phasing	0.763	0.285	0.181	QUAL > 30
Nanonet	Heuristic with phasing	0.827	0.162	0.184	QUAL > 30 & ((PE1 + 0.5 * QD) > 2)
Nanonet	Heuristic with phasing	0.836	0.138	0.188	QUAL > 30 & ((PE1 + 0.5 * QD) > 2.25)
Nanonet	Heuristic with phasing	0.839	0.123	0.196	QUAL > 30 & ((PE1 + 0.5 * QD) > 2.5)
Nanonet	Heuristic with phasing	0.812	0.183	0.193	QUAL > 30 & (max(SRP, SAP) < 30)
Nanonet	Heuristic with phasing	0.841	0.119	0.195	QUAL > 30 & (max(SRP, SAP) < 30) & ((PE1 + 0.5 * QD) > 2)
Nanonet	Heuristic with phasing	0.845	0.104	0.2	QUAL > 30 & (max(SRP, SAP) < 30) & ((PE1 + 0.5 * QD) > 2.25)
Nanonet	Heuristic with phasing	0.845	0.096	0.207	QUAL > 30 & (max(SRP, SAP) < 30) & ((PE1 + 0.5 * QD) > 2.5)
Nanonet	Heuristic with phasing	0.769	0.262	0.197	QUAL > 30 & (0 < SB1&2 < 1)
Nanonet	Heuristic with phasing	0.835	0.129	0.199	QUAL > 30 & (0 < SB1&2 < 1) & ((PE1 + 0.5 * QD) > 2)
Nanonet	Heuristic with phasing	0.843	0.103	0.204	QUAL > 30 & (0 < SB1&2 < 1) & ((PE1 + 0.5 * QD) > 2.25)
Nanonet	Heuristic with phasing	0.846	0.089	0.211	QUAL > 30 & (0 < SB1&2 < 1) & ((PE1 + 0.5 * QD) > 2.5)
Nanonet	Heuristic with phasing	0.815	0.161	0.207	QUAL > 30 & (0 < SB1&2 < 1) & (max(SRP, SAP) < 30)
Nanonet	Heuristic with phasing	0.844	0.093	0.21	QUAL > 30 & (0 < SB1&2 < 1) & (max(SRP, SAP) < 30) & ((PE1 + 0.5 * QD) > 2)
Nanonet	Heuristic with phasing	0.849	0.077	0.214	QUAL > 30 & (0 < SB1&2 < 1) & (max(SRP, SAP) < 30) & ((PE1 + 0.5 * QD) > 2.25)
Nanonet	Heuristic with phasing	0.848	0.069	0.221	QUAL > 30 & (0 < SB1&2 < 1) & (max(SRP, SAP) < 30) & ((PE1 + 0.5 * QD) > 2.5)
Nanonet	Heuristic with phasing	0.81	0.193	0.186	QUAL > 30 & GT != 0|0
Nanonet	Heuristic with phasing	0.831	0.152	0.186	QUAL > 30 & GT != 0|0 & ((PE1 + 0.5 * QD) > 2)
Nanonet	Heuristic with phasing	0.838	0.132	0.19	QUAL > 30 & GT != 0|0 & ((PE1 + 0.5 * QD) > 2.25)
Nanonet	Heuristic with phasing	0.841	0.119	0.196	QUAL > 30 & GT != 0|0 & ((PE1 + 0.5 * QD) > 2.5)
Nanonet	Heuristic with phasing	0.838	0.124	0.197	QUAL > 30 & GT != 0|0 & (max(SRP, SAP) < 30)
Nanonet	Heuristic with phasing	0.844	0.111	0.197	QUAL > 30 & GT != 0|0 & (max(SRP, SAP) < 30) & ((PE1 + 0.5 * QD) > 2)
Nanonet	Heuristic with phasing	0.846	0.1	0.201	QUAL > 30 & GT != 0|0 & (max(SRP, SAP) < 30) & ((PE1 + 0.5 * QD) > 2.25)
Nanonet	Heuristic with phasing	0.847	0.092	0.207	QUAL > 30 & GT != 0|0 & (max(SRP, SAP) < 30) & ((PE1 + 0.5 * QD) > 2.5)
Nanonet	Heuristic with phasing	0.818	0.162	0.201	QUAL > 30 & GT != 0|0 & (0 < SB1&2 < 1)
Nanonet	Heuristic with phasing	0.838	0.118	0.201	QUAL > 30 & GT != 0|0 & (0 < SB1&2 < 1) & ((PE1 + 0.5 * QD) > 2)
Nanonet	Heuristic with phasing	0.845	0.098	0.205	QUAL > 30 & GT != 0|0 & (0 < SB1&2 < 1) & ((PE1 + 0.5 * QD) > 2.25)
Nanonet	Heuristic with phasing	0.847	0.085	0.211	QUAL > 30 & GT != 0|0 & (0 < SB1&2 < 1) & ((PE1 + 0.5 * QD) > 2.5)
Nanonet	Heuristic with phasing	0.842	0.098	0.211	QUAL > 30 & GT != 0|0 & (0 < SB1&2 < 1) & (max(SRP, SAP) < 30)
Nanonet	Heuristic with phasing	0.847	0.084	0.212	QUAL > 30 & GT != 0|0 & (0 < SB1&2 < 1) & (max(SRP, SAP) < 30) & ((PE1 + 0.5 * QD) > 2)
Nanonet	Heuristic with phasing	0.851	0.072	0.215	QUAL > 30 & GT != 0|0 & (0 < SB1&2 < 1) & (max(SRP, SAP) < 30) & ((PE1 + 0.5 * QD) > 2.25)
Nanonet	Heuristic with phasing	0.85	0.065	0.221	QUAL > 30 & GT != 0|0 & (0 < SB1&2 < 1) & (max(SRP, SAP) < 30) & ((PE1 + 0.5 * QD) > 2.5)
Scrappie	Heuristic with phasing	0.757	0.28	0.203	QUAL > 30
Scrappie	Heuristic with phasing	0.757	0.279	0.203	QUAL > 30 & ((PE1 + 0.5 * QD) > 2)
Scrappie	Heuristic with phasing	0.758	0.278	0.203	QUAL > 30 & ((PE1 + 0.5 * QD) > 2.25)
Scrappie	Heuristic with phasing	0.758	0.277	0.203	QUAL > 30 & ((PE1 + 0.5 * QD) > 2.5)
Scrappie	Heuristic with phasing	0.766	0.254	0.212	QUAL > 30 & (max(SRP, SAP) < 30)
Scrappie	Heuristic with phasing	0.767	0.253	0.212	QUAL > 30 & (max(SRP, SAP) < 30) & ((PE1 + 0.5 * QD) > 2)
Scrappie	Heuristic with phasing	0.767	0.252	0.212	QUAL > 30 & (max(SRP, SAP) < 30) & ((PE1 + 0.5 * QD) > 2.25)
Scrappie	Heuristic with phasing	0.767	0.252	0.212	QUAL > 30 & (max(SRP, SAP) < 30) & ((PE1 + 0.5 * QD) > 2.5)
Scrappie	Heuristic with phasing	0.755	0.28	0.206	QUAL > 30 & (0 < SB1&2 < 1)
Scrappie	Heuristic with phasing	0.756	0.279	0.206	QUAL > 30 & (0 < SB1&2 < 1) & ((PE1 + 0.5 * QD) > 2)
Scrappie	Heuristic with phasing	0.756	0.278	0.206	QUAL > 30 & (0 < SB1&2 < 1) & ((PE1 + 0.5 * QD) > 2.25)
Scrappie	Heuristic with phasing	0.756	0.277	0.207	QUAL > 30 & (0 < SB1&2 < 1) & ((PE1 + 0.5 * QD) > 2.5)
Scrappie	Heuristic with phasing	0.765	0.254	0.215	QUAL > 30 & (0 < SB1&2 < 1) & (max(SRP, SAP) < 30)
Scrappie	Heuristic with phasing	0.766	0.253	0.215	QUAL > 30 & (0 < SB1&2 < 1) & (max(SRP, SAP) < 30) & ((PE1 + 0.5 * QD) > 2)
Scrappie	Heuristic with phasing	0.766	0.252	0.215	QUAL > 30 & (0 < SB1&2 < 1) & (max(SRP, SAP) < 30) & ((PE1 + 0.5 * QD) > 2.25)
Scrappie	Heuristic with phasing	0.766	0.252	0.215	QUAL > 30 & (0 < SB1&2 < 1) & (max(SRP, SAP) < 30) & ((PE1 + 0.5 * QD) > 2.5)
Scrappie	Heuristic with phasing	0.841	0.085	0.222	QUAL > 30 & GT != 0|0
Scrappie	Heuristic with phasing	0.842	0.083	0.222	QUAL > 30 & GT != 0|0 & ((PE1 + 0.5 * QD) > 2)
Scrappie	Heuristic with phasing	0.842	0.082	0.222	QUAL > 30 & GT != 0|0 & ((PE1 + 0.5 * QD) > 2.25)
Scrappie	Heuristic with phasing	0.842	0.081	0.223	QUAL > 30 & GT != 0|0 & ((PE1 + 0.5 * QD) > 2.5)
Scrappie	Heuristic with phasing	0.841	0.072	0.231	QUAL > 30 & GT != 0|0 & (max(SRP, SAP) < 30)
Scrappie	Heuristic with phasing	0.841	0.071	0.231	QUAL > 30 & GT != 0|0 & (max(SRP, SAP) < 30) & ((PE1 + 0.5 * QD) > 2)
Scrappie	Heuristic with phasing	0.841	0.071	0.231	QUAL > 30 & GT != 0|0 & (max(SRP, SAP) < 30) & ((PE1 + 0.5 * QD) > 2.25)
Scrappie	Heuristic with phasing	0.841	0.07	0.232	QUAL > 30 & GT != 0|0 & (max(SRP, SAP) < 30) & ((PE1 + 0.5 * QD) > 2.5)
Scrappie	Heuristic with phasing	0.839	0.084	0.226	QUAL > 30 & GT != 0|0 & (0 < SB1&2 < 1)
Scrappie	Heuristic with phasing	0.839	0.083	0.226	QUAL > 30 & GT != 0|0 & (0 < SB1&2 < 1) & ((PE1 + 0.5 * QD) > 2)
Scrappie	Heuristic with phasing	0.84	0.081	0.226	QUAL > 30 & GT != 0|0 & (0 < SB1&2 < 1) & ((PE1 + 0.5 * QD) > 2.25)
Scrappie	Heuristic with phasing	0.841	0.08	0.226	QUAL > 30 & GT != 0|0 & (0 < SB1&2 < 1) & ((PE1 + 0.5 * QD) > 2.5)
Scrappie	Heuristic with phasing	0.839	0.072	0.234	QUAL > 30 & GT != 0|0 & (0 < SB1&2 < 1) & (max(SRP, SAP) < 30)
Scrappie	Heuristic with phasing	0.839	0.071	0.235	QUAL > 30 & GT != 0|0 & (0 < SB1&2 < 1) & (max(SRP, SAP) < 30) & ((PE1 + 0.5 * QD) > 2)
Scrappie	Heuristic with phasing	0.839	0.07	0.235	QUAL > 30 & GT != 0|0 & (0 < SB1&2 < 1) & (max(SRP, SAP) < 30) & ((PE1 + 0.5 * QD) > 2.25)
Scrappie	Heuristic with phasing	0.84	0.069	0.235	QUAL > 30 & GT != 0|0 & (0 < SB1&2 < 1) & (max(SRP, SAP) < 30) & ((PE1 + 0.5 * QD) > 2.5)

3 Supplementary Figures

3.1 Supplementary Figures - NA12878

Supplementary Figure 1: Proportion of PASS/FAIL reads in NA12878 Proportion of reads per-flowcell that pass Albacore v2.0.2 QC metrics (PASS) or do not (FAIL), sorted by flowcell run date.

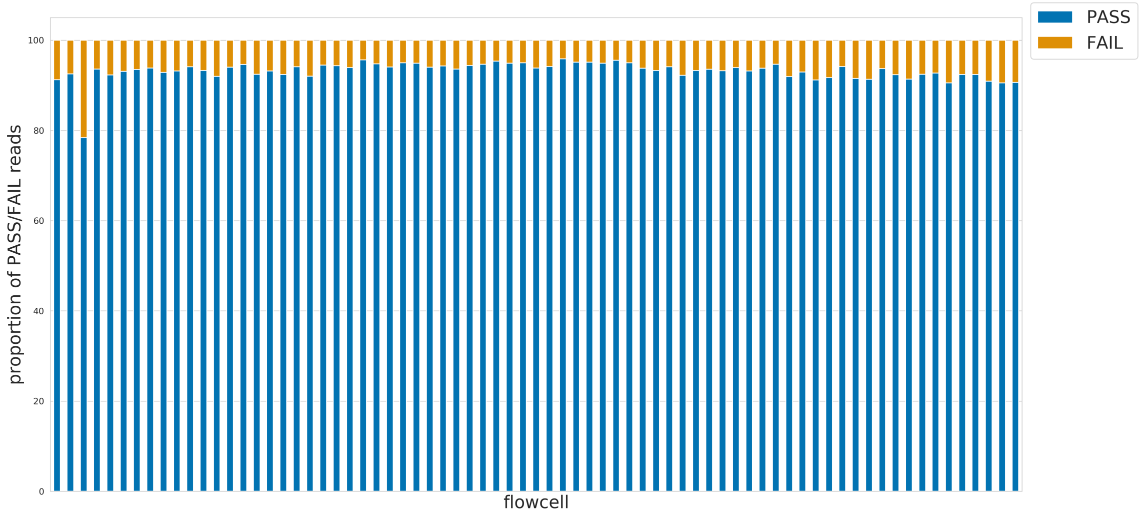

Supplementary Figure 2: Substitution error rates in NA12878 sample Different colours correspond to different flow cells.

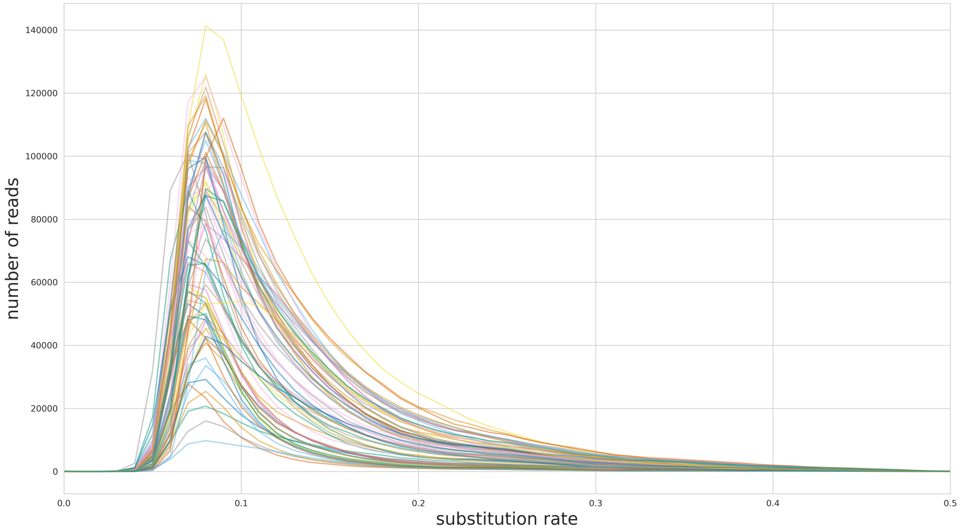

Supplementary Figure 3: Distribution of substitution error rates in alignments from different base-callers Distribution of substitution error rates in alignments from different base-callers

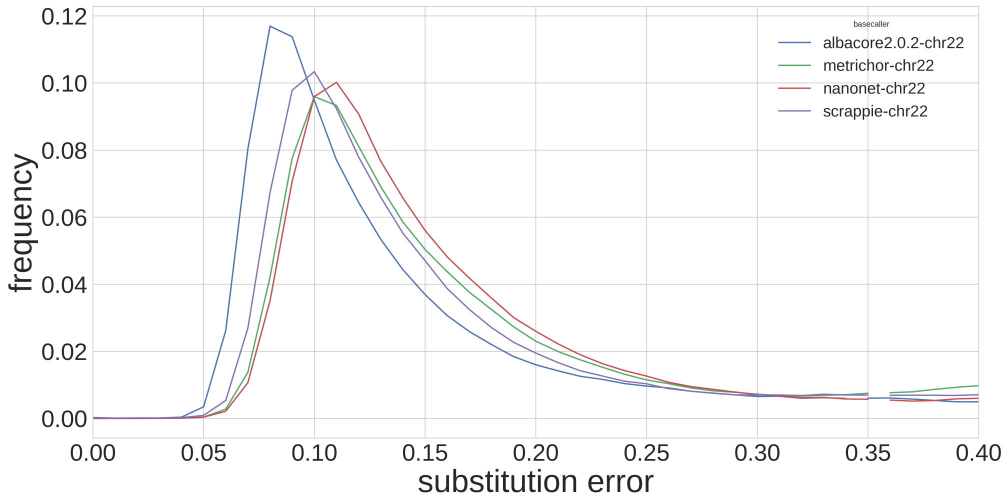

Supplementary Figure 4: Distribution of insertion error rates in alignments from different base-callers Distribution of insertion error rates in alignments from different base-callers

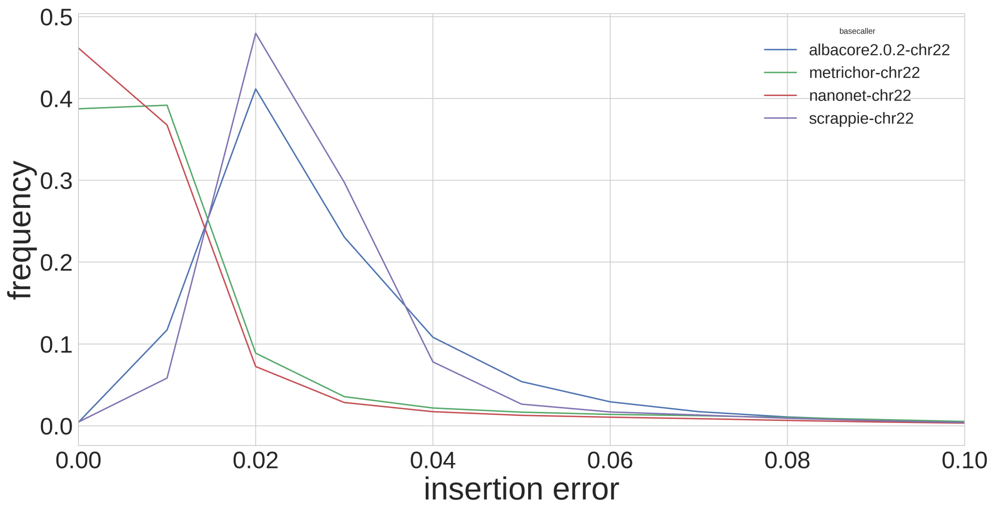

Supplementary Figure 5: Distribution of deletion error rates in alignments from different base-callers Distribution of deletion error rates in alignments from different base-callers

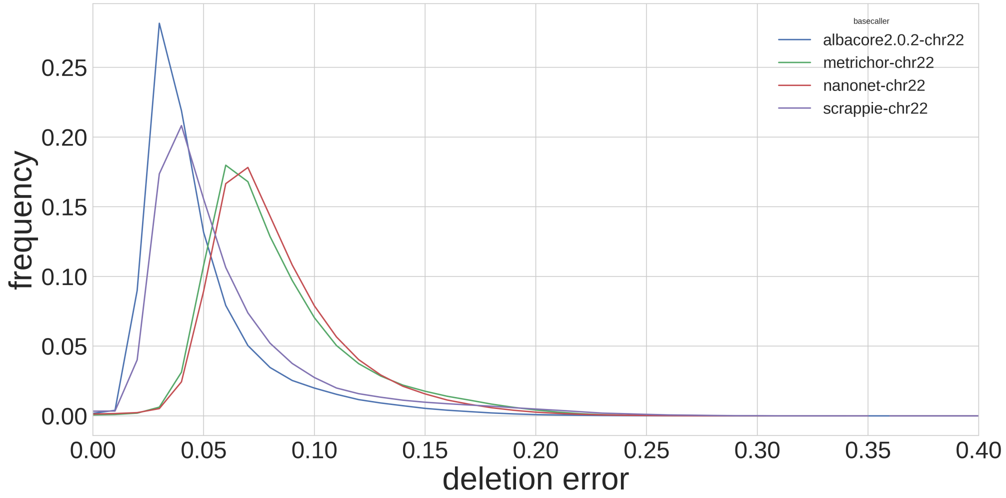

Supplementary Figure 6: Visualization of metrics for Albacore dataset after phasing Shown are SNVs from Albacore that were phased, which are a combination of true positives (green) and false positives (red). (Top left) Shown are the parameters estimated from phasing for the probability of the arbitrarily labelleled first and second haplotype of emitting an alternate allele. After phasing, SNVs with both haplotype probabilities have genotypes reset to 0/0. (Top right) Shown is the QUAL score versus the phasing entropy metric. Low scores indicate poorer phasing. (Bottom left) Shown is quality by depth versus the phasing entropy metric. This slightly cleaner view permits more precise distinguishing of true versus false positives. Lines indicate cutoffs considered. (Bottom right) Phasing derived strand biases for the two haplotypes.

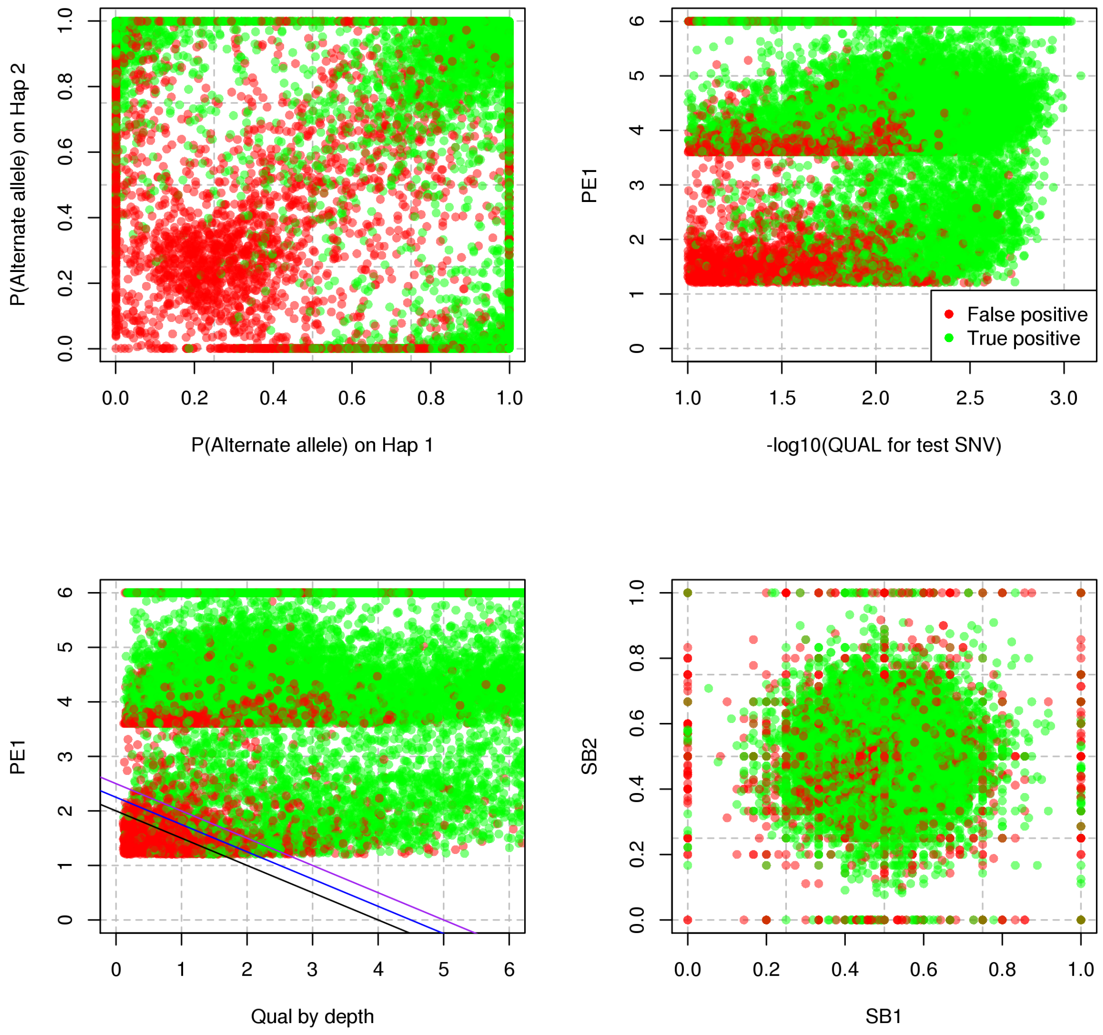

Supplementary Figure 7: Read depth distributions illustrate different error modes Read depth at variant calling sites in the NA12878 genome (chr22). Read depth is listed for false negative calls (“fn”), false positive calls (“fp”), true negative calls (“tn”) and true positive calls (“tp”). “ref” shows all variant sites within the NA12878 genome. Sites with low depth are enriched in false negatives and false positives variant calls. False positives variant calls have a bimodal read depth distribution, indicating two separate error modes, one that is dependent on depth, and one that is not. Read depth is measured as the mean read depth in a 40bp window around a site.

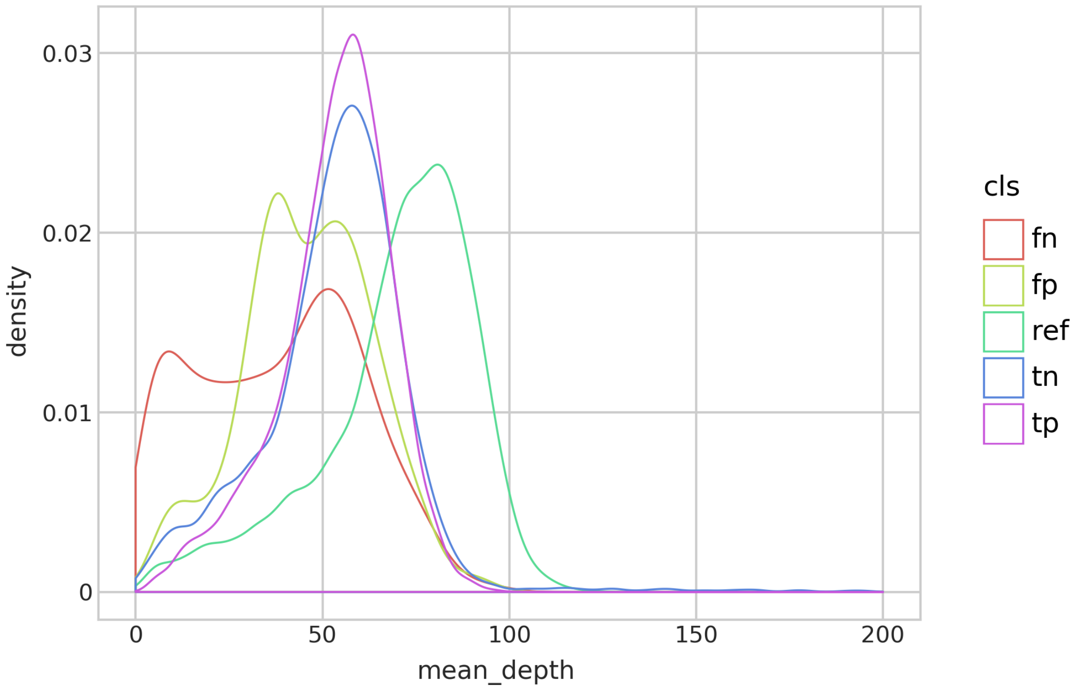

Supplementary Figure 8: Allele frequencies show alignment bias Allele frequencies at sites in the NA12878 genome (chr22). The figure shows the allele frequency at heterozygous sites ("het"), sites that are homozoygous for the alternative allele ("hom alt") and sites that are homozygous for the reference allele ("hom ref"). Allele frequencies are shown for the reference allele ("freq_ref") and the alternate allele ("freq_alt"). The modes of the reference allele frequency at homozygous reference positions and the alternate allele frequency at homozygous alternate allele positions are shifted from their expectation at 0 and 1, respectively. Part of the shift is explained by the substitution error rate in the sequence data. However, the larger shift of alternate allele frequencies at sites that are homozygous for the alternate allele ("hom-alt freq_alt") compared to reference allele frequencies at sites that are homozygous for the reference allele ("hom-ref freq_ref") indicates a reference alignment bias: the alignment algorithm places gaps to minimize the number of substitutions with respect to the reference sequence. Similarly shifted from the expectation at 0.5 are allele frequencies at heterozygous sites with a higher frequency of the reference allele compared to the alternate allele.

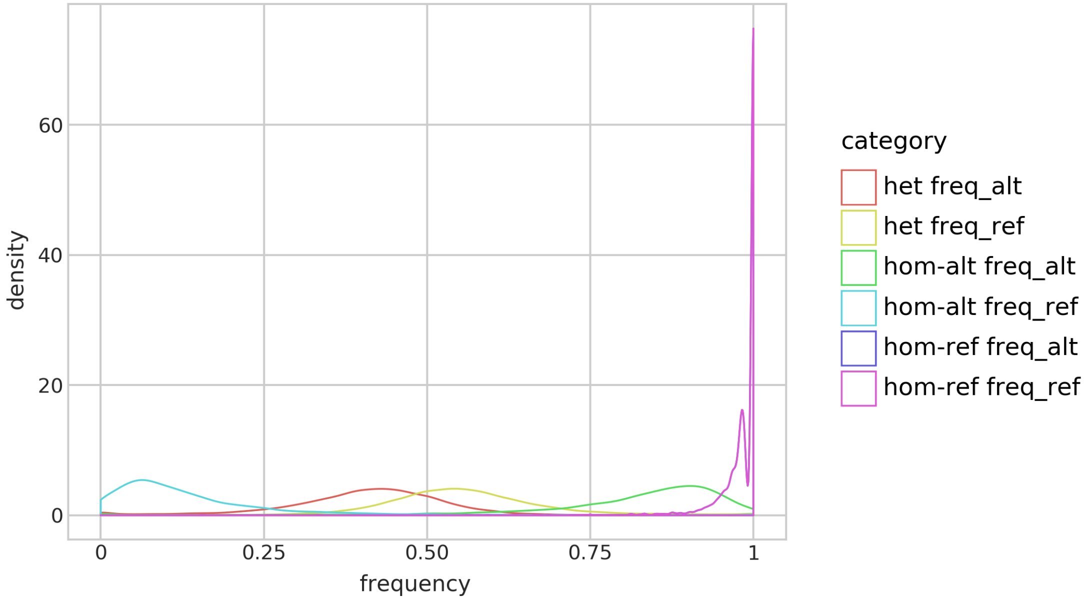

Supplementary Figure 9: Kmer frequencies at false positive sites indicate base-calling bias Shown here are frequencies of kmers of size 3. Each dot represent a particular kmer, with the X-axis showing the frequency of this kmer in chr22. The Y-axis shows the frequency of the kmer in the set of false positives. Deviation from the diagonal indicates an enrichment of a kmer in the set of false positives. A kmer is coloured black if it contains a CpG dinucleotide and orange if it does not. Rare kmers are enriched in the set of false positives and all include a CpG dinucleotide. CpG dinucleotides are rare in the genome. As a consequence, base callers trained on genomic data will have less training data available increasing the error rate at such sites.

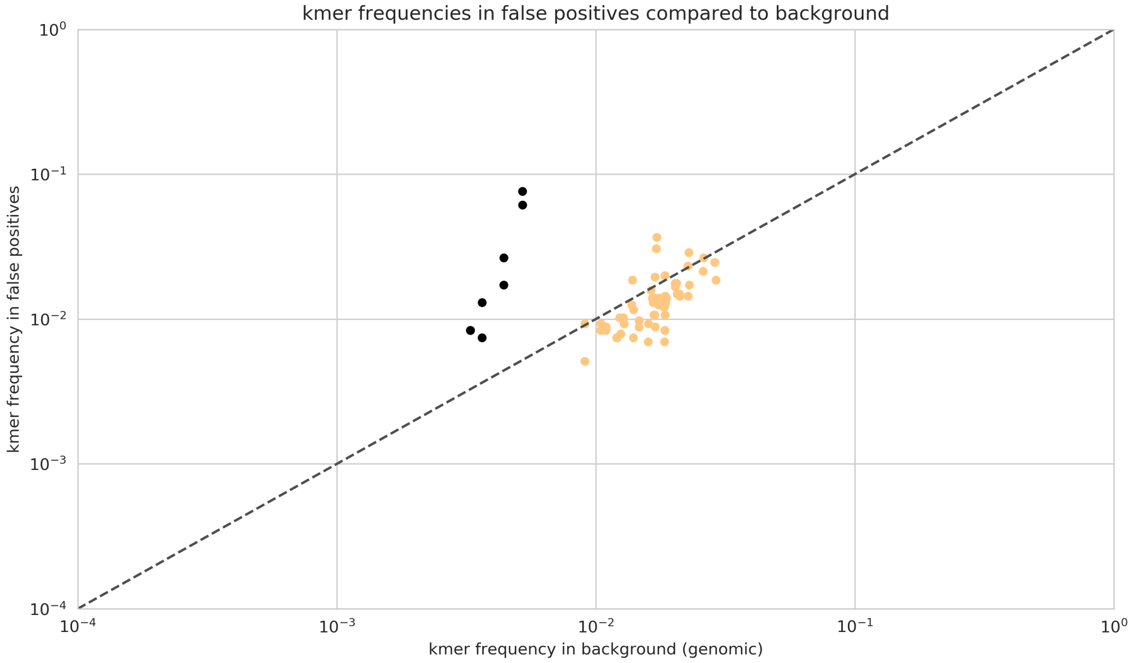

Supplementary Figure 10: Distribution of pLI scores in false positive and true positive variant calls located in protein coding genes. Both intronig and exonic variants are considered.

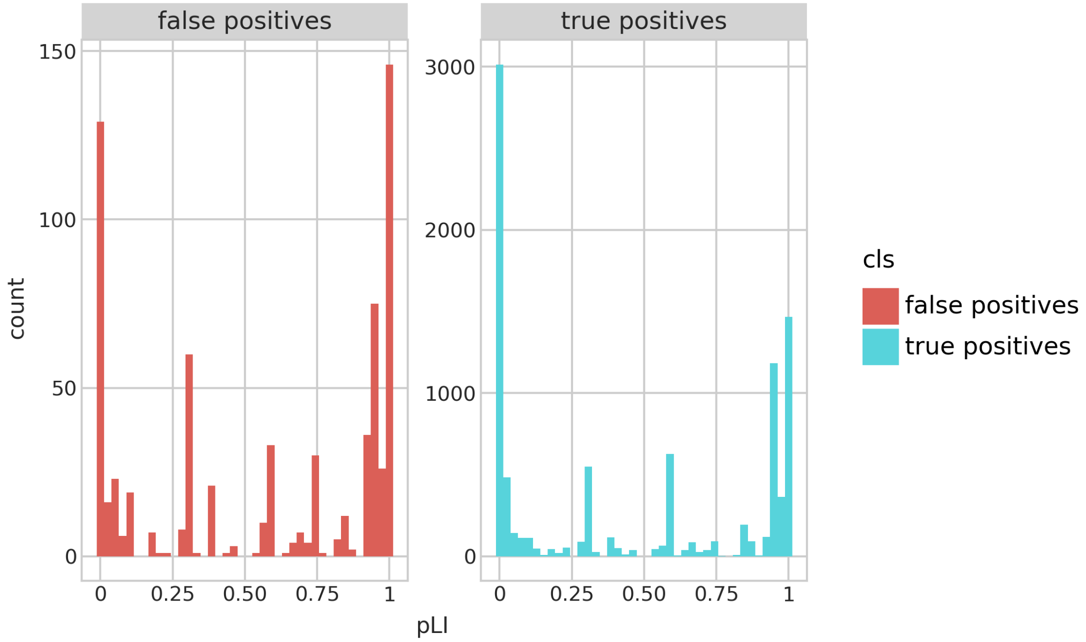

Supplementary Figure 11: Number of reads in the data sets for base-caller comparison The plot shows the number of reads in the different data sets generated for variant calling performance using different base callers. fixed_error: only use alignments with less than 20% error rate, fixed_size: remove 20% of alignments with the highest error rate, unfiltered: all alignments

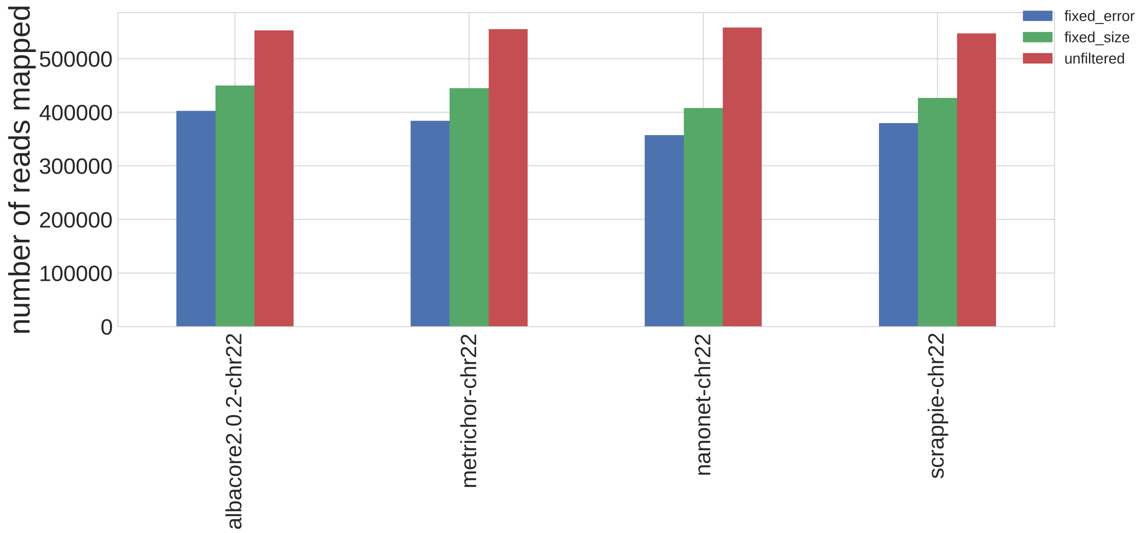

Supplementary Figure 12: Variant calling F1 measures for different base-called input The optimimum F1 value is shown based on optimizing across variant caller parameters and using a variant call quality threshold (QUAL) producing the highest F1 score.

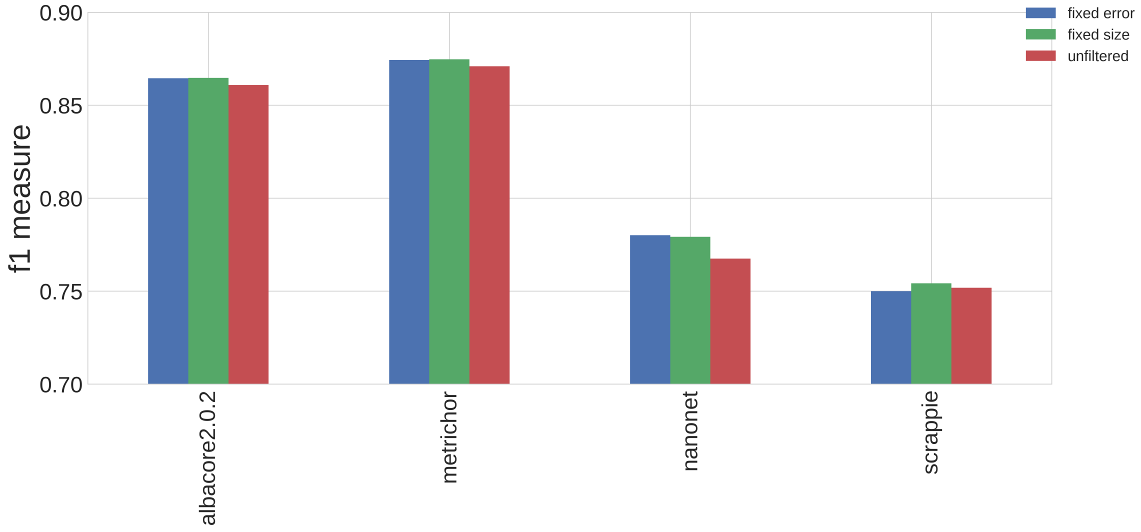

Supplementary Figure 13: Variant calling error rates for different base-called input False negative and discovery rates are shown based on optimizing the F1 metric across variant caller parameters and using a variant call quality threshold (QUAL) producing the highest F1 score. Also displayed is the false negative rate without optimization for QUAL, which gives an indication of the highest achievable sensitivity.

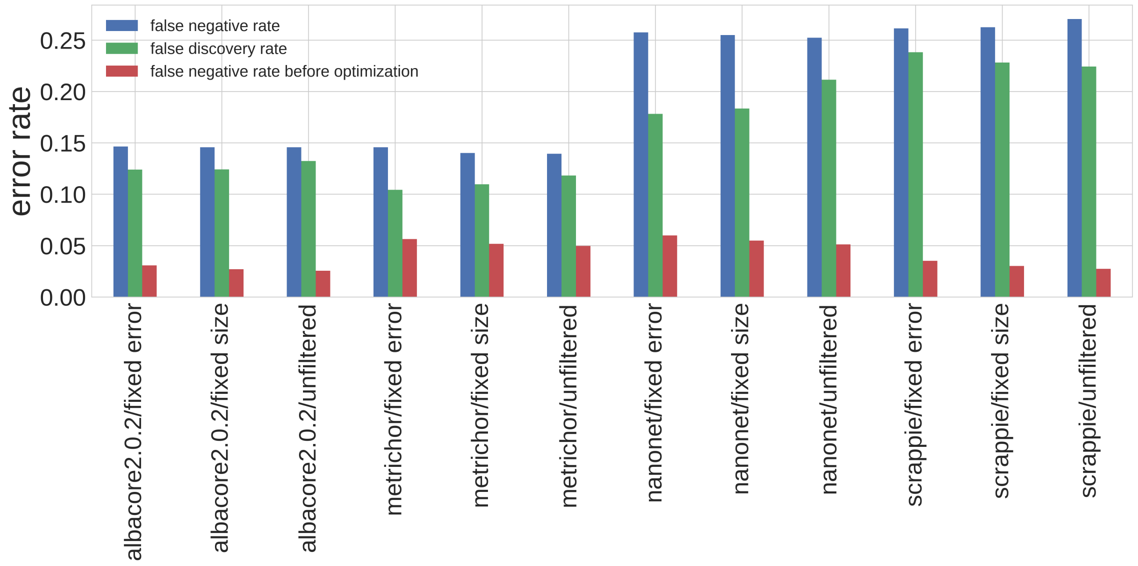

Supplementary Figure 14: Comparison of annotated errors among basecallers Annotation of called or truth SNPs using genomic features or sequencing context in NA12878 for the 4 basecallers, Albacore (Alb), Metrichor (Met), Nanonet (Nan) and Scrappie (Scr). Results are given for both pre-phasing (A, B) and post-phasing (C, D), either at all SNPs (A, C) or specifically focusing on those that have at least 60X coverage (B, D). The top panel gives counts of FDR and FNR for each basecaller, while the lower panels stratify SNPs that are true positives, false positives or false negatives into annotations that may reflect sources of SNP calling errors. Bars split into two or three represent the fraction of SNPs of that class that have multiple annotations. Annotations are the same as Figure 2 of the main text.

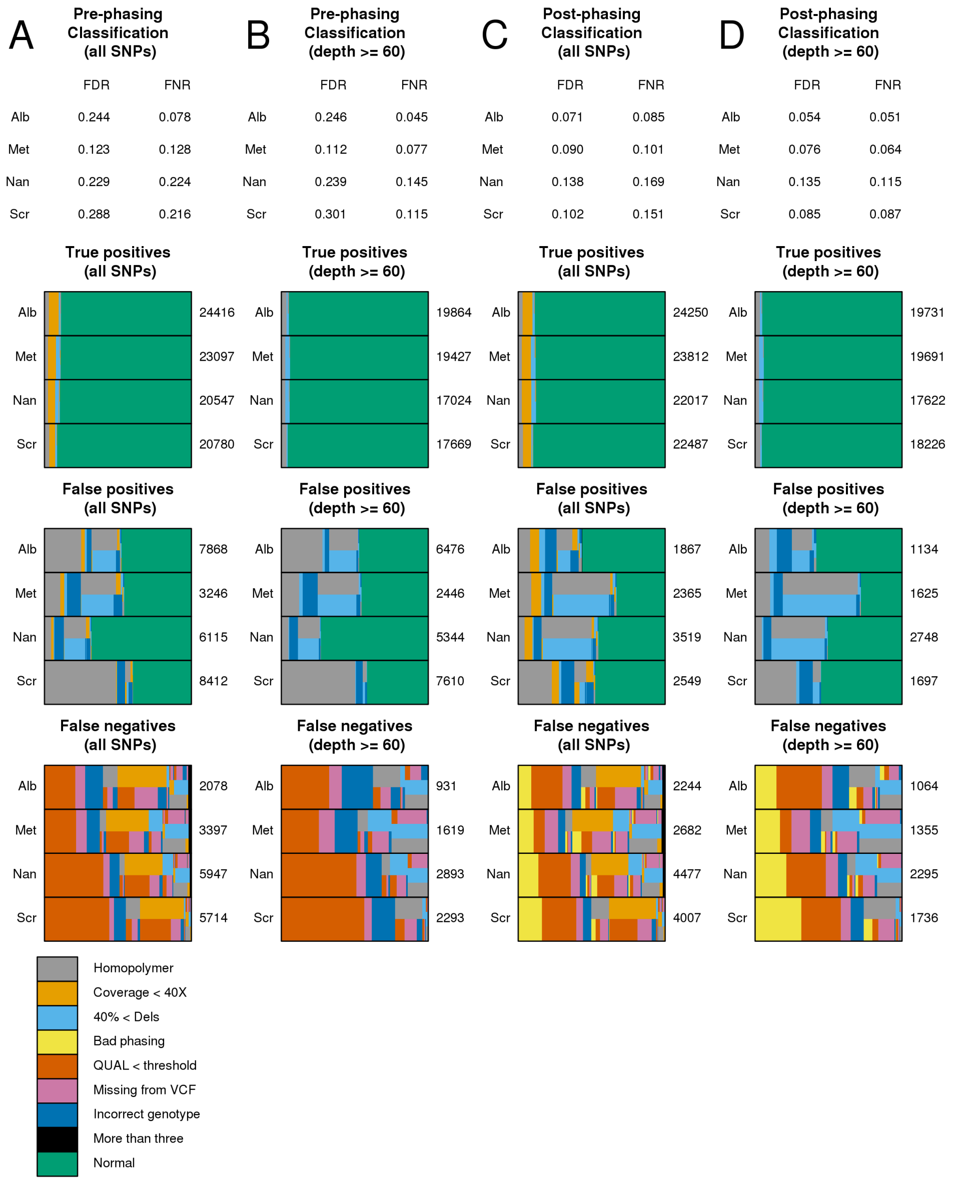

Supplementary Figure 15: Simulated error rates Error rates (substitution/deletion/insertion error) in simulated BAM Files. The legend indicates the shift in base quality score applied to each data set and the substitution/insertion/deletion ratio used. “Observed” are indicates the error rates observed from unsimulated data.

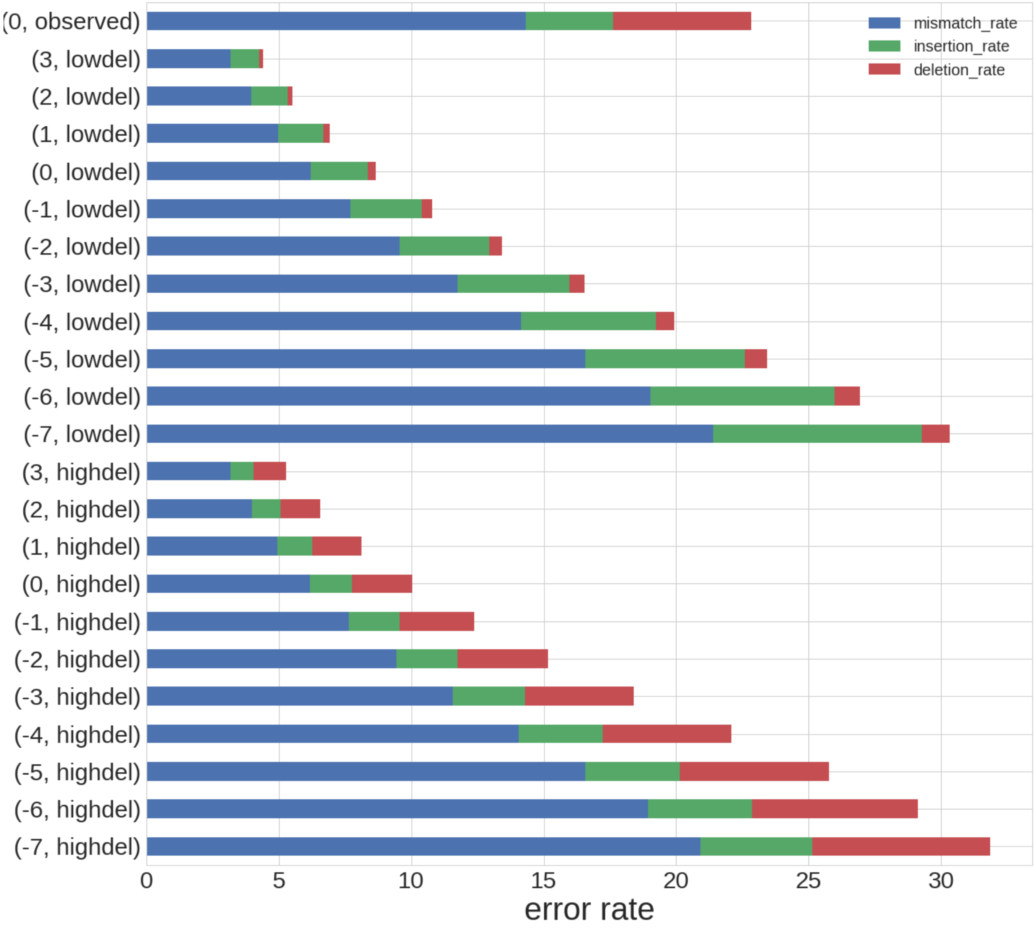

Supplementary Figure 16: Simulated read depth Observed read depths (coverage) in simulated data are close to the target depth.

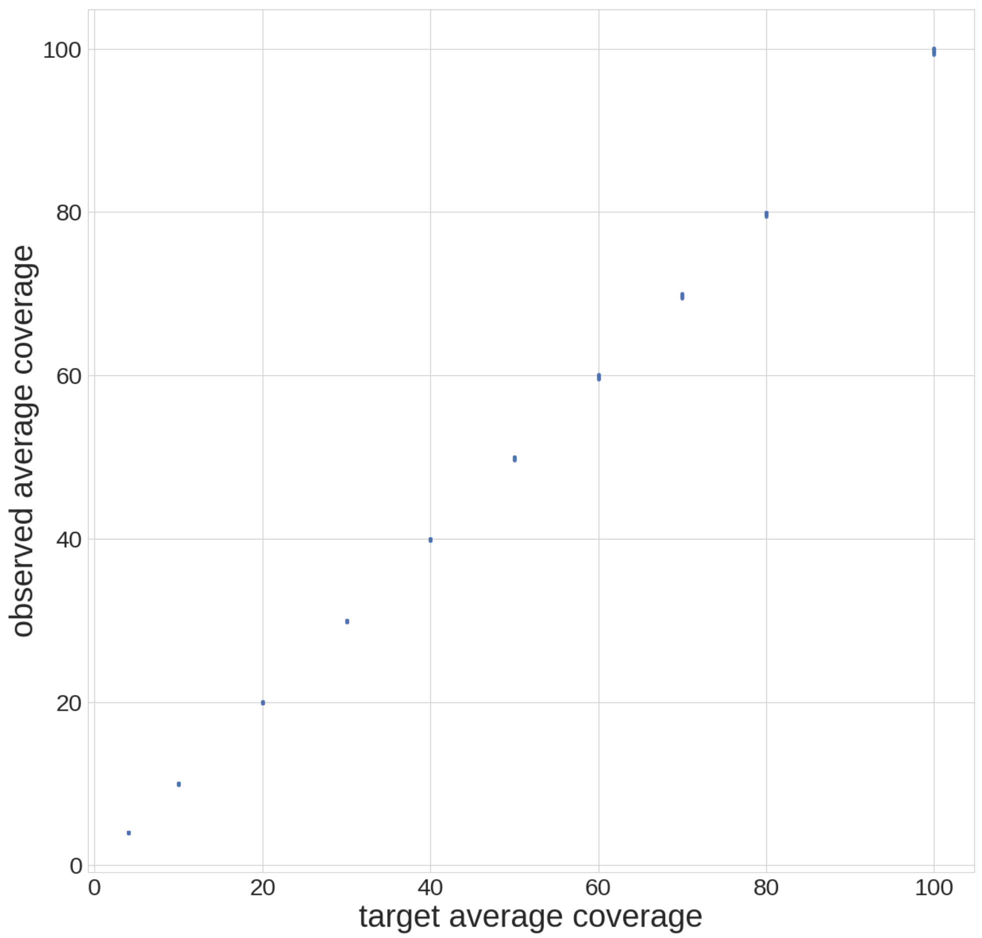

Supplementary Figure 17: Variant calling performance in mapped simulated data at different depths and error rates. Error rates are reported as substitution rate, insertion rate and deletion rate.

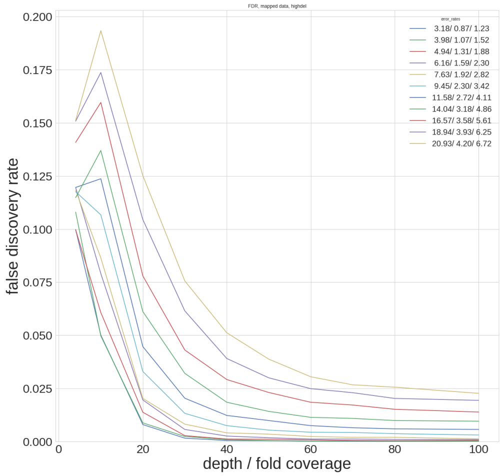

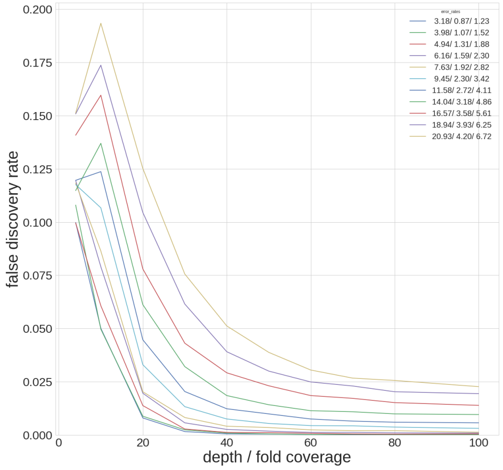

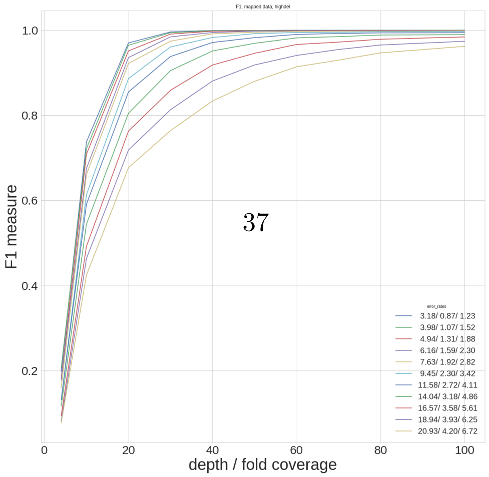

Supplementary Figure 18: IGV snapshot of alignment error near homopolymer from simulated data The figure shows three alignment tracks. Top track: simulated data using a high insertion/low deletion rate, middle track: simulated data using a high deletion/low insertion rate, bottom track: Illumina data. Note how deletions, indicated by black bars, are stacked at the beginning of a homopolymer while the homopolymer itself is depleted of deletions. As a consequence, substitution errors are enriched and a heterozygous variant is reported erroneously. On the other hand, insertions are uniformly placed within the hompolymer.

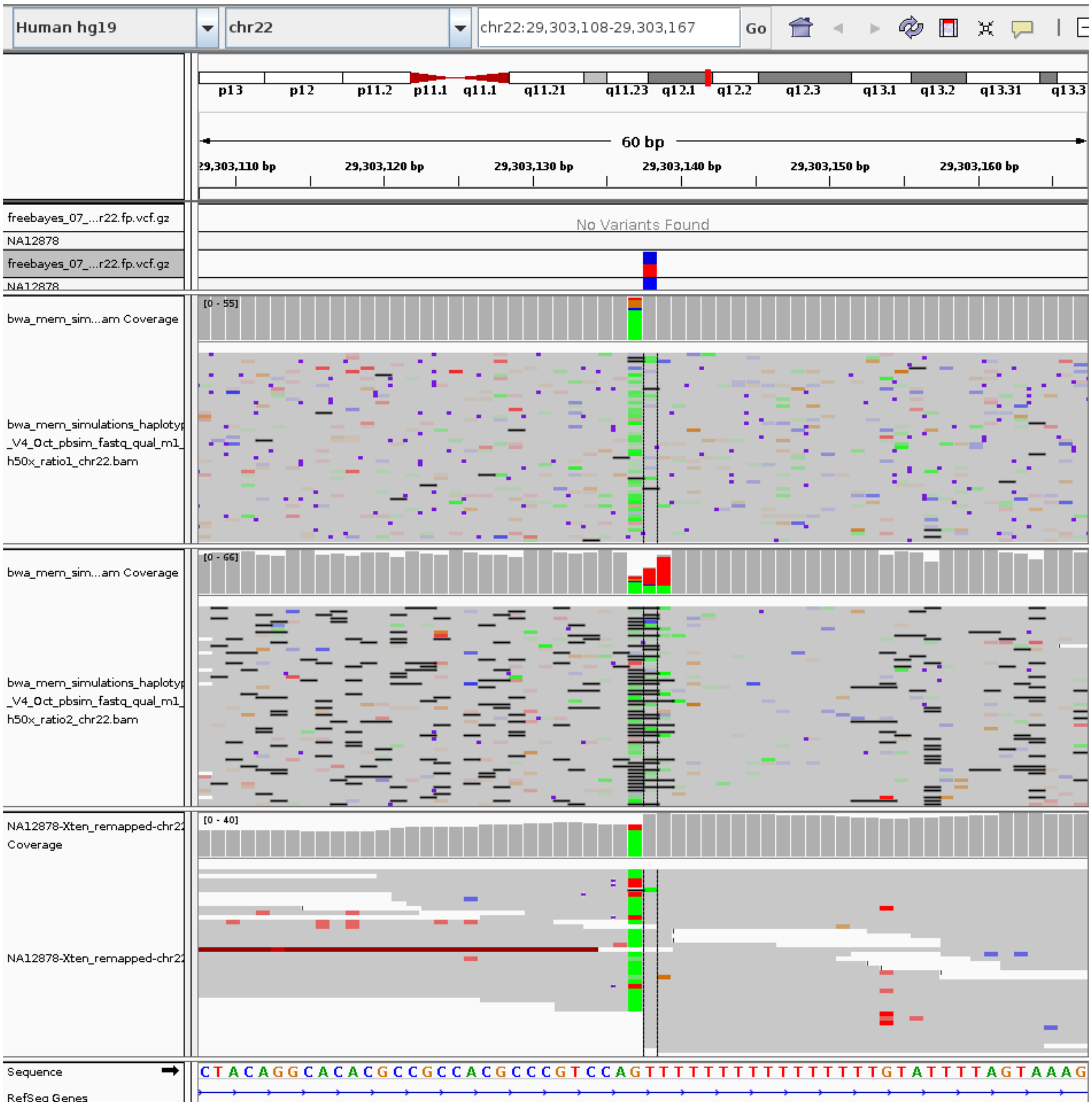

Supplementary Figure 19: Variant calling performance in unmapped simulated data at different depths and error rates. Base error rates are reported as substitution rate, insertion rate and deletion rate. Unmapped data has no variant calling errors due to alignment artifacts as the alignment contains the correct position of each base.

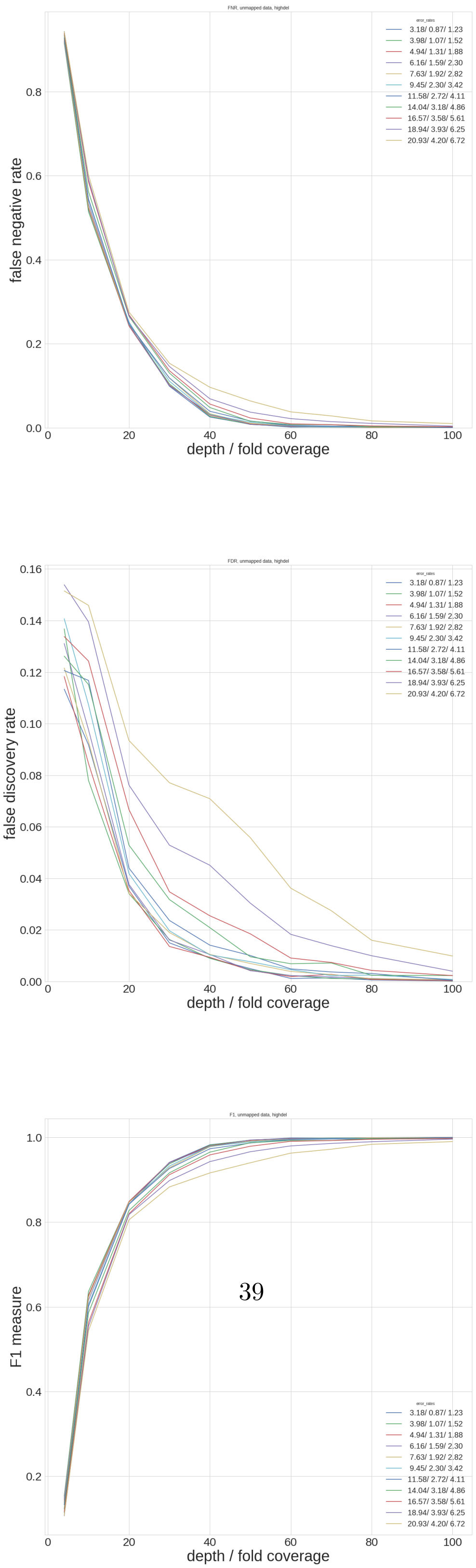

Supplementary Figure 20: Variant calling performance in mapped simulated data at different depths and error rates, with higher insertion error rates than deletion error rates. Base error rates are reported as substitution rate, insertion rate and deletion rate.

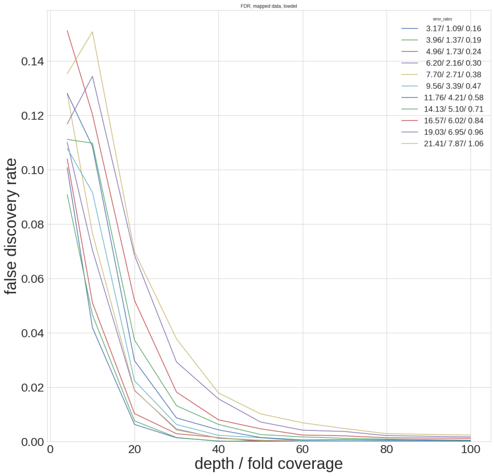

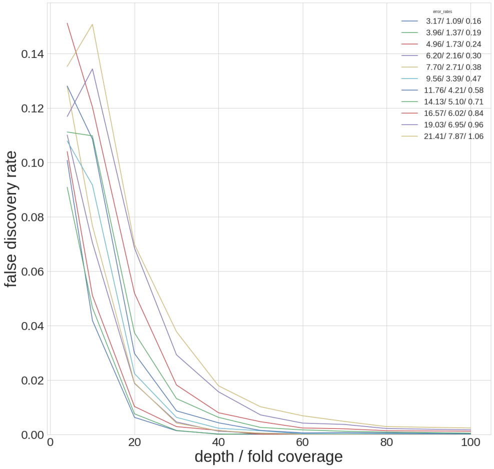

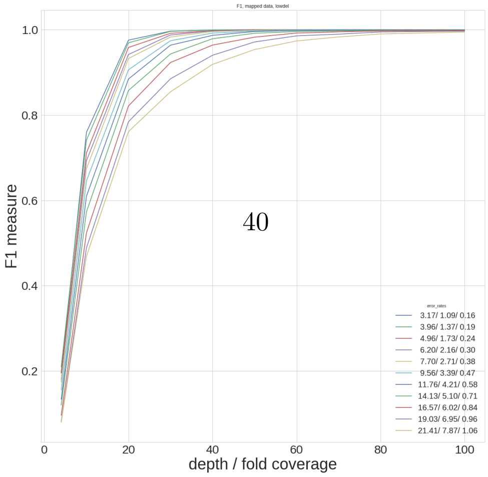

3.2 Supplementary Figures - LVC Snapshots

This section contains various IGV snapshots. The track layout in all snapshots is the same. The top of the figure shows repeat annotations from the UCSC genome browser. The middle section displays read data from ONT, Illumina and Pacbio. The bottom section contains large variants called by the SVClassify method (SVS), the Pacbio data set (PACBIO) and the large variant calls using the variant caller on ONT data (ONT) described in this manuscript.

3.2.1 Large variant calls in ONT data not present in reference

This section shows large variant calls based on ONT data, which are not present in reference data set and have no or weak evidence in PACBIO data. Based on visual inspection we believe these calls to be true positives.

Supplementary Figure 21: Subfigure 1

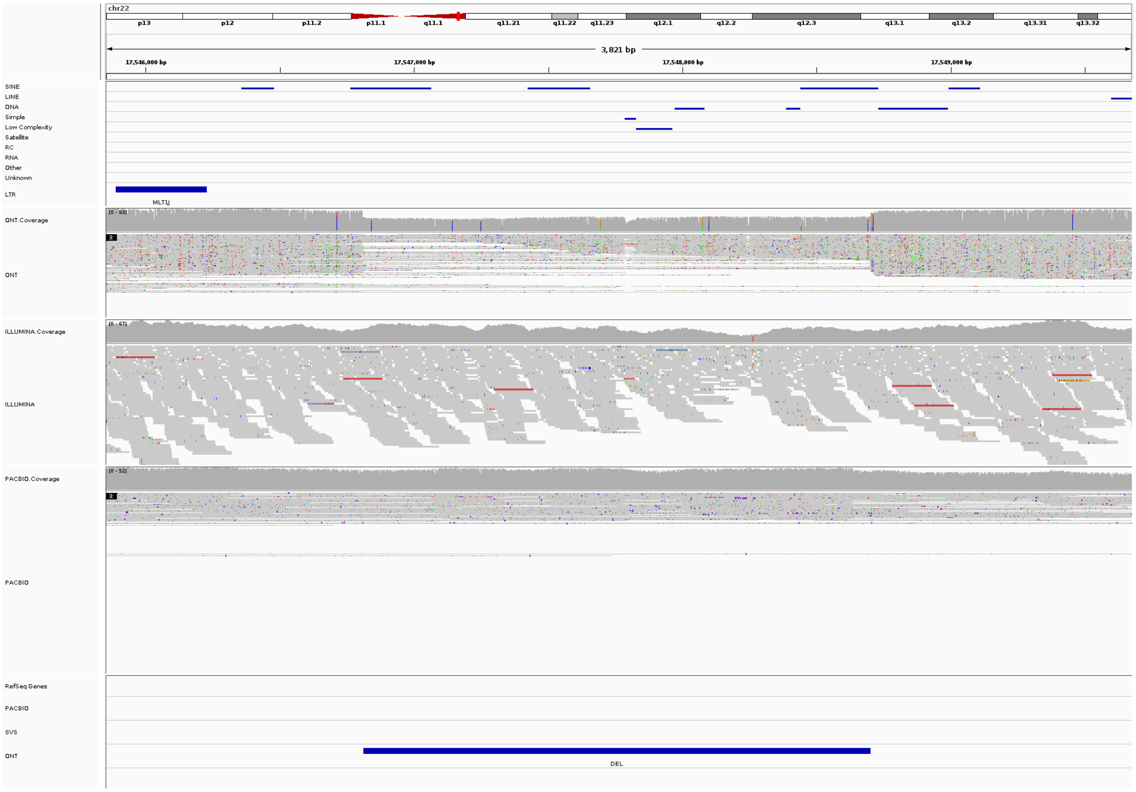

Supplementary Figure 21: Subfigure 2

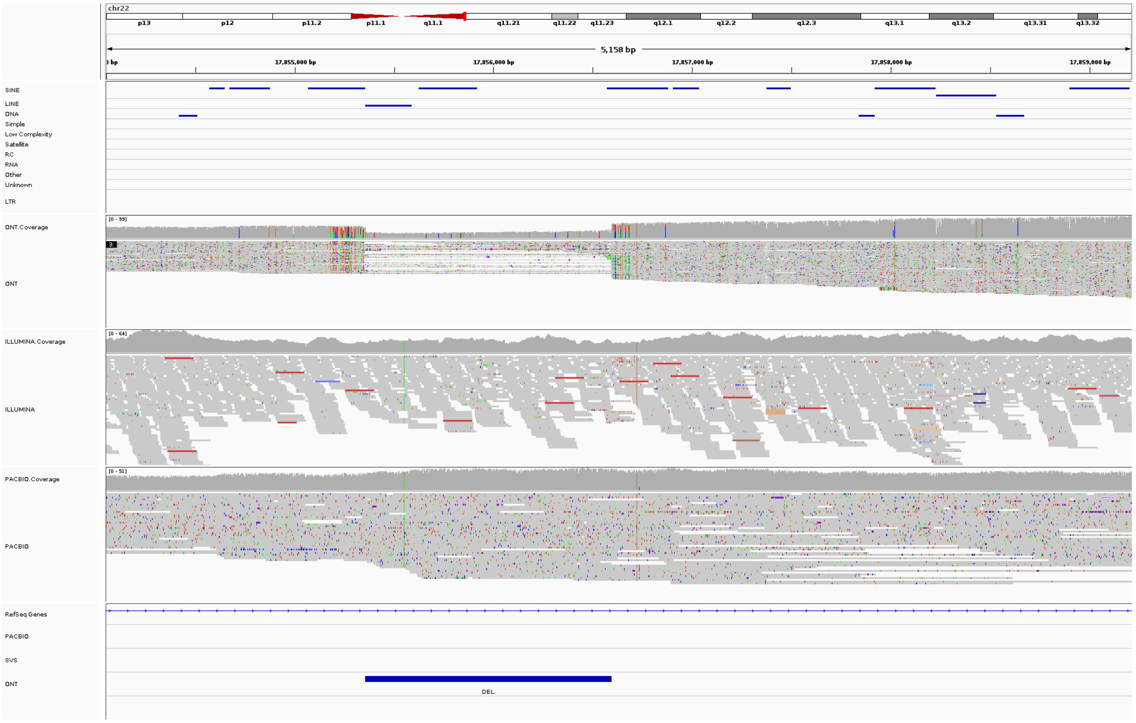

Supplementary Figure 21: Subfigure 3

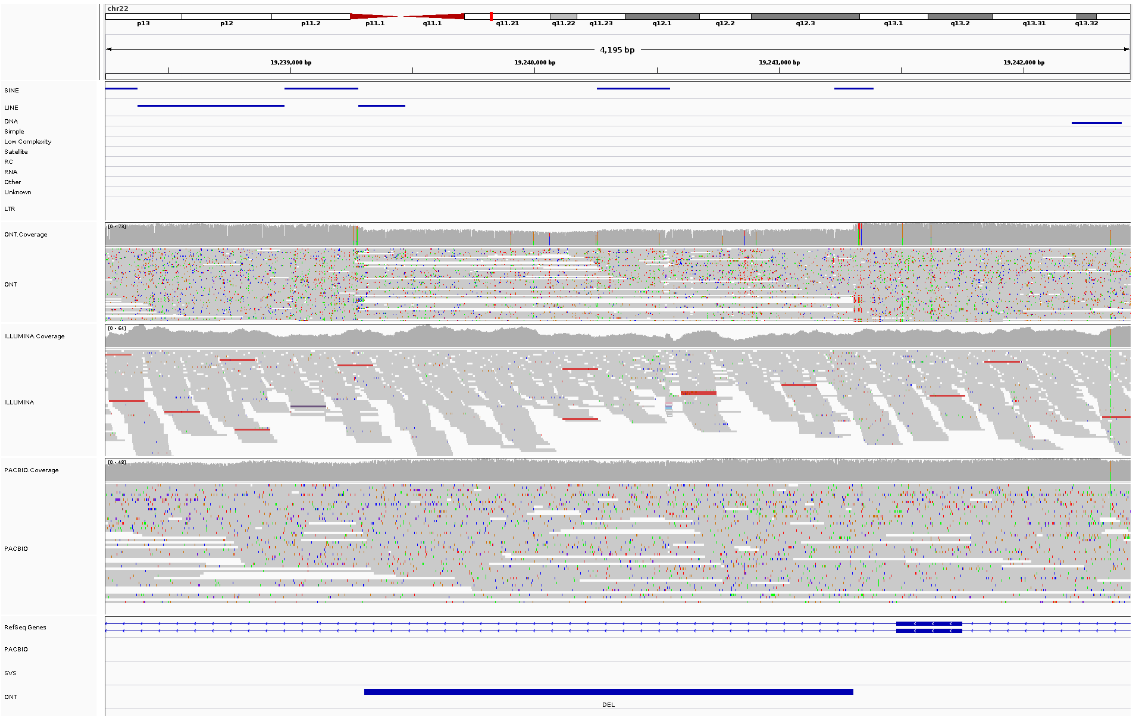

Supplementary Figure 21: Subfigure 4

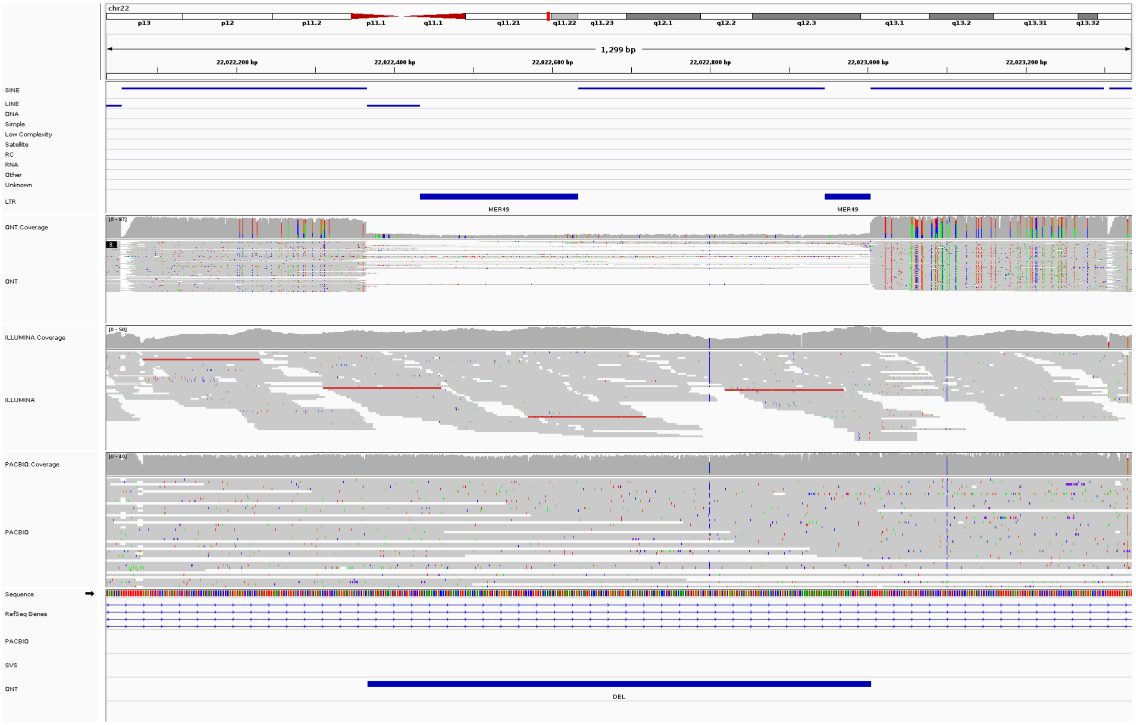

Supplementary Figure 21: Subfigure 5

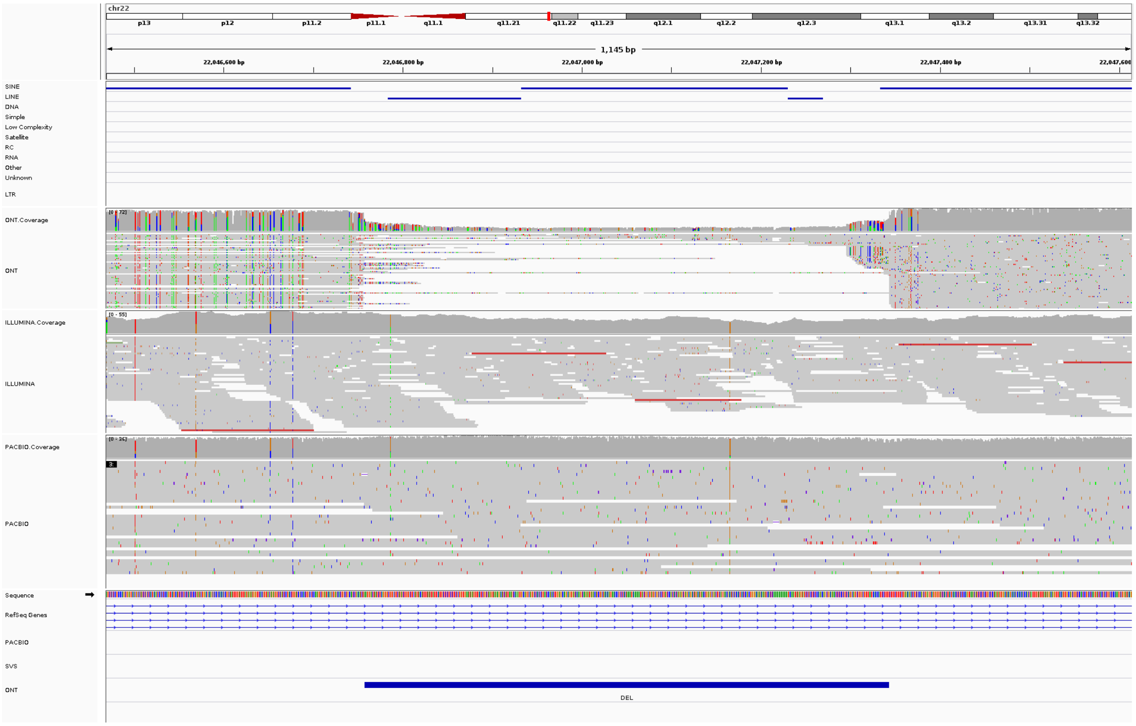

Supplementary Figure 21: Subfigure 6

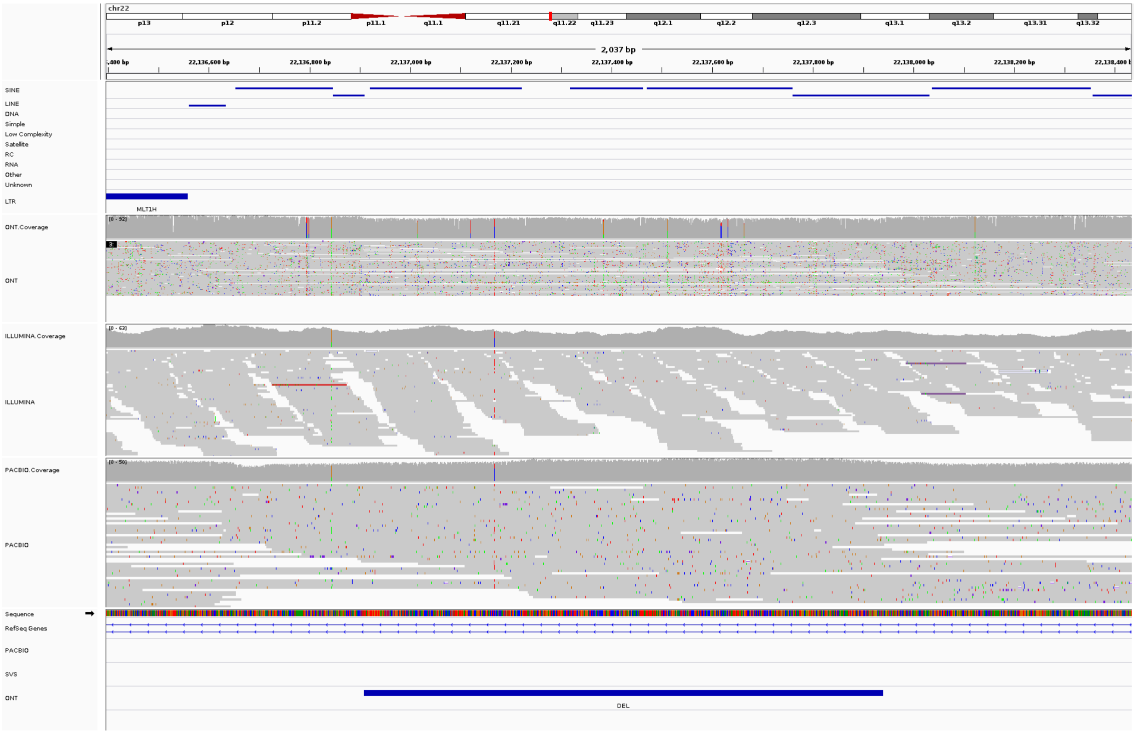

Supplementary Figure 21: Subfigure 7

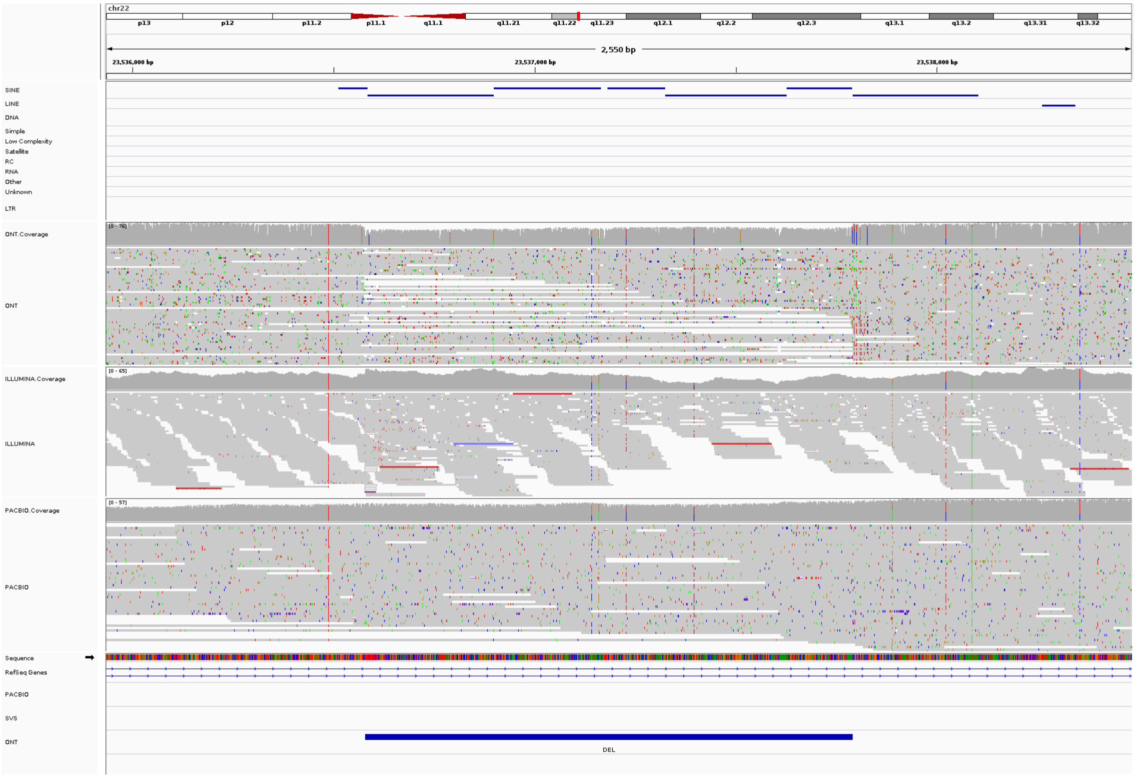

Supplementary Figure 21: Subfigure 8

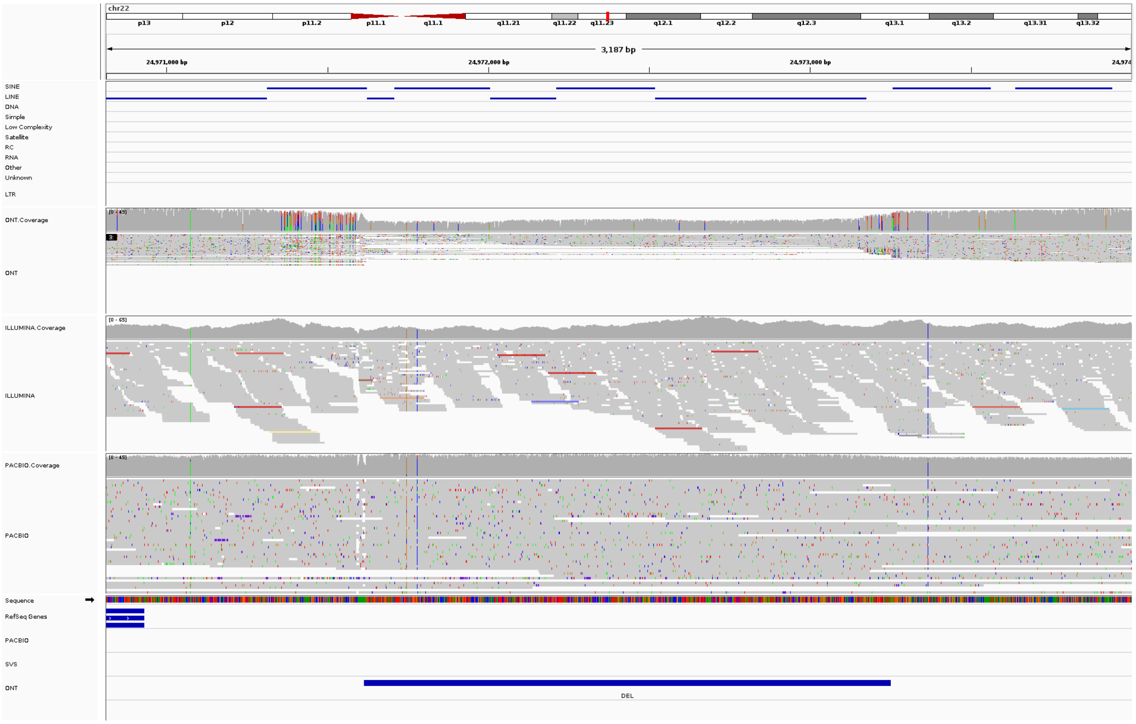

Supplementary Figure 21: Subfigure 9

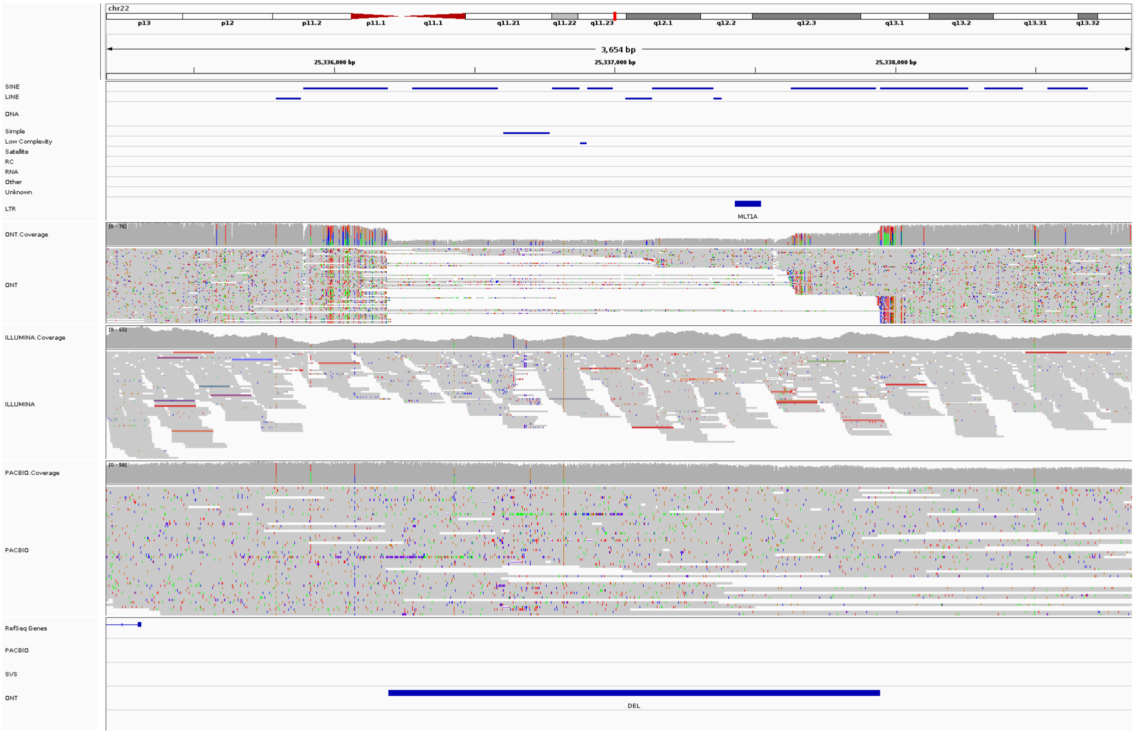

Supplementary Figure 21: Subfigure 10

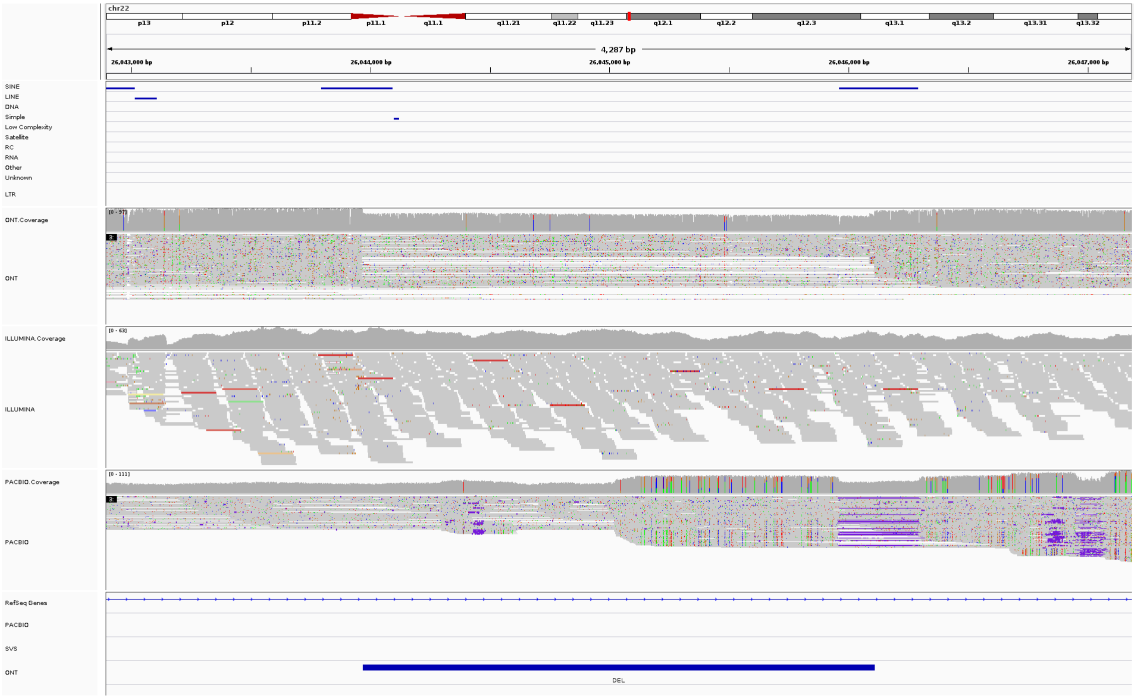

Supplementary Figure 21: Subfigure 11

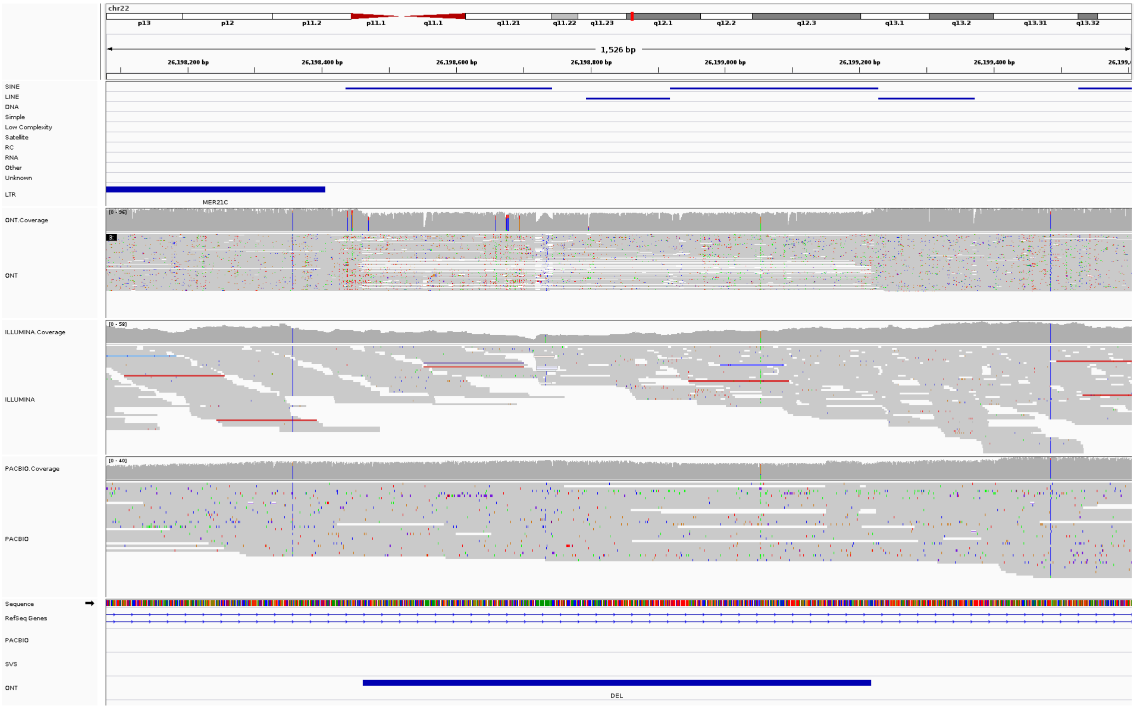

Supplementary Figure 21: Subfigure 12

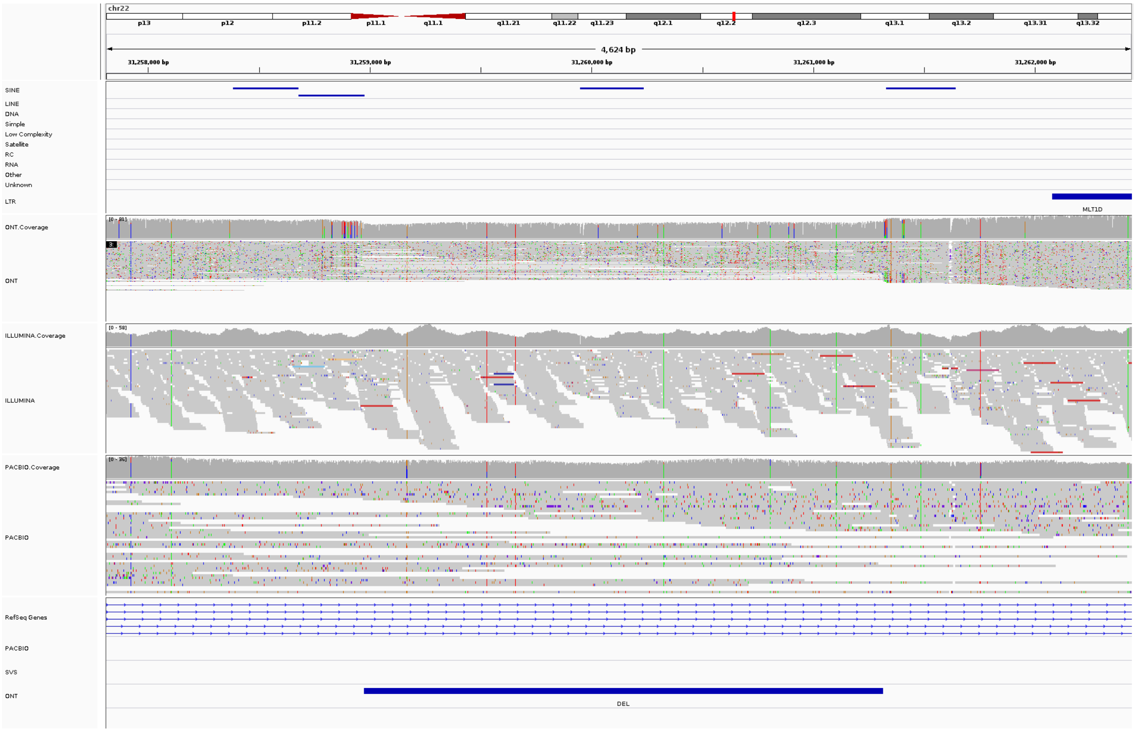

Supplementary Figure 21: Subfigure 13

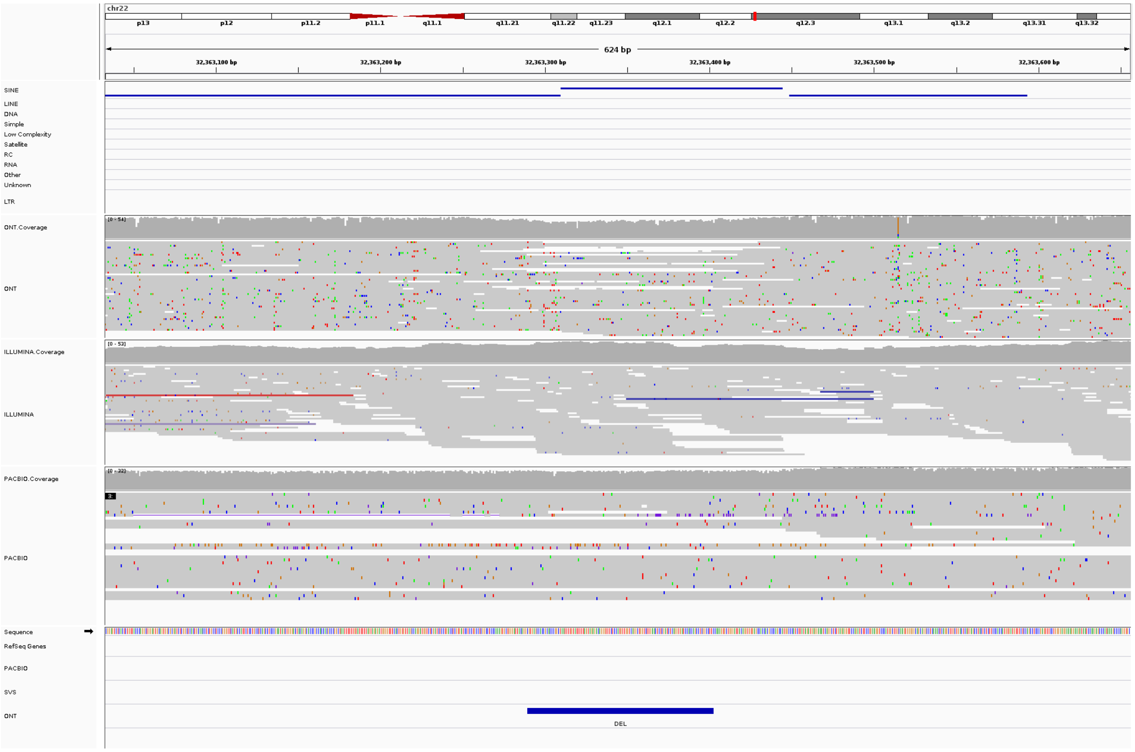

Supplementary Figure 21: Subfigure 14

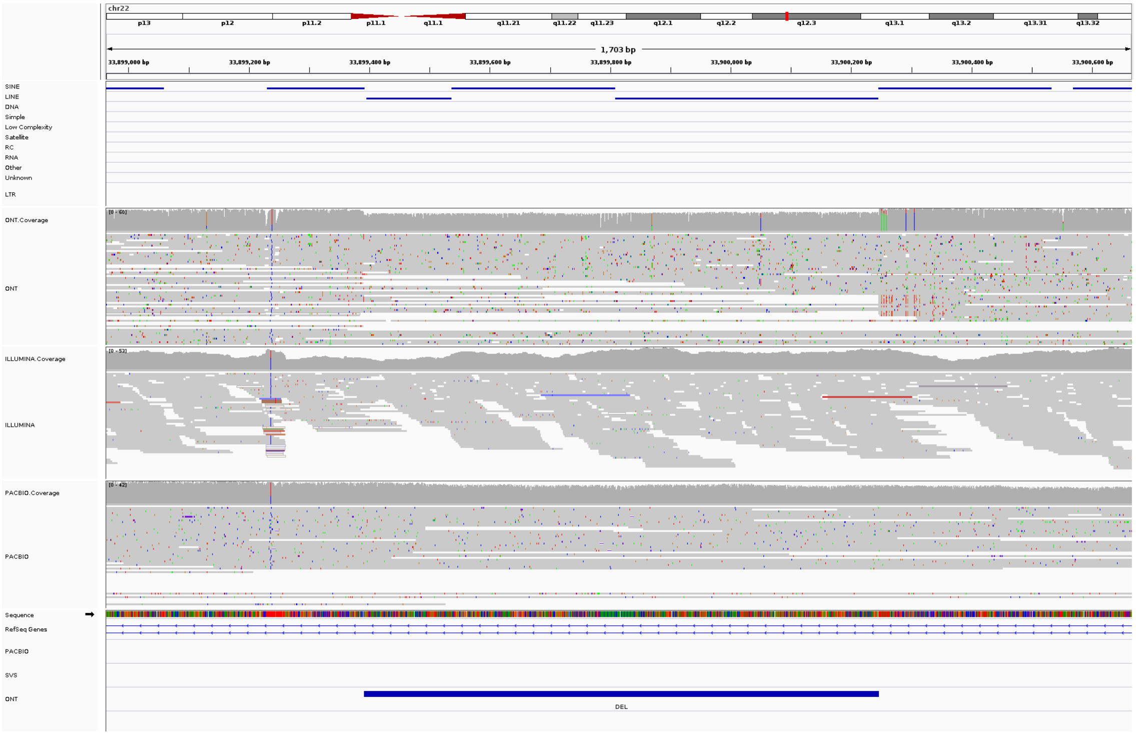

Supplementary Figure 21: Subfigure 15

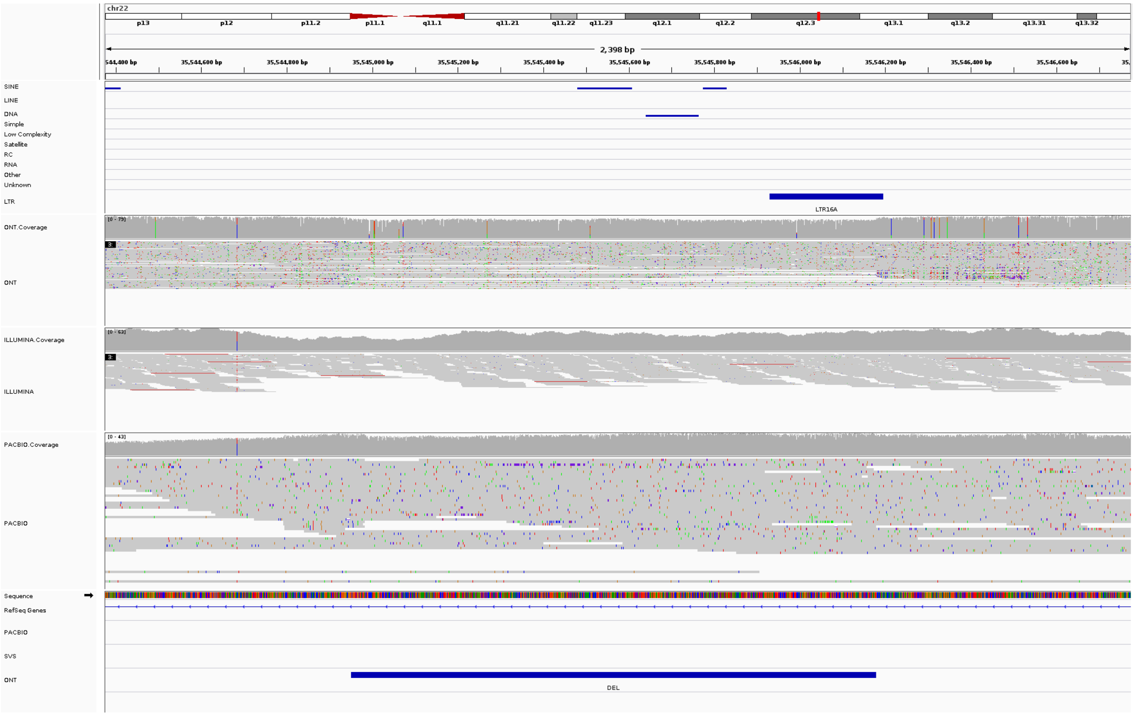

Supplementary Figure 21: Subfigure 16

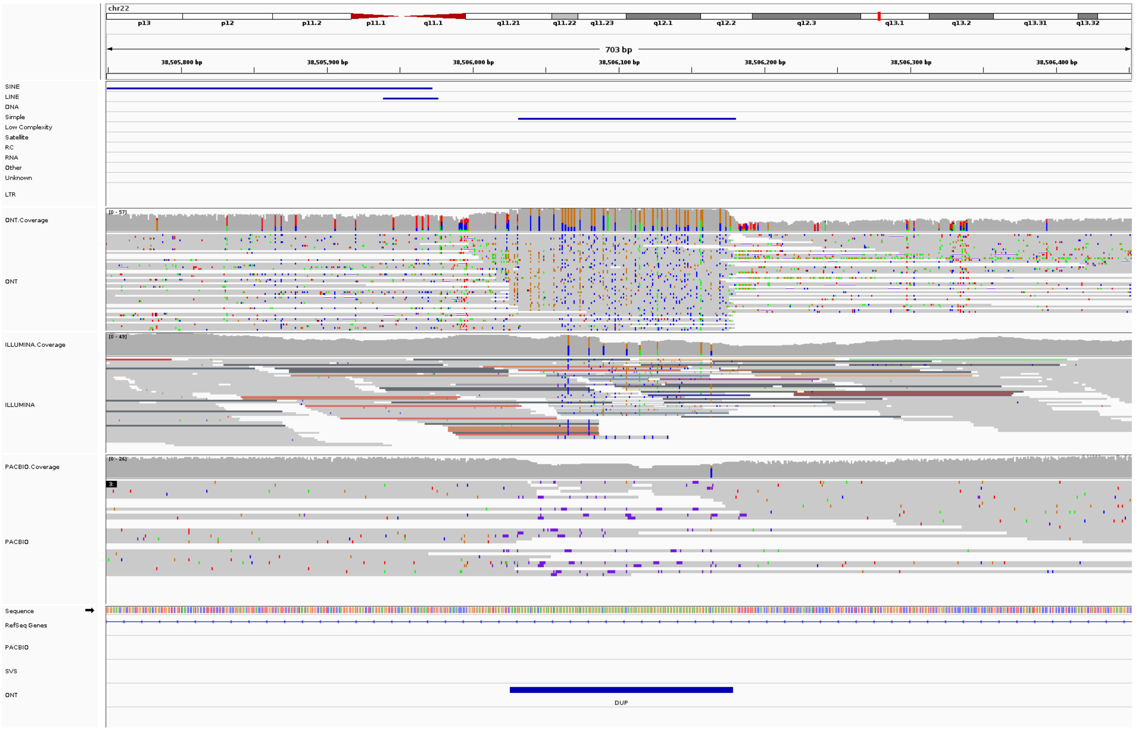

Supplementary Figure 21: Subfigure 17

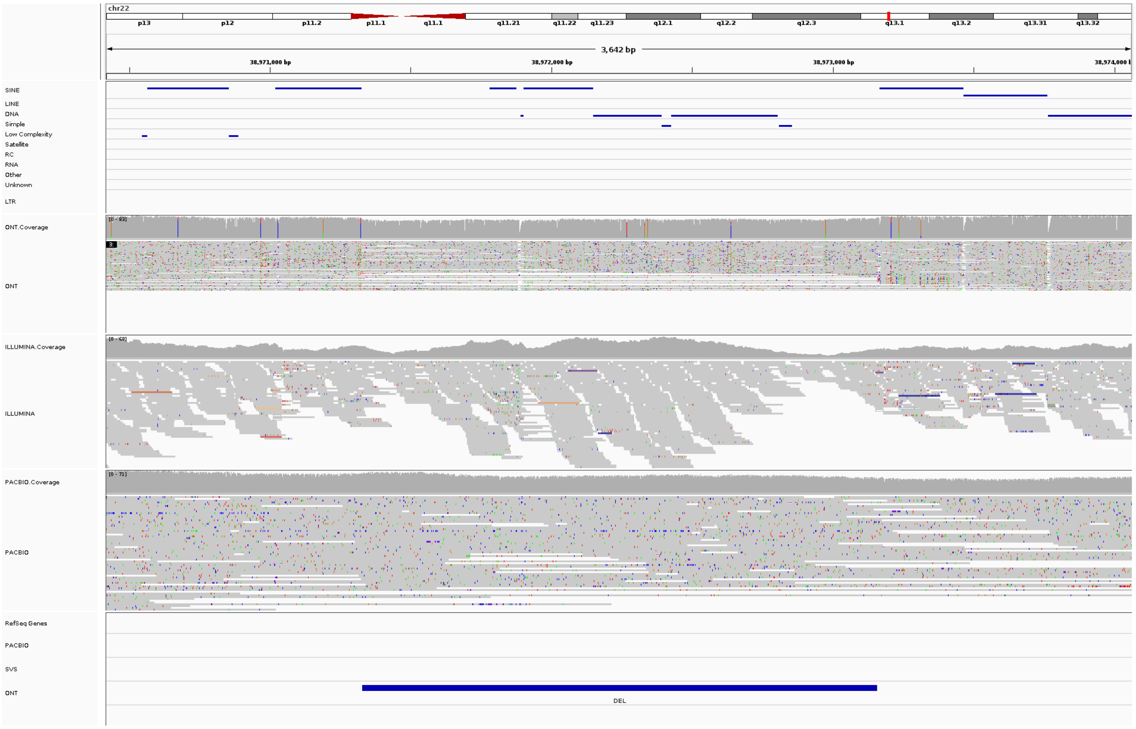

Supplementary Figure 21: Subfigure 18

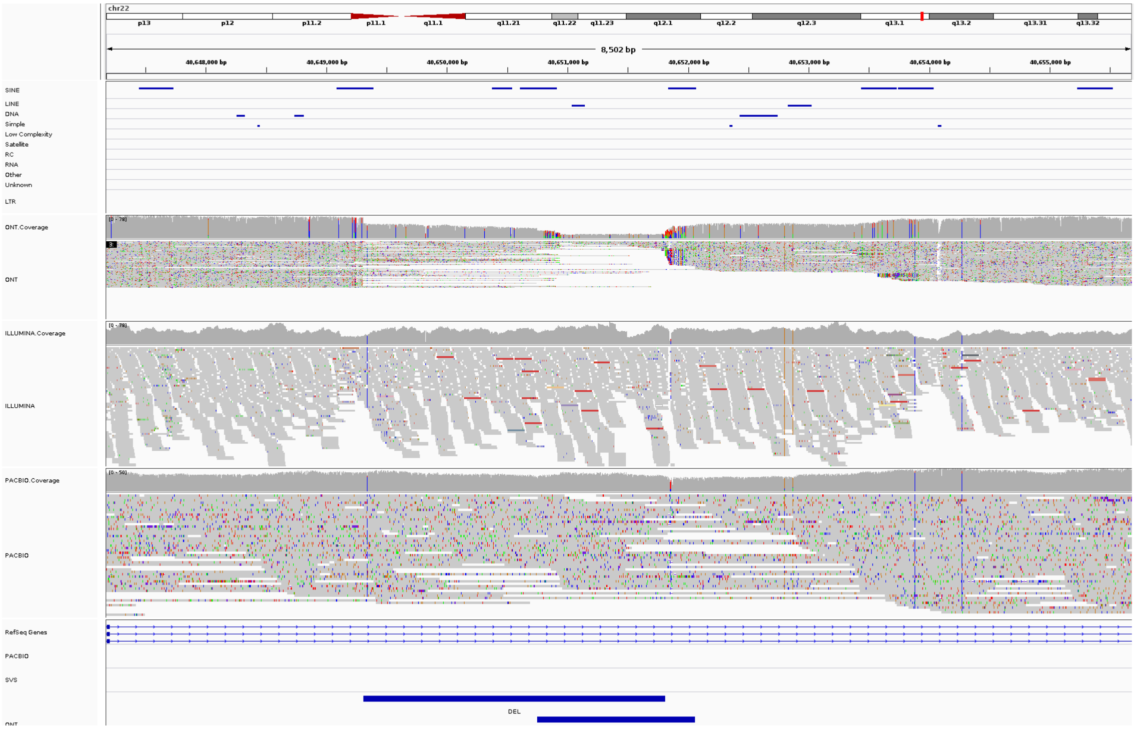

Supplementary Figure 21: Subfigure 19

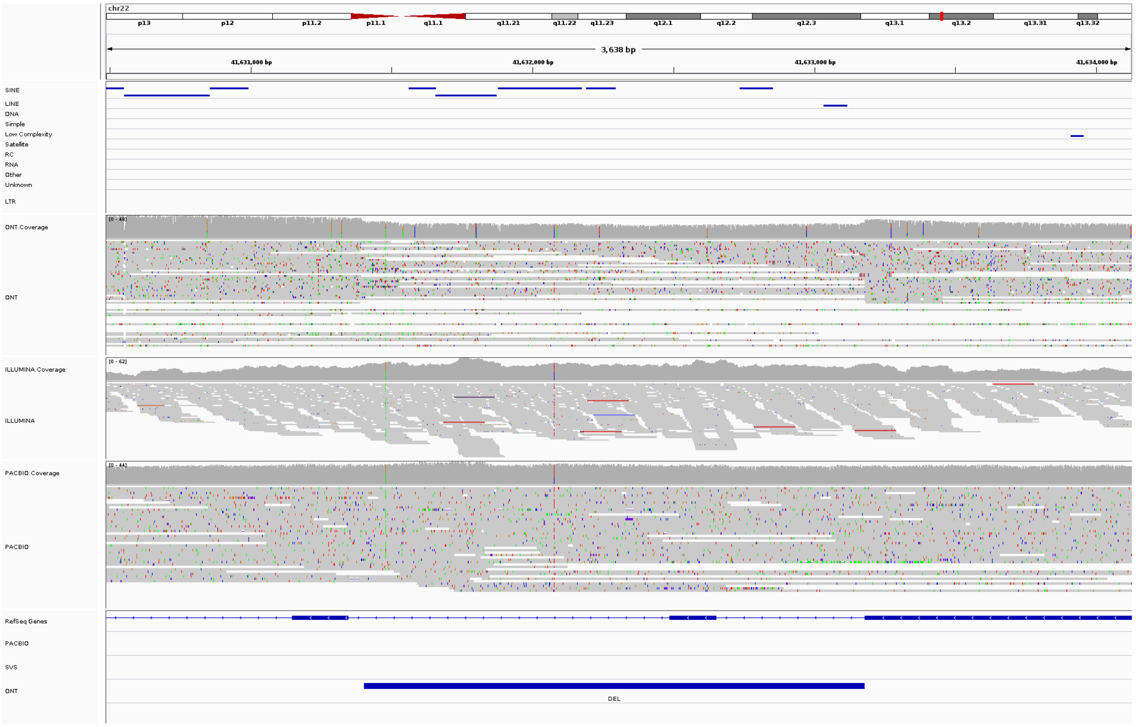

Supplementary Figure 21: Subfigure 20

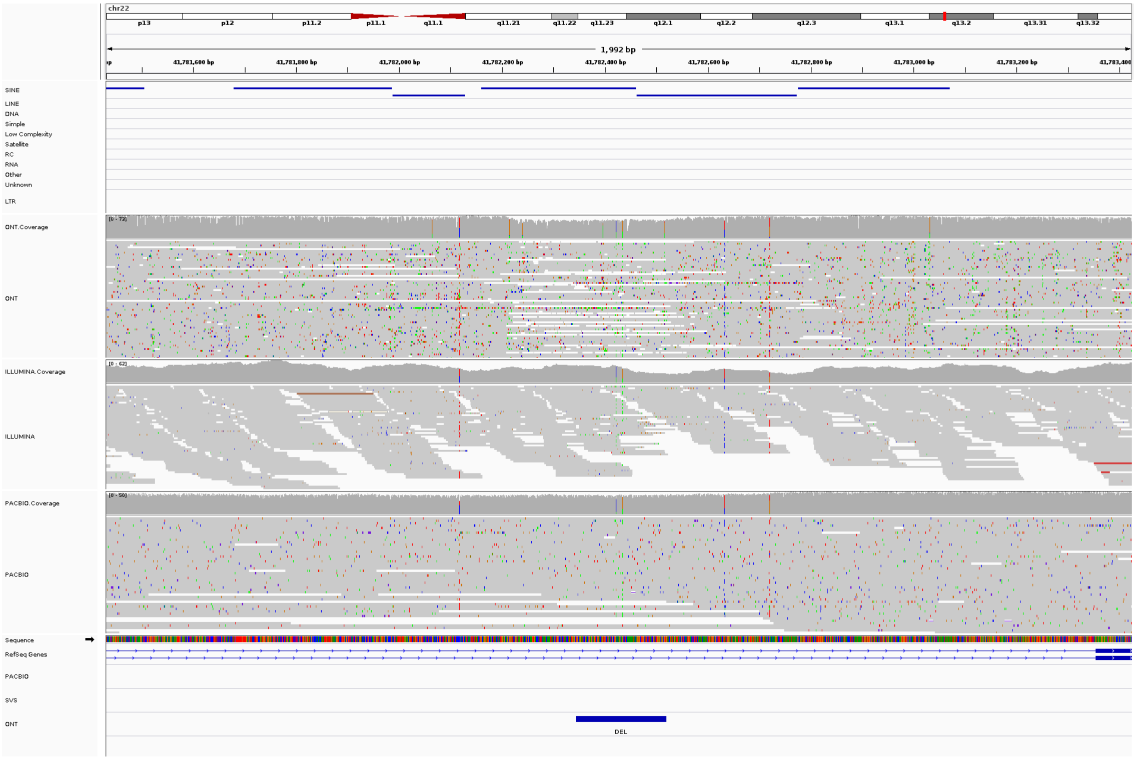

Supplementary Figure 21: Subfigure 21

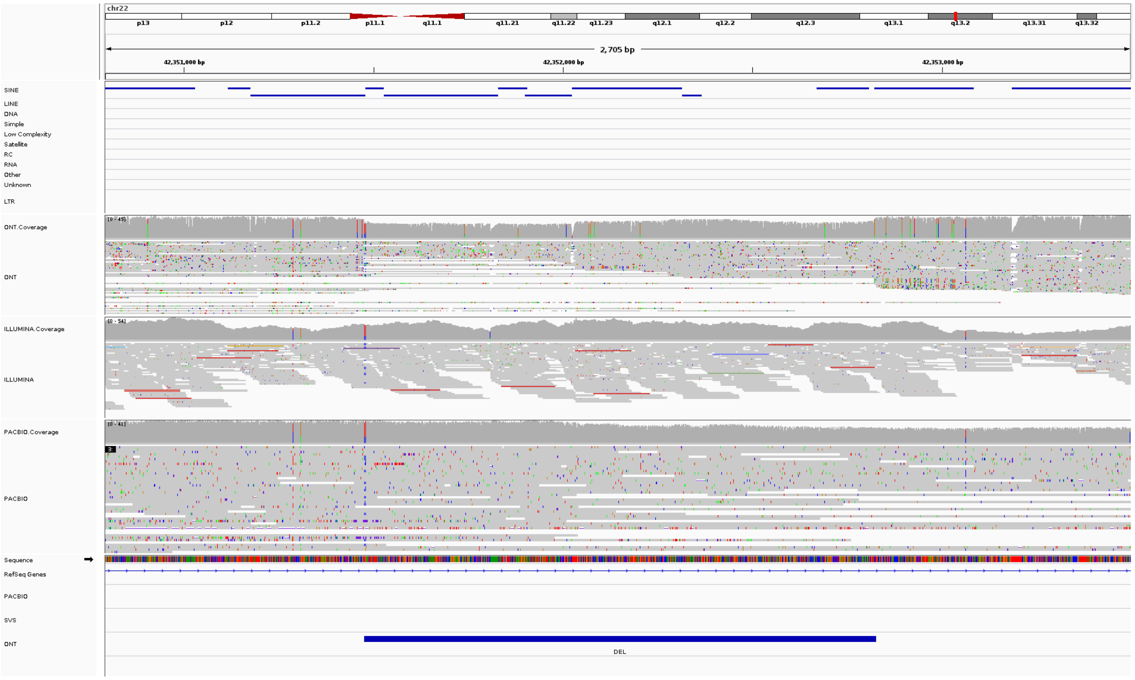

Supplementary Figure 21: Subfigure 22

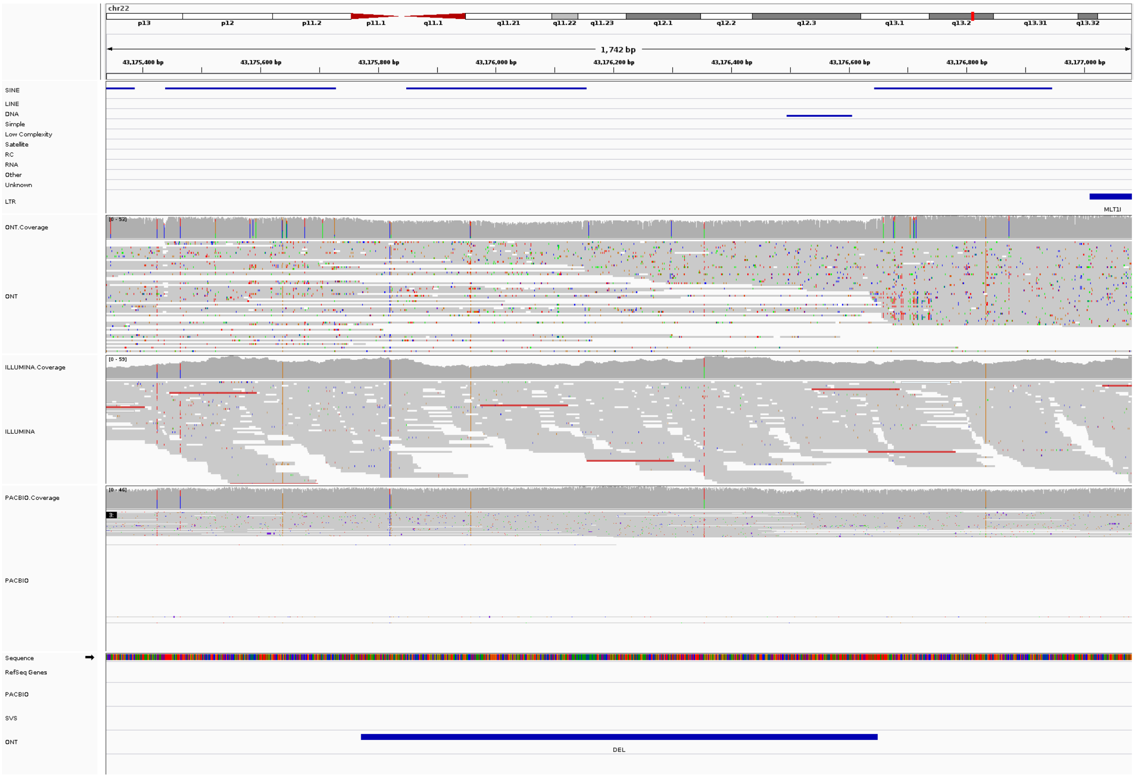

Supplementary Figure 21: Subfigure 23

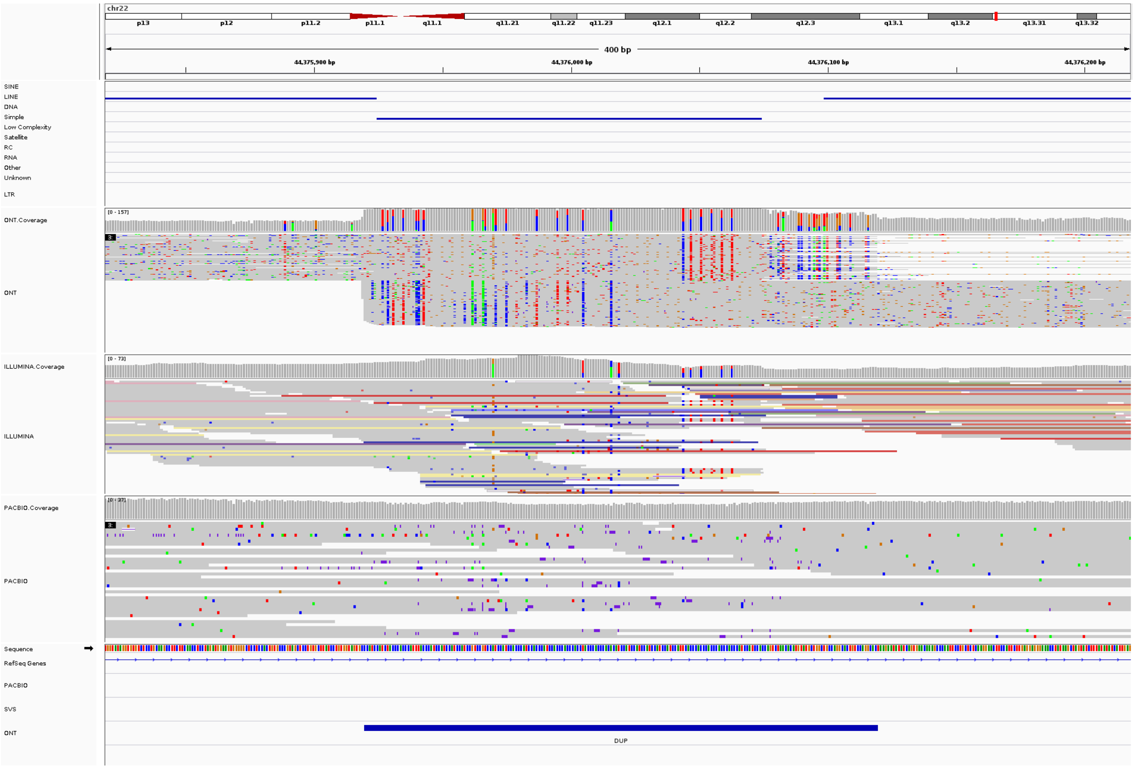

Supplementary Figure 21: Subfigure 24

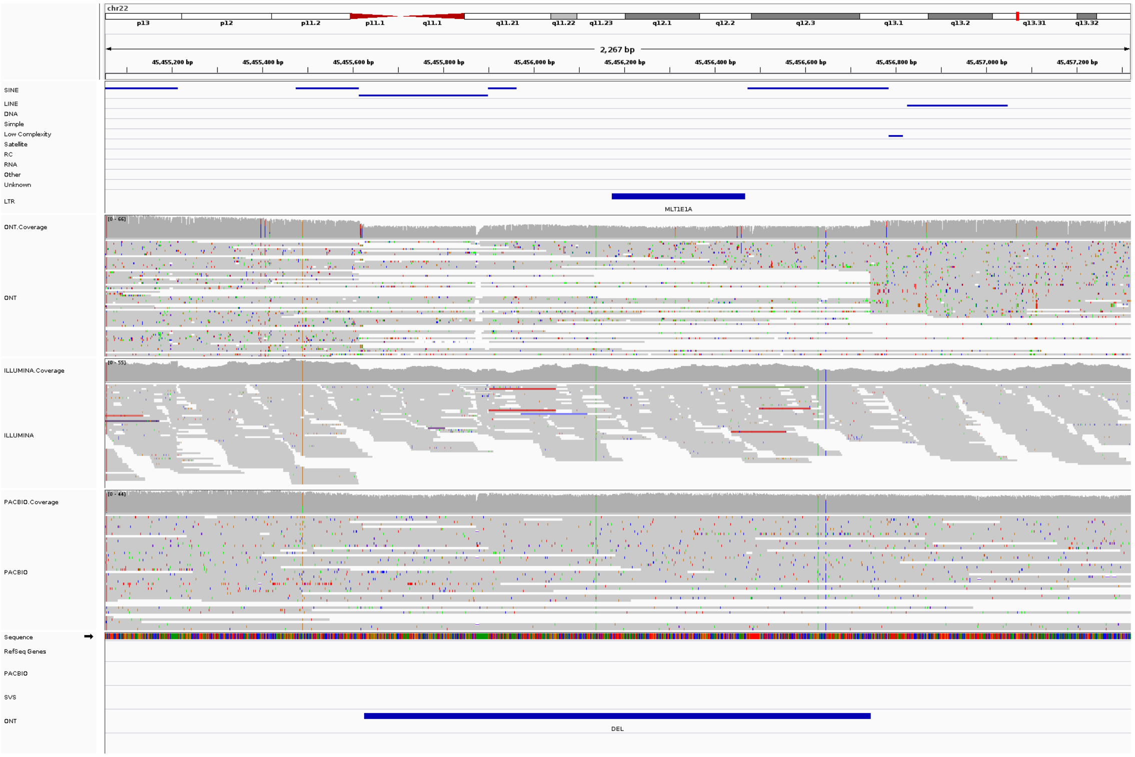

Supplementary Figure 21: Subfigure 25

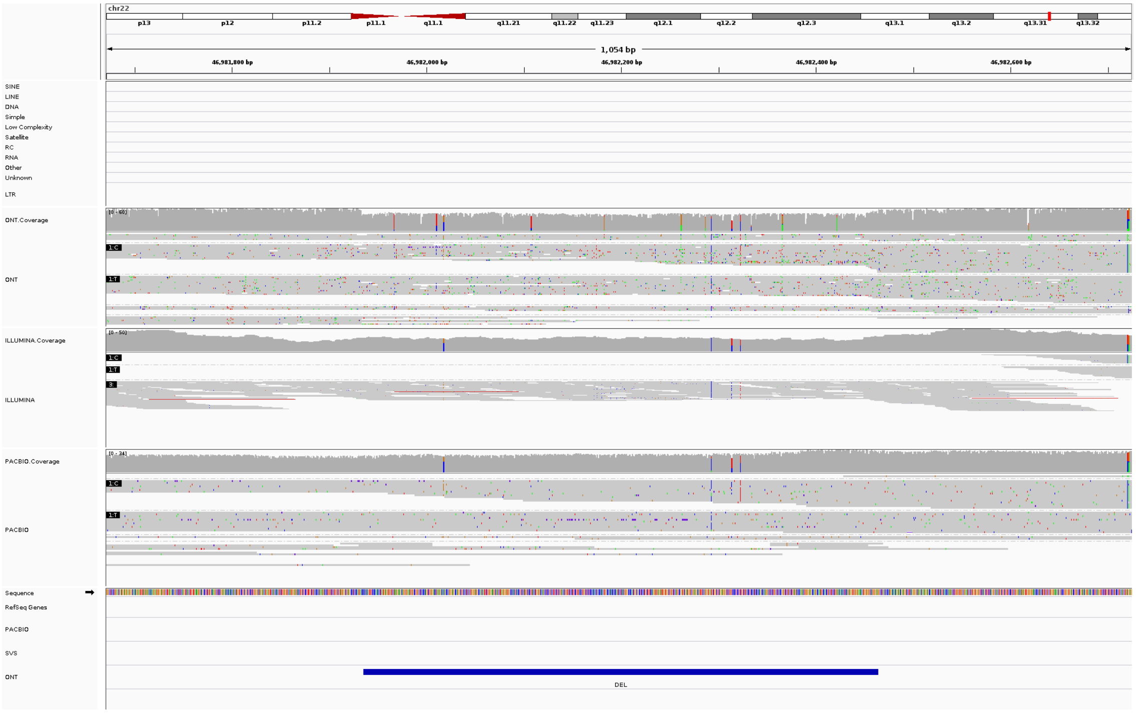

Supplementary Figure 21: Subfigure 26

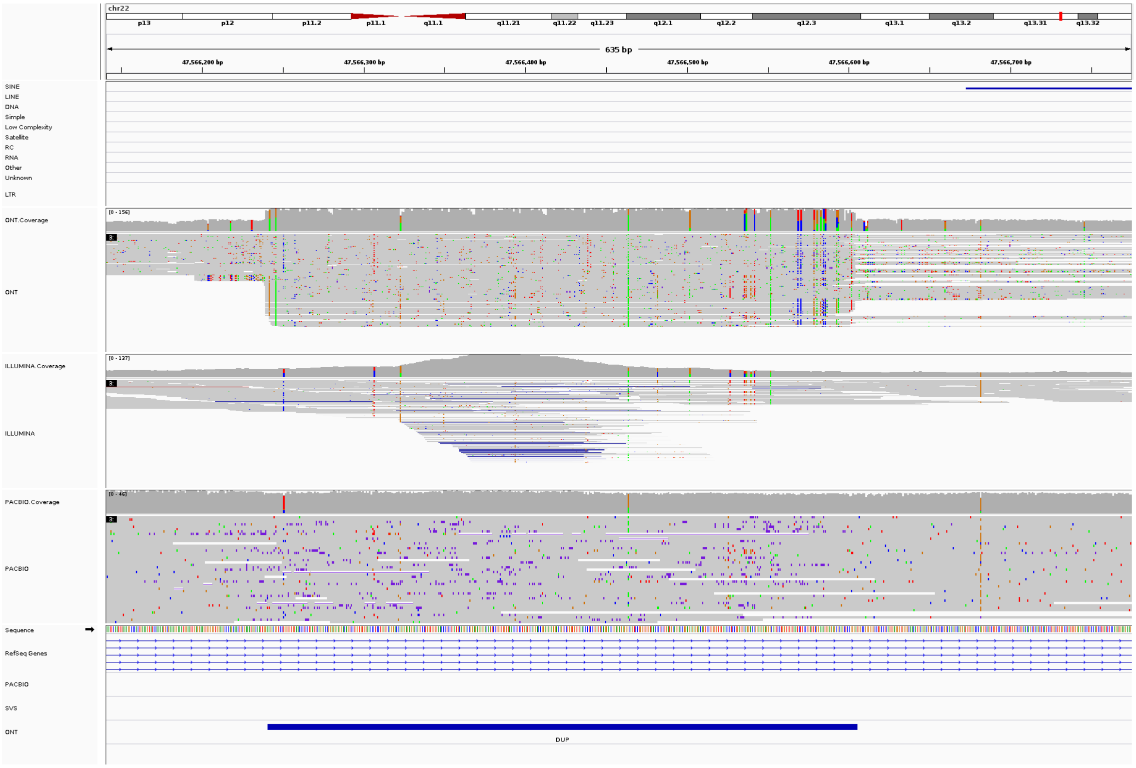

Supplementary Figure 21: Subfigure 27

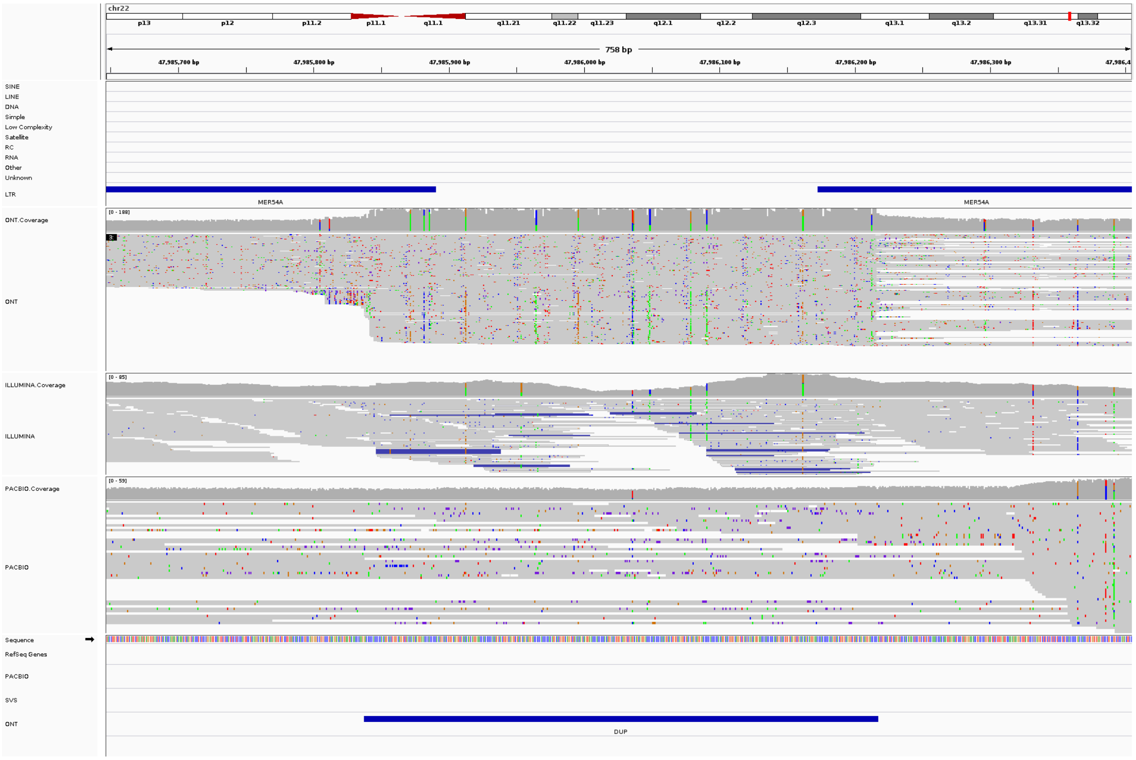

Supplementary Figure 21: Subfigure 28

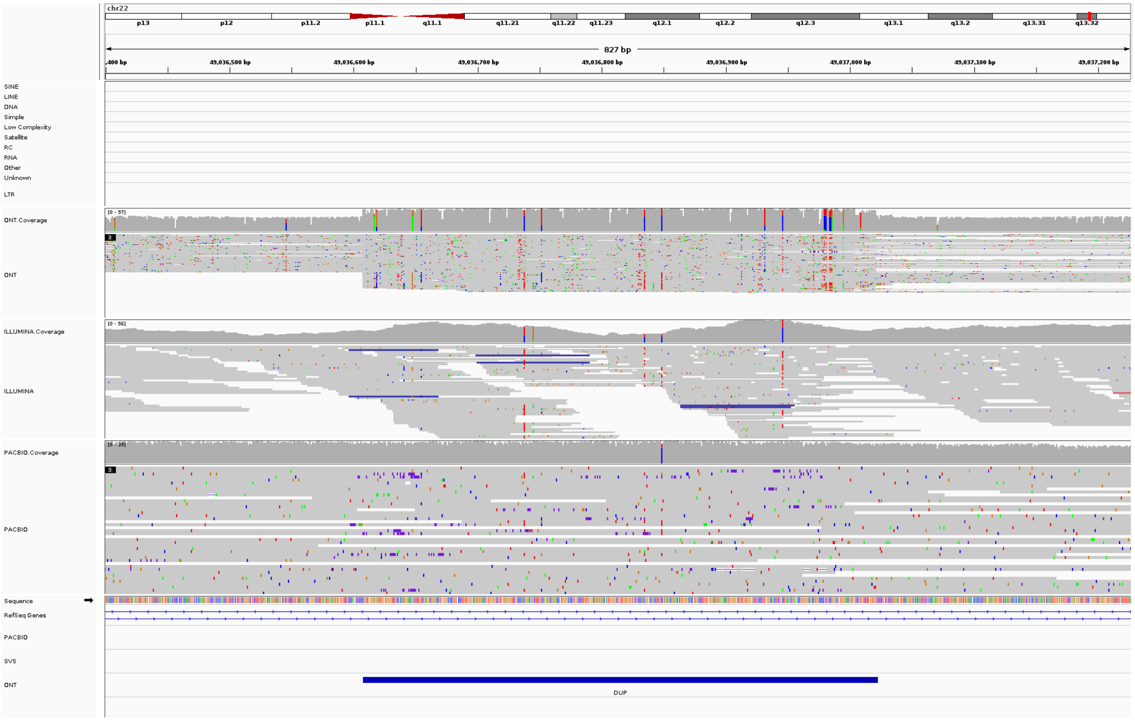

Supplementary Figure 21: Subfigure 29

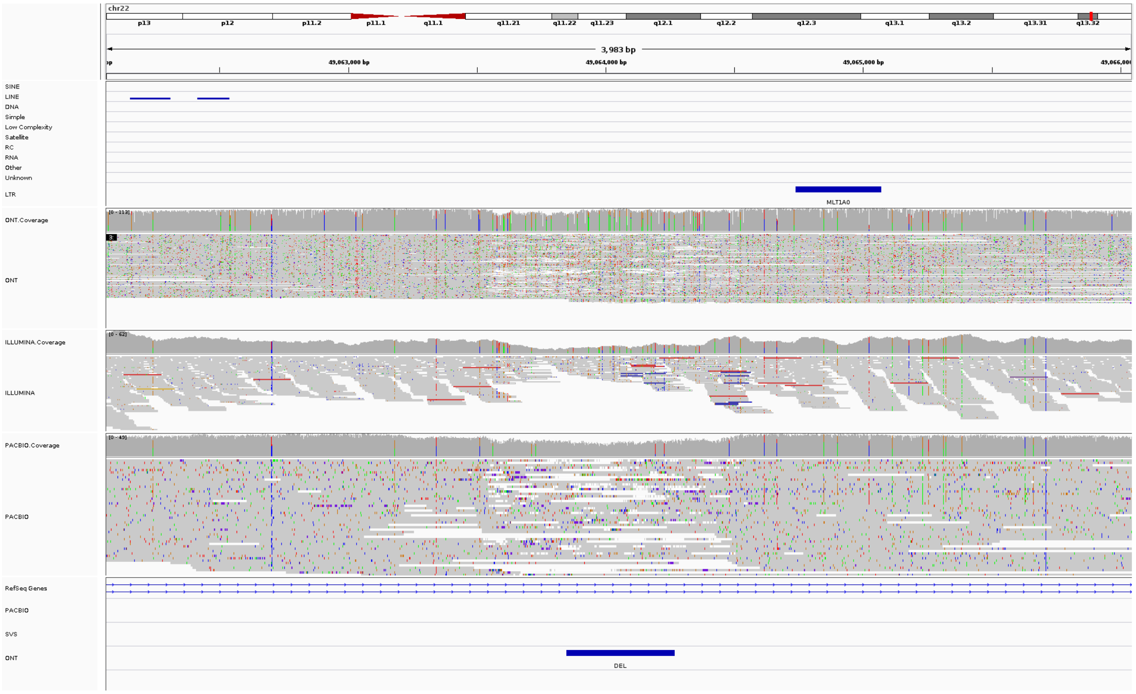

Supplementary Figure 21: Subfigure 30

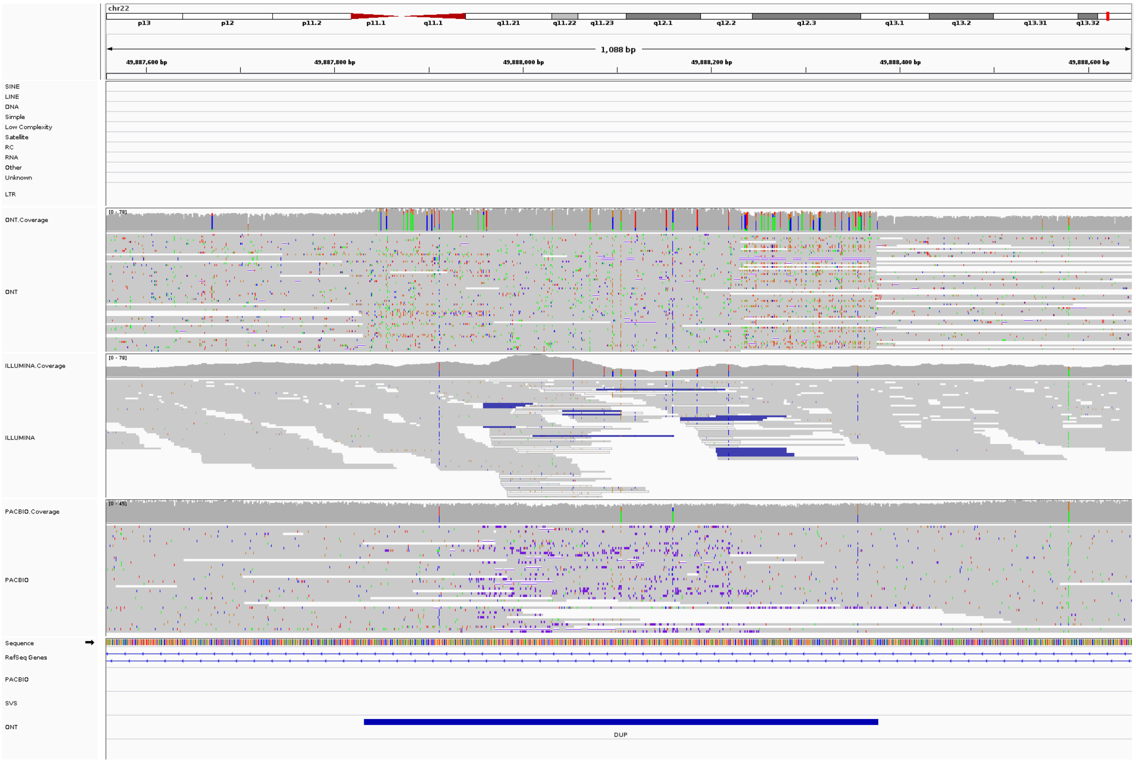

Supplementary Figure 21: Subfigure 31

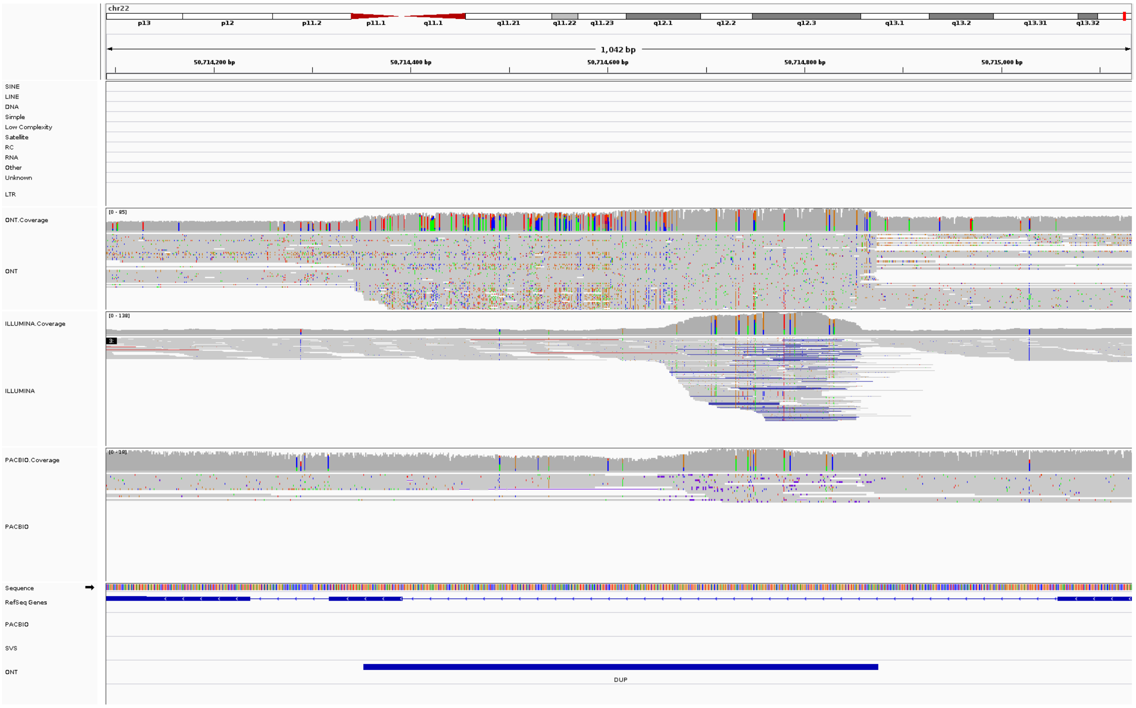

3.2.2 Large variant calls in ONT data not present in reference - duplications

This section shows large variant calls based on ONT data, which are not present in reference data set and have no or weak evidence in PACBIO data. Based on visual inspection we believe these calls to be true positives. The plots here show for duplicated regions a modified snapshot from the previous figure with indels smaller than 5 bases ignored and insertions marked by the length of the inserted sequence.

Supplementary Figure 22: Subfigure 1

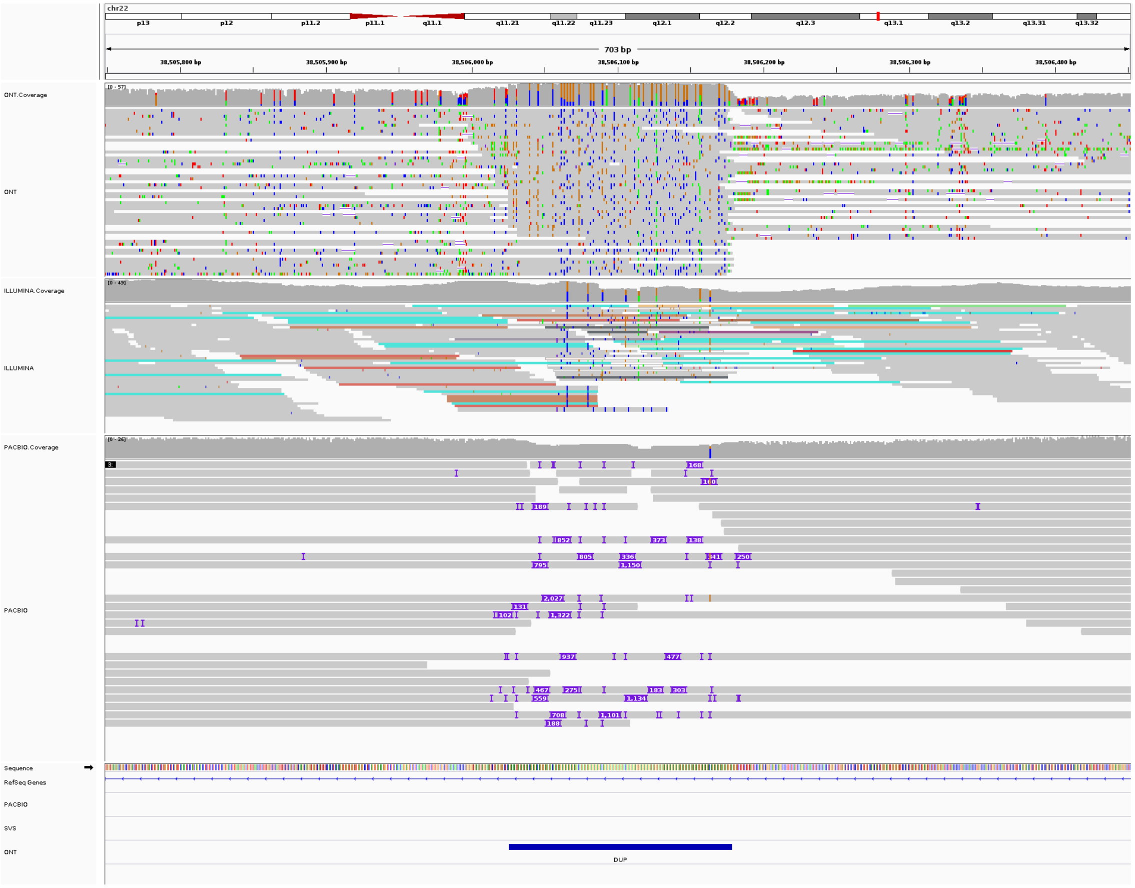

Supplementary Figure 22: Subfigure 2

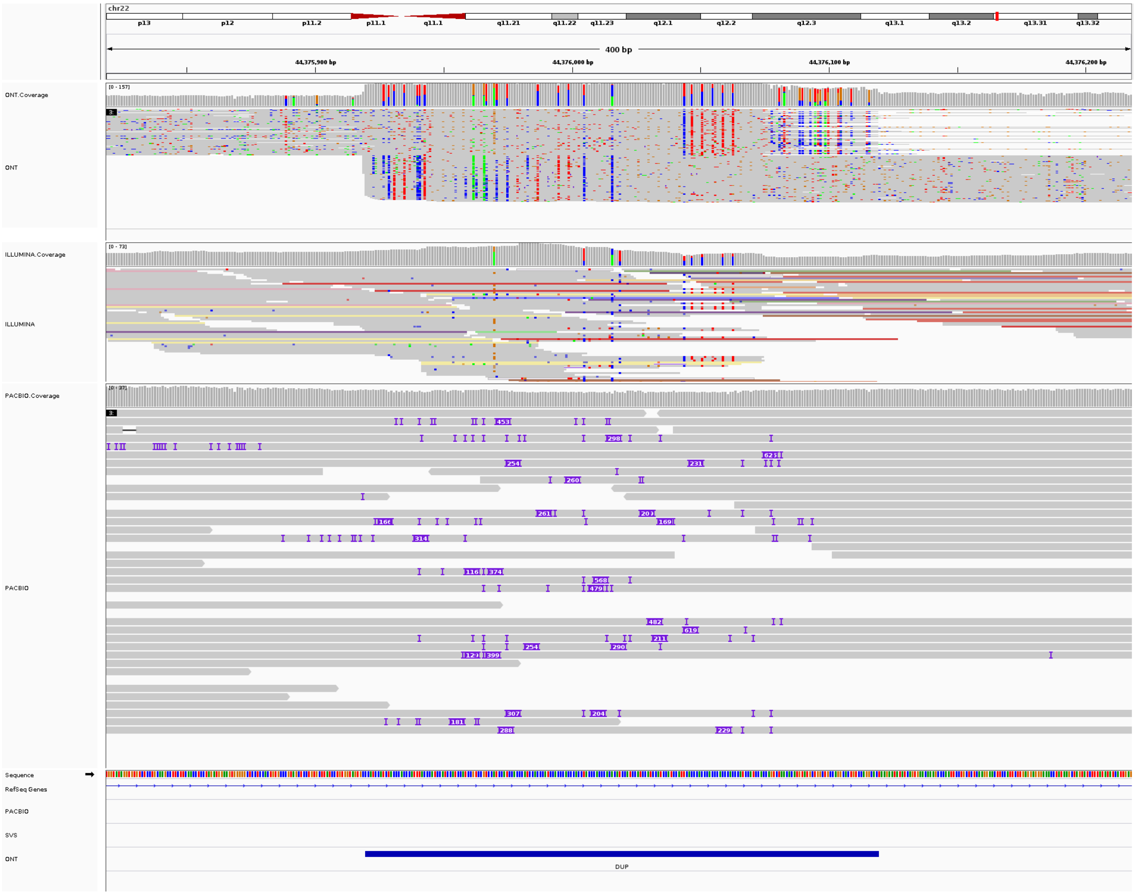

Supplementary Figure 22: Subfigure 3

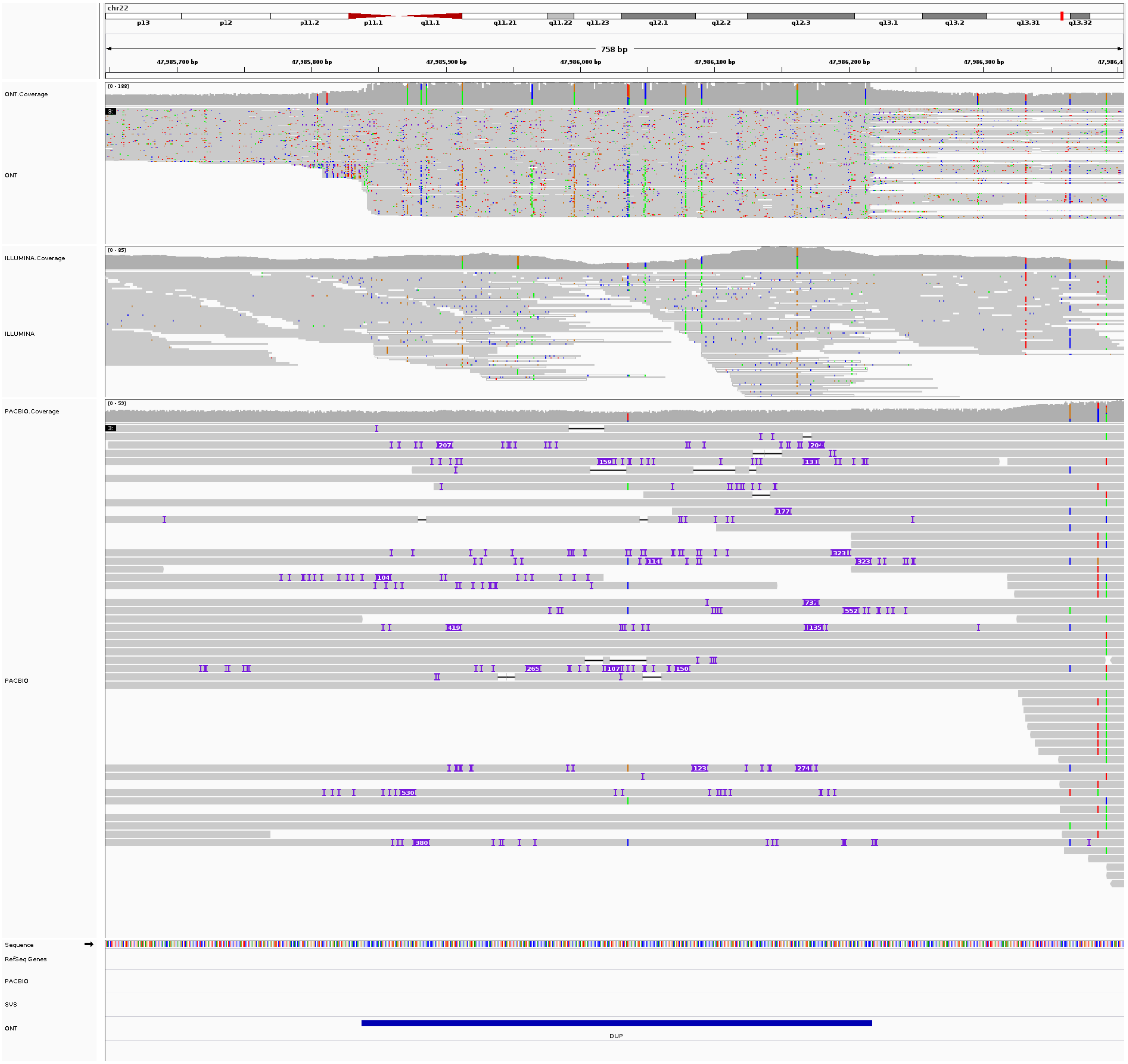

Supplementary Figure 22: Subfigure 4

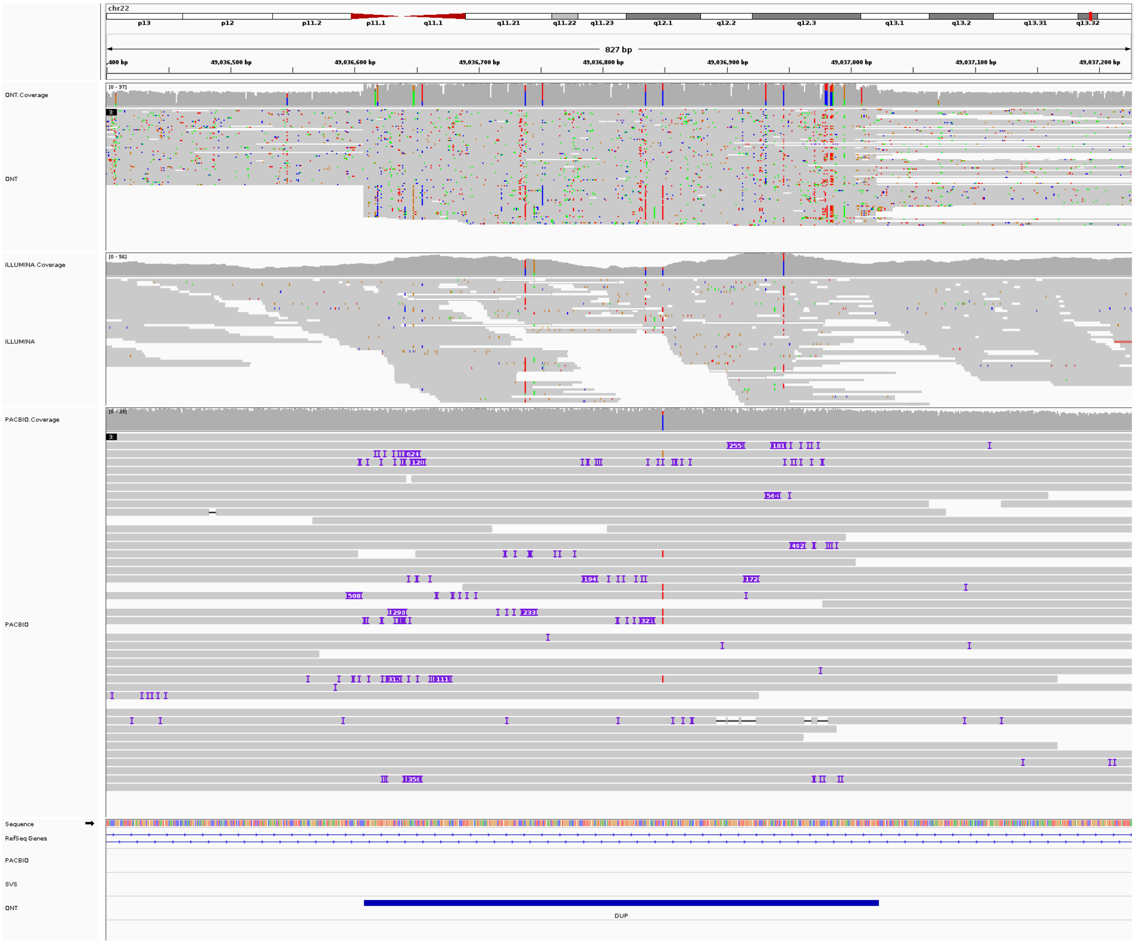

Supplementary Figure 22: Subfigure 5

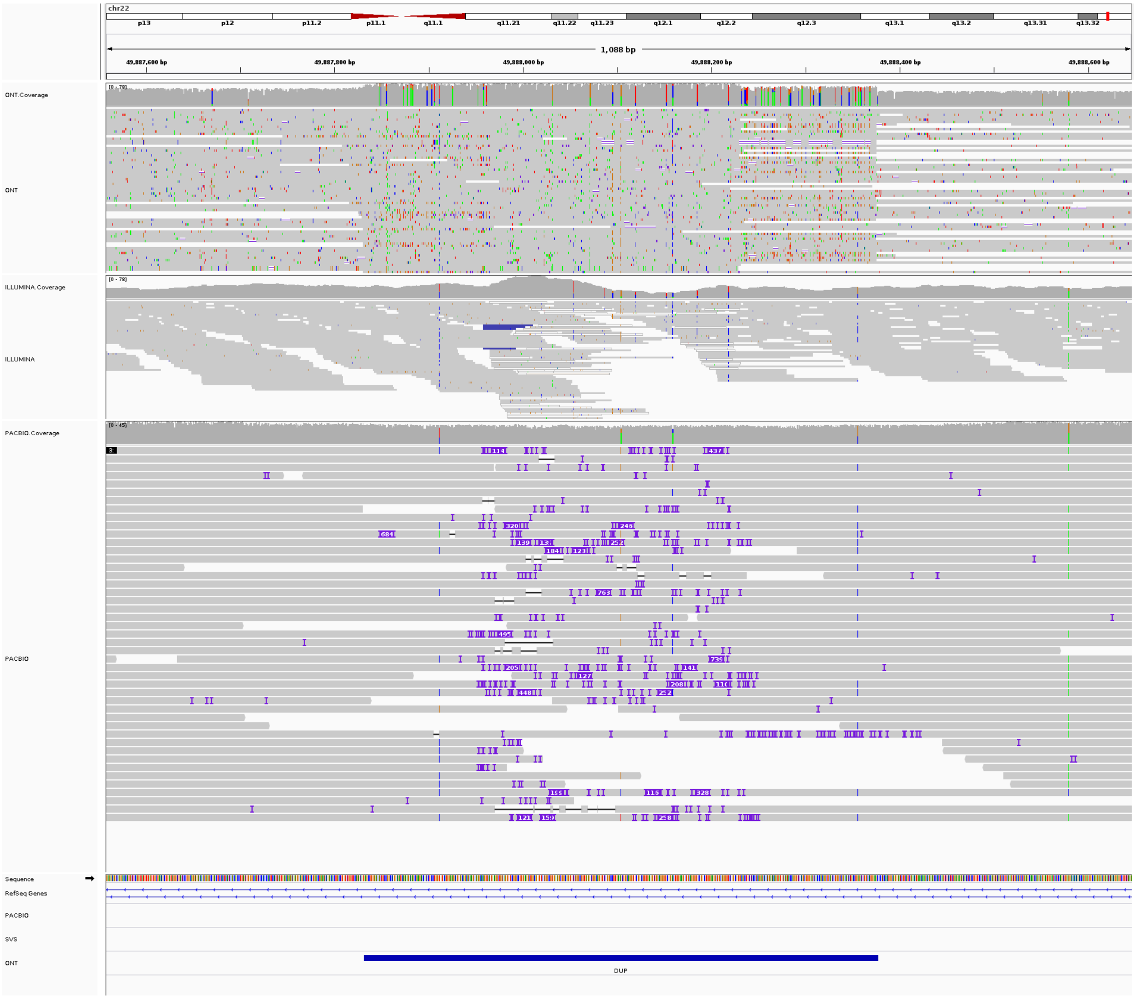

Supplementary Figure 22: Subfigure 6

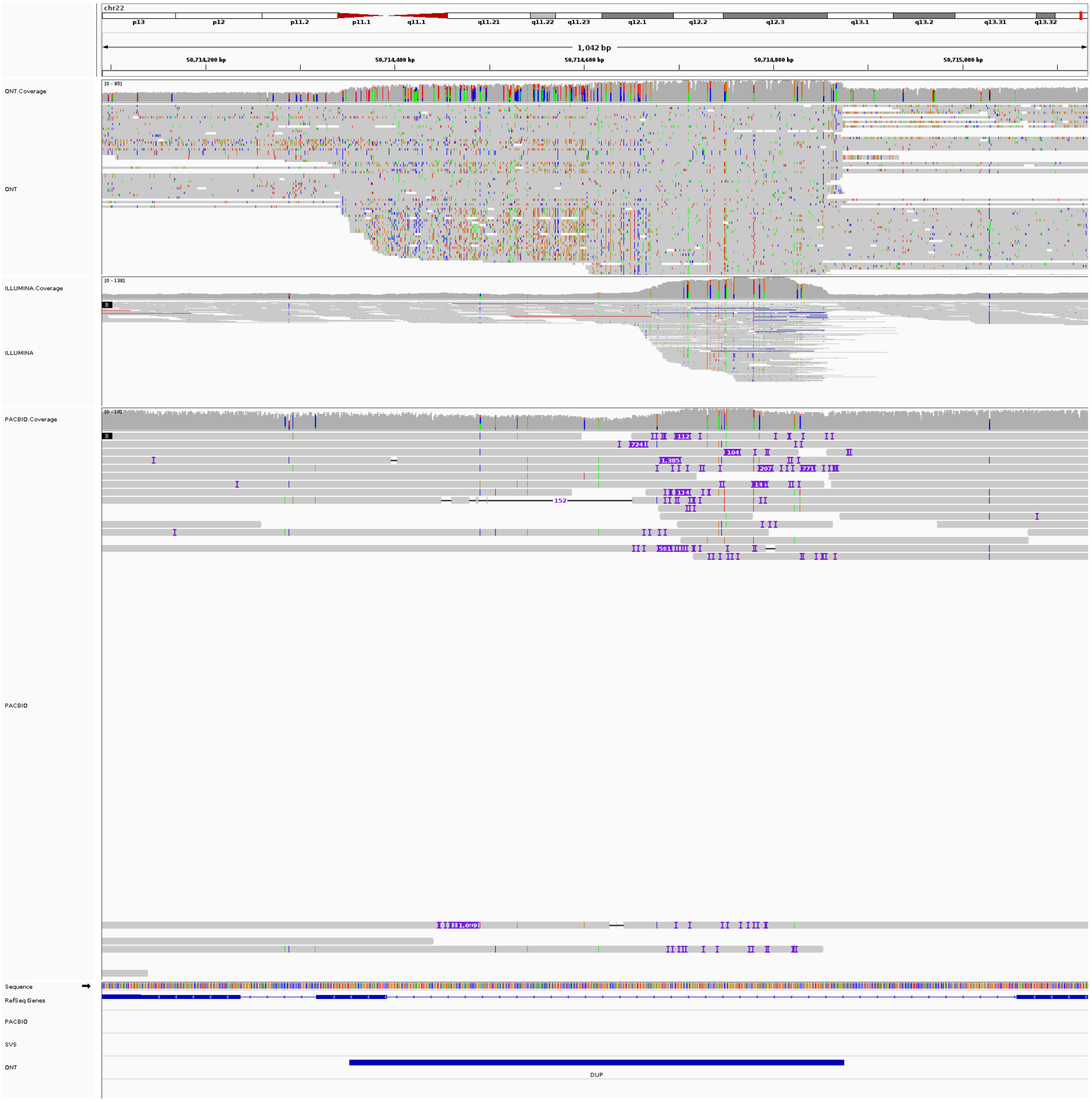

3.2.3 Large variant calls in reference data not called, but with ONT data support

This section shows large variant calls that are present in the reference data set, but which have not been called using our method and have support in the ONT data. Based on visual inspection we believe these calls to be false negatives.

Supplementary Figure 23: Subfigure 1

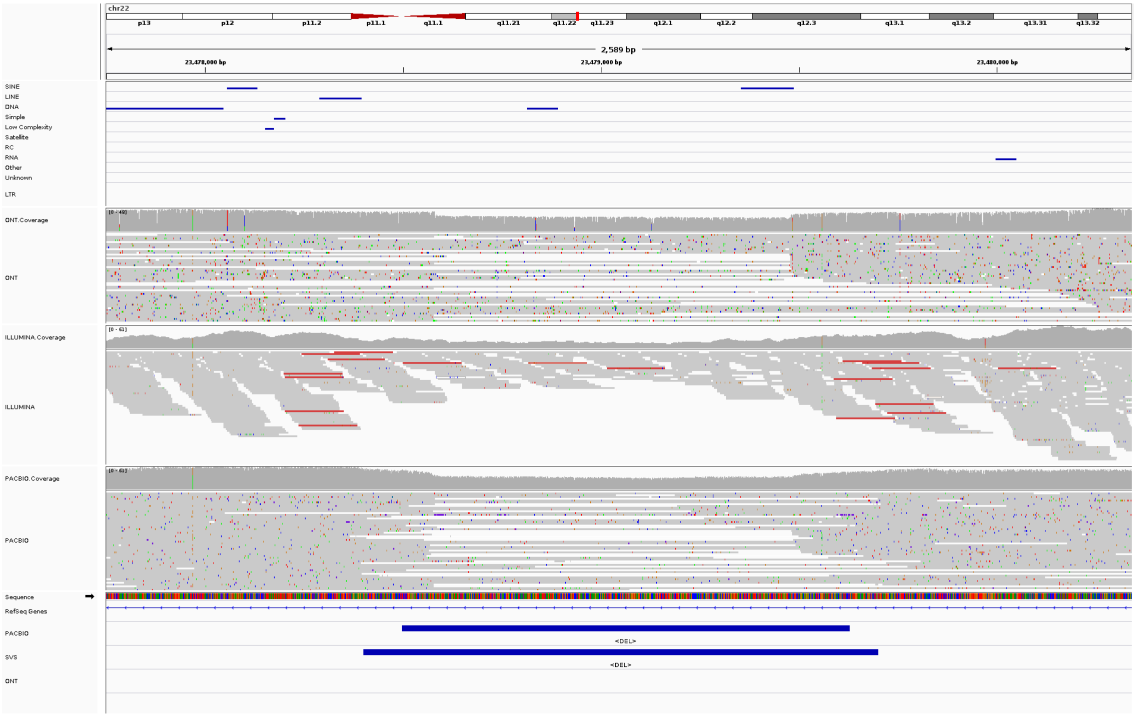

Supplementary Figure 23: Subfigure 2

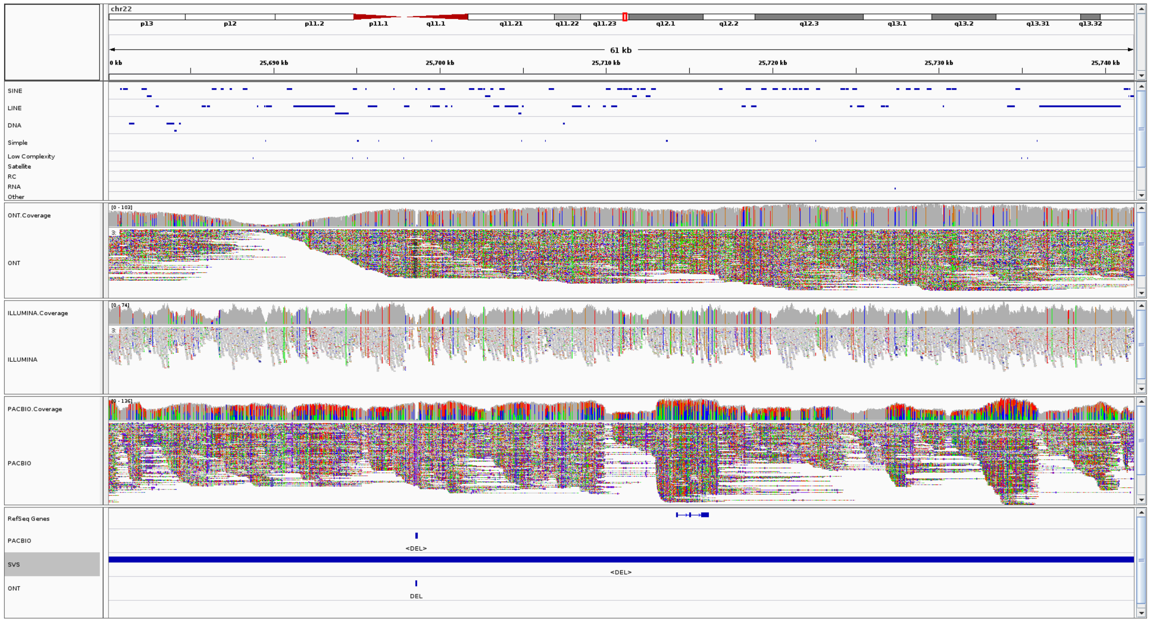

Supplementary Figure 23: Subfigure 3

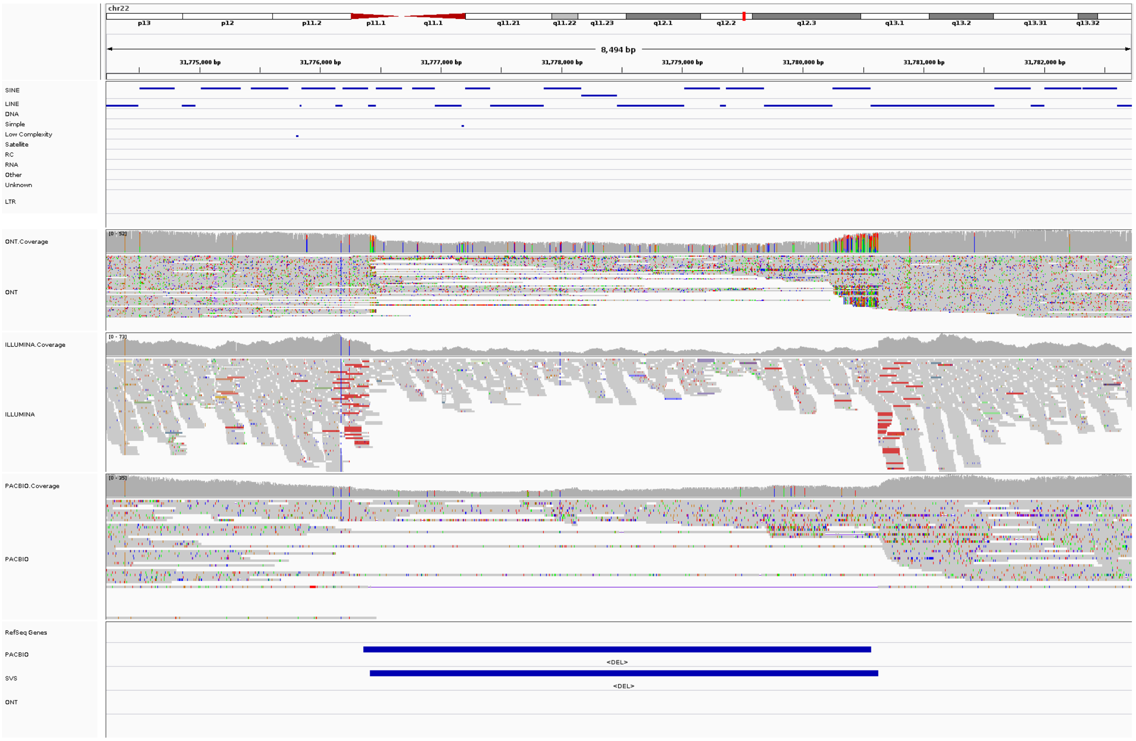

Supplementary Figure 23: Subfigure 4

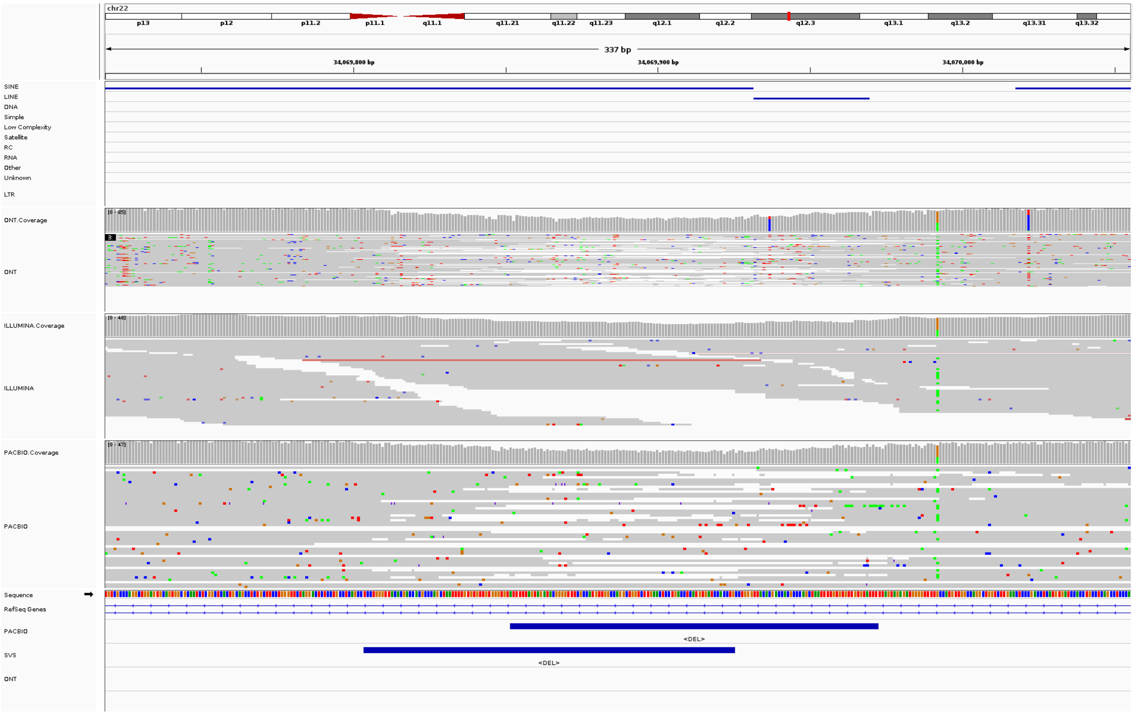

Supplementary Figure 23: Subfigure 5

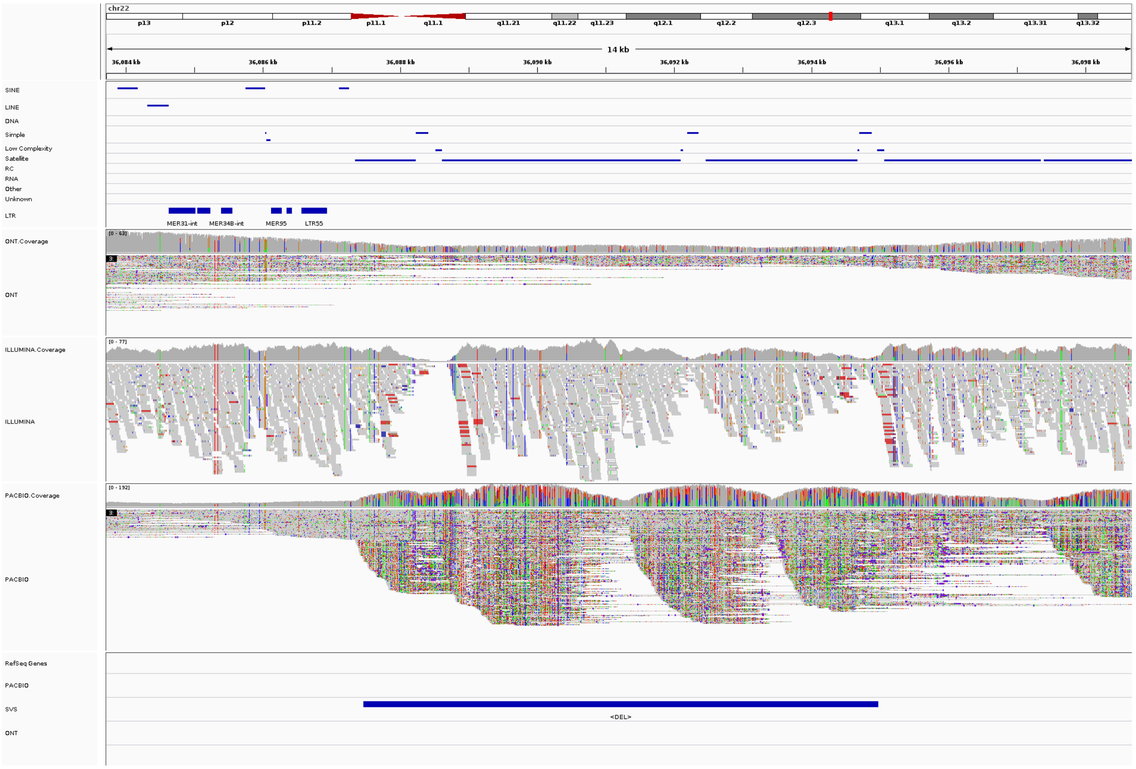

Supplementary Figure 23: Subfigure 6

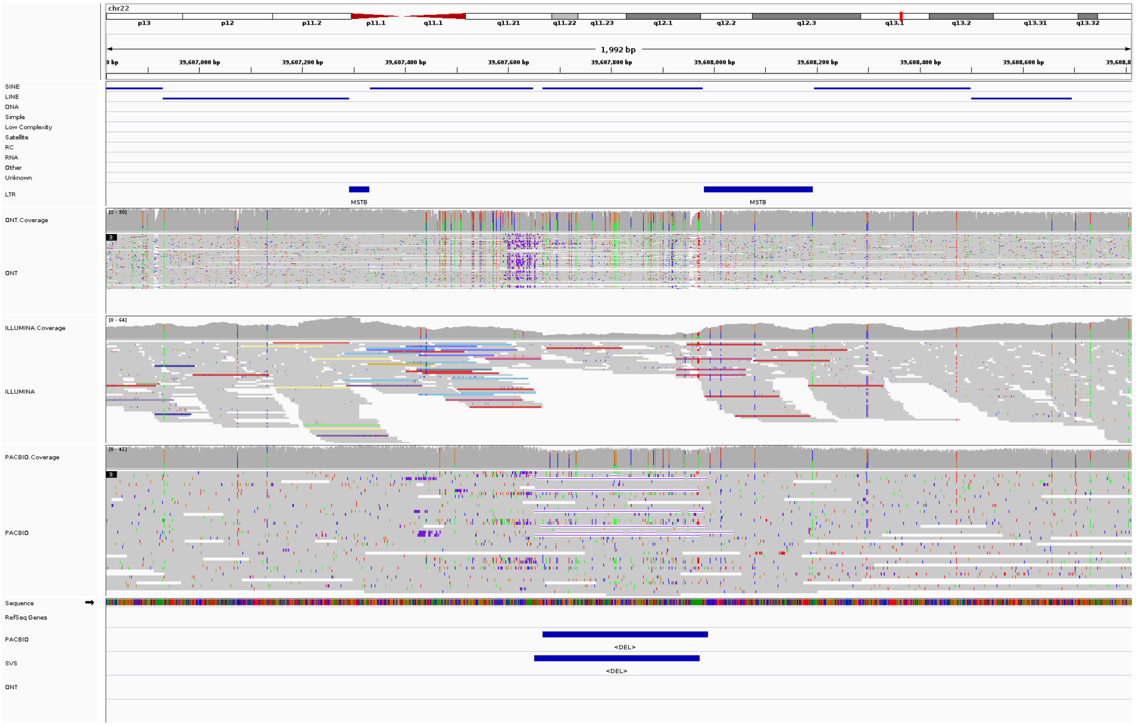

Supplementary Figure 23: Subfigure 7

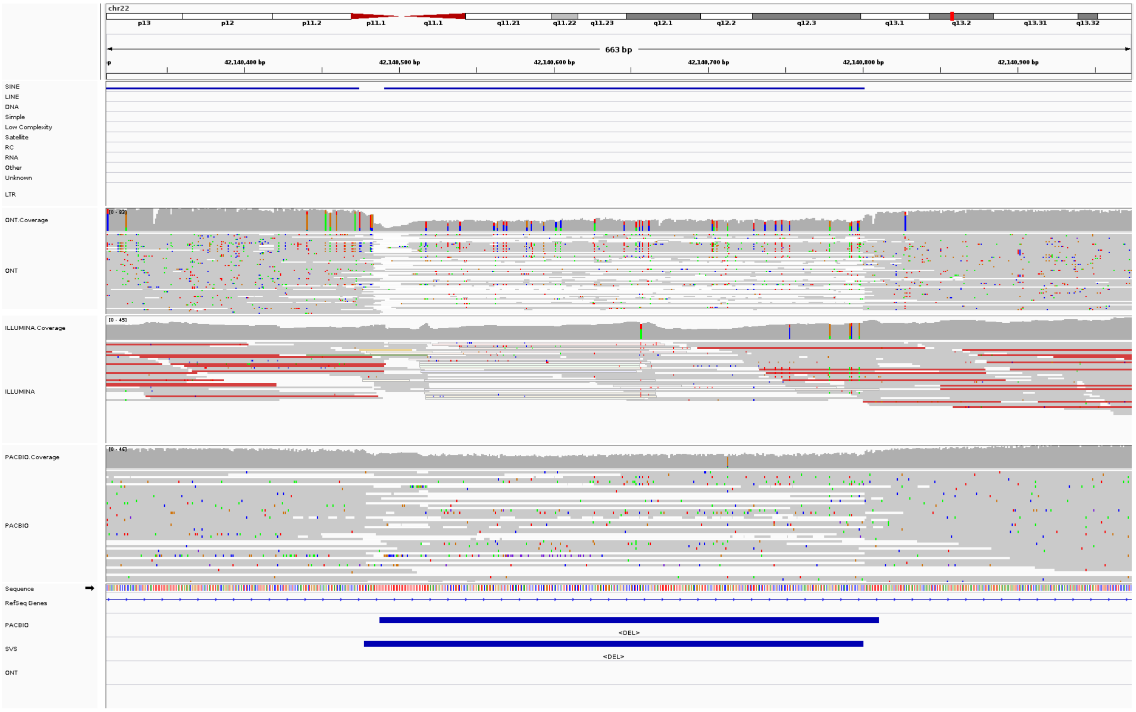

Supplementary Figure 23: Subfigure 8

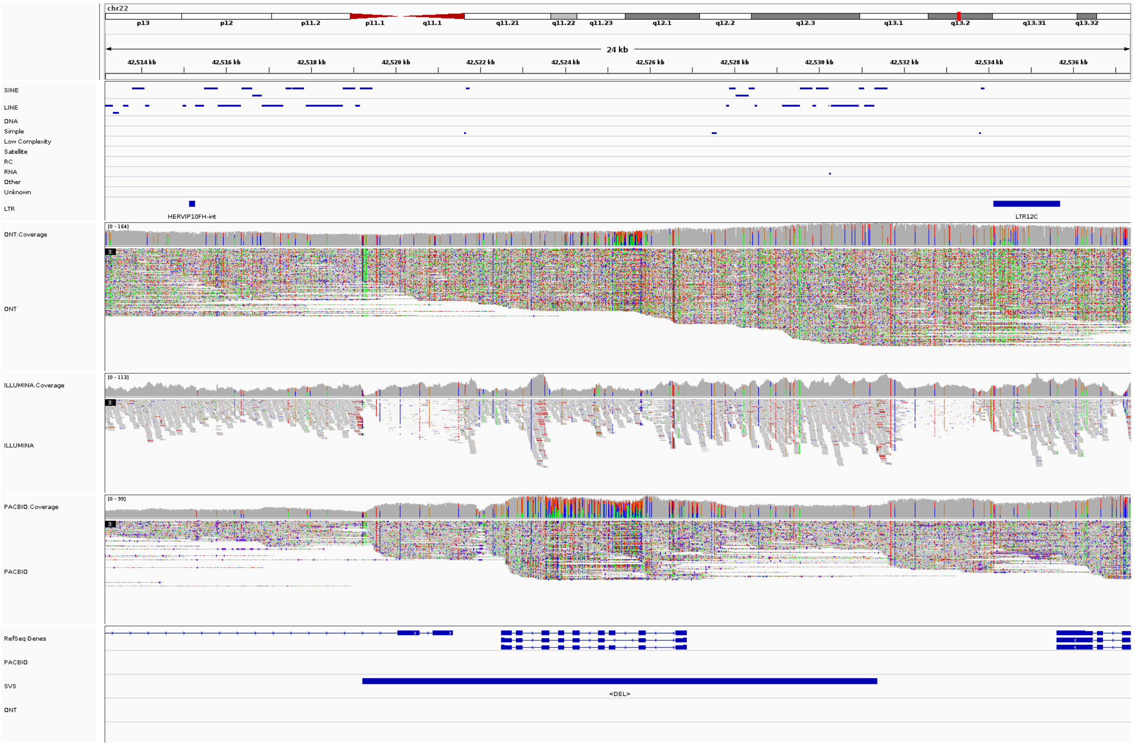

Supplementary Figure 23: Subfigure 9

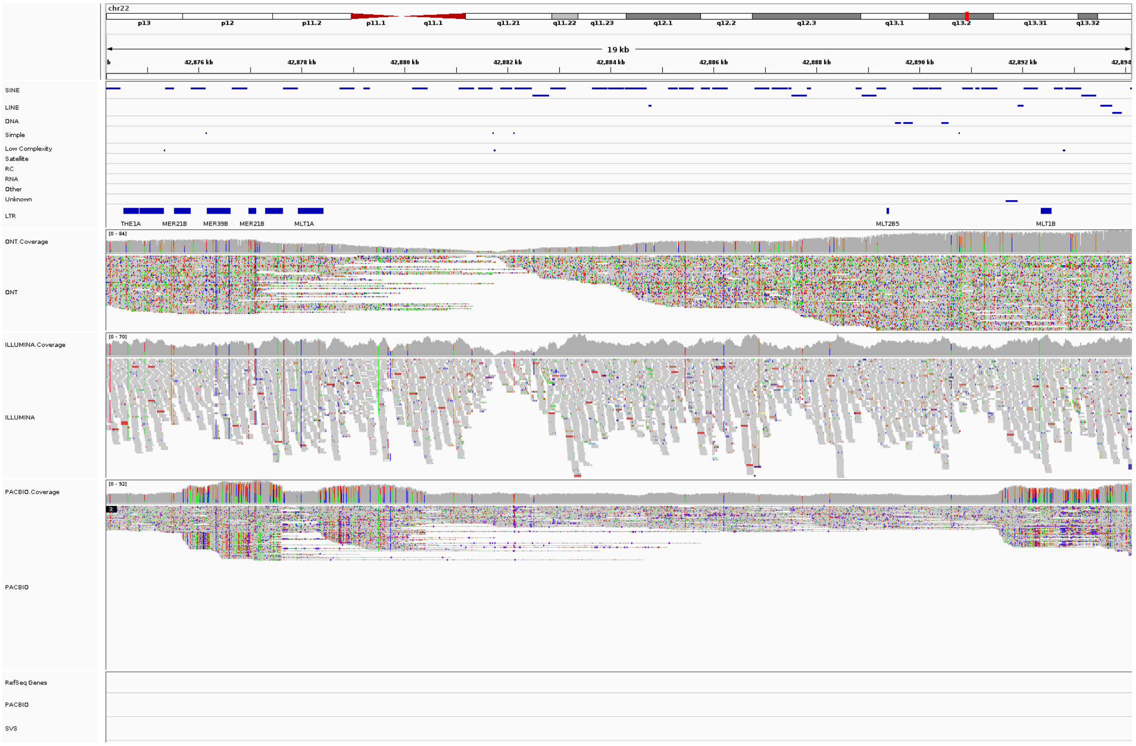

Supplementary Figure 23: Subfigure 10

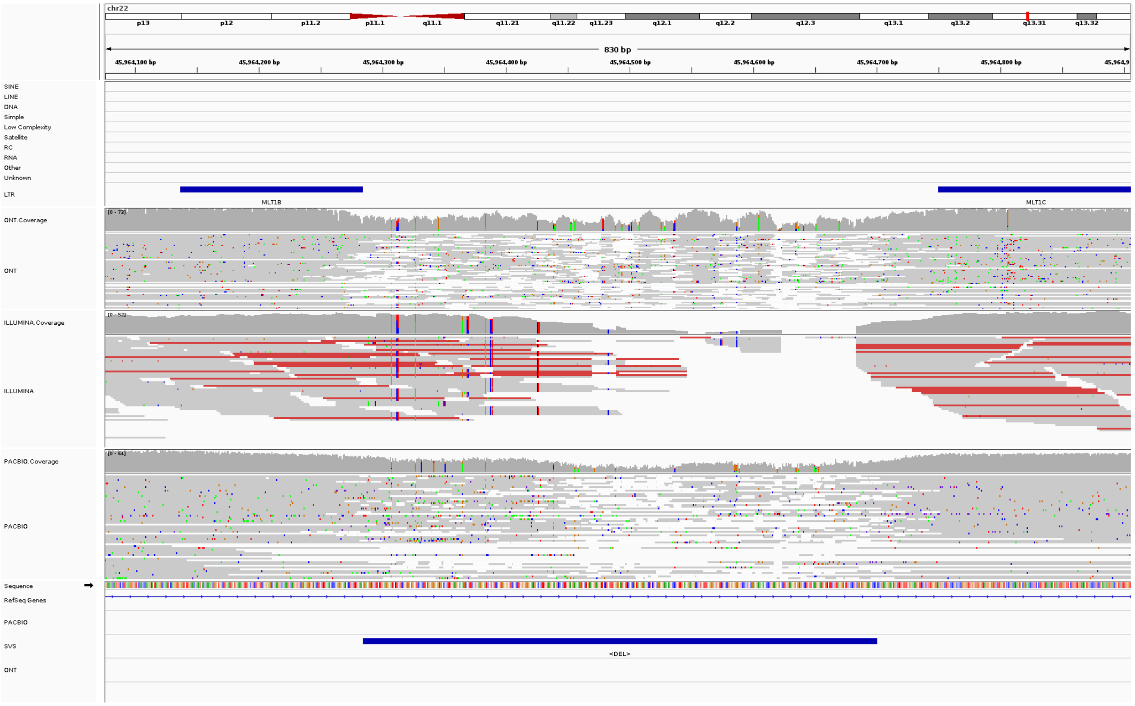

Supplementary Figure 23: Subfigure 11

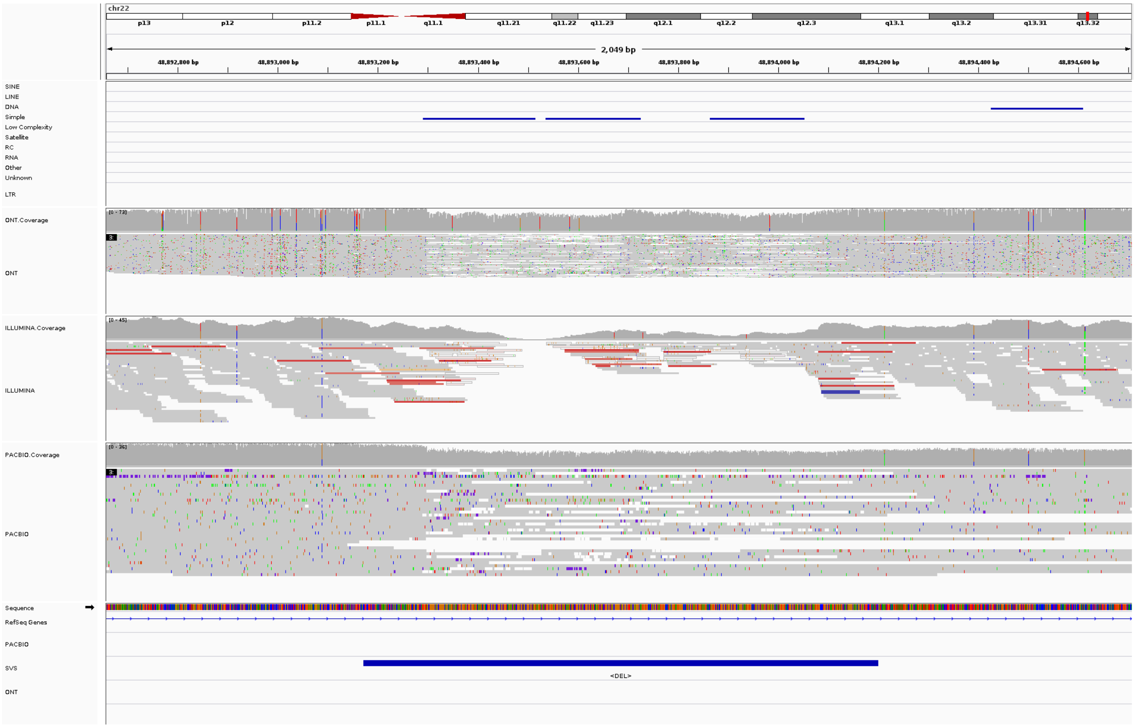

3.2.4 Large variant calls in reference data not present in ONT data

This section shows large variant calls that are present in the reference data set, but which have not been called using our method and have very little support in ONT data. Based on visual inspection we believe these calls to be true negatives.

Supplementary Figure 24: Subfigure 1

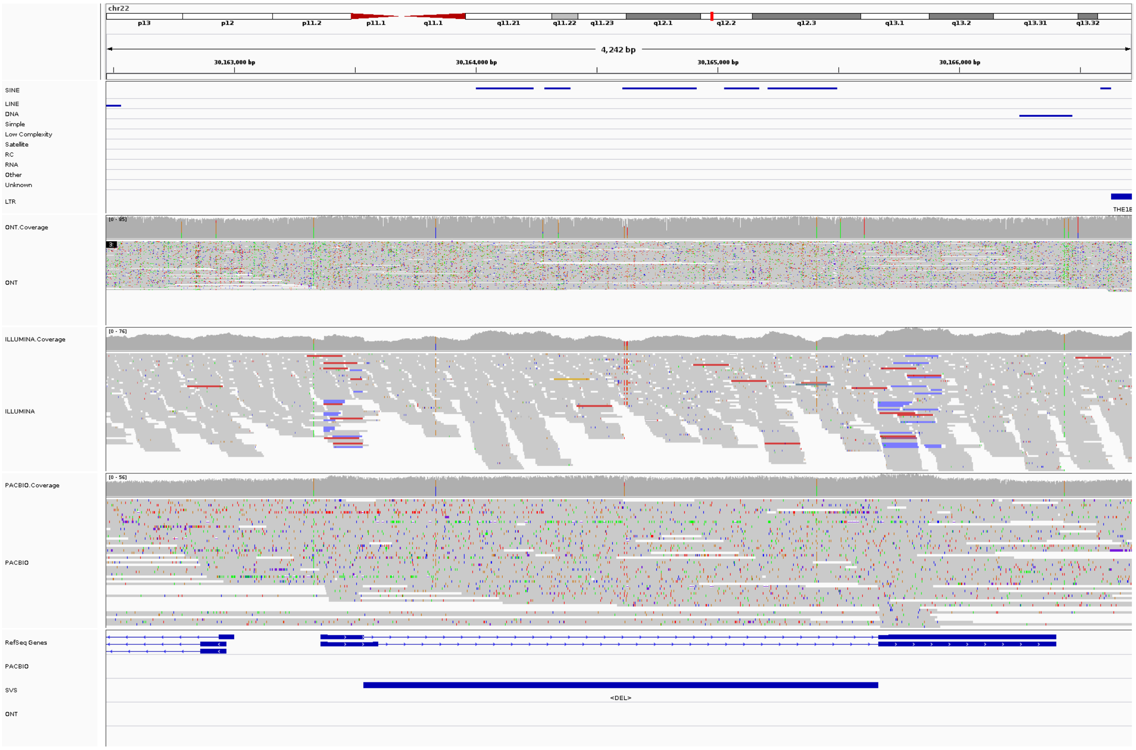

Supplementary Figure 24: Subfigure 2

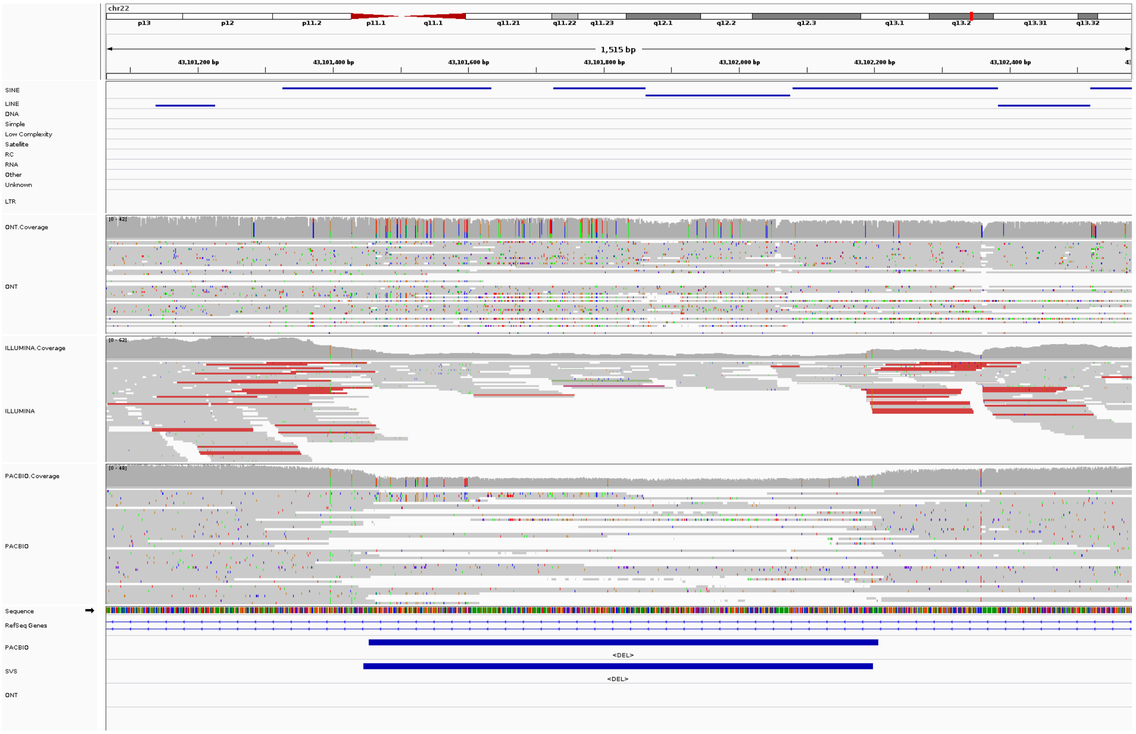

Supplementary Figure 24: Subfigure 3

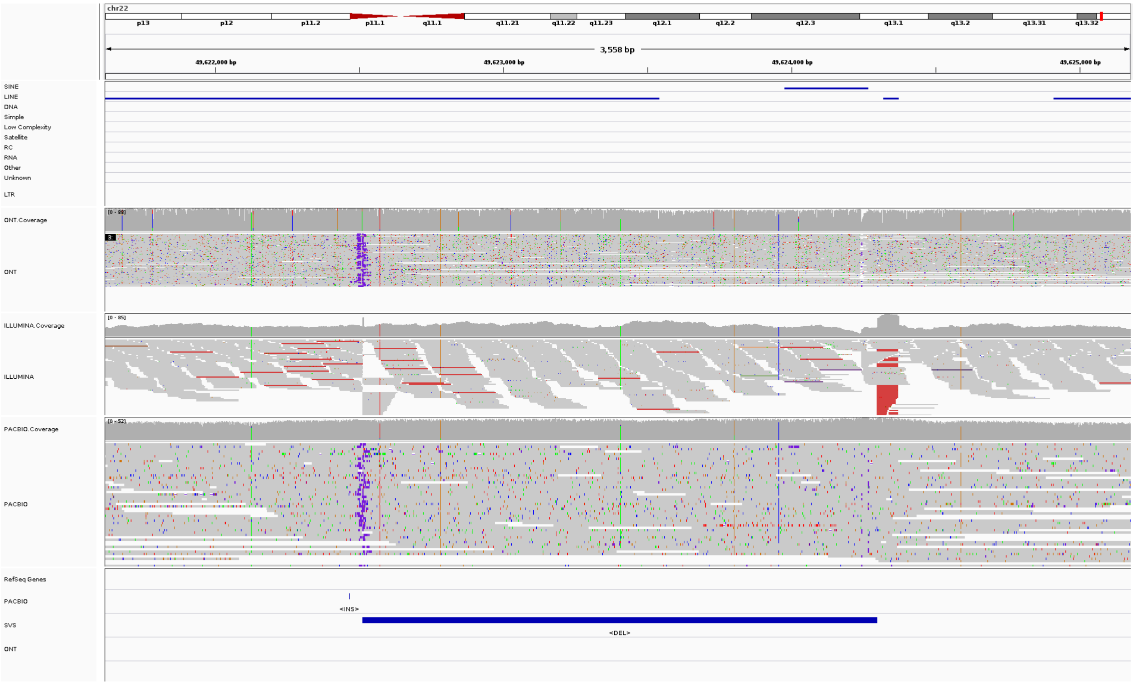

3.3 Supplementary Figures - Clinical sample

Supplementary Figure 25: Proportion of PASS/FAIL reads in clinical sample Proportion of reads per flowcell that meet the minimum required read length (1000 bp) and minimum average base quality score (14) (PASS) or do not (FAIL), sorted by flowcell run date.

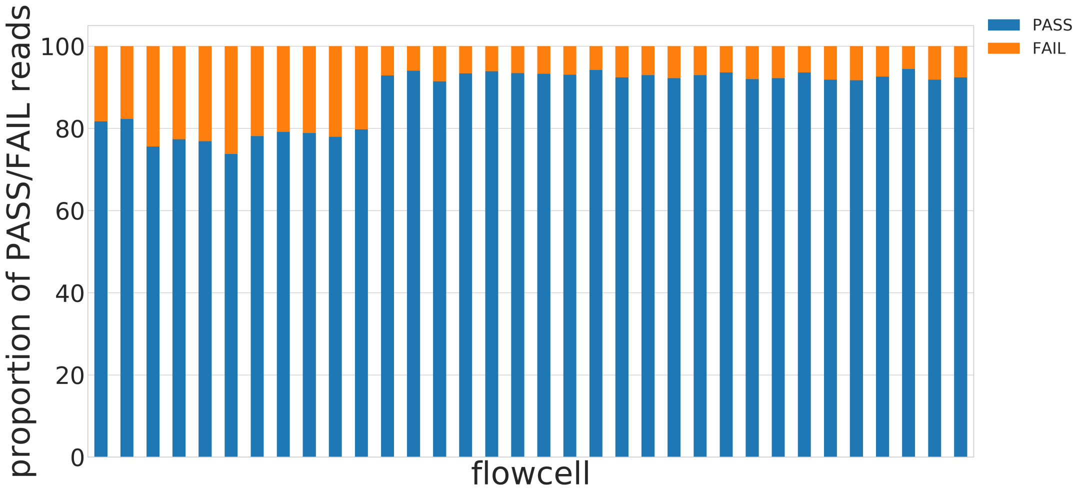

Supplementary Figure 26: Sequencing yield per flowcell in clinical sample

li

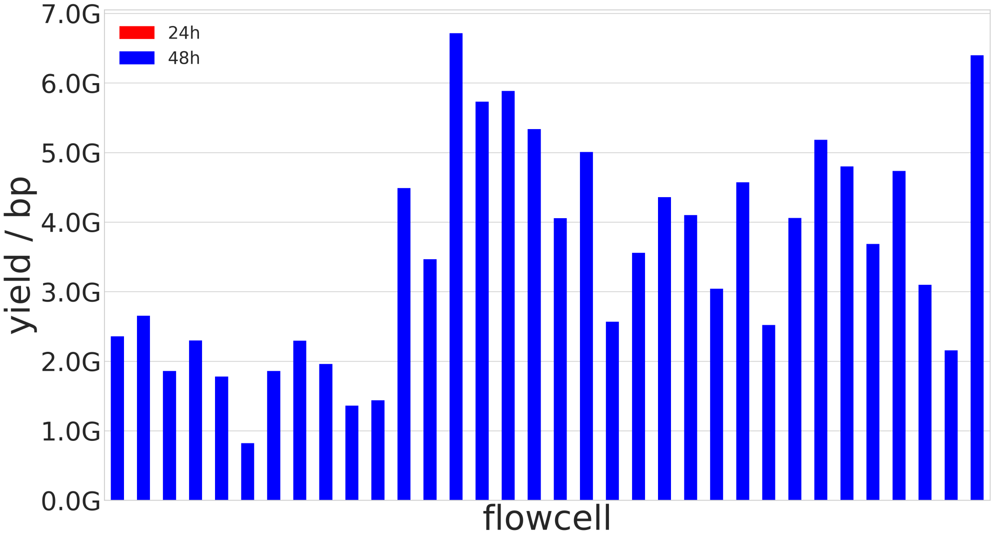

Supplementary Figure 27: Average read length per flowcell Flowcells are sorted by run date. The average mapped read length is the average length of reads in the PASS set and that have been mapped to the reference genome

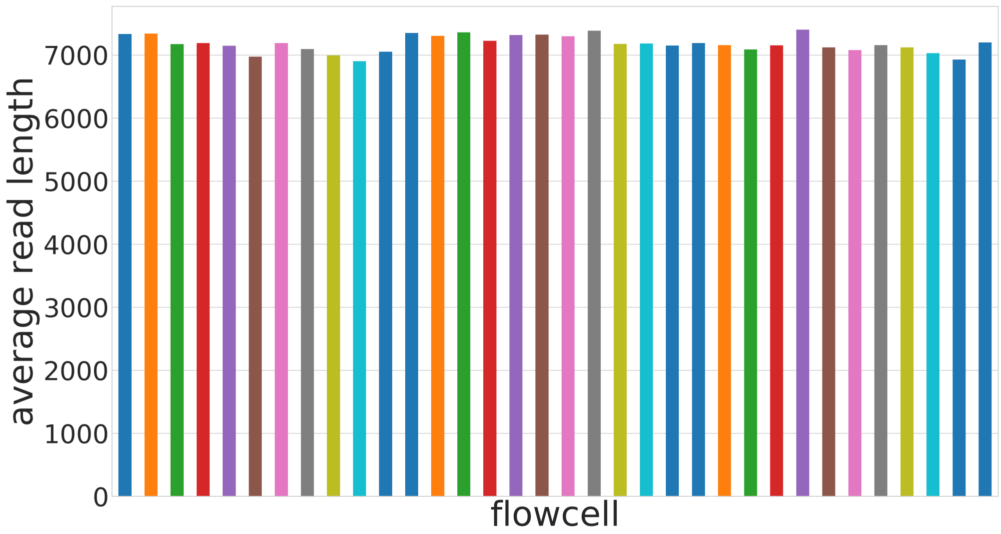

Supplementary Figure 28: Mapping status of reads from clinical sample Yield (number of reads) per flowcell. The total size of the bar represents the number of reads from each flow cell and is split into the proportion of reads that have been mapped in a single alignment (single alignment), mapped in multiple alignments (multiple alignments), have been base-called, but not been mapped (unmapped) and reads that have not been base-called (not base-called).

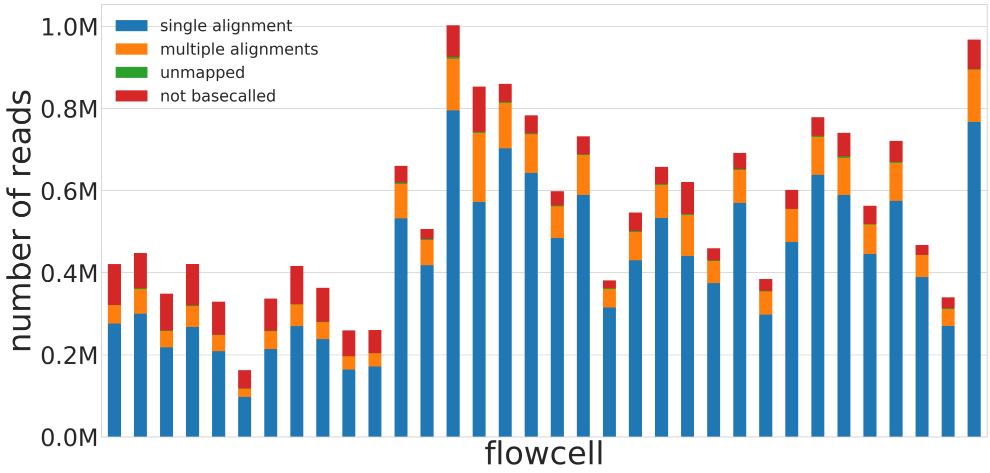

Supplementary Figure 29: Substitution error rates in clinical sample Different colours correspond to different flow cells.

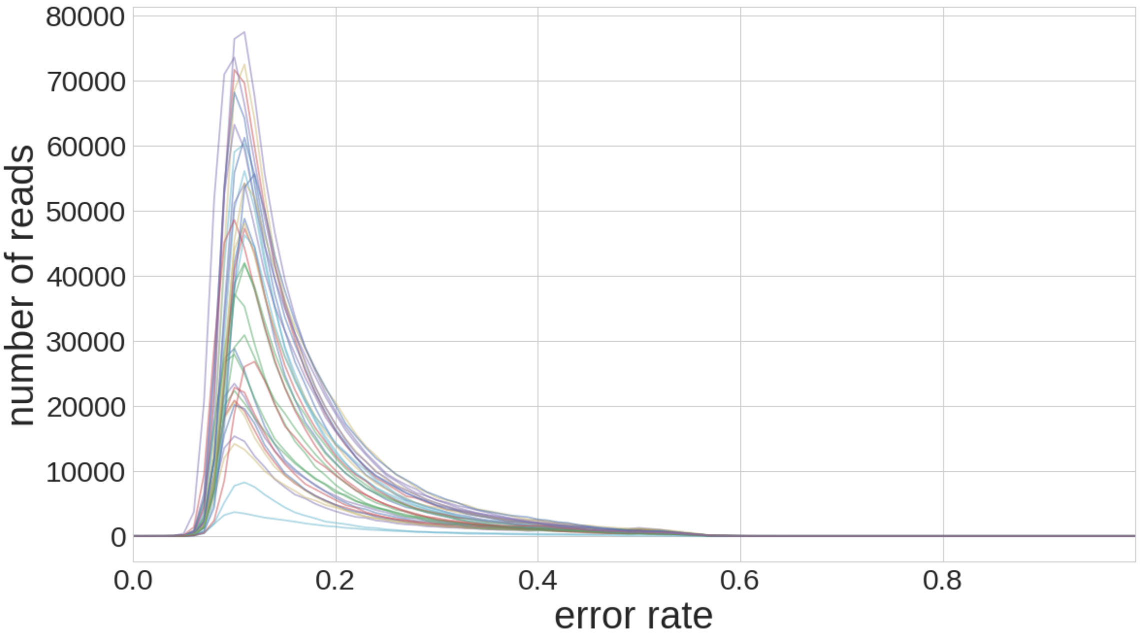

Supplementary Figure 30: Distribution of genomic coverage in clinical sample Depth is the number of read bases than are aligned to a particular base in the reference genome.

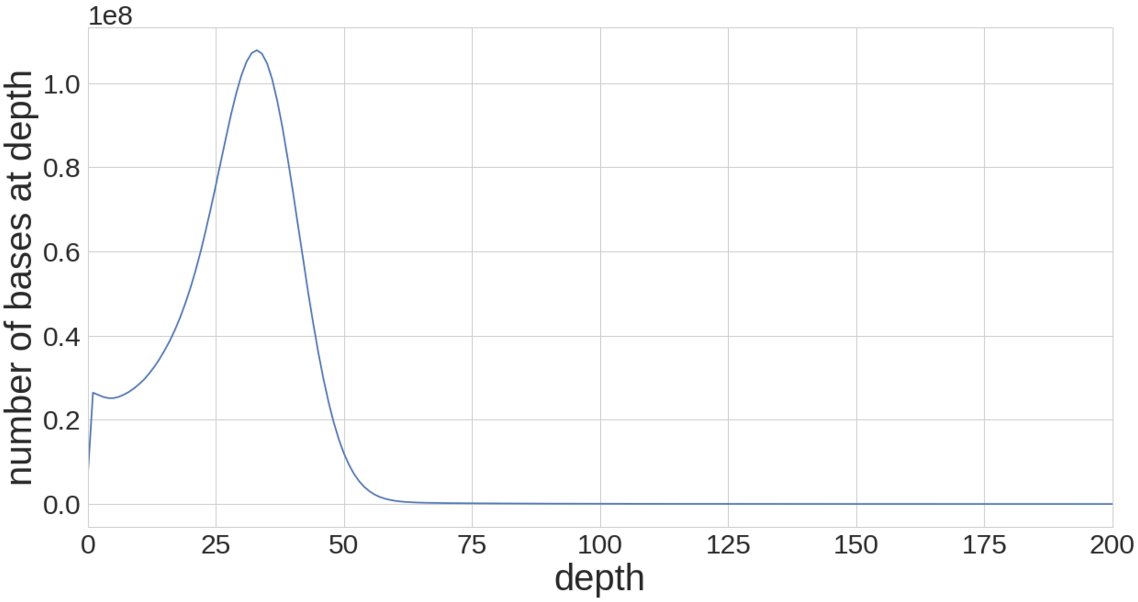

Supplementary Figure 31: Substitution rates in reads spanning variants of interest in SAMD9L Shown is the subsitution rate per read of reads spanning the two variants c.1076G>A and c.3353A>G. Reads are grouped into reads supporting a cis arrangement of the variants, reads supporting a trans arrangement of the variants, and reads where the base is neither the reference nor the alternate variant at either of the sites.

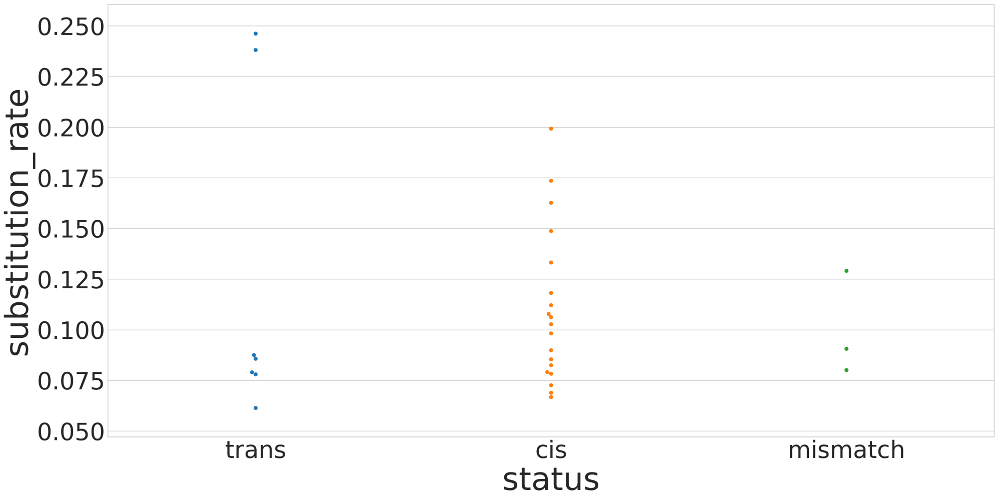

Supplementary Figure 32: Phasing variants using allele-specific PCR A) Strategy 1 is explained in the schematic diagram and employed a semi-nested PCR format. The first-round PCR used a universal R primer in tandem with one of two different F primers where the 3'-terminal base was either a T or a C, i.e. complementary to either the reference or alternate base at the site of the first *de novo* mutation (chr7:92,761,932T>C). The second-round of PCR used primers FN and R. PCR amplicons were purified using the exoSAP method and Sanger sequenced using BigDye3.1 chemistry. Where the "932C" primer had been used which is complementary to the mutant allele at the first *de novo* site, the predominant peak at the site of the second *de novo* mutation corresponds to the mutant T allele (red arrow). Where the "932T" (reference) primer had been used, it is predominantly the reference C allele seen at the second site. B) Strategy 2 is the same as Strategy 1 but in reverse. Allele-specific primers were designed at the site of the second mutation (chr7:92,764,209C>T). Again, it is predominantly the mutant C allele observed (blue arrow) at the site of the first *de novo* site where the "209T" (mutant) primer had been used. The genomic coordinates shown are based on the hg19 build.

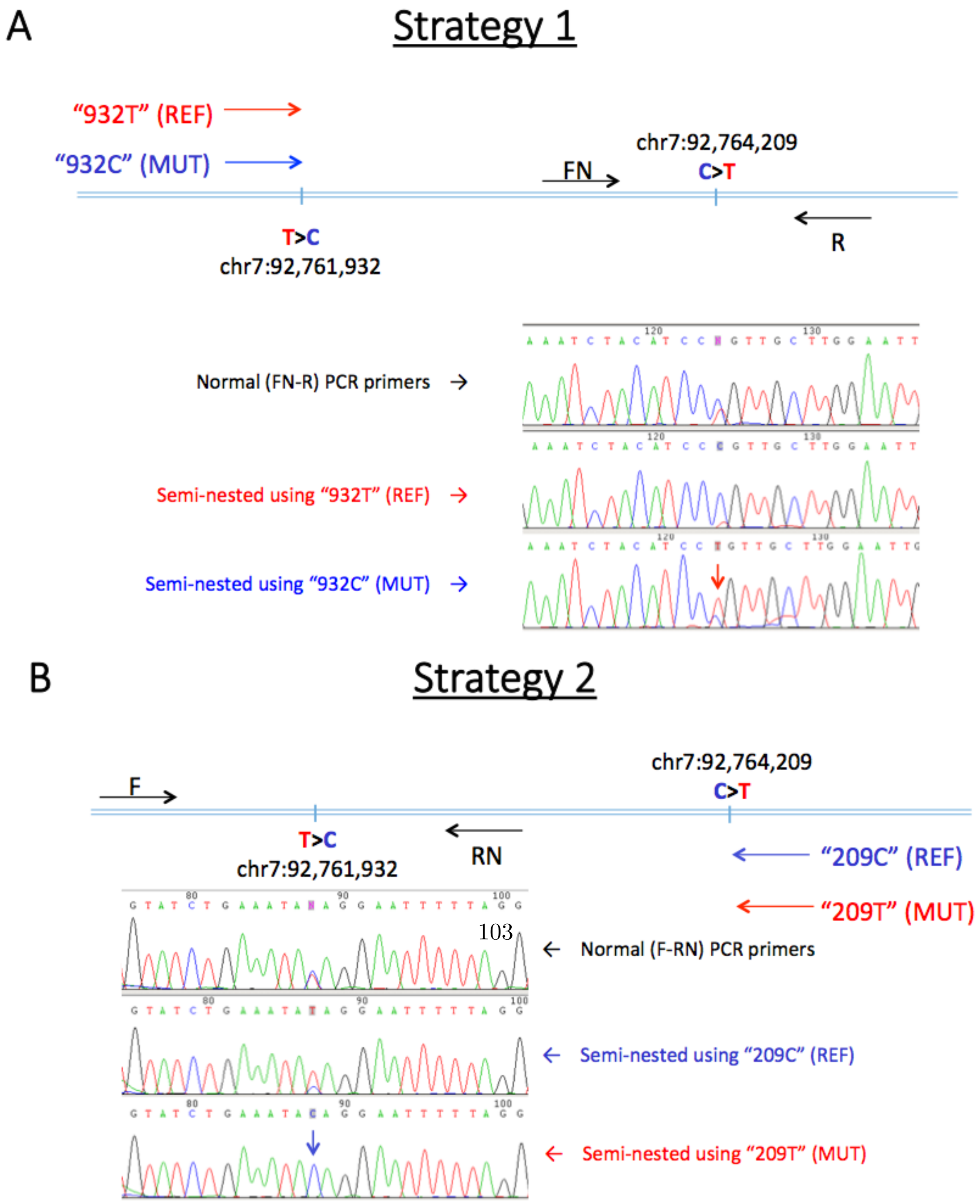

4 References

[1] Davies RW, Flint J, Myers S, Mott R. Rapid genotype imputation from sequence without reference panels. Nature Genetics. 2016 Aug;48(8):965–969. Available from: http://www.nature.com/ng/journal/v48/n8/abs/ng.3594.html.
