# Supplementary material for: Sequencing of human genomes with nanopore technology
Source: Nat Commun. 2019 Apr 23;10:1869. doi: 10.1038/s41467-019-09637-5 (PMC6478738; doi:10.1038/s41467-019-09637-5)
Supplement: Supplementary file 2 — Reporting Summary [file 41467_2019_9637_MOESM2_ESM.pdf]

nature research

Corresponding author(s): *Double-blind peer review submissions: write DBPR and your manuscript number here instead of author names.*

Last updated by author(s): *YYYY-MM-DD*

Reporting Summary

Nature Research wishes to improve the reproducibility of the work that we publish. This form provides structure for consistency and transparency in reporting. For further information on Nature Research policies, see Authors & Referees and the Editorial Policy Checklist.

Statistics

For all statistical analyses, confirm that the following items are present in the figure legend, table legend, main text, or Methods section.

n/a	Confirmed	
☒	☐	The exact sample size (*n*) for each experimental group/condition, given as a discrete number and unit of measurement
☒	☐	A statement on whether measurements were taken from distinct samples or whether the same sample was measured repeatedly
☒	☐	The statistical test(s) used AND whether they are one- or two-sided<br>*Only common tests should be described solely by name; describe more complex techniques in the Methods section.*
☒	☐	A description of all covariates tested
☒	☐	A description of any assumptions or corrections, such as tests of normality and adjustment for multiple comparisons
☒	☐	A full description of the statistical parameters including central tendency (e.g. means) or other basic estimates (e.g. regression coefficient) AND variation (e.g. standard deviation) or associated estimates of uncertainty (e.g. confidence intervals)
☒	☐	For null hypothesis testing, the test statistic (e.g. *F*, *t*, *r*) with confidence intervals, effect sizes, degrees of freedom and *P* value noted<br>*Give P values as exact values whenever suitable.*
☒	☐	For Bayesian analysis, information on the choice of priors and Markov chain Monte Carlo settings
☒	☐	For hierarchical and complex designs, identification of the appropriate level for tests and full reporting of outcomes
☒	☐	Estimates of effect sizes (e.g. Cohen's *d*, Pearson's *r*), indicating how they were calculated

Our web collection on statistics for biologists contains articles on many of the points above.

Software and code

Policy information about availability of computer code

Data collection	Instrument control: MinKNOW versions 1.1.15-1.1.21 NC_48Hr_Sequencing_Run_FLO-MIN106_SQK-LSK108 script<br>Basecalling: Albacore v2.0.2 using the R9.4 450bps linear config
Data analysis	samtools mpileup<br>Freebayes version v1.0.2-16-gd466dde using options --no-indels --use-best-n-alleles 4 -C 5 --no-mnps --no-complex --haplotype-length 0 --pooled-continuous<br>PBSIM 42, Version 1.0.3 (5ae589d1)) --accuracy-min=0.40 --accuracy-sd=0.20<br>RTG vcfeval version 3.5.1<br>BLASR (v1.3.2)<br>svclassify<br>Exac version 0.3.1<br>SEW version 1.0.0 https://github.com/Genomicsplc/SEW<br>whatshap version 0.13+2.gf854c0b.dirty

For manuscripts utilizing custom algorithms or software that are central to the research but not yet described in published literature, software must be made available to editors/reviewers. We strongly encourage code deposition in a community repository (e.g. GitHub). See the Nature Research guidelines for submitting code & software for further information.

Data

Policy information about availability of data

All manuscripts must include a data availability statement. This statement should provide the following information, where applicable:

- Accession codes, unique identifiers, or web links for publicly available datasets
- A list of figures that have associated raw data
- A description of any restrictions on data availability

Sequence data that support the findings of this study have been deposited in ENA (NA12878) with accession PRJEB30620 [https://www.ebi.ac.uk/ena/data/view/

PRJEB30620] and in EGA (clinical sample) with the accession EGAS00001003469 [https://www.ebi.ac.uk/ega/studies/EGAS00001003469]. All other relevant data is available upon request.

Field-specific reporting

Please select the one below that is the best fit for your research. If you are not sure, read the appropriate sections before making your selection.

☒ Life sciences ☐ Behavioural & social sciences ☐ Ecological, evolutionary & environmental sciences

For a reference copy of the document with all sections, see nature.com/documents/nr-reporting-summary-flat.pdf

Life sciences study design

All studies must disclose on these points even when the disclosure is negative.

Sample size	The aim was to study in depth one sample that had previously been studied extensively by other methods, to compare data across a large (genomic) scale, and to study one original sample in order to demonstrate a particular example of the utility of the method.
Data exclusions	Only data (sequence reads) that did not meet the device's internally defined quality standards were excluded.
Replication	In genome sequencing, replication exists in the average number of reads of each genome position that are obtained. These depths of coverage (82x and 30x respectively) were assessed in terms of the accuracy of data that they yielded.
Randomization	The order in which the experiments were done is irrelevant to a study of this type.
Blinding	Blinding was irrelevant to this study because the investigators could not influence the detailed form of the data being produced, and a comparison of the amount and quality of data was not relevant to the analysis.

Reporting for specific materials, systems and methods

We require information from authors about some types of materials, experimental systems and methods used in many studies. Here, indicate whether each material, system or method listed is relevant to your study. If you are not sure if a list item applies to your research, read the appropriate section before selecting a response.

Materials & experimental systems

n/a	Involved in the study	
☒	☐	Antibodies
☒	☐	Eukaryotic cell lines
☒	☐	Palaeontology
☒	☐	Animals and other organisms
☐	☒	Human research participants
☒	☐	Clinical data

Methods

n/a	Involved in the study	
☒	☐	ChIP-seq
☒	☐	Flow cytometry
☒	☐	MRI-based neuroimaging

Human research participants

Policy information about studies involving human research participants

Population characteristics	There are no relevant population statistics
Recruitment	The research participants were a family trio recruited to a larger study of potential genomic causes of sporadic disease and selected for this study because they exemplified a particular situation that we wished to illustrate.
Ethics oversight	West Midlands REC, UK

Note that full information on the approval of the study protocol must also be provided in the manuscript.
